# Identifying novel fruit-related genes in *Arabidopsis thaliana* based on the random walk with restart algorithm

**DOI:** 10.1371/journal.pone.0177017

**Published:** 2017-05-04

**Authors:** Yunhua Zhang, Li Dai, Ying Liu, YuHang Zhang, ShaoPeng Wang

**Affiliations:** 1 School of Resources and Environment, Anhui Agricultural University, Hefei, China; 2 School of Forestry and Landscape Architecture, Anhui Agricultural University, Hefei, China; 3 Institute of Health Sciences, Shanghai Institutes for Biological Sciences, Chinese Academy of Sciences, Shanghai, China; 4 School of Life Sciences, Shanghai University, Shanghai, China; Tianjin University, CHINA

## Abstract

Fruit is essential for plant reproduction and is responsible for protection and dispersal of seeds. The development and maturation of fruit is tightly regulated by numerous genetic factors that respond to environmental and internal stimulation. In this study, we attempted to identify novel fruit-related genes in a model organism, *Arabidopsis thaliana*, using a computational method. Based on validated fruit-related genes, the random walk with restart (RWR) algorithm was applied on a protein-protein interaction (PPI) network using these genes as seeds. The identified genes with high probabilities were filtered by the permutation test and linkage tests. In the permutation test, the genes that were selected due to the structure of the PPI network were discarded. In the linkage tests, the importance of each candidate gene was measured from two aspects: (1) its functional associations with validated genes and (2) its similarity with validated genes on gene ontology (GO) terms and KEGG pathways. Finally, 255 inferred genes were obtained, subsequent extensive analysis of important genes revealed that they mainly contribute to ubiquitination (UBQ9, UBQ8, UBQ11, UBQ10), serine hydroxymethyl transfer (SHM7, SHM5, SHM6) or glycol-metabolism (HXKL2_ARATH, CSY5, GAPCP1), suggesting essential roles during the development and maturation of fruit in *Arabidopsis thaliana*.

## Introduction

Fruit, as specialized seed-bearing structures, are designed to protect the reproductive organs of plants [1]. During the development and maturation of seeds, fruit has been confirmed to play a specific role. Based on recent publications, there are two main functions of fruit that may contribute to the reproductive processes of plants [2]. First, fruits protect seeds. For most angiosperms, fruits serve as a solid physical barrier between seeds and the external environment, providing a reliable shelter for seed development and maturation [2, 3]. Second, some fruits may contribute to the dispersal of mature seeds. Not all mature seeds of a plant can grow into fertile plants. During the germination and growing processes of a mature seed, the surrounding environment is highly significant [4, 5]. During transfer of the seed to a proper environment for further germination and growth, fruits play a crucial role [5]. Take coffee as an example. The solid fruit of coffee berries protect the seed inside from the diverse external environment [6, 7]. Digestion by a specific civet cat (*Paradoxurus hermaphroditus*) can destroy the fruit and expose the seeds, which also may complete the dispersal process [8]. Considering fruit is crucial for the plant reproduction, it is important to identify fruit-associated regulatory factors. Among such factors, specific intrinsic factors, especially genetic factors, have been confirmed to play an irreplaceable role during the development and maturation of fruit.

*Arabidopsis thaliana* is an annual plant native to Eurasia, and has a height of 20–25 cm [9]. First described in 1577 by Johannes Thal, *Arabidopsis thaliana* has unique advantages for use as a model plant. At first, *Arabidopsis thaliana* has an appropriate size (7–40 cm), which not only is suitable for morphologic observation but also enables large-scale indoor cultivation [10]. Furthermore, the reproductive capacity of Arabidopsis is robust, enabling scientists to obtain many plant seedlings within a short time [10]. Further, as a self-pollinated plant, the fertilization process of *Arabidopsis thaliana* can be easily interfered with and controlled to meet the needs of biological experiments, avoiding other external environmental factors and making it a perfect model organism for genetics research [11]. Finally, the whole genome of *Arabidopsis thaliana*, the smallest among cruciferous plants, only contains five chromosomes and 100 million base pairs that have been completely sequenced [12]. Considering these advantages, *Arabidopsis thaliana* is an optimal model organism and has contributed greater understanding in genetics and botany.

As we have analyzed above, fruit associated biological processes are regulated by multiple internal and external regulatory factors in multiple plant subtypes, including *Arabidopsis thaliana*, among which genetic factors play a specific critical role. Considering the advantages of *Arabidopsis thaliana* as a typical model plant, as we have analyzed above, it is reasonable to take such plant as a model for further study on fruit associated genes. In *Arabidopsis thaliana*, a self-pollinated plant, various genes contribute to regulation of fruit development. For example, *KANADI1* and *KANADI2* participate in the development of lateral organs [13]. However, it is expensive and time-consuming to identify functional fruit-associated genes in *Arabidopsis thaliana* by traditional experimental methods. On the other hand, with the development of computer technology and its successful application in the fields of biology, medicine [14–19], it becomes possible to develop reliable computational methods for the identification of fruit-associated genes.

Here, based on the validated fruit-related genes in *Arabidopsis thaliana*, we tried to identify novel fruit-related genes using computational techniques. Up to now, several network methods have been developed to identify novel disease genes [15, 20–25]. Some of these methods employed the classic network algorithms, such as random walk with restart (RWR) algorithm [26, 27] and shortest path algorithm [28]. These algorithms gave novel directions for identification of functional fruit-associated genes. Recently, Zhu *et al*. built a method that used the shortest path algorithm as the basic search algorithm to identify novel fruit-associated genes in *Arabidopsis thaliana* [29]. In this study, we adopted the RWR algorithm to construct the method. In detail, it was executed on a protein-protein interaction (PPI) network, which was constructed using PPI information in STRING [30], with validated genes as seed nodes. Genes with high probabilities were selected. To identify false positives, the permutation test and linkage tests were built to screen out essential genes. The permutation test can discard genes selected due to the structure of the PPI network. In the linkage tests, we measured the importance of each candidate gene from two points: (1) its functional associations with validated genes and (2) its similarity with validated genes on gene ontology (GO) terms [31] and Kyoto encyclopedia of genes and genomes (KEGG) pathways [32]. A group of inferred genes were accessed, some of which were extensively analyzed. Our results indicate that these genes may participate in fruit-associated biological processes of *Arabidopsis thaliana*.

## Materials and methods

### Dataset

To obtain the validated fruit-related genes in *Arabidopsis thaliana*, we first accessed fruit-related PO terms. A file named plant_ontology.obo (accessed on March 24, 2005) was downloaded from Plant Ontology (PO, http://www.plantontology.org/download) [33]. This file provided easy-to-retrieve structures of PO terms. Terms containing fruit (PO: 0009001) and its children terms (PO: 0004707, PO: 0008001, PO: 0004536, PO: 0004535, PO: 0008002, PO: 0025268, PO: 0000033, PO: 0008003, PO: 0009087, and PO: 0009084) were extracted and regarded as fruit-related PO terms. The descriptions of these PO terms are listed in Table 1. Accordingly, 994 genes annotated by these PO terms were accessed and considered as the validated fruit-related genes. The IDs of these 994 genes are provided in S1 Table.

**Table 1:** The information of fruit-related PO terms.

PO term ID	Description
PO:0009001	fruit
PO:0004707	fruit dehiscence zone
PO:0008001	fruit distal end
PO:0004536	fruit pedicel
PO:0004535	fruit placenta
PO:0008002	fruit proximal end
PO:0025268	fruit septum
PO:0000033	fruit valve
PO:0008003	fruit vascular system
PO:0009087	mesocarp
PO:0009084	pericarp

### PPI network

Because the functions of proteins need some factors to regulate, intercellular and intracellular proteins rarely execute their functions alone. Thus, the PPIs were essential for the normal metabolism of organisms. Some computational methods have been developed to identify novel PPIs [34–37], which can yield more abundant PPIs to investigate related problems. The accumulated information of PPIs can be used to construct a large PPI network, which is helpful to investigate different properties of proteins, such as protein functions [38–40], relationship with different diseases [21, 22, 41–45].

In this study, we used the PPIs of *Arabidopsis thaliana* reported in STRING [30] (http://string-db.org/, Version 9.1), a well-known public database collecting PPIs of several organisms, to construct the PPI network. PPIs in STRING are derived from genomic context, high throughput experiments, (conserved) co-expression, and previous knowledge, indicating they can measure both the direct (physical) and indirect (functional) associations between proteins. To retrieve the PPIs of *Arabidopsis thaliana*, a file ‘protein.links.v9.1.txt.gz’ was downloaded from STRING, which contains PPIs of 1,133 organisms covering 5,214,234 proteins. Because ‘3702’ is the organism code of *Arabidopsis thaliana* in STRING, lines starting with ‘3702’ in the obtained file were extracted, obtaining 3,123,482 PPIs covering 25,123 proteins of *Arabidopsis thaliana*. In each PPI, there are two Ensembl IDs and a score ranging between 150 and 999. For formulation, the score of a PPI with proteins *p*_*a*_ and *p*_*b*_ was denoted by *S*_*I*_(*p*_*a*_, *p*_*b*_). The constructed PPI network *G* defined 25,123 proteins as nodes and each edge represented one PPI, i.e., two nodes were adjacent if and only if their corresponding proteins can constitute a PPI. In addition, the score of each interaction was assigned to the corresponding edge as its weight.

### RWR algorithm

The RWR algorithm [46, 47] is a classic ranking algorithm. This algorithm stimulates a walker that starts from a seed node or some seed nodes and randomly moves in a network. Here, 994 fruit-related genes were deemed as seed nodes, and the RWR algorithm was applied on the PPI network *G* to discover possible novel fruit-related genes.

Before executing the RWR algorithm on *G*, an initialization vector *P*_0_ was constructed containing 25,123 components that corresponded to 25,123 nodes in the PPI network *G*. In *P*_0_, the component corresponding to the validated fruit-related genes was set to 1/994 and others were set to zero. The RWR algorithm repeatedly updates this vector and denotes *P*_*i*_ as the vector after the *i*-th round has been done. The updating rule is as follows:

$$P_{i+1}=A^{T}P_{i}+cP_{0}$$ (1)

where ***A*** is the column normalized adjacency matrix (the sum of members in each column equals to one) of the PPI network *G* and *c* is the restart probability (it was set to 0.8 in this study). The updating procedure stops until the vector *P*_*i*_ becomes stable, which is measured by the condition of **||** *P*_*i*+1_ –*P*_*i*_ **||**_*L*1_ < 10^−6^. The vector *P*_*i*+1_ is outputted, which indicates the probabilities of all nodes (genes) to be fruit-related genes.

Clearly, genes receiving larger probabilities are more likely to be the potential fruit-related genes. To avoid omitting too many potential genes, we set a threshold of 10^−5^, which was used in another study [24] to select possible genes, i.e., genes with probabilities larger than 10^−5^ were selected for further analysis and were called RWR genes for convenience.

### Permutation test

In Section “RWR algorithm”, the RWR algorithm was applied on the PPI network *G* using validated fruit-related genes as seed nodes, producing some RWR genes. However, not all these genes are tightly associated with the fruits of *Arabidopsis thaliana*. Some RWR genes were selected because of the structure of the PPI network, i.e., the structure of the network can influence the utility of the RWR algorithm. To discard these genes, the permutation test [43, 48–50] was designed as follows.

1. 1,000 node sets, formulated as *S*_1_, *S*_2_, *S*_3_…, *S*_1000_, were randomly constructed, and each set contained 994 nodes in the PPI network *G*;
2. For each set, the RWR algorithm was applied on the PPI network *G* using nodes in the set as seed nodes;
3. For each RWR gene, there were 1,000 probabilities produced by *S*_1_, *S*_2_, *S*_3_,…, *S*_1000_ and one probability yielded by validated fruit-related genes. Accordingly, a P-value was calculated for each RWR gene *g*, which was defined as
  P−value(g)=Θ/1000 (2)
  where Θ is the number of node sets on which the probabilities of *g* were larger than that on validated fruit-related genes. Clearly, a RWR gene with a high P-value indicates that it is not specific for fruit because several randomly produced sets can discover it. Thus, we should select RWR genes with low P-values. Because 0.05 is always selected as a cutoff of the significance level of statistical tests, it was set to be the threshold of the P-value. Thus, RWR genes with P-values less than 0.05 were selected, and the selected genes were called candidate genes for convenience.

### Linkage tests

As described in Section “Permutation test”, some candidate genes with P-values less than 0.05 were selected. These genes are deemed to have more or less associations with the fruit of *Arabidopsis thaliana*. This section built two linkage tests that can identify candidate genes with close relation to the fruit of *Arabidopsis thaliana*.

It is known that proteins that can form a PPI are often share similar functions or located in same signal pathways [39, 40, 51, 52]. Accordingly, candidate genes that can interact with at least one validated fruit-related gene are more likely to be novel fruit-related genes. Furthermore, each interaction was assigned a score ranging between 150 and 999 to indicate its strength. Thus, we can further consider this score to measure the functional associations between candidate genes and validated fruit-related genes. For each candidate gene *g*, a measurement, called maximum interaction score (*MIS*), was calculated, which is defined as:

$$MIS(g)=\text{max}\{S_{I}(g,g\prime ):g\prime \text{ is a validated fruit-related gene}\}$$ (3)

Clearly, a larger *MIS* for a candidate gene indicate tight interaction with at least one validated gene, and thus has a high likelihood to be a novel fruit-related gene. In the STRING database, 900 is the threshold of the highest confidence level of PPIs. Thus, this value was adopted as the threshold of *MIS* to screen out important candidate genes.

The GO terms [31] and KEGG pathways [32] are always utilized to elucidate and describe molecular functions, cellular components, biological and signal processes of genes. Each gene can be annotated by some GO terms or KEGG pathways. If a candidate gene exhibits similar GO terms or KEGG pathways with some validated fruit-related genes, it is more likely to be a novel fruit-related gene. To measure the similarity of candidate genes and validated genes on GO terms and KEGG pathways, the enrichment theory of GO terms and KEGG pathways was employed, based on which the relationship between a gene and GO terms or KEGG pathways can be formulated as a numeric vector [53–57]. For formulation, the vector yielded by the enrichment theory for a gene *g* was denoted by *V*(*g*). The similarity of two genes *g* and *g*′ on GO terms and KEGG pathways can be measured by the proximity of their corresponding vectors, which was computed by

$$\Gamma (g,g\prime )=\frac{V(g)\cdot V(g\prime )}{\left\|V(g)\right\|\cdot \left\|V(g\prime )\right\|}$$ (4)

Obviously, a high outcome of Eq 4 indicates a close relationship between *g* and *g*′. Like Eq 3, we can calculate a measurement, called maximum function score (*MFS*), for each candidate gene *g*, which was defined as

MFS(g)=max{Γ(g,g′):g′ is a validated fruit-related gene} (5)

A large *MFS* for a given candidate gene implies significant overlap of GO terms or KEGG pathways with at least one validated fruit-related gene. 0.9 was set to be the threshold of *MFS* in this study, i.e., candidate genes with *MFS*s larger than 0.9 were selected.

Eventually, by considering these two linkage tests, candidate genes with *MIS*s no less than 900 and *MFS*s larger than 0.9 were selected as the putative genes in this study.

## Results

**Fig 1:**
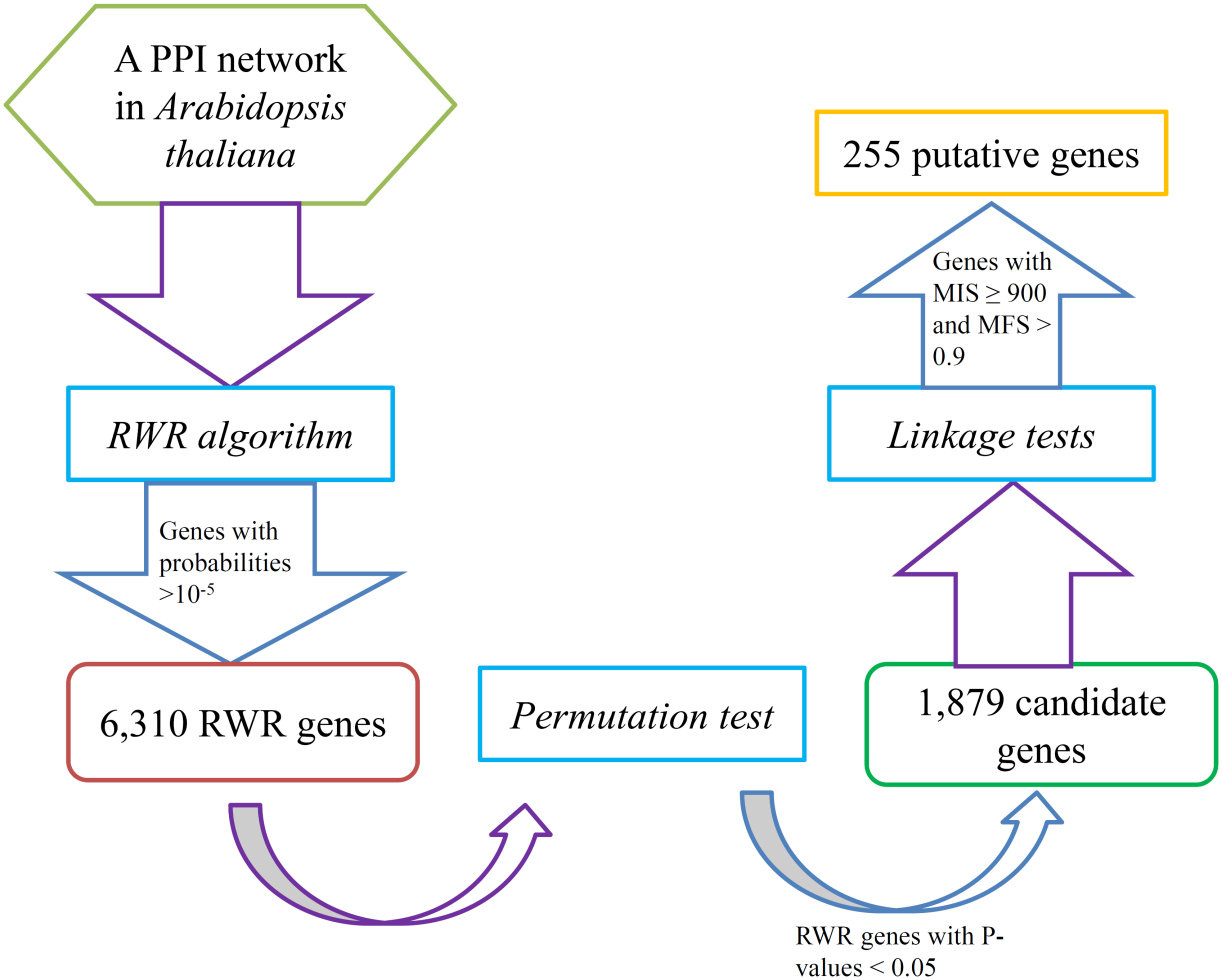
The flowchart of the method for identifying novel fruit-related genes.

A flowchart (Fig 1) illustrates the entire method used for identifying novel fruit-related genes. This chart also shows the results yielded by each procedure.

Our method first applied the RWR algorithm to the PPI network constructed in Section “PPI network” using validated fruit-related genes as seed nodes. Each gene in the network received a probability that indicated the possibility of it being a novel fruit-related gene. To reduce the searching scope, we selected genes with probabilities larger than 10^−5^, obtaining 6,310 RWR genes. These genes and their probabilities yielded by the RWR algorithm are listed in S2 Table.

Among the 6,310 RWR genes, not all are related to the fruit of *Arabidopsis thaliana*. As mentioned in Section “Permutation test”, some were selected due to the structure of the PPI network but had no relationship with the fruit of *Arabidopsis thaliana*. Thus, a permutation test was adopted to filter these types of RWR genes. A P-value was assigned to each RWR gene, which is also provided in S2 Table. Because 0.05 was selected as the criterion for statistical significance, we selected RWR genes with P-values less than 0.05, resulting in 1,879 candidate genes. These genes are available in S2 Table.

To further select essential fruit-related genes among the 1,879 candidate genes, two linkage tests were executed. Each candidate gene was assigned an MIS and an MFS, which are also available in S2 Table. The strict thresholds 900 and 0.9 for *MIS* and *MFS* were applied. 255 genes remained, which are listed in S3 Table. These genes were deemed to be significant to fruit of *Arabidopsis thaliana*. For convenience, they were called putative genes in this study.

## Discussion

Based on our computational method, we identified a group of putative genes (255 genes) that may directly or indirectly contribute to the development and maturation of the fruit in *Arabidopsis thaliana*. In another study, Zhu *et al*. adopted the shortest path algorithm to search possible fruit-related genes in *Arabidopsis thaliana* [29]. In fact, the shortest path algorithm always identifies possible genes using a pair of validated genes and collects all identified possible genes together as the candidate genes. Its principle is quite different from the RWR algorithm because RWR algorithm tries to search possible genes using the validated genes as a whole and diffusing the probabilities on validated genes to other possible genes. It is anticipated that the candidate genes obtained by these two algorithms are quite different. To prove this claim, we downloaded the identified genes in Zhu *et al*.’s study, totally 517 candidate genes. Of the 255 putative genes obtained in this study, 44 genes were also identified in Zhu *et al*.’s study and 211 genes were exclusively reported in our study (see S3 Table for detailed information). Less than one-fifth putative genes were identified in Zhu *et al*.’s study, which further proves the different influence of the shortest path algorithm and RWR algorithm for identifying possible fruit-related genes in *Arabidopsis thaliana*. Thus, the putative genes reported in this study can be an important supplement for the complete identification of fruit-related genes in *Arabidopsis thaliana*.

**Fig 2:**
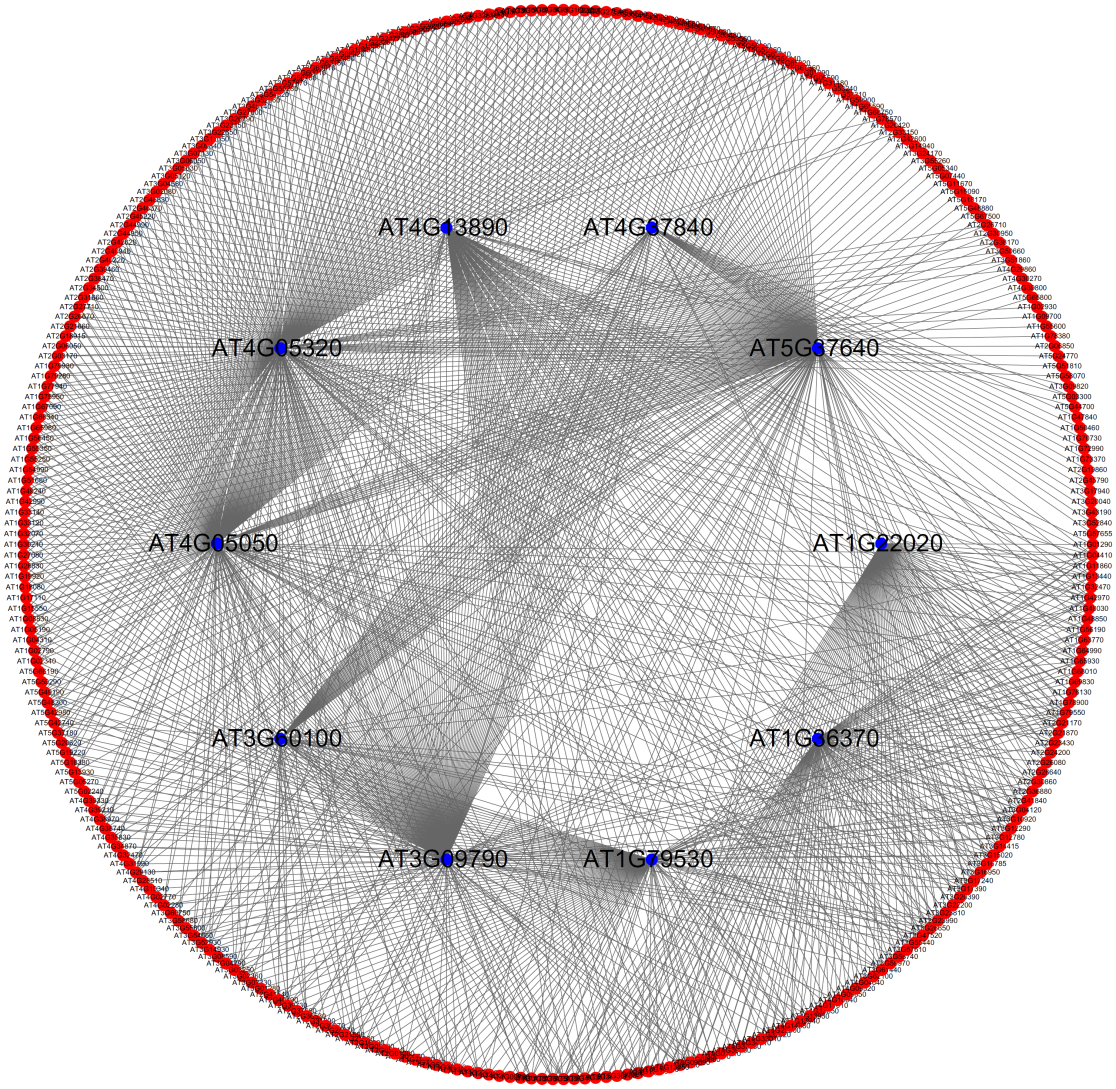
The linkages between ten important putative genes and validated fruit-related genes that were extracted from the PPI network. Red nodes represent validated fruit-related genes. Blue nodes represent putative genes.

Among the 255 putative genes, 211 genes were identified in our study and not predicted in Zhu *et al*.’ study. Of these 211 genes, some of these can be validated based upon results reported in recent publications, reflecting the accuracy and efficacy of our method. At this point in our investigation, we selected ten important putative genes (see Table 2), to analyze their relationship with the development and maturation of *Arabidopsis* fruit. The linkages between these ten putative genes and validated genes are illustrated in Fig 2. Intuitively, they all have strong associations with validated fruit-related genes, thereby inducing their close relationships with the development and maturation of the fruit in *Arabidopsis thaliana*.

**Table 2:** The detailed information of ten important putative genes.

Ensembl ID	Gene Name	Protein name	Probability	P-value	MIS	MFS
AT5G37640	UBQ9	Polyubiquitin 9	7.47E-05	<0.001	946	0.999
AT3G09790	UBQ8	Polyubiquitin 8	7.49E-05	<0.001	946	0.999
AT4G05050	UBQ11	Polyubiquitin 11	8.00E-05	<0.001	946	0.999
AT4G05320	UBQ10	Polyubiquitin 10	8.37E-05	<0.001	940	0.999
AT1G36370	SHM7	Serine hydroxymethyltransferase 7	2.82E-05	0.001	994	0.997
AT4G13890	SHM5	Serine hydroxymethyltransferase 5	3.11E-05	0.001	995	0.997
AT1G22020	SHM6	Serine hydroxymethyltransferase 6	3.10E-05	<0.001	994	0.997
AT4G37840	HXKL2_ARATH	Probable hexokinase-like 2 protein	3.08E-05	0.007	966	0.997
AT3G60100	CSY5	Citrate synthase 5	2.94E-05	<0.001	980	0.997
AT1G79530	GAPCP1	Glyceraldehyde-3-phosphate dehydrogenase	2.98E-05	0.001	998	0.997

Among the putative genes, various ubiquitin associated genes appear to contribute to fruit-associated biological processes. **UBQ9**, which is also known as AT5G37640 in *Arabidopsis thaliana*, has been mainly reported to contribute to the response to certain cytokines and the ubiquitin-dependent protein catabolic processes [58, 59]. Ubiquitin exists either attached to another protein or unanchored. Depending on the specific Lys site ubiquitin linked, anchored proteins have many specific functions, including lysosomal degradation, endocytosis and DNA damage response [60–62]. Ubiquitin-dependent proteins have been widely reported to contribute to the development and maturation of fruits in various plants, such as tomatoes, bananas and our model plant, *Arabidopsis thaliana* [63–65]. Recent publications confirmed that UBQ9 interacts with various core components of ubiquitin-dependent protein catabolic processes [58]. Therefore, the putative gene UBQ9 may contribute to the specific fruit-associated biological processes. Another putative gene, **UBQ8**, is known as AT3G09790 in *Arabidopsis thaliana*. It is the homologue of UBQ9. As mentioned above, ubiquitin-dependent proteins may mediate the degradation of specific target proteins, such as HOS1, MdCOP1, and ETO1, which may further promote the development and maturation of fruit in *Arabidopsis thaliana* [63–66]. Therefore, such genes may, such as its homologue UBQ9, contribute to specific fruit-associated metabolic processes. **UBQ11**, also known as AT4G05050, has also been confirmed as one of the poly-ubiquitin families which contribute to ERAD (endoplasmic reticulum-associated degradation) [67]. Recent publications confirm that, at least in rice, ERAD-associated biological processes may be directly related to the development and maturation of the fruit, implying its specific role in plant fruiting [68]. Considering that recent publications also identified ERAD-associated biological processes as a crucial regulator for reproduction (including fruits) of *Arabidopsis thaliana*, it is reasonable to regard the putative gene UBQ11 as a candidate fruit-associated gene [69]. The putative gene **UBQ10** encodes another specific ubiquitin-associated protein, also known as AT4G05320, involving ubiquitin-dependent protein catabolism, which we have analyzed above and confirmed its association with fruiting [70].

Apart from the ubiquitin genes analyzed above, we also obtained a group of functional enzymes. Three of them encode subtypes of serine hydroxy methyltransferases. **SHM7**, also known as AT1G36370, encodes the specific serine hydroxy methyltransferase 7, which further contributes to catalyzing the interconversion of serine and glycine [71]. Recent publications confirm that the main function of SHM7, pyridoxal phosphate binding, may contribute to the development and maturation of fruit in citrus, implying its potential role in various plant subtypes [72, 73]. Furthermore, a specific mutant screening of *Arabidopsis thaliana*, based on high-throughput HPLC-MS/MS assay has confirmed that, together with another enzyme threonine aldolase, the serine hydroxy methyltransferase may play a specific role during the development and maturation of fruit, affecting seed nutritional quality [74]. **SHM5** (AT4G13890), another crucial serine hydroxy methyltransferase, has also been predicted to be a candidate fruit-related gene in *Arabidopsis thaliana* [74]. Similar to SHM7 analyzed above, SHM5 has also been confirmed to interact with specific endogenous compounds in plants, such as 5-Methyltetrahydrofolate and 5-formyltetrahydrofolate, in one-carbon metabolism. Thus, SHM5 may further participate in the maturation of fruits in *Arabidopsis thaliana*, validating the specific relationship between this gene and fruit development [75–77]. **SHM6** (AT1G22020) also encodes a specific subtype of serine hydroxy methyltransferases. Although no publications confirmed that such genes contribute to the development or maturation of fruits, considering the crucial regulatory role of serine hydroxy methyltransferases in *Arabidopsis thaliana*, and validated interactions in this study, it is reasonable to regard SHM6 as a candidate functional fruit-related gene [78, 79].

Apart from such serine hydroxy methyltransferases, we also identified specific hexokinase-associated genes **HXKL2_ARATH** (probable hexokinase-like 2 protein) and HXL3 (hexokinase like 3), also known as AT4G37840. Recent publications have confirmed the complex functions of such genes in plants. Expression in citrus guard cells regulates specific sugar-sensing functions during fruit development and maturation, at least in citrus [80]. An earlier exploration confirmed that such genes may contribute to AtRGS1-medated sugar signaling in *Arabidopsis thaliana* [81]. Considering that sugar signaling contributes to the nutrient accumulation and freezing tolerance of fruit, and may affect the longevity of seeds, it is reasonable to conclude that as a functional sugar metabolism regulator, AT4G37840 may contribute to fruit- associated biological processes in *Arabidopsis thaliana* [2, 82, 83]. Another gene, **CSY5**, also known as AT3G60100, contribute to fruit-associated biological processes. Recent publications not only revealed the potential functions of citrate synthase during seed germination but also suggests a crucial role of in fruit development, especially at low temperature [84, 85]. Expressed in fruit and seedlings of *Arabidopsis thaliana*, the putative gene CSY5 may contribute to fruit associated biological processes [84]. **GAPCP1** (glyceraldehyde-3-phosphate dehydrogenase), also known as AT1G79530, is a candidate fruit-related gene in *Arabidopsis thaliana*. Involved in the plastid glycolytic pathway, this gene may contribute to the production of glycolytic energy in non-photosynthetic tissues, especially in fruits [86, 87]. Considering that the glycolytic energy in fruits is important in cellular metabolism and seed oil accumulation in the fruit of *Arabidopsis thaliana*, the putative gene GAPCP1 may be a crucial fruit-related gene.

As we have analyzed above, ten putative genes have been validated to participate in fruit associated biological processes of *Arabidopsis thaliana*. Based on recent publications and our analysis, we identified two crucial biological processes: ubiquitin- associated biological processes and serine hydroxy methyltransferase-associated biological processes that may contribute to the development and maturation of fruit in *Arabidopsis thaliana*. The typical enrichment of some putative genes in such biological processes implies the role and underlying mechanisms of them for fruit development and maturation. The remaining putative genes are not discussed here but, may also be related to fruit-associated biological processes of *Arabidopsis thaliana*. We hope that other investigators will test this hypothesis.

## Conclusions

In this study, we utilized powerful computational techniques for identifying novel fruit-related genes in *Arabidopsis thaliana*, yielding 255 inferred genes. These genes may provide new directions to investigate the biological processes associated with fruiting in *Arabidopsis thaliana*. Furthermore, we believe that this method can be further applied to the recognition of various functional genes/proteins of multiple species, such as DNA-binding protein prediction [88], promoting the development of large-scale gene/protein function prediction and identification.

## Supporting information

S1 Table 994 validated fruit-related genes in *Arabidopsis thaliana* and their fruit-related PO terms. (DOCX)

S2 Table The 6,310 RWR genes with probabilities larger than 10^−5^. (DOCX)

S3 Table The 255 putative fruit-related genes. (DOCX)
