# Supplementary material for: Identifying novel fruit-related genes in Arabidopsis thaliana based on the random walk with restart algorithm
Source: PLoS One. 2017 May 4;12(5):e0177017. doi: 10.1371/journal.pone.0177017 (PMC5417634; doi:10.1371/journal.pone.0177017)
Supplement: S1 Table — (DOCX) [file pone.0177017.s001.docx]

**S1 Table.** 994 validated fruit-related genes in *Arabidopsis thaliana* and their fruit-related PO terms

| **NO.** | **ID** | **PO term** |
| --- | --- | --- |
| 1 | AT4G19440 | PO:0009001 |
| 2 | AT4G32470 | PO:0009001 |
| 3 | AT3G58780 | PO:0004707 |
| 4 | AT2G28000 | PO:0009001 |
| 5 | AT1G75500 | PO:0009001 |
| 6 | AT1G04690 | PO:0009001 |
| 7 | AT5G36700 | PO:0009001 |
| 8 | AT2G21140 | PO:0009001 |
| 9 | AT2G40370 | PO:0009001 |
| 10 | AT3G22690 | PO:0009001 |
| 11 | AT3G19170 | PO:0009001 |
| 12 | AT1G24020 | PO:0009001 |
| 13 | AT4G37610 | PO:0009001 |
| 14 | AT3G01480 | PO:0009001 |
| 15 | AT1G77410 | PO:0009001 |
| 16 | AT5G47760 | PO:0009001 |
| 17 | AT1G11860 | PO:0009001 |
| 18 | AT1G50460 | PO:0009001 |
| 19 | AT1G66730 | PO:0009001 |
| 20 | AT1G05190 | PO:0009001 |
| 21 | AT2G27680 | PO:0009001 |
| 22 | AT3G61415 | PO:0009001 |
| 23 | AT5G61780 | PO:0009001 |
| 24 | AT2G32370 | PO:0009001 |
| 25 | AT4G21650 | PO:0009001 |
| 26 | AT1G78220 | PO:0009001 |
| 27 | AT2G40100 | PO:0009001 |
| 28 | AT5G67360 | PO:0009001 |
| 29 | AT4G26070 | PO:0009001 |
| 30 | AT4G21120 | PO:0009001 |
| 31 | AT5G64560 | PO:0009001 |
| 32 | AT2G45950 | PO:0009001 |
| 33 | AT5G42190 | PO:0009001, PO:0000033 |
| 34 | AT1G66340 | PO:0009001 |
| 35 | AT1G21680 | PO:0009001 |
| 36 | AT5G09590 | PO:0009001 |
| 37 | AT1G15390 | PO:0009001 |
| 38 | AT1G30530 | PO:0009001 |
| 39 | AT1G10590 | PO:0009001 |
| 40 | AT4G00360 | PO:0009001 |
| 41 | AT3G01500 | PO:0009001 |
| 42 | AT2G30210 | PO:0009001 |
| 43 | AT1G13930 | PO:0009001 |
| 44 | AT1G73680 | PO:0009001 |
| 45 | AT3G02310 | PO:0009001 |
| 46 | AT2G18550 | PO:0009001 |
| 47 | AT2G45240 | PO:0009001 |
| 48 | AT1G47840 | PO:0009001 |
| 49 | AT2G01520 | PO:0009001 |
| 50 | AT4G33680 | PO:0009001 |
| 51 | AT4G18480 | PO:0009001 |
| 52 | AT3G50820 | PO:0009001 |
| 53 | AT2G36640 | PO:0009001 |
| 54 | AT5G57390 | PO:0009001 |
| 55 | AT4G37680 | PO:0009001 |
| 56 | AT1G29670 | PO:0009001 |
| 57 | AT4G21630 | PO:0009001 |
| 58 | AT5G09410 | PO:0009001 |
| 59 | AT4G38320 | PO:0009001 |
| 60 | AT3G56950 | PO:0009001 |
| 61 | AT3G23980 | PO:0009001 |
| 62 | AT5G56300 | PO:0009001 |
| 63 | AT2G15230 | PO:0009001 |
| 64 | AT5G37830 | PO:0009001 |
| 65 | AT3G52150 | PO:0009001 |
| 66 | AT3G09820 | PO:0009001 |
| 67 | AT5G38940 | PO:0009001 |
| 68 | AT3G04280 | PO:0009001 |
| 69 | AT1G53850 | PO:0009001 |
| 70 | AT5G08100 | PO:0009001 |
| 71 | AT3G54610 | PO:0009001 |
| 72 | AT1G50900 | PO:0009001 |
| 73 | AT2G38080 | PO:0009001, PO:0000033 |
| 74 | AT4G32810 | PO:0009001 |
| 75 | AT2G38170 | PO:0009001 |
| 76 | AT3G04680 | PO:0009001 |
| 77 | AT2G05840 | PO:0009001 |
| 78 | AT3G14790 | PO:0009001 |
| 79 | AT4G35830 | PO:0009001 |
| 80 | AT3G10870 | PO:0009001 |
| 81 | AT4G37930 | PO:0009001 |
| 82 | AT3G43810 | PO:0009001 |
| 83 | AT3G02080 | PO:0009001 |
| 84 | AT1G14890 | PO:0009001 |
| 85 | AT2G38470 | PO:0009001 |
| 86 | AT2G37660 | PO:0009001 |
| 87 | AT3G48350 | PO:0009001 |
| 88 | AT3G26650 | PO:0009001 |
| 89 | AT1G15550 | PO:0009001 |
| 90 | AT5G15150 | PO:0009001 |
| 91 | AT2G47510 | PO:0009001 |
| 92 | AT5G25980 | PO:0009001 |
| 93 | AT4G23670 | PO:0009001 |
| 94 | AT1G31630 | PO:0009001 |
| 95 | AT2G30860 | PO:0009001 |
| 96 | AT5G64440 | PO:0009001 |
| 97 | AT5G58230 | PO:0009001 |
| 98 | AT5G36210 | PO:0009001 |
| 99 | AT3G51920 | PO:0009001 |
| 100 | AT2G41100 | PO:0009001 |
| 101 | AT1G56190 | PO:0009001 |
| 102 | AT4G03210 | PO:0009001 |
| 103 | AT5G35630 | PO:0009001 |
| 104 | AT2G25700 | PO:0000033 |
| 105 | AT2G27040 | PO:0009001 |
| 106 | AT5G09690 | PO:0009001 |
| 107 | AT5G11950 | PO:0009001 |
| 108 | AT2G41110 | PO:0009001 |
| 109 | AT5G56640 | PO:0009001 |
| 110 | AT5G10170 | PO:0009001 |
| 111 | AT5G63840 | PO:0009001 |
| 112 | AT5G15450 | PO:0009001 |
| 113 | AT3G20310 | PO:0009001 |
| 114 | AT5G56290 | PO:0009001 |
| 115 | AT4G39650 | PO:0009001 |
| 116 | AT1G78900 | PO:0009001 |
| 117 | AT5G48880 | PO:0009001 |
| 118 | AT1G51680 | PO:0009001 |
| 119 | AT4G15490 | PO:0009001 |
| 120 | AT3G57650 | PO:0009001 |
| 121 | AT3G04580 | PO:0009001 |
| 122 | AT5G53520 | PO:0009001 |
| 123 | AT4G15560 | PO:0009001 |
| 124 | AT3G27000 | PO:0009001 |
| 125 | AT4G28750 | PO:0009001 |
| 126 | AT5G63860 | PO:0009001 |
| 127 | AT5G01500 | PO:0009001 |
| 128 | AT5G27320 | PO:0009001 |
| 129 | AT1G20330 | PO:0009001 |
| 130 | AT2G44750 | PO:0009001 |
| 131 | AT5G03300 | PO:0009001 |
| 132 | AT5G48300 | PO:0009001 |
| 133 | AT5G38110 | PO:0009001 |
| 134 | AT1G08830 | PO:0009001 |
| 135 | AT1G79930 | PO:0009001 |
| 136 | AT3G52420 | PO:0009001 |
| 137 | AT1G31930 | PO:0009001 |
| 138 | AT5G65310 | PO:0009001 |
| 139 | AT1G27050 | PO:0009001 |
| 140 | AT1G16080 | PO:0009001 |
| 141 | AT1G62960 | PO:0009001 |
| 142 | AT5G60760 | PO:0009001 |
| 143 | AT4G16160 | PO:0009001 |
| 144 | AT5G35770 | PO:0009001 |
| 145 | AT2G16600 | PO:0009001 |
| 146 | AT3G01150 | PO:0009001 |
| 147 | AT5G63800 | PO:0009001 |
| 148 | AT5G58330 | PO:0009001 |
| 149 | AT3G07130 | PO:0009001 |
| 150 | AT2G21660 | PO:0009001 |
| 151 | AT1G15080 | PO:0009001 |
| 152 | AT5G62810 | PO:0009001 |
| 153 | AT4G00150 | PO:0009001 |
| 154 | AT3G58970 | PO:0009001 |
| 155 | AT1G04580 | PO:0009001 |
| 156 | AT5G18290 | PO:0009001 |
| 157 | AT1G22270 | PO:0009001 |
| 158 | AT4G25000 | PO:0009001 |
| 159 | AT2G03450 | PO:0009001 |
| 160 | AT4G15500 | PO:0009001 |
| 161 | AT3G20390 | PO:0009001 |
| 162 | AT3G21850 | PO:0009001, PO:0000033 |
| 163 | AT5G37180 | PO:0009001 |
| 164 | AT5G06300 | PO:0009001 |
| 165 | AT1G14760 | PO:0009001 |
| 166 | AT1G26830 | PO:0009001 |
| 167 | AT5G54500 | PO:0009001 |
| 168 | AT5G48480 | PO:0009001 |
| 169 | AT1G03130 | PO:0009001 |
| 170 | AT4G28485 | PO:0009001 |
| 171 | AT2G40490 | PO:0009001 |
| 172 | AT3G24170 | PO:0009001 |
| 173 | AT5G51810 | PO:0009001 |
| 174 | AT2G01290 | PO:0009001 |
| 175 | AT5G42180 | PO:0009001 |
| 176 | AT3G21840 | PO:0009001 |
| 177 | AT3G16785 | PO:0009001 |
| 178 | AT3G19700 | PO:0009001 |
| 179 | AT3G06580 | PO:0009001 |
| 180 | AT3G52780 | PO:0009001 |
| 181 | AT1G22450 | PO:0009001 |
| 182 | AT2G45160 | PO:0009001 |
| 183 | AT2G46020 | PO:0009001 |
| 184 | AT4G39210 | PO:0009001 |
| 185 | AT3G52190 | PO:0009001 |
| 186 | AT3G23990 | PO:0009001 |
| 187 | AT2G33150 | PO:0009001 |
| 188 | AT3G22650 | PO:0009001 |
| 189 | AT1G13440 | PO:0009001 |
| 190 | AT3G63150 | PO:0009001 |
| 191 | AT3G51820 | PO:0009001 |
| 192 | AT5G04530 | PO:0009001 |
| 193 | AT1G68560 | PO:0009001 |
| 194 | AT1G10230 | PO:0009001 |
| 195 | AT3G15356 | PO:0009001 |
| 196 | AT2G22430 | PO:0009001 |
| 197 | AT5G60970 | PO:0009001 |
| 198 | AT3G57610 | PO:0009001 |
| 199 | AT1G54360 | PO:0009001 |
| 200 | AT2G06050 | PO:0009001 |
| 201 | AT5G46090 | PO:0009001 |
| 202 | AT1G29920 | PO:0009001 |
| 203 | AT5G04885 | PO:0009001 |
| 204 | AT1G17060 | PO:0009001 |
| 205 | AT5G17920 | PO:0009001 |
| 206 | AT5G53180 | PO:0009001 |
| 207 | AT3G01220 | PO:0009001 |
| 208 | AT1G72320 | PO:0009001 |
| 209 | AT4G03050 | PO:0009001 |
| 210 | AT1G21910 | PO:0009001 |
| 211 | AT4G12560 | PO:0009001 |
| 212 | AT1G30210 | PO:0009001 |
| 213 | AT1G30000 | PO:0009001 |
| 214 | AT1G35730 | PO:0004707 |
| 215 | AT1G20140 | PO:0000033 |
| 216 | AT5G53560 | PO:0009001 |
| 217 | AT5G20950 | PO:0009001 |
| 218 | AT3G28300 | PO:0009001 |
| 219 | AT2G30950 | PO:0009001 |
| 220 | AT2G39730 | PO:0009001 |
| 221 | AT1G36160 | PO:0009001 |
| 222 | AT2G01850 | PO:0009001 |
| 223 | AT5G18380 | PO:0009001 |
| 224 | AT3G28917 | PO:0009001 |
| 225 | AT3G05630 | PO:0009001 |
| 226 | AT1G53070 | PO:0009001 |
| 227 | AT3G44735 | PO:0009001 |
| 228 | AT2G28760 | PO:0009001 |
| 229 | AT3G16470 | PO:0009001 |
| 230 | AT1G14870 | PO:0009001 |
| 231 | AT5G10330 | PO:0009001 |
| 232 | AT5G01040 | PO:0009001 |
| 233 | AT1G05590 | PO:0009001 |
| 234 | AT4G13930 | PO:0009001 |
| 235 | AT1G16010 | PO:0009001 |
| 236 | AT1G53000 | PO:0009001 |
| 237 | AT3G02230 | PO:0009001 |
| 238 | AT1G03900 | PO:0009001 |
| 239 | AT5G01190 | PO:0009001 |
| 240 | AT3G13750 | PO:0009001 |
| 241 | AT1G75020 | PO:0009001 |
| 242 | AT1G63940 | PO:0009001 |
| 243 | AT1G52400 | PO:0009001 |
| 244 | AT2G21170 | PO:0009001 |
| 245 | AT1G08110 | PO:0009001 |
| 246 | AT1G56450 | PO:0009001 |
| 247 | AT2G35960 | PO:0009001 |
| 248 | AT4G33000 | PO:0009001 |
| 249 | AT3G60630 | PO:0009001 |
| 250 | AT3G52720 | PO:0009001 |
| 251 | AT1G73220 | PO:0009001 |
| 252 | AT2G47730 | PO:0009001 |
| 253 | AT5G11450 | PO:0009001 |
| 254 | AT4G33410 | PO:0009001 |
| 255 | AT1G19440 | PO:0009001 |
| 256 | AT1G16470 | PO:0009001 |
| 257 | AT1G77210 | PO:0009001 |
| 258 | AT5G23720 | PO:0009001 |
| 259 | AT3G52930 | PO:0009001 |
| 260 | AT5G14740 | PO:0009001 |
| 261 | AT3G59970 | PO:0009001 |
| 262 | AT2G35980 | PO:0009001 |
| 263 | AT2G46370 | PO:0009001 |
| 264 | AT1G21750 | PO:0009001 |
| 265 | AT2G26710 | PO:0009001 |
| 266 | AT4G13850 | PO:0009001 |
| 267 | AT1G18080 | PO:0009001 |
| 268 | AT2G38110 | PO:0009001 |
| 269 | AT2G28305 | PO:0009001 |
| 270 | AT1G66400 | PO:0009001 |
| 271 | AT3G55200 | PO:0009001 |
| 272 | AT3G43190 | PO:0009001 |
| 273 | AT2G26080 | PO:0009001 |
| 274 | AT5G50380 | PO:0009001 |
| 275 | AT1G75090 | PO:0009001 |
| 276 | AT1G52760 | PO:0009001 |
| 277 | AT4G34060 | PO:0009001 |
| 278 | AT5G13930 | PO:0009001 |
| 279 | AT5G01370 | PO:0009087 |
| 280 | AT3G58700 | PO:0009001 |
| 281 | AT1G22985 | PO:0009001 |
| 282 | AT4G38770 | PO:0009001 |
| 283 | AT4G09650 | PO:0009001 |
| 284 | AT4G27490 | PO:0009001 |
| 285 | AT1G32150 | PO:0009001 |
| 286 | AT5G22640 | PO:0009001 |
| 287 | AT3G25100 | PO:0009001 |
| 288 | AT3G14415 | PO:0009001 |
| 289 | AT4G30830 | PO:0004707 |
| 290 | AT2G05100 | PO:0009001 |
| 291 | AT5G05390 | PO:0009001, PO:0000033 |
| 292 | AT2G47050 | PO:0009001 |
| 293 | AT3G12890 | PO:0009001 |
| 294 | AT3G31350 | PO:0009001 |
| 295 | AT1G70730 | PO:0009001 |
| 296 | AT4G27630 | PO:0009001 |
| 297 | AT4G29940 | PO:0009001 |
| 298 | AT3G61440 | PO:0009001 |
| 299 | AT1G17920 | PO:0009001 |
| 300 | AT4G24280 | PO:0009001 |
| 301 | AT5G42740 | PO:0009001 |
| 302 | AT2G25680 | PO:0009001 |
| 303 | AT1G04220 | PO:0009001 |
| 304 | AT5G28500 | PO:0009001 |
| 305 | AT5G04770 | PO:0009001 |
| 306 | AT1G74380 | PO:0009001 |
| 307 | AT3G19270 | PO:0009084 |
| 308 | AT1G67140 | PO:0009001 |
| 309 | AT1G03790 | PO:0009001 |
| 310 | AT2G29090 | PO:0009001 |
| 311 | AT2G22475 | PO:0009001 |
| 312 | AT1G14700 | PO:0009001 |
| 313 | AT3G05420 | PO:0009001 |
| 314 | AT3G26450 | PO:0009001 |
| 315 | AT3G15020 | PO:0009001 |
| 316 | AT5G16020 | PO:0009001 |
| 317 | AT2G29525 | PO:0009001 |
| 318 | AT5G67500 | PO:0009001 |
| 319 | AT2G15090 | PO:0009001 |
| 320 | AT5G02790 | PO:0009001 |
| 321 | AT1G59900 | PO:0009001 |
| 322 | AT1G59610 | PO:0009001 |
| 323 | AT3G08590 | PO:0009001 |
| 324 | AT2G45790 | PO:0009001 |
| 325 | AT5G27630 | PO:0009001 |
| 326 | AT2G16280 | PO:0009001 |
| 327 | AT4G29010 | PO:0009001 |
| 328 | AT1G10170 | PO:0009001 |
| 329 | AT2G39330 | PO:0009001 |
| 330 | AT2G37220 | PO:0009001 |
| 331 | AT3G63010 | PO:0009001 |
| 332 | AT2G21540 | PO:0009001 |
| 333 | AT2G03170 | PO:0009001 |
| 334 | AT3G55990 | PO:0009001 |
| 335 | AT5G43760 | PO:0009001 |
| 336 | AT1G02880 | PO:0009001 |
| 337 | AT3G52890 | PO:0009001 |
| 338 | AT2G46070 | PO:0009001 |
| 339 | AT5G02640 | PO:0009001, PO:0004707 |
| 340 | AT1G13020 | PO:0009001 |
| 341 | AT4G34050 | PO:0009001 |
| 342 | AT1G01120 | PO:0009001 |
| 343 | AT4G26200 | PO:0009001 |
| 344 | AT3G04090 | PO:0009001 |
| 345 | AT1G13180 | PO:0009001 |
| 346 | AT5G38420 | PO:0009001 |
| 347 | AT1G06520 | PO:0009001 |
| 348 | AT2G03520 | PO:0009001 |
| 349 | AT1G02930 | PO:0009001 |
| 350 | AT3G10150 | PO:0009001 |
| 351 | AT1G48030 | PO:0009001 |
| 352 | AT2G32440 | PO:0009001 |
| 353 | AT5G38430 | PO:0009001 |
| 354 | AT3G06930 | PO:0009001 |
| 355 | AT2G05990 | PO:0009001 |
| 356 | AT1G19920 | PO:0009001 |
| 357 | AT3G57240 | PO:0009001 |
| 358 | AT3G63140 | PO:0009001 |
| 359 | AT3G47520 | PO:0009001 |
| 360 | AT1G80600 | PO:0009001 |
| 361 | AT1G25450 | PO:0009001 |
| 362 | AT2G36610 | PO:0009001 |
| 363 | AT3G50660 | PO:0009001 |
| 364 | AT5G53490 | PO:0009001 |
| 365 | AT1G71160 | PO:0009001 |
| 366 | AT3G17600 | PO:0009001 |
| 367 | AT5G66510 | PO:0009001 |
| 368 | AT3G01280 | PO:0009001 |
| 369 | AT5G26650 | PO:0009001 |
| 370 | AT4G31300 | PO:0009001 |
| 371 | AT5G07350 | PO:0009001 |
| 372 | AT4G22240 | PO:0009001 |
| 373 | AT3G17390 | PO:0009001 |
| 374 | AT3G18830 | PO:0009001 |
| 375 | AT2G29630 | PO:0009001 |
| 376 | AT1G53240 | PO:0009001 |
| 377 | AT2G45310 | PO:0009001 |
| 378 | AT4G14880 | PO:0009001 |
| 379 | AT5G61960 | PO:0009001 |
| 380 | AT5G01840 | PO:0009001 |
| 381 | AT5G09660 | PO:0009001 |
| 382 | AT3G24280 | PO:0009001 |
| 383 | AT4G34510 | PO:0009001 |
| 384 | AT3G61150 | PO:0009001 |
| 385 | AT1G52030 | PO:0009001 |
| 386 | AT5G24770 | PO:0009001 |
| 387 | AT5G15090 | PO:0009001 |
| 388 | AT5G25350 | PO:0009001 |
| 389 | AT2G44180 | PO:0009001 |
| 390 | AT4G34870 | PO:0009001 |
| 391 | AT5G42980 | PO:0009001 |
| 392 | AT2G03160 | PO:0000033 |
| 393 | AT5G63310 | PO:0009001 |
| 394 | AT2G05710 | PO:0009001 |
| 395 | AT3G20470 | PO:0009001 |
| 396 | AT1G28520 | PO:0009001 |
| 397 | AT4G38970 | PO:0009001 |
| 398 | AT1G27460 | PO:0009001 |
| 399 | AT2G46720 | PO:0009001 |
| 400 | AT4G28520 | PO:0009001 |
| 401 | AT5G48810 | PO:0009001 |
| 402 | AT5G48580 | PO:0009001 |
| 403 | AT5G03790 | PO:0009001 |
| 404 | AT2G36305 | PO:0009001 |
| 405 | AT2G30250 | PO:0009001 |
| 406 | AT1G27080 | PO:0009001, PO:0008003 |
| 407 | AT3G08940 | PO:0009001 |
| 408 | AT5G35590 | PO:0009001 |
| 409 | AT5G40370 | PO:0009001 |
| 410 | AT5G66570 | PO:0009001 |
| 411 | AT2G41840 | PO:0009001 |
| 412 | AT1G42970 | PO:0009001 |
| 413 | AT1G31740 | PO:0009001 |
| 414 | AT3G28290 | PO:0009001 |
| 415 | AT3G05120 | PO:0009001 |
| 416 | AT4G28510 | PO:0009001 |
| 417 | AT5G24300 | PO:0009001 |
| 418 | AT1G32060 | PO:0009001 |
| 419 | AT5G63140 | PO:0009001 |
| 420 | AT1G44800 | PO:0009001 |
| 421 | AT1G55250 | PO:0009001 |
| 422 | AT1G16360 | PO:0009001 |
| 423 | AT5G15540 | PO:0009001 |
| 424 | AT5G06250 | PO:0009001 |
| 425 | AT5G57655 | PO:0009001 |
| 426 | AT3G58680 | PO:0009001 |
| 427 | AT4G34580 | PO:0009001 |
| 428 | AT1G04310 | PO:0009001 |
| 429 | AT3G57670 | PO:0025268 |
| 430 | AT2G38230 | PO:0009001 |
| 431 | AT2G06850 | PO:0009001 |
| 432 | AT3G01470 | PO:0009001 |
| 433 | AT1G68530 | PO:0009001 |
| 434 | AT2G37190 | PO:0009001 |
| 435 | AT4G34390 | PO:0009001 |
| 436 | AT2G30870 | PO:0009001 |
| 437 | AT2G32770 | PO:0009001 |
| 438 | AT1G76430 | PO:0009001 |
| 439 | AT1G76450 | PO:0009001 |
| 440 | AT3G56700 | PO:0009001, PO:0004536 |
| 441 | AT2G35990 | PO:0009001 |
| 442 | AT1G11720 | PO:0009001 |
| 443 | AT5G13510 | PO:0009001 |
| 444 | AT1G05320 | PO:0009001 |
| 445 | AT3G13470 | PO:0009001 |
| 446 | AT3G09840 | PO:0009001 |
| 447 | AT4G33010 | PO:0009001 |
| 448 | AT1G73250 | PO:0009001 |
| 449 | AT3G58740 | PO:0009001 |
| 450 | AT1G11840 | PO:0009001 |
| 451 | AT3G52300 | PO:0009001 |
| 452 | AT3G62710 | PO:0009001 |
| 453 | AT1G23190 | PO:0009001 |
| 454 | AT1G02205 | PO:0009001 |
| 455 | AT5G26310 | PO:0009001 |
| 456 | AT4G21690 | PO:0009001 |
| 457 | AT1G16280 | PO:0009001 |
| 458 | AT1G47710 | PO:0009001 |
| 459 | AT1G69500 | PO:0004536 |
| 460 | AT5G11670 | PO:0009001 |
| 461 | AT5G19220 | PO:0009001 |
| 462 | AT5G61980 | PO:0009001 |
| 463 | AT5G64570 | PO:0009001 |
| 464 | AT5G49020 | PO:0009001 |
| 465 | AT5G02840 | PO:0009001 |
| 466 | AT5G01180 | PO:0009001 |
| 467 | AT1G77200 | PO:0004707, PO:0000033 |
| 468 | AT1G58120 | PO:0025268, PO:0008003 |
| 469 | AT3G51860 | PO:0009001 |
| 470 | AT1G80900 | PO:0009001 |
| 471 | AT5G57140 | PO:0009001 |
| 472 | AT1G32470 | PO:0009001 |
| 473 | AT2G40250 | PO:0009001 |
| 474 | AT5G07030 | PO:0009001 |
| 475 | AT3G51160 | PO:0009001 |
| 476 | AT2G31955 | PO:0009001 |
| 477 | AT3G06920 | PO:0009001 |
| 478 | AT3G20500 | PO:0009001 |
| 479 | AT4G25470 | PO:0009001 |
| 480 | AT1G31180 | PO:0009001 |
| 481 | AT2G22760 | PO:0004707, PO:0000033 |
| 482 | AT3G17940 | PO:0009001 |
| 483 | AT3G53970 | PO:0009001 |
| 484 | AT1G63690 | PO:0009001 |
| 485 | AT2G20825 | PO:0009001 |
| 486 | AT1G75110 | PO:0009001 |
| 487 | AT4G38740 | PO:0009001 |
| 488 | AT4G28190 | PO:0009001 |
| 489 | AT5G11190 | PO:0004707 |
| 490 | AT2G01170 | PO:0009001 |
| 491 | AT3G60750 | PO:0009001 |
| 492 | AT4G20140 | PO:0009001 |
| 493 | AT3G02150 | PO:0009001 |
| 494 | AT4G34980 | PO:0009001 |
| 495 | AT4G26420 | PO:0009001 |
| 496 | AT3G55510 | PO:0009001 |
| 497 | AT1G73360 | PO:0009001 |
| 498 | AT1G02340 | PO:0009001 |
| 499 | AT4G04640 | PO:0009001 |
| 500 | AT3G48340 | PO:0009001 |
| 501 | AT5G57800 | PO:0009001 |
| 502 | AT1G35720 | PO:0009001 |
| 503 | AT5G14130 | PO:0009001 |
| 504 | AT1G57750 | PO:0009001 |
| 505 | AT4G27780 | PO:0009001 |
| 506 | AT3G48420 | PO:0009001 |
| 507 | AT2G23510 | PO:0009001 |
| 508 | AT3G07650 | PO:0009001 |
| 509 | AT2G20420 | PO:0009001 |
| 510 | AT5G23120 | PO:0009001 |
| 511 | AT1G08200 | PO:0009001 |
| 512 | AT5G26000 | PO:0009001 |
| 513 | AT2G39460 | PO:0009001 |
| 514 | AT1G58180 | PO:0009001 |
| 515 | AT5G53480 | PO:0009001 |
| 516 | AT2G19110 | PO:0009001 |
| 517 | AT1G60860 | PO:0009001 |
| 518 | AT5G07370 | PO:0009001 |
| 519 | AT4G03280 | PO:0009001 |
| 520 | AT5G27450 | PO:0009001 |
| 521 | AT3G54660 | PO:0009001 |
| 522 | AT1G70070 | PO:0009001 |
| 523 | AT4G38510 | PO:0009001 |
| 524 | AT3G55440 | PO:0009001 |
| 525 | AT5G16710 | PO:0009001 |
| 526 | AT5G06950 | PO:0009001 |
| 527 | AT2G31390 | PO:0009001 |
| 528 | AT1G25340 | PO:0009001, PO:0004707 |
| 529 | AT5G26594 | PO:0009001 |
| 530 | AT1G75280 | PO:0009001 |
| 531 | AT2G32810 | PO:0009001 |
| 532 | AT4G19230 | PO:0009084 |
| 533 | AT3G03260 | PO:0009001 |
| 534 | AT3G02000 | PO:0009001 |
| 535 | AT3G14420 | PO:0009001 |
| 536 | AT1G67590 | PO:0009001 |
| 537 | AT2G42400 | PO:0009001 |
| 538 | AT1G06680 | PO:0009001 |
| 539 | AT2G28630 | PO:0009001 |
| 540 | AT4G34710 | PO:0009001 |
| 541 | AT3G52810 | PO:0009001 |
| 542 | AT5G39570 | PO:0009001 |
| 543 | AT1G05610 | PO:0009001 |
| 544 | AT5G55480 | PO:0009001 |
| 545 | AT2G45970 | PO:0009001 |
| 546 | AT4G31990 | PO:0009001 |
| 547 | AT1G55600 | PO:0009001 |
| 548 | AT1G50500 | PO:0009001 |
| 549 | AT4G01940 | PO:0009001 |
| 550 | AT1G48850 | PO:0009001 |
| 551 | AT4G26840 | PO:0009001 |
| 552 | AT1G65650 | PO:0009001 |
| 553 | AT5G10730 | PO:0009001 |
| 554 | AT2G33380 | PO:0009001 |
| 555 | AT3G44880 | PO:0009001 |
| 556 | AT4G15570 | PO:0009001 |
| 557 | AT4G14800 | PO:0009001 |
| 558 | AT5G07200 | PO:0009001 |
| 559 | AT2G26640 | PO:0009001 |
| 560 | AT4G18240 | PO:0009001 |
| 561 | AT4G39330 | PO:0009001 |
| 562 | AT2G21330 | PO:0009001 |
| 563 | AT2G03680 | PO:0009001 |
| 564 | AT1G80150 | PO:0009001 |
| 565 | AT1G51980 | PO:0009001 |
| 566 | AT1G76680 | PO:0009001 |
| 567 | AT2G23460 | PO:0009001 |
| 568 | AT5G34850 | PO:0009001 |
| 569 | AT4G24310 | PO:0009001 |
| 570 | AT1G71696 | PO:0009001 |
| 571 | AT5G65800 | PO:0009001 |
| 572 | AT4G01310 | PO:0009001 |
| 573 | AT3G06550 | PO:0009001 |
| 574 | AT1G56340 | PO:0009001 |
| 575 | AT5G38970 | PO:0009001 |
| 576 | AT4G36740 | PO:0009001 |
| 577 | AT3G27670 | PO:0009001 |
| 578 | AT1G67090 | PO:0009001 |
| 579 | AT2G01880 | PO:0009001 |
| 580 | AT5G20270 | PO:0009001 |
| 581 | AT3G61890 | PO:0009001 |
| 582 | AT3G04120 | PO:0009001 |
| 583 | AT5G62790 | PO:0009001 |
| 584 | AT2G31081 | PO:0009001 |
| 585 | AT5G65050 | PO:0009001 |
| 586 | AT5G03630 | PO:0009001 |
| 587 | AT1G10010 | PO:0009001 |
| 588 | AT2G28470 | PO:0009001 |
| 589 | AT1G61720 | PO:0009001 |
| 590 | AT2G19860 | PO:0009001 |
| 591 | AT5G20720 | PO:0009001 |
| 592 | AT4G00416 | PO:0009001 |
| 593 | AT1G18835 | PO:0009001 |
| 594 | AT5G55310 | PO:0009001 |
| 595 | AT1G17260 | PO:0009001 |
| 596 | AT4G33580 | PO:0009001 |
| 597 | AT2G35100 | PO:0009001 |
| 598 | AT5G46880 | PO:0009001 |
| 599 | AT1G78590 | PO:0009001 |
| 600 | AT2G46830 | PO:0009001 |
| 601 | AT3G62100 | PO:0009001 |
| 602 | AT3G27785 | PO:0009001 |
| 603 | AT1G75950 | PO:0009001, PO:0000033 |
| 604 | AT1G54990 | PO:0009001 |
| 605 | AT4G21040 | PO:0009001 |
| 606 | AT4G39660 | PO:0009001 |
| 607 | AT4G03430 | PO:0009001 |
| 608 | AT1G77940 | PO:0009001 |
| 609 | AT3G27690 | PO:0009001 |
| 610 | AT2G29130 | PO:0009001, PO:0000033 |
| 611 | AT3G20520 | PO:0009001 |
| 612 | AT5G08335 | PO:0009001 |
| 613 | AT4G12250 | PO:0009001 |
| 614 | AT1G78380 | PO:0009001 |
| 615 | AT1G21640 | PO:0009001 |
| 616 | AT1G20020 | PO:0009001 |
| 617 | AT2G24270 | PO:0009001 |
| 618 | AT5G01600 | PO:0009001 |
| 619 | AT1G04420 | PO:0009001 |
| 620 | AT4G24940 | PO:0009001 |
| 621 | AT2G01890 | PO:0009001 |
| 622 | AT5G23260 | PO:0009001 |
| 623 | AT3G12290 | PO:0009001 |
| 624 | AT2G36880 | PO:0009001 |
| 625 | AT4G39260 | PO:0009001 |
| 626 | AT1G17100 | PO:0009001 |
| 627 | AT3G55260 | PO:0009001 |
| 628 | AT5G06320 | PO:0009001 |
| 629 | AT3G17790 | PO:0009001 |
| 630 | AT4G29350 | PO:0009001 |
| 631 | AT5G03260 | PO:0009001 |
| 632 | AT4G09320 | PO:0009001 |
| 633 | AT1G30270 | PO:0009001 |
| 634 | AT5G60540 | PO:0009001 |
| 635 | AT4G19180 | PO:0000033 |
| 636 | AT5G49910 | PO:0009001 |
| 637 | AT3G51550 | PO:0009001 |
| 638 | AT3G25740 | PO:0009001 |
| 639 | AT2G42600 | PO:0009001 |
| 640 | AT4G18390 | PO:0009001 |
| 641 | AT4G21280 | PO:0009001 |
| 642 | AT4G12420 | PO:0009001 |
| 643 | AT3G19640 | PO:0009001 |
| 644 | AT3G59820 | PO:0009001 |
| 645 | AT2G22240 | PO:0009001 |
| 646 | AT1G68010 | PO:0009001 |
| 647 | AT4G24120 | PO:0009001 |
| 648 | AT4G14640 | PO:0009001 |
| 649 | AT2G18915 | PO:0009001 |
| 650 | AT2G35700 | PO:0009001 |
| 651 | AT2G17780 | PO:0009001 |
| 652 | AT2G37270 | PO:0009001 |
| 653 | AT3G18850 | PO:0009001 |
| 654 | AT3G22990 | PO:0009001 |
| 655 | AT2G26670 | PO:0009001 |
| 656 | AT3G45140 | PO:0009001 |
| 657 | AT2G27710 | PO:0009001 |
| 658 | AT3G44110 | PO:0009001 |
| 659 | AT2G23430 | PO:0009001 |
| 660 | AT2G19800 | PO:0009001 |
| 661 | AT1G26210 | PO:0008003 |
| 662 | AT4G13985 | PO:0004536 |
| 663 | AT1G63770 | PO:0009001 |
| 664 | AT1G71100 | PO:0009001 |
| 665 | AT3G59010 | PO:0009001 |
| 666 | AT3G60010 | PO:0009001 |
| 667 | AT1G56550 | PO:0008001, PO:0008002 |
| 668 | AT4G12130 | PO:0009001 |
| 669 | AT1G33120 | PO:0009001 |
| 670 | AT1G79280 | PO:0009001 |
| 671 | AT1G10270 | PO:0009001 |
| 672 | AT3G21520 | PO:0009001 |
| 673 | AT2G44080 | PO:0009001 |
| 674 | AT4G11150 | PO:0009001 |
| 675 | AT1G48605 | PO:0009001 |
| 676 | AT5G38410 | PO:0009001 |
| 677 | AT4G09600 | PO:0009001 |
| 678 | AT5G02240 | PO:0009001 |
| 679 | AT1G53510 | PO:0009001 |
| 680 | AT5G44700 | PO:0009001 |
| 681 | AT4G10040 | PO:0009001 |
| 682 | AT1G52940 | PO:0009001 |
| 683 | AT5G20830 | PO:0009001, PO:0008003 |
| 684 | AT4G22260 | PO:0009001 |
| 685 | AT1G09210 | PO:0009001 |
| 686 | AT5G50920 | PO:0009001 |
| 687 | AT5G56870 | PO:0009001 |
| 688 | AT2G37940 | PO:0009001 |
| 689 | AT5G07440 | PO:0009001 |
| 690 | AT4G28600 | PO:0009001 |
| 691 | AT2G44950 | PO:0009001 |
| 692 | AT5G20620 | PO:0009001 |
| 693 | AT1G07770 | PO:0009001 |
| 694 | AT1G44090 | PO:0009001 |
| 695 | AT1G32070 | PO:0009001 |
| 696 | AT1G47260 | PO:0009001 |
| 697 | AT5G42020 | PO:0009001 |
| 698 | AT3G52880 | PO:0009001 |
| 699 | AT1G33140 | PO:0009001 |
| 700 | AT3G16950 | PO:0009001 |
| 701 | AT5G54690 | PO:0009001 |
| 702 | AT3G61050 | PO:0009001 |
| 703 | AT5G42810 | PO:0009001 |
| 704 | AT1G07890 | PO:0009001 |
| 705 | AT4G29130 | PO:0009001 |
| 706 | AT5G36790 | PO:0009001 |
| 707 | AT4G21050 | PO:0009001 |
| 708 | AT3G54020 | PO:0009001 |
| 709 | AT5G20940 | PO:0009001 |
| 710 | AT3G53460 | PO:0009001 |
| 711 | AT3G14930 | PO:0009001 |
| 712 | AT2G34500 | PO:0009001 |
| 713 | AT5G45340 | PO:0009001, PO:0009084 |
| 714 | AT3G54320 | PO:0009001 |
| 715 | AT5G49570 | PO:0009001 |
| 716 | AT1G03600 | PO:0009001 |
| 717 | AT2G03120 | PO:0009001 |
| 718 | AT4G02280 | PO:0009001 |
| 719 | AT3G19370 | PO:0009001 |
| 720 | AT4G16120 | PO:0009001 |
| 721 | AT1G76690 | PO:0009001 |
| 722 | AT2G39550 | PO:0009001 |
| 723 | AT2G31070 | PO:0009001 |
| 724 | AT1G51590 | PO:0009001 |
| 725 | AT4G13940 | PO:0009001 |
| 726 | AT3G01180 | PO:0009001 |
| 727 | AT2G42620 | PO:0009001 |
| 728 | AT5G14780 | PO:0009001 |
| 729 | AT2G47990 | PO:0009001 |
| 730 | AT2G30330 | PO:0009001 |
| 731 | AT3G63520 | PO:0009001 |
| 732 | AT5G65870 | PO:0009001 |
| 733 | AT5G08070 | PO:0009001 |
| 734 | AT1G76130 | PO:0009001 |
| 735 | AT1G68750 | PO:0009001 |
| 736 | AT3G03250 | PO:0009001 |
| 737 | AT4G18950 | PO:0009001 |
| 738 | AT4G37780 | PO:0004707, PO:0004536 |
| 739 | AT4G17460 | PO:0009001 |
| 740 | AT1G02000 | PO:0009001 |
| 741 | AT5G13450 | PO:0009001 |
| 742 | AT5G16290 | PO:0009001 |
| 743 | AT2G38530 | PO:0009001 |
| 744 | AT1G53500 | PO:0009001 |
| 745 | AT1G19570 | PO:0009001 |
| 746 | AT3G25650 | PO:0000033 |
| 747 | AT1G65930 | PO:0009001 |
| 748 | AT3G55330 | PO:0009001 |
| 749 | AT2G13790 | PO:0009001 |
| 750 | AT4G32520 | PO:0009001 |
| 751 | AT2G26890 | PO:0009001 |
| 752 | AT4G26690 | PO:0009001 |
| 753 | AT4G29860 | PO:0009001 |
| 754 | AT3G23150 | PO:0009001 |
| 755 | AT1G05160 | PO:0009001 |
| 756 | AT5G51860 | PO:0009001 |
| 757 | AT5G05340 | PO:0009001 |
| 758 | AT1G11530 | PO:0009001 |
| 759 | AT5G44160 | PO:0009001 |
| 760 | AT2G26250 | PO:0009001 |
| 761 | AT5G11080 | PO:0009001 |
| 762 | AT3G63190 | PO:0009001 |
| 763 | AT3G21160 | PO:0009001 |
| 764 | AT5G63770 | PO:0009001 |
| 765 | AT5G27960 | PO:0009001 |
| 766 | AT1G17110 | PO:0009001 |
| 767 | AT2G37210 | PO:0009001 |
| 768 | AT1G75750 | PO:0009001 |
| 769 | AT4G01060 | PO:0009001 |
| 770 | AT3G17240 | PO:0009001 |
| 771 | AT1G09340 | PO:0009001 |
| 772 | AT1G53310 | PO:0009001 |
| 773 | AT5G08670 | PO:0009001 |
| 774 | AT3G11650 | PO:0009001 |
| 775 | AT2G24640 | PO:0008001, PO:0008002 |
| 776 | AT5G65970 | PO:0009001 |
| 777 | AT5G24820 | PO:0009001 |
| 778 | AT1G78570 | PO:0009001 |
| 779 | AT5G49360 | PO:0009001 |
| 780 | AT2G27190 | PO:0009001 |
| 781 | AT5G55160 | PO:0009001 |
| 782 | AT1G15360 | PO:0009001, PO:0004707 |
| 783 | AT1G07720 | PO:0009001 |
| 784 | AT2G43070 | PO:0009001 |
| 785 | AT1G72990 | PO:0009001 |
| 786 | AT3G21560 | PO:0009001 |
| 787 | AT5G42100 | PO:0009001 |
| 788 | AT1G22640 | PO:0009001 |
| 789 | AT1G42990 | PO:0009001 |
| 790 | AT5G11320 | PO:0009001 |
| 791 | AT3G02360 | PO:0009001 |
| 792 | AT1G01090 | PO:0009001 |
| 793 | AT5G50600 | PO:0009001 |
| 794 | AT1G02790 | PO:0009001 |
| 795 | AT2G14540 | PO:0009001 |
| 796 | AT3G62030 | PO:0009001 |
| 797 | AT5G20490 | PO:0009001 |
| 798 | AT1G10130 | PO:0009001 |
| 799 | AT1G01290 | PO:0009001 |
| 800 | AT3G23820 | PO:0009001 |
| 801 | AT5G09650 | PO:0009001 |
| 802 | AT5G27540 | PO:0009001 |
| 803 | AT3G16150 | PO:0009001 |
| 804 | AT4G25080 | PO:0009001 |
| 805 | AT5G18170 | PO:0009001 |
| 806 | AT1G27680 | PO:0009001 |
| 807 | AT5G47380 | PO:0009001 |
| 808 | AT2G40790 | PO:0009001 |
| 809 | AT1G31140 | PO:0009001 |
| 810 | AT3G21550 | PO:0009001 |
| 811 | AT3G11630 | PO:0009001 |
| 812 | AT4G11010 | PO:0009001 |
| 813 | AT4G33430 | PO:0009001 |
| 814 | AT3G26790 | PO:0009001 |
| 815 | AT3G52960 | PO:0009001 |
| 816 | AT2G31660 | PO:0009001 |
| 817 | AT5G28540 | PO:0009001 |
| 818 | AT4G30270 | PO:0009001 |
| 819 | AT4G34250 | PO:0009001 |
| 820 | AT3G04790 | PO:0009001 |
| 821 | AT5G23540 | PO:0009001 |
| 822 | AT4G39800 | PO:0009001 |
| 823 | AT5G22830 | PO:0009001 |
| 824 | AT3G59990 | PO:0009001 |
| 825 | AT2G21870 | PO:0009001 |
| 826 | AT3G54890 | PO:0009001 |
| 827 | AT4G26260 | PO:0009001 |
| 828 | AT1G79460 | PO:0009001 |
| 829 | AT5G06610 | PO:0009001 |
| 830 | AT3G15030 | PO:0009001 |
| 831 | AT2G03190 | PO:0009001 |
| 832 | AT1G12310 | PO:0009001 |
| 833 | AT3G20820 | PO:0009001 |
| 834 | AT1G65590 | PO:0009001 |
| 835 | AT5G66190 | PO:0009001 |
| 836 | AT5G01410 | PO:0009001 |
| 837 | AT3G11050 | PO:0009001 |
| 838 | AT3G52380 | PO:0009001 |
| 839 | AT3G53450 | PO:0009001 |
| 840 | AT3G55280 | PO:0009001 |
| 841 | AT3G14940 | PO:0009001 |
| 842 | AT3G54140 | PO:0009001, PO:0004536 |
| 843 | AT1G23730 | PO:0009001 |
| 844 | AT3G55800 | PO:0009001 |
| 845 | AT2G34490 | PO:0009001 |
| 846 | AT4G19410 | PO:0009001 |
| 847 | AT5G51480 | PO:0009001 |
| 848 | AT4G31160 | PO:0009001 |
| 849 | AT3G06050 | PO:0009001 |
| 850 | AT3G02730 | PO:0009001 |
| 851 | AT1G03400 | PO:0009001 |
| 852 | AT4G15510 | PO:0009001 |
| 853 | AT4G21080 | PO:0009001 |
| 854 | AT5G63810 | PO:0009001 |
| 855 | AT1G72970 | PO:0009001 |
| 856 | AT5G09360 | PO:0009001 |
| 857 | AT1G64030 | PO:0009001 |
| 858 | AT5G39190 | PO:0009001 |
| 859 | AT5G58070 | PO:0009001 |
| 860 | AT5G24780 | PO:0009001, PO:0000033 |
| 861 | AT3G23810 | PO:0009001 |
| 862 | AT1G04950 | PO:0009001 |
| 863 | AT5G42310 | PO:0009001 |
| 864 | AT2G30970 | PO:0009001 |
| 865 | AT1G53230 | PO:0009001 |
| 866 | AT3G07780 | PO:0009001 |
| 867 | AT1G77510 | PO:0009001 |
| 868 | AT3G21830 | PO:0009001 |
| 869 | AT1G10870 | PO:0009001 |
| 870 | AT4G10450 | PO:0009001 |
| 871 | AT4G30850 | PO:0009001 |
| 872 | AT2G46680 | PO:0009001 |
| 873 | AT2G26550 | PO:0009001 |
| 874 | AT1G09780 | PO:0009001 |
| 875 | AT1G68870 | PO:0008003 |
| 876 | AT4G26140 | PO:0009001 |
| 877 | AT2G31690 | PO:0009001 |
| 878 | AT3G46440 | PO:0009001 |
| 879 | AT3G11660 | PO:0009001 |
| 880 | AT2G16500 | PO:0009001 |
| 881 | AT4G10340 | PO:0009001 |
| 882 | AT5G62680 | PO:0009001 |
| 883 | AT1G55480 | PO:0009001 |
| 884 | AT2G20160 | PO:0009001 |
| 885 | AT4G30440 | PO:0009001 |
| 886 | AT3G63490 | PO:0009001 |
| 887 | AT1G09700 | PO:0009001 |
| 888 | AT1G26960 | PO:0009001 |
| 889 | AT4G24250 | PO:0004535 |
| 890 | AT5G60020 | PO:0009001 |
| 891 | AT1G14520 | PO:0009001 |
| 892 | AT1G05230 | PO:0009001 |
| 893 | AT5G59290 | PO:0009001 |
| 894 | AT5G49740 | PO:0009001 |
| 895 | AT3G45280 | PO:0025268 |
| 896 | AT1G42560 | PO:0009001 |
| 897 | AT2G40220 | PO:0009001 |
| 898 | AT3G03980 | PO:0009001 |
| 899 | AT4G05180 | PO:0009001 |
| 900 | AT1G21760 | PO:0009001 |
| 901 | AT1G04080 | PO:0009001 |
| 902 | AT1G69830 | PO:0009001 |
| 903 | AT4G02770 | PO:0009001 |
| 904 | AT1G55350 | PO:0009001 |
| 905 | AT5G18570 | PO:0009001 |
| 906 | AT2G27860 | PO:0009001 |
| 907 | AT2G40940 | PO:0009001 |
| 908 | AT4G03520 | PO:0009001 |
| 909 | AT4G34520 | PO:0009001 |
| 910 | AT4G06746 | PO:0009001 |
| 911 | AT2G46990 | PO:0009001 |
| 912 | AT3G49780 | PO:0009001 |
| 913 | AT5G50260 | PO:0009001 |
| 914 | AT2G44990 | PO:0009001 |
| 915 | AT2G16430 | PO:0009001 |
| 916 | AT2G33800 | PO:0009001 |
| 917 | AT3G23360 | PO:0009001 |
| 918 | AT1G04410 | PO:0009001 |
| 919 | AT5G57360 | PO:0009001 |
| 920 | AT1G70410 | PO:0009001 |
| 921 | AT5G46210 | PO:0009001 |
| 922 | AT3G10920 | PO:0009001 |
| 923 | AT1G74660 | PO:0009001 |
| 924 | AT5G26930 | PO:0008003 |
| 925 | AT4G35190 | PO:0009001 |
| 926 | AT2G01250 | PO:0009001 |
| 927 | AT1G16060 | PO:0009001 |
| 928 | AT2G24200 | PO:0009001 |
| 929 | AT3G10280 | PO:0009001 |
| 930 | AT1G73370 | PO:0009001 |
| 931 | AT1G67830 | PO:0009001 |
| 932 | AT2G36530 | PO:0009001 |
| 933 | AT3G52820 | PO:0009001 |
| 934 | AT1G80330 | PO:0009001 |
| 935 | AT5G18450 | PO:0009001 |
| 936 | AT4G31120 | PO:0009001 |
| 937 | AT4G25480 | PO:0009001 |
| 938 | AT1G01610 | PO:0009001 |
| 939 | AT1G18450 | PO:0009001 |
| 940 | AT2G45220 | PO:0009001 |
| 941 | AT5G02500 | PO:0009001 |
| 942 | AT4G40060 | PO:0009001 |
| 943 | AT5G08680 | PO:0009001 |
| 944 | AT2G27100 | PO:0009001 |
| 945 | AT1G07750 | PO:0009001 |
| 946 | AT5G20650 | PO:0009001 |
| 947 | AT3G26060 | PO:0009001 |
| 948 | AT5G55220 | PO:0009001 |
| 949 | AT5G48160 | PO:0009001 |
| 950 | AT1G65980 | PO:0009001 |
| 951 | AT3G22200 | PO:0009001 |
| 952 | AT4G08920 | PO:0009001 |
| 953 | AT1G76570 | PO:0009001 |
| 954 | AT2G21590 | PO:0009001 |
| 955 | AT1G19150 | PO:0009001 |
| 956 | AT5G05270 | PO:0009001 |
| 957 | AT1G29260 | PO:0009001 |
| 958 | AT2G30370 | PO:0008002 |
| 959 | AT3G52840 | PO:0009001 |
| 960 | AT5G16310 | PO:0009001 |
| 961 | AT5G13980 | PO:0009001 |
| 962 | AT5G67480 | PO:0009001 |
| 963 | AT5G22650 | PO:0009001 |
| 964 | AT3G57870 | PO:0009001 |
| 965 | AT3G55220 | PO:0009001 |
| 966 | AT5G41520 | PO:0009001 |
| 967 | AT5G65020 | PO:0009001 |
| 968 | AT4G34210 | PO:0000033 |
| 969 | AT4G24230 | PO:0009001 |
| 970 | AT1G56380 | PO:0009001 |
| 971 | AT4G24770 | PO:0009001 |
| 972 | AT4G38220 | PO:0009001 |
| 973 | AT1G79550 | PO:0009001 |
| 974 | AT4G04020 | PO:0009001 |
| 975 | AT3G12780 | PO:0009001 |
| 976 | AT5G50400 | PO:0009001 |
| 977 | AT3G11510 | PO:0009001 |
| 978 | AT1G22590 | PO:0009001 |
| 979 | AT1G66970 | PO:0009001 |
| 980 | AT2G41540 | PO:0009001 |
| 981 | AT1G49240 | PO:0009001 |
| 982 | AT2G16730 | PO:0009001 |
| 983 | AT1G52040 | PO:0009001 |
| 984 | AT5G09810 | PO:0009001 |
| 985 | AT3G20040 | PO:0009001 |
| 986 | AT1G73910 | PO:0009001 |
| 987 | AT3G47990 | PO:0009001 |
| 988 | AT5G49190 | PO:0009001 |
| 989 | AT1G64990 | PO:0009001 |
| 990 | AT3G30720 | PO:0009001 |
| 991 | AT4G21030 | PO:0009001 |
| 992 | AT5G55470 | PO:0009001 |
| 993 | AT5G16990 | PO:0009001 |
| 994 | AT5G48100 | PO:0009001, PO:0000033 |
