# Supplementary material for: Identifying novel fruit-related genes in Arabidopsis thaliana based on the random walk with restart algorithm
Source: PLoS One. 2017 May 4;12(5):e0177017. doi: 10.1371/journal.pone.0177017 (PMC5417634; doi:10.1371/journal.pone.0177017)
Supplement: S2 Table — (DOCX) [file pone.0177017.s002.docx]

**S2 Table.** The 6,310 RWR genes with probabilities larger than 10^-5^.

| **ID** | **Probability** | **P-value** | **MIS** | **MFS** |
| --- | --- | --- | --- | --- |
| AT2G34555 | 1.93E-04 | <0.001 | 925 | 0.778 |
| AT3G54470 | 1.10E-04 | <0.001 | 720 | 0.000 |
| AT3G52590 | 1.05E-04 | <0.001 | 995 | 0.000 |
| AT5G04140 | 9.49E-05 | <0.001 | 999 | 0.000 |
| AT1G23410 | 8.78E-05 | <0.001 | 946 | 0.000 |
| AT4G05320 | 8.37E-05 | <0.001 | 940 | 0.999 |
| AT3G54050 | 8.28E-05 | <0.001 | 995 | 0.000 |
| AT3G29320 | 8.05E-05 | <0.001 | 998 | 0.856 |
| AT4G05050 | 8.00E-05 | <0.001 | 946 | 0.999 |
| AT5G24240 | 7.72E-05 | <0.001 | 946 | 0.957 |
| AT2G26330 | 7.61E-05 | <0.001 | 716 | 0.736 |
| AT5G03240 | 7.56E-05 | <0.001 | 946 | 0.992 |
| AT3G09790 | 7.49E-05 | <0.001 | 946 | 0.999 |
| AT5G37640 | 7.47E-05 | <0.001 | 946 | 0.999 |
| AT4G26530 | 7.17E-05 | <0.001 | 984 | 0.988 |
| AT3G46970 | 7.11E-05 | <0.001 | 998 | 0.792 |
| AT4G26520 | 7.06E-05 | <0.001 | 984 | 0.984 |
| AT1G78630 | 6.95E-05 | <0.001 | 994 | 0.000 |
| AT1G12900 | 6.72E-05 | <0.001 | 997 | 0.000 |
| AT2G01140 | 6.58E-05 | <0.001 | 987 | 0.872 |
| AT5G56350 | 6.56E-05 | <0.001 | 995 | 0.932 |
| AT5G51820 | 6.48E-05 | <0.001 | 998 | 0.976 |
| AT5G23310 | 6.39E-05 | <0.001 | 973 | 0.986 |
| AT4G24620 | 6.39E-05 | <0.001 | 998 | 0.917 |
| AT4G35090 | 6.38E-05 | <0.001 | 993 | 0.909 |
| AT4G30920 | 6.38E-05 | <0.001 | 901 | 0.817 |
| AT1G22300 | 6.14E-05 | <0.001 | 810 | 0.767 |
| AT5G25900 | 6.13E-05 | <0.001 | 992 | 0.936 |
| AT4G25050 | 6.09E-05 | <0.001 | 949 | 0.000 |
| AT1G37130 | 6.05E-05 | <0.001 | 899 | 0.872 |
| AT1G50940 | 6.01E-05 | <0.001 | 721 | 0.756 |
| AT2G22780 | 5.98E-05 | <0.001 | 982 | 0.985 |
| AT3G55360 | 5.81E-05 | <0.001 | 942 | 0.602 |
| AT5G35790 | 5.79E-05 | <0.001 | 981 | 0.000 |
| AT5G20570 | 5.78E-05 | <0.001 | 999 | 0.690 |
| AT4G28660 | 5.75E-05 | <0.001 | 962 | 0.000 |
| AT1G72090 | 5.72E-05 | <0.001 | 601 | 0.787 |
| AT5G19550 | 5.70E-05 | <0.001 | 989 | 0.901 |
| AT5G12370 | 5.70E-05 | <0.001 | 788 | 0.653 |
| AT1G20630 | 5.68E-05 | <0.001 | 995 | 0.853 |
| AT3G24430 | 5.65E-05 | <0.001 | 929 | 0.000 |
| AT3G19820 | 5.65E-05 | <0.001 | 972 | 0.548 |
| AT2G43030 | 5.63E-05 | <0.001 | 996 | 0.000 |
| AT2G26900 | 5.62E-05 | <0.001 | 764 | 0.000 |
| AT5G63680 | 5.61E-05 | <0.001 | 988 | 0.922 |
| AT5G43330 | 5.57E-05 | <0.001 | 972 | 0.974 |
| AT1G44575 | 5.57E-05 | <0.001 | 998 | 0.000 |
| AT1G72610 | 5.54E-05 | <0.001 | 909 | 0.989 |
| AT1G71500 | 5.49E-05 | <0.001 | 944 | 0.000 |
| AT3G22110 | 5.48E-05 | <0.001 | 999 | 0.974 |
| AT4G14890 | 5.44E-05 | <0.001 | 914 | 0.000 |
| AT1G32990 | 5.44E-05 | <0.001 | 991 | 0.000 |
| AT3G29360 | 5.43E-05 | <0.001 | 975 | 0.688 |
| AT5G47190 | 5.43E-05 | <0.001 | 982 | 0.000 |
| AT1G23310 | 5.40E-05 | <0.001 | 964 | 0.000 |
| AT1G23740 | 5.37E-05 | <0.001 | 923 | 0.000 |
| AT2G46880 | 5.35E-05 | <0.001 | 921 | 0.677 |
| AT1G02500 | 5.30E-05 | <0.001 | 984 | 0.000 |
| AT1G49430 | 5.30E-05 | <0.001 | 940 | 0.452 |
| AT3G16250 | 5.29E-05 | <0.001 | 949 | 0.000 |
| AT4G36590 | 5.26E-05 | <0.001 | 865 | 0.946 |
| AT3G61870 | 5.26E-05 | <0.001 | 931 | 0.000 |
| AT1G75350 | 5.25E-05 | <0.001 | 989 | 0.000 |
| AT1G79850 | 5.22E-05 | <0.001 | 981 | 0.000 |
| AT3G13120 | 5.22E-05 | <0.001 | 997 | 0.000 |
| AT1G03970 | 5.22E-05 | <0.001 | 548 | 0.221 |
| AT1G29150 | 5.21E-05 | <0.001 | 998 | 0.655 |
| AT2G43560 | 5.20E-05 | <0.001 | 934 | 0.000 |
| AT2G36580 | 5.20E-05 | <0.001 | 990 | 0.929 |
| AT2G45290 | 5.20E-05 | <0.001 | 972 | 0.897 |
| AT4G24820 | 5.19E-05 | <0.001 | 999 | 0.890 |
| AT4G25100 | 5.17E-05 | <0.001 | 982 | 0.937 |
| AT1G31190 | 5.17E-05 | <0.001 | 963 | 0.972 |
| AT2G30390 | 5.13E-05 | <0.001 | 982 | 0.000 |
| AT1G26570 | 5.13E-05 | <0.001 | 974 | 0.518 |
| AT5G44080 | 5.13E-05 | <0.001 | 718 | 0.571 |
| AT5G67030 | 5.09E-05 | <0.001 | 821 | 0.800 |
| AT2G43090 | 5.07E-05 | <0.001 | 992 | 0.962 |
| AT1G43670 | 5.07E-05 | <0.001 | 966 | 0.000 |
| AT1G62750 | 5.06E-05 | <0.001 | 954 | 0.000 |
| AT3G56350 | 5.05E-05 | <0.001 | 973 | 0.941 |
| AT3G60820 | 5.04E-05 | <0.001 | 999 | 0.897 |
| AT4G24190 | 5.04E-05 | <0.001 | 991 | 0.727 |
| AT4G29060 | 5.03E-05 | <0.001 | 976 | 0.000 |
| AT4G02530 | 4.99E-05 | <0.001 | 996 | 0.000 |
| AT1G75690 | 4.94E-05 | <0.001 | 929 | 0.000 |
| AT5G03690 | 4.93E-05 | <0.001 | 971 | 0.766 |
| AT4G28080 | 4.92E-05 | <0.001 | 873 | 0.000 |
| AT4G17600 | 4.91E-05 | <0.001 | 919 | 0.000 |
| AT5G64380 | 4.90E-05 | <0.001 | 969 | 0.972 |
| AT1G77490 | 4.89E-05 | <0.001 | 922 | 0.000 |
| AT5G15490 | 4.88E-05 | <0.001 | 974 | 0.697 |
| AT4G20360 | 4.88E-05 | <0.001 | 938 | 0.000 |
| AT5G50010 | 4.86E-05 | <0.001 | 375 | 0.844 |
| AT5G30510 | 4.82E-05 | <0.001 | 959 | 0.000 |
| AT2G46280 | 4.79E-05 | <0.001 | 908 | 0.000 |
| AT2G44350 | 4.79E-05 | <0.001 | 988 | 0.984 |
| AT5G13630 | 4.78E-05 | <0.001 | 998 | 0.000 |
| AT2G20890 | 4.75E-05 | <0.001 | 948 | 0.000 |
| AT1G17220 | 4.73E-05 | <0.001 | 859 | 0.000 |
| ATCG00490 | 4.72E-05 | <0.001 | 993 | 0.965 |
| AT5G53460 | 4.71E-05 | <0.001 | 997 | 0.000 |
| AT1G56070 | 4.71E-05 | <0.001 | 963 | 0.985 |
| AT4G01850 | 4.70E-05 | <0.001 | 979 | 0.000 |
| AT1G76100 | 4.69E-05 | <0.001 | 943 | 0.000 |
| AT5G07020 | 4.69E-05 | <0.001 | 921 | 0.000 |
| AT2G46800 | 4.67E-05 | <0.001 | 883 | 0.492 |
| AT5G03940 | 4.67E-05 | <0.001 | 952 | 0.000 |
| AT5G52920 | 4.67E-05 | <0.001 | 988 | 0.940 |
| AT1G52220 | 4.63E-05 | <0.001 | 981 | 0.000 |
| AT3G44890 | 4.63E-05 | <0.001 | 975 | 0.000 |
| AT2G36990 | 4.61E-05 | <0.001 | 891 | 0.000 |
| AT3G54210 | 4.60E-05 | <0.001 | 991 | 0.000 |
| AT2G10940 | 4.60E-05 | <0.001 | 815 | 0.979 |
| AT2G44160 | 4.58E-05 | <0.001 | 980 | 0.888 |
| AT3G42830 | 4.58E-05 | <0.001 | 906 | 0.399 |
| AT4G35250 | 4.57E-05 | <0.001 | 923 | 0.000 |
| AT2G42680 | 4.56E-05 | <0.001 | 599 | 0.712 |
| AT4G21210 | 4.55E-05 | <0.001 | 845 | 0.000 |
| AT1G03680 | 4.53E-05 | <0.001 | 941 | 0.000 |
| AT1G32440 | 4.53E-05 | <0.001 | 986 | 0.937 |
| AT5G38520 | 4.51E-05 | <0.001 | 913 | 0.000 |
| AT5G13420 | 4.50E-05 | <0.001 | 996 | 0.903 |
| AT5G14320 | 4.49E-05 | <0.001 | 977 | 0.000 |
| AT2G05755 | 4.48E-05 | <0.001 | 420 | 0.320 |
| AT3G47070 | 4.48E-05 | <0.001 | 943 | 0.000 |
| AT3G55650 | 4.48E-05 | <0.001 | 987 | 0.928 |
| AT3G02870 | 4.48E-05 | <0.001 | 977 | 0.883 |
| AT1G32080 | 4.48E-05 | <0.001 | 931 | 0.000 |
| AT3G58810 | 4.47E-05 | <0.001 | 746 | 0.614 |
| AT1G77090 | 4.46E-05 | <0.001 | 920 | 0.000 |
| AT3G25960 | 4.44E-05 | <0.001 | 986 | 0.927 |
| AT4G25420 | 4.43E-05 | <0.001 | 953 | 0.664 |
| AT3G55810 | 4.43E-05 | <0.001 | 986 | 0.927 |
| AT3G04050 | 4.43E-05 | <0.001 | 986 | 0.928 |
| AT1G48350 | 4.43E-05 | <0.001 | 994 | 0.000 |
| AT3G10370 | 4.42E-05 | <0.001 | 984 | 0.786 |
| AT4G08870 | 4.41E-05 | <0.001 | 847 | 0.645 |
| AT3G27850 | 4.41E-05 | <0.001 | 977 | 0.000 |
| AT4G09010 | 4.40E-05 | <0.001 | 968 | 0.988 |
| AT5G03650 | 4.37E-05 | <0.001 | 983 | 0.947 |
| AT3G52800 | 4.37E-05 | <0.001 | 728 | 0.853 |
| AT2G03420 | 4.36E-05 | <0.001 | 889 | 0.000 |
| AT3G14440 | 4.36E-05 | <0.001 | 679 | 0.607 |
| AT5G02120 | 4.35E-05 | <0.001 | 931 | 0.000 |
| AT1G74730 | 4.35E-05 | <0.001 | 923 | 0.000 |
| AT1G32550 | 4.35E-05 | <0.001 | 913 | 0.000 |
| AT2G39470 | 4.35E-05 | <0.001 | 966 | 0.000 |
| AT3G08740 | 4.35E-05 | <0.001 | 990 | 0.000 |
| AT5G65010 | 4.33E-05 | <0.001 | 992 | 0.952 |
| AT1G18060 | 4.33E-05 | <0.001 | 873 | 0.987 |
| AT1G67740 | 4.32E-05 | <0.001 | 973 | 0.000 |
| AT2G34860 | 4.32E-05 | <0.001 | 919 | 0.000 |
| AT4G17560 | 4.31E-05 | <0.001 | 983 | 0.000 |
| AT4G24460 | 4.31E-05 | <0.001 | 425 | 0.324 |
| AT5G58260 | 4.31E-05 | <0.001 | 979 | 0.000 |
| AT5G64040 | 4.29E-05 | <0.001 | 984 | 0.000 |
| AT2G42220 | 4.29E-05 | <0.001 | 938 | 0.000 |
| AT4G12800 | 4.27E-05 | <0.001 | 997 | 0.000 |
| AT1G45145 | 4.25E-05 | <0.001 | 808 | 0.000 |
| AT5G36880 | 4.23E-05 | <0.001 | 977 | 0.783 |
| AT1G67700 | 4.22E-05 | <0.001 | 915 | 0.000 |
| AT1G29070 | 4.22E-05 | <0.001 | 923 | 0.000 |
| AT1G51110 | 4.21E-05 | <0.001 | 971 | 0.000 |
| AT1G20340 | 4.19E-05 | <0.001 | 970 | 0.000 |
| AT1G64770 | 4.19E-05 | <0.001 | 909 | 0.000 |
| AT3G47810 | 4.18E-05 | <0.001 | 786 | 0.367 |
| AT5G52100 | 4.17E-05 | <0.001 | 929 | 0.000 |
| AT3G47650 | 4.10E-05 | <0.001 | 881 | 0.000 |
| AT1G12000 | 4.10E-05 | <0.001 | 962 | 0.955 |
| AT4G27700 | 4.09E-05 | <0.001 | 941 | 0.000 |
| AT3G27160 | 4.08E-05 | <0.001 | 911 | 0.000 |
| AT1G48520 | 4.08E-05 | <0.001 | 903 | 0.970 |
| AT1G17840 | 4.08E-05 | <0.001 | 923 | 0.569 |
| AT3G52750 | 4.07E-05 | <0.001 | 860 | 0.000 |
| AT5G08050 | 4.07E-05 | <0.001 | 901 | 0.000 |
| AT1G70820 | 4.06E-05 | <0.001 | 919 | 0.000 |
| AT2G39940 | 4.05E-05 | <0.001 | 999 | 0.583 |
| AT4G24510 | 4.04E-05 | <0.001 | 856 | 0.489 |
| AT1G65230 | 4.04E-05 | <0.001 | 933 | 0.000 |
| AT1G76080 | 4.03E-05 | <0.001 | 875 | 0.000 |
| AT3G46780 | 4.02E-05 | <0.001 | 915 | 0.000 |
| AT1G14320 | 4.01E-05 | <0.001 | 989 | 0.000 |
| AT3G15360 | 4.00E-05 | <0.001 | 953 | 0.000 |
| AT5G47110 | 3.97E-05 | <0.001 | 847 | 0.989 |
| AT5G52970 | 3.96E-05 | <0.001 | 958 | 0.000 |
| AT5G08300 | 3.96E-05 | <0.001 | 999 | 0.969 |
| AT4G16410 | 3.96E-05 | <0.001 | 867 | 0.000 |
| AT5G14910 | 3.95E-05 | <0.001 | 919 | 0.000 |
| AT1G62780 | 3.95E-05 | <0.001 | 917 | 0.000 |
| AT4G34190 | 3.93E-05 | <0.001 | 939 | 0.000 |
| AT3G16140 | 3.92E-05 | <0.001 | 984 | 0.000 |
| AT1G78440 | 3.92E-05 | <0.001 | 928 | 0.758 |
| AT3G04840 | 3.92E-05 | <0.001 | 996 | 0.000 |
| AT1G64510 | 3.89E-05 | <0.001 | 891 | 0.984 |
| AT1G54340 | 3.86E-05 | <0.001 | 976 | 0.938 |
| AT3G15730 | 3.85E-05 | <0.001 | 969 | 0.000 |
| AT3G55410 | 3.83E-05 | <0.001 | 998 | 0.935 |
| AT5G45930 | 3.81E-05 | <0.001 | 997 | 0.000 |
| AT1G74470 | 3.80E-05 | <0.001 | 968 | 0.985 |
| AT5G54600 | 3.80E-05 | <0.001 | 978 | 0.000 |
| AT1G31330 | 3.80E-05 | <0.001 | 996 | 0.000 |
| AT5G18200 | 3.79E-05 | <0.001 | 999 | 0.479 |
| AT1G09310 | 3.78E-05 | <0.001 | 808 | 0.897 |
| AT3G56650 | 3.78E-05 | <0.001 | 958 | 0.000 |
| AT1G26910 | 3.78E-05 | <0.001 | 994 | 0.000 |
| AT5G24520 | 3.77E-05 | <0.001 | 834 | 0.424 |
| AT2G24090 | 3.76E-05 | <0.001 | 891 | 0.000 |
| AT1G76730 | 3.76E-05 | <0.001 | 905 | 0.967 |
| AT2G20260 | 3.75E-05 | <0.001 | 987 | 0.000 |
| AT1G74030 | 3.74E-05 | <0.001 | 994 | 0.979 |
| AT5G39950 | 3.73E-05 | <0.001 | 811 | 0.000 |
| AT1G05180 | 3.72E-05 | <0.001 | 925 | 0.636 |
| AT5G65730 | 3.72E-05 | <0.001 | 726 | 0.978 |
| AT1G18730 | 3.71E-05 | <0.001 | 923 | 0.000 |
| AT3G27830 | 3.71E-05 | <0.001 | 952 | 0.000 |
| AT2G39010 | 3.70E-05 | <0.001 | 688 | 0.830 |
| AT1G54780 | 3.69E-05 | <0.001 | 915 | 0.000 |
| AT1G73230 | 3.68E-05 | <0.001 | 990 | 0.000 |
| AT4G10120 | 3.68E-05 | <0.001 | 919 | 0.000 |
| AT3G51510 | 3.68E-05 | <0.001 | 883 | 0.000 |
| AT2G29560 | 3.67E-05 | <0.001 | 987 | 0.979 |
| AT3G16370 | 3.67E-05 | <0.001 | 875 | 0.642 |
| AT3G15190 | 3.67E-05 | <0.001 | 925 | 0.000 |
| AT5G56030 | 3.66E-05 | <0.001 | 919 | 0.698 |
| AT2G29410 | 3.65E-05 | <0.001 | 368 | 0.350 |
| AT3G61940 | 3.65E-05 | <0.001 | 368 | 0.354 |
| AT3G59780 | 3.64E-05 | <0.001 | 849 | 0.000 |
| AT5G60440 | 3.64E-05 | <0.001 | 844 | 0.928 |
| AT3G58750 | 3.64E-05 | <0.001 | 980 | 0.979 |
| AT2G36320 | 3.64E-05 | <0.001 | 468 | 0.323 |
| AT2G04039 | 3.63E-05 | <0.001 | 901 | 0.000 |
| AT3G52990 | 3.63E-05 | <0.001 | 897 | 0.937 |
| AT1G64680 | 3.63E-05 | <0.001 | 885 | 0.000 |
| AT1G29700 | 3.63E-05 | <0.001 | 798 | 0.968 |
| AT5G05740 | 3.62E-05 | <0.001 | 925 | 0.000 |
| AT2G18850 | 3.61E-05 | <0.001 | 790 | 0.662 |
| AT5G48230 | 3.60E-05 | <0.001 | 916 | 0.868 |
| AT5G26780 | 3.60E-05 | <0.001 | 995 | 0.995 |
| AT5G01530 | 3.60E-05 | <0.001 | 957 | 0.000 |
| AT2G21960 | 3.59E-05 | <0.001 | 879 | 0.000 |
| AT1G79840 | 3.59E-05 | <0.001 | 821 | 0.482 |
| AT5G05780 | 3.58E-05 | <0.001 | 999 | 0.934 |
| AT4G35260 | 3.57E-05 | <0.001 | 985 | 0.911 |
| AT1G33020 | 3.57E-05 | <0.001 | 368 | 0.355 |
| AT2G47170 | 3.56E-05 | <0.001 | 677 | 0.742 |
| AT4G32260 | 3.56E-05 | <0.001 | 968 | 0.000 |
| AT5G50850 | 3.54E-05 | <0.001 | 999 | 0.947 |
| AT1G55670 | 3.54E-05 | <0.001 | 990 | 0.000 |
| AT4G22890 | 3.53E-05 | <0.001 | 926 | 0.000 |
| AT5G49460 | 3.53E-05 | <0.001 | 969 | 0.962 |
| AT1G12230 | 3.53E-05 | <0.001 | 996 | 0.916 |
| AT1G20950 | 3.52E-05 | <0.001 | 947 | 0.953 |
| AT4G01150 | 3.51E-05 | <0.001 | 949 | 0.000 |
| AT5G18660 | 3.51E-05 | <0.001 | 885 | 0.000 |
| AT3G63160 | 3.50E-05 | <0.001 | 744 | 0.000 |
| AT2G29180 | 3.50E-05 | <0.001 | 869 | 0.000 |
| AT4G34090 | 3.50E-05 | <0.001 | 907 | 0.000 |
| AT2G26170 | 3.49E-05 | <0.001 | 966 | 0.000 |
| AT3G61470 | 3.48E-05 | <0.001 | 962 | 0.996 |
| AT4G16155 | 3.47E-05 | <0.001 | 978 | 0.984 |
| AT3G04710 | 3.46E-05 | <0.001 | 872 | 0.940 |
| AT3G13930 | 3.45E-05 | <0.001 | 993 | 0.969 |
| AT3G25920 | 3.45E-05 | <0.001 | 987 | 0.000 |
| AT1G16720 | 3.45E-05 | <0.001 | 793 | 0.973 |
| AT2G38140 | 3.45E-05 | <0.001 | 871 | 0.000 |
| AT1G80340 | 3.44E-05 | <0.001 | 946 | 0.803 |
| AT2G36390 | 3.44E-05 | <0.001 | 983 | 0.813 |
| AT1G60000 | 3.43E-05 | <0.001 | 859 | 0.982 |
| AT5G20000 | 3.42E-05 | <0.001 | 999 | 0.584 |
| AT3G10060 | 3.42E-05 | <0.001 | 907 | 0.000 |
| AT1G12270 | 3.42E-05 | <0.001 | 851 | 0.789 |
| AT1G29395 | 3.40E-05 | <0.001 | 740 | 0.430 |
| AT1G67730 | 3.39E-05 | <0.001 | 809 | 0.549 |
| AT5G10240 | 3.39E-05 | <0.001 | 986 | 0.886 |
| AT5G13650 | 3.39E-05 | <0.001 | 826 | 0.000 |
| AT3G58610 | 3.39E-05 | <0.001 | 976 | 0.000 |
| AT4G00400 | 3.37E-05 | <0.001 | 937 | 0.708 |
| AT5G19940 | 3.37E-05 | <0.001 | 911 | 0.000 |
| AT5G27290 | 3.37E-05 | <0.001 | 893 | 0.000 |
| AT5G65750 | 3.36E-05 | <0.001 | 998 | 0.902 |
| AT1G12250 | 3.36E-05 | <0.001 | 913 | 0.982 |
| AT3G09940 | 3.36E-05 | <0.001 | 991 | 0.963 |
| AT2G41510 | 3.35E-05 | <0.001 | 572 | 0.541 |
| AT5G58730 | 3.35E-05 | <0.001 | 895 | 0.832 |
| AT4G22820 | 3.35E-05 | <0.001 | 501 | 0.486 |
| AT5G27560 | 3.35E-05 | <0.001 | 877 | 0.000 |
| AT1G54350 | 3.35E-05 | <0.001 | 754 | 0.000 |
| AT2G22990 | 3.34E-05 | <0.001 | 758 | 0.925 |
| AT5G57030 | 3.34E-05 | <0.001 | 762 | 0.000 |
| AT1G27400 | 3.34E-05 | <0.001 | 996 | 0.000 |
| AT4G01050 | 3.32E-05 | <0.001 | 911 | 0.000 |
| AT1G45474 | 3.32E-05 | <0.001 | 889 | 0.000 |
| AT5G56630 | 3.31E-05 | <0.001 | 974 | 0.907 |
| AT5G66470 | 3.31E-05 | <0.001 | 889 | 0.986 |
| AT1G63000 | 3.31E-05 | <0.001 | 984 | 0.775 |
| AT5G48790 | 3.31E-05 | <0.001 | 869 | 0.000 |
| AT5G38660 | 3.30E-05 | <0.001 | 895 | 0.000 |
| AT3G09200 | 3.30E-05 | <0.001 | 993 | 0.000 |
| AT1G21720 | 3.29E-05 | <0.001 | 999 | 0.940 |
| AT4G30950 | 3.27E-05 | <0.001 | 833 | 0.000 |
| AT1G77440 | 3.27E-05 | <0.001 | 999 | 0.968 |
| AT1G70760 | 3.27E-05 | <0.001 | 966 | 0.000 |
| AT1G21500 | 3.27E-05 | <0.001 | 881 | 0.000 |
| AT5G17310 | 3.26E-05 | <0.001 | 988 | 0.823 |
| AT5G53580 | 3.26E-05 | <0.001 | 841 | 0.000 |
| AT3G49260 | 3.25E-05 | <0.001 | 563 | 0.916 |
| AT1G19730 | 3.24E-05 | <0.001 | 808 | 0.000 |
| AT4G10300 | 3.21E-05 | <0.001 | 891 | 0.000 |
| AT5G05690 | 3.20E-05 | <0.001 | 911 | 0.959 |
| AT5G18520 | 3.19E-05 | <0.001 | 679 | 0.532 |
| AT3G25660 | 3.18E-05 | <0.001 | 899 | 0.970 |
| AT3G03780 | 3.18E-05 | <0.001 | 998 | 0.934 |
| AT5G16400 | 3.18E-05 | <0.001 | 855 | 0.814 |
| AT5G54770 | 3.17E-05 | <0.001 | 955 | 0.977 |
| AT1G74970 | 3.17E-05 | <0.001 | 940 | 0.000 |
| AT1G64355 | 3.17E-05 | <0.001 | 846 | 0.000 |
| AT2G26500 | 3.16E-05 | <0.001 | 913 | 0.000 |
| AT2G34480 | 3.16E-05 | <0.001 | 996 | 0.000 |
| AT4G26910 | 3.15E-05 | <0.001 | 998 | 0.954 |
| AT5G02630 | 3.15E-05 | <0.001 | 790 | 0.278 |
| AT3G51030 | 3.15E-05 | <0.001 | 809 | 0.000 |
| AT5G59850 | 3.14E-05 | <0.001 | 995 | 0.000 |
| AT3G47800 | 3.13E-05 | <0.001 | 939 | 0.932 |
| AT2G47610 | 3.12E-05 | <0.001 | 996 | 0.000 |
| AT2G36630 | 3.12E-05 | <0.001 | 679 | 0.340 |
| AT5G03880 | 3.11E-05 | <0.001 | 825 | 0.000 |
| AT1G22020 | 3.10E-05 | <0.001 | 994 | 0.997 |
| AT1G73110 | 3.10E-05 | <0.001 | 971 | 0.971 |
| AT4G39970 | 3.09E-05 | <0.001 | 904 | 0.988 |
| AT1G66130 | 3.09E-05 | <0.001 | 822 | 0.947 |
| AT4G27440 | 3.08E-05 | <0.001 | 955 | 0.974 |
| AT3G15110 | 3.08E-05 | <0.001 | 821 | 0.000 |
| AT3G29185 | 3.07E-05 | <0.001 | 881 | 0.000 |
| AT1G30040 | 3.07E-05 | <0.001 | 679 | 0.689 |
| AT2G35260 | 3.07E-05 | <0.001 | 875 | 0.990 |
| AT1G10960 | 3.06E-05 | <0.001 | 822 | 0.932 |
| AT1G52780 | 3.05E-05 | <0.001 | 679 | 0.227 |
| AT1G74880 | 3.05E-05 | <0.001 | 891 | 0.989 |
| AT3G49160 | 3.05E-05 | <0.001 | 968 | 0.916 |
| AT1G30380 | 3.05E-05 | <0.001 | 988 | 0.996 |
| AT1G10670 | 3.04E-05 | <0.001 | 955 | 0.970 |
| AT1G17290 | 3.03E-05 | <0.001 | 929 | 0.903 |
| AT4G25960 | 3.03E-05 | <0.001 | 792 | 0.000 |
| AT5G14760 | 3.03E-05 | <0.001 | 923 | 0.722 |
| AT5G15140 | 3.02E-05 | <0.001 | 939 | 0.895 |
| AT3G56010 | 3.01E-05 | <0.001 | 845 | 0.000 |
| AT5G54270 | 3.00E-05 | <0.001 | 950 | 0.995 |
| AT3G11170 | 3.00E-05 | <0.001 | 696 | 0.000 |
| AT3G12630 | 2.99E-05 | <0.001 | 537 | 0.243 |
| AT5G08690 | 2.99E-05 | <0.001 | 998 | 0.994 |
| AT1G31160 | 2.99E-05 | <0.001 | 453 | 0.938 |
| AT5G40580 | 2.98E-05 | <0.001 | 998 | 0.718 |
| AT1G79210 | 2.98E-05 | <0.001 | 998 | 0.842 |
| AT3G08920 | 2.97E-05 | <0.001 | 869 | 0.000 |
| AT3G19800 | 2.97E-05 | <0.001 | 883 | 0.984 |
| AT3G59090 | 2.97E-05 | <0.001 | 679 | 0.724 |
| AT1G49670 | 2.97E-05 | <0.001 | 788 | 0.839 |
| AT1G66350 | 2.96E-05 | <0.001 | 993 | 0.590 |
| AT3G05010 | 2.95E-05 | <0.001 | 679 | 0.681 |
| AT5G08380 | 2.95E-05 | <0.001 | 905 | 0.582 |
| AT5G57330 | 2.95E-05 | <0.001 | 928 | 0.661 |
| AT3G60100 | 2.94E-05 | <0.001 | 980 | 0.997 |
| AT5G23250 | 2.93E-05 | <0.001 | 997 | 0.977 |
| AT2G18130 | 2.93E-05 | <0.001 | 867 | 0.949 |
| AT2G21385 | 2.93E-05 | <0.001 | 877 | 0.000 |
| AT2G34460 | 2.93E-05 | <0.001 | 813 | 0.971 |
| AT1G73060 | 2.92E-05 | <0.001 | 774 | 0.000 |
| AT5G27210 | 2.92E-05 | <0.001 | 679 | 0.442 |
| AT4G19490 | 2.91E-05 | <0.001 | 999 | 0.501 |
| AT1G52510 | 2.91E-05 | <0.001 | 883 | 0.000 |
| AT3G56490 | 2.91E-05 | <0.001 | 468 | 0.984 |
| AT3G24220 | 2.91E-05 | <0.001 | 877 | 0.606 |
| AT1G15820 | 2.90E-05 | <0.001 | 923 | 0.995 |
| AT1G30950 | 2.90E-05 | <0.001 | 999 | 0.596 |
| AT1G72730 | 2.90E-05 | <0.001 | 925 | 0.989 |
| AT2G06520 | 2.87E-05 | <0.001 | 946 | 0.979 |
| AT5G28750 | 2.87E-05 | <0.001 | 780 | 0.000 |
| AT1G76030 | 2.86E-05 | <0.001 | 991 | 0.908 |
| AT5G08370 | 2.86E-05 | <0.001 | 905 | 0.531 |
| AT2G31040 | 2.85E-05 | <0.001 | 839 | 0.000 |
| AT1G52410 | 2.84E-05 | <0.001 | 900 | 0.644 |
| AT1G62740 | 2.84E-05 | <0.001 | 749 | 0.542 |
| AT2G29360 | 2.83E-05 | <0.001 | 807 | 0.958 |
| AT5G10560 | 2.82E-05 | <0.001 | 790 | 0.827 |
| AT2G38540 | 2.81E-05 | <0.001 | 831 | 0.326 |
| AT3G43720 | 2.81E-05 | <0.001 | 760 | 0.588 |
| AT5G59030 | 2.80E-05 | <0.001 | 621 | 0.797 |
| AT1G17880 | 2.80E-05 | <0.001 | 987 | 0.000 |
| AT4G27600 | 2.79E-05 | <0.001 | 821 | 0.979 |
| AT1G80310 | 2.78E-05 | <0.001 | 677 | 0.502 |
| AT3G27430 | 2.78E-05 | <0.001 | 997 | 0.723 |
| AT2G40840 | 2.78E-05 | <0.001 | 834 | 0.521 |
| AT1G78390 | 2.78E-05 | <0.001 | 877 | 0.584 |
| AT3G18080 | 2.75E-05 | <0.001 | 590 | 0.812 |
| AT2G22480 | 2.74E-05 | <0.001 | 974 | 0.909 |
| AT1G08520 | 2.74E-05 | <0.001 | 998 | 0.000 |
| AT1G05010 | 2.73E-05 | <0.001 | 972 | 0.405 |
| AT2G43750 | 2.71E-05 | <0.001 | 907 | 0.958 |
| AT1G78000 | 2.70E-05 | <0.001 | 955 | 0.290 |
| AT1G16880 | 2.70E-05 | <0.001 | 815 | 0.000 |
| AT1G02560 | 2.69E-05 | <0.001 | 762 | 0.000 |
| AT2G17130 | 2.69E-05 | <0.001 | 979 | 0.908 |
| AT3G55040 | 2.69E-05 | <0.001 | 916 | 0.981 |
| AT1G26220 | 2.69E-05 | <0.001 | 839 | 0.981 |
| AT2G42830 | 2.67E-05 | <0.001 | 932 | 0.863 |
| AT1G64150 | 2.66E-05 | <0.001 | 780 | 0.986 |
| AT5G04470 | 2.66E-05 | <0.001 | 784 | 0.672 |
| AT1G32220 | 2.65E-05 | <0.001 | 883 | 0.000 |
| AT4G18810 | 2.64E-05 | <0.001 | 798 | 0.965 |
| AT5G19760 | 2.64E-05 | <0.001 | 935 | 0.961 |
| AT5G13730 | 2.64E-05 | <0.001 | 798 | 0.977 |
| AT4G13770 | 2.62E-05 | <0.001 | 774 | 0.820 |
| AT5G55070 | 2.60E-05 | <0.001 | 998 | 0.967 |
| AT1G79750 | 2.60E-05 | <0.001 | 979 | 0.877 |
| AT3G15180 | 2.59E-05 | <0.001 | 858 | 0.546 |
| AT1G29930 | 2.59E-05 | <0.001 | 892 | 0.979 |
| AT5G39110 | 2.58E-05 | <0.001 | 599 | 0.470 |
| AT1G12440 | 2.58E-05 | <0.001 | 424 | 0.274 |
| AT1G52340 | 2.58E-05 | <0.001 | 927 | 0.846 |
| AT3G50685 | 2.58E-05 | <0.001 | 855 | 0.986 |
| AT3G52200 | 2.57E-05 | <0.001 | 994 | 0.959 |
| AT4G08900 | 2.57E-05 | <0.001 | 983 | 0.599 |
| AT5G10920 | 2.55E-05 | <0.001 | 962 | 0.971 |
| AT5G10690 | 2.54E-05 | <0.001 | 794 | 0.992 |
| AT3G16240 | 2.54E-05 | <0.001 | 555 | 0.784 |
| AT5G15470 | 2.54E-05 | <0.001 | 899 | 0.610 |
| AT3G25530 | 2.53E-05 | <0.001 | 762 | 0.975 |
| AT5G58240 | 2.53E-05 | <0.001 | 806 | 0.556 |
| AT2G20180 | 2.53E-05 | <0.001 | 819 | 0.724 |
| AT3G62160 | 2.52E-05 | <0.001 | 716 | 0.481 |
| AT4G24930 | 2.51E-05 | <0.001 | 883 | 0.000 |
| AT3G59760 | 2.50E-05 | <0.001 | 906 | 0.977 |
| AT2G32480 | 2.50E-05 | <0.001 | 706 | 0.000 |
| AT3G21720 | 2.48E-05 | <0.001 | 977 | 0.725 |
| AT1G75270 | 2.47E-05 | <0.001 | 977 | 0.587 |
| AT5G43300 | 2.47E-05 | <0.001 | 881 | 0.684 |
| AT2G19900 | 2.46E-05 | <0.001 | 974 | 0.885 |
| AT5G59250 | 2.46E-05 | <0.001 | 815 | 0.000 |
| AT3G54700 | 2.46E-05 | <0.001 | 679 | 0.771 |
| AT5G47210 | 2.45E-05 | <0.001 | 899 | 0.991 |
| AT2G38460 | 2.45E-05 | <0.001 | 428 | 0.501 |
| AT1G02780 | 2.44E-05 | <0.001 | 998 | 0.000 |
| AT4G33790 | 2.44E-05 | <0.001 | 873 | 0.731 |
| AT2G19590 | 2.42E-05 | <0.001 | 953 | 0.633 |
| AT2G13560 | 2.41E-05 | <0.001 | 970 | 0.862 |
| AT2G32700 | 2.41E-05 | <0.001 | 599 | 0.327 |
| AT2G27580 | 2.41E-05 | <0.001 | 424 | 0.369 |
| AT5G48545 | 2.41E-05 | <0.001 | 376 | 0.874 |
| AT3G14600 | 2.40E-05 | <0.001 | 991 | 0.000 |
| AT4G23890 | 2.40E-05 | <0.001 | 798 | 0.984 |
| AT2G35780 | 2.40E-05 | <0.001 | 538 | 0.754 |
| AT2G35580 | 2.39E-05 | <0.001 | 909 | 0.774 |
| AT2G34520 | 2.37E-05 | <0.001 | 901 | 0.000 |
| AT4G14225 | 2.37E-05 | <0.001 | 424 | 0.340 |
| AT2G03550 | 2.36E-05 | <0.001 | 784 | 0.976 |
| AT5G21920 | 2.35E-05 | <0.001 | 547 | 0.000 |
| AT2G34590 | 2.35E-05 | <0.001 | 994 | 0.942 |
| AT1G20260 | 2.35E-05 | <0.001 | 978 | 0.889 |
| AT3G53430 | 2.34E-05 | <0.001 | 998 | 0.000 |
| AT5G46800 | 2.33E-05 | <0.001 | 876 | 0.958 |
| AT4G25380 | 2.33E-05 | <0.001 | 424 | 0.308 |
| AT5G20080 | 2.33E-05 | <0.001 | 671 | 0.901 |
| AT5G55500 | 2.33E-05 | <0.001 | 716 | 0.587 |
| AT3G07480 | 2.32E-05 | <0.001 | 605 | 0.934 |
| AT5G14590 | 2.32E-05 | <0.001 | 969 | 0.969 |
| AT1G06690 | 2.31E-05 | <0.001 | 746 | 0.972 |
| AT1G47220 | 2.29E-05 | <0.001 | 916 | 0.616 |
| AT3G21860 | 2.26E-05 | <0.001 | 748 | 0.967 |
| AT1G02640 | 2.23E-05 | <0.001 | 752 | 0.885 |
| AT5G06070 | 2.22E-05 | <0.001 | 467 | 0.620 |
| AT3G21055 | 2.22E-05 | <0.001 | 942 | 0.995 |
| AT1G50960 | 2.21E-05 | <0.001 | 534 | 0.883 |
| AT5G26820 | 2.20E-05 | <0.001 | 571 | 0.947 |
| AT1G01080 | 2.20E-05 | <0.001 | 733 | 0.985 |
| AT1G15140 | 2.17E-05 | <0.001 | 689 | 0.979 |
| AT1G34000 | 2.17E-05 | <0.001 | 785 | 0.000 |
| AT2G20810 | 2.16E-05 | <0.001 | 900 | 0.493 |
| AT5G45390 | 2.16E-05 | <0.001 | 780 | 0.000 |
| AT1G51400 | 2.15E-05 | <0.001 | 931 | 0.000 |
| AT5G42270 | 2.14E-05 | <0.001 | 956 | 0.996 |
| AT5G45830 | 2.14E-05 | <0.001 | 798 | 0.633 |
| AT2G03710 | 2.13E-05 | <0.001 | 873 | 0.868 |
| AT4G28030 | 2.12E-05 | <0.001 | 742 | 0.977 |
| AT5G13550 | 2.09E-05 | <0.001 | 508 | 0.430 |
| AT1G12410 | 2.09E-05 | <0.001 | 736 | 0.000 |
| AT3G45220 | 2.08E-05 | <0.001 | 912 | 0.756 |
| AT4G18130 | 2.07E-05 | <0.001 | 934 | 0.711 |
| AT5G13400 | 2.07E-05 | <0.001 | 724 | 0.721 |
| AT1G32160 | 2.06E-05 | <0.001 | 706 | 0.977 |
| AT5G19440 | 2.06E-05 | <0.001 | 762 | 0.963 |
| AT1G55680 | 2.06E-05 | <0.001 | 339 | 0.295 |
| AT2G34420 | 2.05E-05 | <0.001 | 957 | 0.990 |
| AT4G27800 | 2.04E-05 | <0.001 | 734 | 0.000 |
| AT5G11720 | 2.04E-05 | <0.001 | 899 | 0.545 |
| AT2G39450 | 2.02E-05 | <0.001 | 718 | 0.514 |
| AT1G54220 | 2.02E-05 | <0.001 | 992 | 0.957 |
| AT4G24750 | 2.01E-05 | <0.001 | 837 | 0.000 |
| AT4G38090 | 2.00E-05 | <0.001 | 488 | 0.343 |
| AT2G46030 | 1.98E-05 | <0.001 | 829 | 0.344 |
| AT5G25880 | 1.97E-05 | <0.001 | 972 | 0.894 |
| AT5G56190 | 1.95E-05 | <0.001 | 298 | 0.480 |
| AT4G03020 | 1.94E-05 | <0.001 | 355 | 0.917 |
| AT4G02730 | 1.93E-05 | <0.001 | 283 | 0.903 |
| AT5G45030 | 1.92E-05 | <0.001 | 156 | 0.365 |
| AT1G74040 | 1.91E-05 | <0.001 | 913 | 0.964 |
| AT5G57345 | 1.90E-05 | <0.001 | 732 | 0.989 |
| AT5G62840 | 1.90E-05 | <0.001 | 776 | 0.985 |
| AT5G66530 | 1.90E-05 | <0.001 | 697 | 0.000 |
| AT4G13090 | 1.89E-05 | <0.001 | 408 | 0.782 |
| AT4G10380 | 1.86E-05 | <0.001 | 623 | 0.978 |
| AT1G30100 | 1.84E-05 | <0.001 | 534 | 0.470 |
| AT5G39160 | 1.83E-05 | <0.001 | 677 | 0.539 |
| AT5G19600 | 1.82E-05 | <0.001 | 837 | 0.288 |
| AT2G30575 | 1.82E-05 | <0.001 | 899 | 0.329 |
| AT5G03730 | 1.82E-05 | <0.001 | 999 | 0.688 |
| AT2G16405 | 1.82E-05 | <0.001 | 283 | 0.269 |
| AT2G34850 | 1.81E-05 | <0.001 | 899 | 0.651 |
| AT1G76550 | 1.78E-05 | <0.001 | 952 | 0.895 |
| AT2G05070 | 1.78E-05 | <0.001 | 920 | 0.987 |
| AT1G61050 | 1.78E-05 | <0.001 | 899 | 0.596 |
| AT4G23730 | 1.77E-05 | <0.001 | 899 | 0.314 |
| AT4G39040 | 1.77E-05 | <0.001 | 784 | 0.980 |
| AT2G01620 | 1.77E-05 | <0.001 | 855 | 0.805 |
| AT3G23760 | 1.75E-05 | <0.001 | 732 | 0.973 |
| AT5G28030 | 1.75E-05 | <0.001 | 907 | 0.979 |
| AT3G22460 | 1.75E-05 | <0.001 | 906 | 0.960 |
| AT5G14260 | 1.73E-05 | <0.001 | 808 | 0.984 |
| AT3G15520 | 1.73E-05 | <0.001 | 776 | 0.974 |
| AT5G56500 | 1.72E-05 | <0.001 | 946 | 0.984 |
| AT1G22700 | 1.72E-05 | <0.001 | 762 | 0.000 |
| AT3G25770 | 1.71E-05 | <0.001 | 969 | 0.443 |
| AT5G55120 | 1.71E-05 | <0.001 | 790 | 0.464 |
| AT5G17870 | 1.70E-05 | <0.001 | 760 | 0.973 |
| AT1G06020 | 1.67E-05 | <0.001 | 931 | 0.939 |
| AT4G35390 | 1.66E-05 | <0.001 | 659 | 0.456 |
| AT1G64690 | 1.65E-05 | <0.001 | 506 | 0.447 |
| AT1G13590 | 1.64E-05 | <0.001 | 966 | 0.287 |
| AT1G60810 | 1.63E-05 | <0.001 | 948 | 0.953 |
| AT5G40380 | 1.63E-05 | <0.001 | 346 | 0.780 |
| AT5G18090 | 1.60E-05 | <0.001 | 466 | 0.545 |
| AT4G37510 | 1.59E-05 | <0.001 | 700 | 0.987 |
| AT5G12040 | 1.58E-05 | <0.001 | 901 | 0.464 |
| AT2G01870 | 1.55E-05 | <0.001 | 629 | 0.963 |
| AT4G17830 | 1.54E-05 | <0.001 | 944 | 0.923 |
| AT1G77060 | 1.51E-05 | <0.001 | 697 | 0.971 |
| AT4G10260 | 1.47E-05 | <0.001 | 919 | 0.936 |
| AT1G59960 | 1.46E-05 | <0.001 | 659 | 0.530 |
| AT1G69935 | 1.46E-05 | <0.001 | 677 | 0.922 |
| AT2G44060 | 1.46E-05 | <0.001 | 402 | 0.576 |
| AT5G22620 | 1.45E-05 | <0.001 | 881 | 0.963 |
| AT2G30695 | 1.45E-05 | <0.001 | 704 | 0.988 |
| AT1G31830 | 1.43E-05 | <0.001 | 453 | 0.599 |
| AT3G26720 | 1.42E-05 | <0.001 | 919 | 0.554 |
| AT5G37300 | 1.35E-05 | <0.001 | 599 | 0.669 |
| AT1G78995 | 1.34E-05 | <0.001 | 587 | 0.982 |
| AT3G44020 | 1.34E-05 | <0.001 | 641 | 0.981 |
| AT2G33180 | 1.31E-05 | <0.001 | 631 | 0.973 |
| AT2G21370 | 1.30E-05 | <0.001 | 914 | 0.950 |
| AT2G42780 | 1.30E-05 | <0.001 | 699 | 0.261 |
| AT4G16100 | 1.30E-05 | <0.001 | 368 | 0.996 |
| AT2G01260 | 1.29E-05 | <0.001 | 368 | 0.317 |
| AT1G19600 | 1.27E-05 | <0.001 | 437 | 0.866 |
| AT5G14470 | 1.25E-05 | <0.001 | 563 | 0.354 |
| AT3G03080 | 1.24E-05 | <0.001 | 599 | 0.747 |
| AT5G34780 | 1.23E-05 | <0.001 | 971 | 0.710 |
| AT5G50950 | 1.20E-05 | <0.001 | 809 | 0.986 |
| AT5G09300 | 1.17E-05 | <0.001 | 971 | 0.944 |
| AT3G48720 | 1.15E-05 | <0.001 | 490 | 0.897 |
| AT2G24280 | 1.10E-05 | <0.001 | 441 | 0.895 |
| AT3G01640 | 1.09E-05 | <0.001 | 432 | 0.293 |
| AT3G61610 | 1.09E-05 | <0.001 | 899 | 0.615 |
| AT4G13080 | 1.04E-05 | <0.001 | 283 | 0.770 |
| AT4G13500 | 1.04E-05 | <0.001 | 510 | 0.987 |
| AT2G38380 | 1.03E-05 | <0.001 | 899 | 0.581 |
| AT3G46520 | 8.43E-05 | 0.001 | 982 | 0.771 |
| AT3G24310 | 6.12E-05 | 0.001 | 883 | 0.967 |
| AT1G03630 | 5.89E-05 | 0.001 | 985 | 0.000 |
| AT5G23060 | 5.41E-05 | 0.001 | 933 | 0.000 |
| AT3G02090 | 4.99E-05 | 0.001 | 989 | 0.953 |
| AT5G08570 | 4.88E-05 | 0.001 | 987 | 0.941 |
| AT1G54500 | 4.42E-05 | 0.001 | 901 | 0.000 |
| AT4G17260 | 4.36E-05 | 0.001 | 918 | 0.937 |
| AT1G51500 | 4.35E-05 | 0.001 | 967 | 0.559 |
| AT1G32200 | 4.31E-05 | 0.001 | 923 | 0.000 |
| AT5G61850 | 4.30E-05 | 0.001 | 790 | 0.663 |
| AT2G36270 | 4.28E-05 | 0.001 | 993 | 0.694 |
| AT5G43750 | 4.20E-05 | 0.001 | 917 | 0.000 |
| AT1G03475 | 4.14E-05 | 0.001 | 997 | 0.000 |
| AT5G20630 | 4.09E-05 | 0.001 | 744 | 0.980 |
| AT3G19480 | 3.93E-05 | 0.001 | 853 | 0.978 |
| AT3G25860 | 3.93E-05 | 0.001 | 975 | 0.974 |
| AT4G39350 | 3.88E-05 | 0.001 | 718 | 0.539 |
| AT4G18960 | 3.84E-05 | 0.001 | 985 | 0.835 |
| AT1G07030 | 3.82E-05 | 0.001 | 679 | 0.411 |
| AT4G16130 | 3.82E-05 | 0.001 | 872 | 0.920 |
| AT1G12640 | 3.76E-05 | 0.001 | 915 | 0.390 |
| AT1G64670 | 3.71E-05 | 0.001 | 875 | 0.668 |
| AT4G33400 | 3.67E-05 | 0.001 | 679 | 0.777 |
| AT2G47450 | 3.61E-05 | 0.001 | 947 | 0.906 |
| AT5G59750 | 3.59E-05 | 0.001 | 829 | 0.000 |
| AT5G02160 | 3.59E-05 | 0.001 | 933 | 0.992 |
| AT1G66430 | 3.54E-05 | 0.001 | 919 | 0.947 |
| AT3G26340 | 3.54E-05 | 0.001 | 998 | 0.533 |
| AT3G17810 | 3.53E-05 | 0.001 | 463 | 0.843 |
| AT1G63050 | 3.46E-05 | 0.001 | 915 | 0.454 |
| AT3G47930 | 3.44E-05 | 0.001 | 963 | 0.856 |
| AT3G07770 | 3.39E-05 | 0.001 | 965 | 0.855 |
| AT5G56000 | 3.32E-05 | 0.001 | 916 | 0.701 |
| AT4G09000 | 3.31E-05 | 0.001 | 940 | 0.945 |
| AT2G41220 | 3.27E-05 | 0.001 | 985 | 0.000 |
| AT1G14270 | 3.26E-05 | 0.001 | 879 | 0.000 |
| AT1G07020 | 3.25E-05 | 0.001 | 424 | 0.677 |
| AT1G07025 | 3.25E-05 | 0.001 | 424 | 0.458 |
| AT4G18395 | 3.13E-05 | 0.001 | 919 | 0.000 |
| AT4G13890 | 3.11E-05 | 0.001 | 995 | 0.997 |
| AT2G37420 | 3.11E-05 | 0.001 | 675 | 0.781 |
| AT1G09430 | 3.09E-05 | 0.001 | 951 | 0.946 |
| AT5G64860 | 3.08E-05 | 0.001 | 976 | 0.783 |
| AT5G42970 | 3.07E-05 | 0.001 | 733 | 0.573 |
| AT5G27470 | 3.07E-05 | 0.001 | 623 | 0.975 |
| AT3G55630 | 3.05E-05 | 0.001 | 835 | 0.967 |
| AT2G36200 | 3.04E-05 | 0.001 | 689 | 0.805 |
| AT2G18170 | 3.02E-05 | 0.001 | 791 | 0.000 |
| AT3G26090 | 3.01E-05 | 0.001 | 598 | 0.564 |
| AT1G69120 | 3.00E-05 | 0.001 | 770 | 0.800 |
| AT4G37910 | 2.99E-05 | 0.001 | 945 | 0.000 |
| AT5G54960 | 2.98E-05 | 0.001 | 933 | 0.701 |
| AT1G79530 | 2.98E-05 | 0.001 | 998 | 0.997 |
| AT4G35790 | 2.95E-05 | 0.001 | 972 | 0.000 |
| AT1G11750 | 2.95E-05 | 0.001 | 979 | 0.000 |
| AT1G09010 | 2.89E-05 | 0.001 | 690 | 0.789 |
| AT5G16390 | 2.83E-05 | 0.001 | 999 | 0.970 |
| AT1G36370 | 2.82E-05 | 0.001 | 994 | 0.997 |
| AT1G65620 | 2.82E-05 | 0.001 | 875 | 0.630 |
| AT5G43350 | 2.81E-05 | 0.001 | 788 | 0.598 |
| AT1G30230 | 2.73E-05 | 0.001 | 801 | 0.000 |
| AT5G36220 | 2.71E-05 | 0.001 | 657 | 0.963 |
| AT1G07110 | 2.64E-05 | 0.001 | 962 | 0.960 |
| AT3G55120 | 2.62E-05 | 0.001 | 999 | 0.645 |
| AT1G23490 | 2.62E-05 | 0.001 | 642 | 0.574 |
| AT1G75330 | 2.62E-05 | 0.001 | 958 | 0.970 |
| AT2G04570 | 2.61E-05 | 0.001 | 748 | 0.614 |
| AT2G21160 | 2.56E-05 | 0.001 | 964 | 0.978 |
| AT4G00110 | 2.56E-05 | 0.001 | 899 | 0.000 |
| AT1G67070 | 2.56E-05 | 0.001 | 998 | 0.534 |
| AT5G14420 | 2.54E-05 | 0.001 | 677 | 0.242 |
| AT5G53120 | 2.54E-05 | 0.001 | 904 | 0.823 |
| AT1G15260 | 2.53E-05 | 0.001 | 613 | 0.926 |
| AT3G24830 | 2.52E-05 | 0.001 | 990 | 0.000 |
| AT2G26390 | 2.46E-05 | 0.001 | 909 | 0.533 |
| AT1G29910 | 2.45E-05 | 0.001 | 945 | 0.976 |
| AT4G37980 | 2.43E-05 | 0.001 | 595 | 0.646 |
| AT4G35460 | 2.43E-05 | 0.001 | 798 | 0.940 |
| AT3G09580 | 2.41E-05 | 0.001 | 808 | 0.846 |
| AT5G37360 | 2.40E-05 | 0.001 | 800 | 0.986 |
| AT4G26370 | 2.36E-05 | 0.001 | 776 | 0.000 |
| AT1G79440 | 2.34E-05 | 0.001 | 861 | 0.860 |
| AT1G15700 | 2.32E-05 | 0.001 | 991 | 0.988 |
| AT2G02850 | 2.27E-05 | 0.001 | 507 | 0.400 |
| AT4G02440 | 2.25E-05 | 0.001 | 999 | 0.609 |
| AT2G47390 | 2.22E-05 | 0.001 | 591 | 0.586 |
| AT5G10180 | 2.19E-05 | 0.001 | 693 | 0.280 |
| AT4G37560 | 2.19E-05 | 0.001 | 907 | 0.434 |
| AT4G16210 | 2.18E-05 | 0.001 | 899 | 0.833 |
| AT3G51140 | 2.13E-05 | 0.001 | 776 | 0.000 |
| AT2G39090 | 2.13E-05 | 0.001 | 955 | 0.598 |
| AT5G28020 | 2.09E-05 | 0.001 | 907 | 0.979 |
| AT3G25400 | 2.08E-05 | 0.001 | 243 | 0.609 |
| AT1G67760 | 2.06E-05 | 0.001 | 720 | 0.994 |
| AT4G02520 | 2.04E-05 | 0.001 | 939 | 0.976 |
| AT1G50480 | 1.98E-05 | 0.001 | 992 | 0.877 |
| AT3G49120 | 1.89E-05 | 0.001 | 899 | 0.529 |
| AT4G19150 | 1.87E-05 | 0.001 | 506 | 0.953 |
| AT5G22860 | 1.83E-05 | 0.001 | 679 | 0.313 |
| AT4G35010 | 1.81E-05 | 0.001 | 500 | 0.988 |
| AT5G45650 | 1.80E-05 | 0.001 | 537 | 0.898 |
| AT3G11090 | 1.76E-05 | 0.001 | 659 | 0.975 |
| AT2G35840 | 1.76E-05 | 0.001 | 661 | 0.804 |
| AT4G37800 | 1.70E-05 | 0.001 | 762 | 0.881 |
| AT3G01590 | 1.70E-05 | 0.001 | 899 | 0.351 |
| AT1G06780 | 1.67E-05 | 0.001 | 899 | 0.413 |
| AT4G36530 | 1.67E-05 | 0.001 | 933 | 0.977 |
| AT2G04700 | 1.65E-05 | 0.001 | 655 | 0.000 |
| AT4G30290 | 1.63E-05 | 0.001 | 428 | 0.582 |
| AT1G20980 | 1.61E-05 | 0.001 | 508 | 0.190 |
| AT3G01010 | 1.60E-05 | 0.001 | 700 | 0.655 |
| AT5G06580 | 1.58E-05 | 0.001 | 918 | 0.771 |
| AT1G47990 | 1.55E-05 | 0.001 | 408 | 0.681 |
| AT1G51420 | 1.48E-05 | 0.001 | 643 | 0.596 |
| AT4G28320 | 1.48E-05 | 0.001 | 744 | 0.346 |
| AT1G07440 | 1.46E-05 | 0.001 | 701 | 0.898 |
| AT5G62165 | 1.42E-05 | 0.001 | 409 | 0.885 |
| AT4G39990 | 1.41E-05 | 0.001 | 649 | 0.613 |
| AT3G59480 | 1.37E-05 | 0.001 | 919 | 0.950 |
| AT4G27570 | 1.31E-05 | 0.001 | 479 | 0.636 |
| AT2G32690 | 1.24E-05 | 0.001 | 970 | 0.662 |
| AT1G35515 | 1.03E-05 | 0.001 | 463 | 0.971 |
| AT1G06180 | 5.75E-05 | 0.002 | 883 | 0.969 |
| AT1G55490 | 5.64E-05 | 0.002 | 989 | 0.000 |
| AT2G42790 | 5.42E-05 | 0.002 | 997 | 0.970 |
| AT5G45680 | 5.10E-05 | 0.002 | 925 | 0.000 |
| AT1G60550 | 4.96E-05 | 0.002 | 859 | 0.000 |
| AT4G13430 | 4.73E-05 | 0.002 | 994 | 0.911 |
| AT3G22960 | 4.73E-05 | 0.002 | 987 | 0.941 |
| AT3G54440 | 4.25E-05 | 0.002 | 936 | 0.816 |
| AT2G18790 | 4.22E-05 | 0.002 | 999 | 0.741 |
| AT4G32551 | 3.93E-05 | 0.002 | 716 | 0.773 |
| AT1G77760 | 3.86E-05 | 0.002 | 910 | 0.878 |
| AT2G30160 | 3.85E-05 | 0.002 | 679 | 0.415 |
| AT4G11260 | 3.77E-05 | 0.002 | 976 | 0.477 |
| AT4G39710 | 3.76E-05 | 0.002 | 963 | 0.000 |
| AT1G68050 | 3.49E-05 | 0.002 | 988 | 0.705 |
| AT3G17820 | 3.45E-05 | 0.002 | 985 | 0.913 |
| AT1G20620 | 3.35E-05 | 0.002 | 901 | 0.861 |
| AT4G26300 | 3.33E-05 | 0.002 | 578 | 0.982 |
| AT4G23600 | 3.20E-05 | 0.002 | 869 | 0.842 |
| AT5G66760 | 3.18E-05 | 0.002 | 989 | 0.951 |
| AT1G06460 | 3.13E-05 | 0.002 | 831 | 0.917 |
| AT2G36145 | 3.12E-05 | 0.002 | 914 | 0.000 |
| AT3G02350 | 3.08E-05 | 0.002 | 920 | 0.641 |
| AT1G74070 | 3.07E-05 | 0.002 | 837 | 0.976 |
| AT3G55250 | 2.97E-05 | 0.002 | 951 | 0.000 |
| AT4G33070 | 2.96E-05 | 0.002 | 935 | 0.584 |
| AT4G11600 | 2.94E-05 | 0.002 | 913 | 0.732 |
| AT5G47720 | 2.86E-05 | 0.002 | 916 | 0.835 |
| AT4G16566 | 2.85E-05 | 0.002 | 577 | 0.375 |
| AT3G10970 | 2.75E-05 | 0.002 | 970 | 0.982 |
| AT3G51770 | 2.73E-05 | 0.002 | 990 | 0.507 |
| AT1G26880 | 2.72E-05 | 0.002 | 994 | 0.000 |
| AT4G37870 | 2.68E-05 | 0.002 | 990 | 0.782 |
| AT2G01590 | 2.57E-05 | 0.002 | 859 | 0.980 |
| AT3G21370 | 2.52E-05 | 0.002 | 534 | 0.820 |
| AT3G02570 | 2.50E-05 | 0.002 | 992 | 0.530 |
| AT5G64120 | 2.49E-05 | 0.002 | 899 | 0.574 |
| AT4G17360 | 2.47E-05 | 0.002 | 990 | 0.878 |
| AT5G13120 | 2.45E-05 | 0.002 | 734 | 0.000 |
| AT3G02660 | 2.44E-05 | 0.002 | 506 | 0.974 |
| AT1G18170 | 2.42E-05 | 0.002 | 851 | 0.000 |
| AT4G29590 | 2.32E-05 | 0.002 | 760 | 0.975 |
| AT3G14150 | 2.30E-05 | 0.002 | 874 | 0.791 |
| AT1G78580 | 2.29E-05 | 0.002 | 939 | 0.708 |
| AT3G63440 | 2.29E-05 | 0.002 | 572 | 0.546 |
| AT4G37190 | 2.27E-05 | 0.002 | 637 | 0.387 |
| AT5G51830 | 2.25E-05 | 0.002 | 920 | 0.898 |
| AT3G05560 | 2.25E-05 | 0.002 | 994 | 0.000 |
| AT5G44520 | 2.17E-05 | 0.002 | 971 | 0.968 |
| AT1G04920 | 2.15E-05 | 0.002 | 927 | 0.000 |
| AT1G10360 | 2.04E-05 | 0.002 | 806 | 0.985 |
| AT1G44920 | 2.02E-05 | 0.002 | 732 | 0.982 |
| AT5G57540 | 1.98E-05 | 0.002 | 407 | 0.774 |
| AT2G39960 | 1.98E-05 | 0.002 | 952 | 0.878 |
| AT1G16570 | 1.96E-05 | 0.002 | 889 | 0.000 |
| AT4G34260 | 1.94E-05 | 0.002 | 966 | 0.497 |
| AT2G03430 | 1.87E-05 | 0.002 | 891 | 0.706 |
| AT4G21860 | 1.84E-05 | 0.002 | 708 | 0.958 |
| AT3G01040 | 1.82E-05 | 0.002 | 899 | 0.610 |
| AT2G44050 | 1.74E-05 | 0.002 | 658 | 0.974 |
| AT1G23780 | 1.71E-05 | 0.002 | 805 | 0.578 |
| AT5G42765 | 1.65E-05 | 0.002 | 880 | 0.982 |
| AT1G49970 | 1.56E-05 | 0.002 | 844 | 0.000 |
| AT4G23920 | 1.51E-05 | 0.002 | 957 | 0.663 |
| AT1G19990 | 1.41E-05 | 0.002 | 786 | 0.973 |
| AT3G52340 | 1.32E-05 | 0.002 | 659 | 0.584 |
| AT1G69880 | 1.29E-05 | 0.002 | 501 | 0.000 |
| AT1G59950 | 1.28E-05 | 0.002 | 659 | 0.490 |
| AT5G39130 | 1.28E-05 | 0.002 | 690 | 0.621 |
| AT3G54270 | 1.27E-05 | 0.002 | 535 | 0.583 |
| AT3G45050 | 1.24E-05 | 0.002 | 685 | 0.978 |
| AT1G47330 | 1.23E-05 | 0.002 | 285 | 0.894 |
| AT5G48070 | 1.18E-05 | 0.002 | 241 | 0.607 |
| AT5G57490 | 1.17E-05 | 0.002 | 568 | 0.908 |
| AT4G09460 | 1.14E-05 | 0.002 | 409 | 0.971 |
| AT4G39980 | 1.11E-05 | 0.002 | 878 | 0.567 |
| AT5G22020 | 1.11E-05 | 0.002 | 312 | 0.492 |
| AT2G07732 | 1.00E-05 | 0.002 | 847 | 0.935 |
| AT2G39800 | 6.34E-05 | 0.003 | 978 | 0.867 |
| AT2G35370 | 6.10E-05 | 0.003 | 998 | 0.000 |
| AT1G13060 | 5.28E-05 | 0.003 | 999 | 0.921 |
| AT5G17230 | 5.06E-05 | 0.003 | 940 | 0.895 |
| AT3G25140 | 4.56E-05 | 0.003 | 916 | 0.811 |
| AT3G58990 | 4.16E-05 | 0.003 | 992 | 0.902 |
| AT3G61130 | 4.12E-05 | 0.003 | 936 | 0.774 |
| AT4G04780 | 3.82E-05 | 0.003 | 899 | 0.511 |
| AT1G66580 | 3.58E-05 | 0.003 | 973 | 0.000 |
| AT1G76110 | 3.54E-05 | 0.003 | 823 | 0.946 |
| AT2G28950 | 3.49E-05 | 0.003 | 653 | 0.901 |
| AT4G05440 | 3.35E-05 | 0.003 | 384 | 0.653 |
| AT5G42090 | 3.30E-05 | 0.003 | 679 | 0.344 |
| AT2G25080 | 3.28E-05 | 0.003 | 906 | 0.980 |
| AT5G23940 | 3.23E-05 | 0.003 | 704 | 0.561 |
| AT3G09260 | 3.16E-05 | 0.003 | 625 | 0.812 |
| AT3G10700 | 3.08E-05 | 0.003 | 912 | 0.946 |
| AT3G45850 | 3.04E-05 | 0.003 | 675 | 0.661 |
| AT3G09210 | 3.03E-05 | 0.003 | 859 | 0.000 |
| AT5G47840 | 2.88E-05 | 0.003 | 922 | 0.000 |
| AT3G61080 | 2.80E-05 | 0.003 | 874 | 0.982 |
| AT5G49730 | 2.59E-05 | 0.003 | 762 | 0.898 |
| AT4G27150 | 2.56E-05 | 0.003 | 905 | 0.716 |
| AT5G20710 | 2.54E-05 | 0.003 | 786 | 0.929 |
| AT5G04900 | 2.53E-05 | 0.003 | 925 | 0.763 |
| AT2G34620 | 2.52E-05 | 0.003 | 843 | 0.988 |
| AT1G08190 | 2.49E-05 | 0.003 | 870 | 0.286 |
| AT3G18060 | 2.44E-05 | 0.003 | 933 | 0.506 |
| AT2G32730 | 2.40E-05 | 0.003 | 962 | 0.659 |
| AT4G24450 | 2.38E-05 | 0.003 | 790 | 0.374 |
| AT5G44480 | 2.35E-05 | 0.003 | 899 | 0.632 |
| AT4G18910 | 2.25E-05 | 0.003 | 742 | 0.991 |
| AT3G06810 | 2.10E-05 | 0.003 | 949 | 0.496 |
| AT1G48900 | 2.09E-05 | 0.003 | 971 | 0.979 |
| AT1G04880 | 2.05E-05 | 0.003 | 823 | 0.629 |
| AT3G28910 | 2.02E-05 | 0.003 | 409 | 0.915 |
| AT1G17200 | 1.96E-05 | 0.003 | 712 | 0.928 |
| AT1G67490 | 1.92E-05 | 0.003 | 969 | 0.470 |
| AT5G61580 | 1.90E-05 | 0.003 | 974 | 0.891 |
| AT1G17210 | 1.87E-05 | 0.003 | 408 | 0.402 |
| AT5G15970 | 1.79E-05 | 0.003 | 838 | 0.500 |
| AT4G30910 | 1.70E-05 | 0.003 | 901 | 0.782 |
| AT5G43440 | 1.69E-05 | 0.003 | 720 | 0.718 |
| AT4G23590 | 1.61E-05 | 0.003 | 435 | 0.865 |
| AT3G27300 | 1.52E-05 | 0.003 | 979 | 0.893 |
| AT1G48950 | 1.50E-05 | 0.003 | 384 | 0.343 |
| ATMG00290 | 1.47E-05 | 0.003 | 407 | 0.947 |
| AT5G49200 | 1.33E-05 | 0.003 | 636 | 0.487 |
| AT1G45050 | 1.29E-05 | 0.003 | 599 | 0.623 |
| AT5G57550 | 1.25E-05 | 0.003 | 504 | 0.821 |
| AT1G03270 | 1.24E-05 | 0.003 | 285 | 0.891 |
| AT4G35600 | 1.21E-05 | 0.003 | 627 | 0.940 |
| AT1G17150 | 1.02E-05 | 0.003 | 463 | 0.584 |
| AT4G02570 | 5.42E-05 | 0.004 | 999 | 0.929 |
| AT5G11520 | 5.25E-05 | 0.004 | 982 | 0.860 |
| AT1G52230 | 4.67E-05 | 0.004 | 985 | 0.000 |
| AT3G24500 | 4.22E-05 | 0.004 | 566 | 0.834 |
| AT4G23710 | 3.49E-05 | 0.004 | 967 | 0.729 |
| AT2G01570 | 3.48E-05 | 0.004 | 999 | 0.583 |
| AT3G15990 | 3.44E-05 | 0.004 | 643 | 0.320 |
| AT2G30920 | 3.19E-05 | 0.004 | 688 | 0.728 |
| AT1G08550 | 3.12E-05 | 0.004 | 839 | 0.000 |
| AT5G44650 | 3.07E-05 | 0.004 | 853 | 0.000 |
| AT1G08380 | 3.07E-05 | 0.004 | 916 | 0.975 |
| AT4G00370 | 2.92E-05 | 0.004 | 782 | 0.000 |
| AT1G26630 | 2.85E-05 | 0.004 | 975 | 0.000 |
| AT5G19690 | 2.77E-05 | 0.004 | 900 | 0.813 |
| AT2G17845 | 2.60E-05 | 0.004 | 679 | 0.925 |
| AT5G13770 | 2.58E-05 | 0.004 | 813 | 0.978 |
| AT1G04640 | 2.56E-05 | 0.004 | 696 | 0.000 |
| AT3G14130 | 2.53E-05 | 0.004 | 874 | 0.775 |
| AT1G26310 | 2.48E-05 | 0.004 | 551 | 0.874 |
| AT3G61590 | 2.35E-05 | 0.004 | 908 | 0.311 |
| AT4G00860 | 2.32E-05 | 0.004 | 825 | 0.960 |
| AT1G07880 | 2.14E-05 | 0.004 | 816 | 0.000 |
| AT4G05390 | 2.13E-05 | 0.004 | 411 | 0.000 |
| AT4G15210 | 2.13E-05 | 0.004 | 501 | 0.361 |
| AT1G79040 | 2.10E-05 | 0.004 | 977 | 0.991 |
| AT1G11430 | 2.04E-05 | 0.004 | 894 | 0.963 |
| AT4G28780 | 1.99E-05 | 0.004 | 581 | 0.958 |
| AT3G18500 | 1.97E-05 | 0.004 | 533 | 0.928 |
| AT1G04140 | 1.94E-05 | 0.004 | 283 | 0.274 |
| AT4G18870 | 1.78E-05 | 0.004 | 534 | 0.768 |
| AT5G58840 | 1.76E-05 | 0.004 | 433 | 0.946 |
| AT1G49190 | 1.67E-05 | 0.004 | 647 | 0.942 |
| AT5G24260 | 1.60E-05 | 0.004 | 408 | 0.609 |
| AT4G08980 | 1.52E-05 | 0.004 | 855 | 0.568 |
| AT3G22425 | 1.42E-05 | 0.004 | 989 | 0.880 |
| AT1G79320 | 1.35E-05 | 0.004 | 541 | 0.285 |
| AT2G45410 | 1.31E-05 | 0.004 | 671 | 0.270 |
| AT5G06720 | 1.26E-05 | 0.004 | 899 | 0.587 |
| AT5G66350 | 1.24E-05 | 0.004 | 677 | 0.457 |
| AT3G23730 | 1.22E-05 | 0.004 | 593 | 0.654 |
| AT5G15310 | 1.15E-05 | 0.004 | 435 | 0.972 |
| AT3G56800 | 4.94E-05 | 0.005 | 901 | 0.900 |
| AT3G15850 | 4.36E-05 | 0.005 | 857 | 0.980 |
| AT5G35170 | 4.04E-05 | 0.005 | 946 | 0.000 |
| AT5G42790 | 3.92E-05 | 0.005 | 999 | 0.970 |
| AT4G36920 | 3.82E-05 | 0.005 | 790 | 0.665 |
| AT3G14290 | 3.65E-05 | 0.005 | 998 | 0.979 |
| AT2G24820 | 3.13E-05 | 0.005 | 817 | 0.966 |
| AT2G42540 | 3.08E-05 | 0.005 | 957 | 0.633 |
| AT1G66200 | 2.93E-05 | 0.005 | 849 | 0.900 |
| AT3G47340 | 2.93E-05 | 0.005 | 978 | 0.680 |
| AT3G06860 | 2.90E-05 | 0.005 | 995 | 0.972 |
| AT2G47240 | 2.90E-05 | 0.005 | 716 | 0.509 |
| AT3G02040 | 2.87E-05 | 0.005 | 821 | 0.555 |
| AT5G46580 | 2.83E-05 | 0.005 | 873 | 0.981 |
| AT3G04550 | 2.76E-05 | 0.005 | 744 | 0.000 |
| AT2G15970 | 2.76E-05 | 0.005 | 734 | 0.427 |
| AT4G26720 | 2.71E-05 | 0.005 | 606 | 0.000 |
| AT2G28800 | 2.58E-05 | 0.005 | 806 | 0.000 |
| AT2G03220 | 2.54E-05 | 0.005 | 919 | 0.716 |
| AT1G79340 | 2.49E-05 | 0.005 | 883 | 0.447 |
| AT1G24470 | 2.46E-05 | 0.005 | 819 | 0.532 |
| AT3G59790 | 2.44E-05 | 0.005 | 808 | 0.000 |
| AT2G15290 | 2.20E-05 | 0.005 | 599 | 0.976 |
| AT1G24180 | 2.20E-05 | 0.005 | 980 | 0.962 |
| AT5G58710 | 2.20E-05 | 0.005 | 836 | 0.995 |
| AT5G51720 | 2.16E-05 | 0.005 | 792 | 0.978 |
| AT1G29660 | 2.08E-05 | 0.005 | 536 | 0.930 |
| AT2G42090 | 2.06E-05 | 0.005 | 629 | 0.940 |
| AT4G10060 | 2.05E-05 | 0.005 | 667 | 0.934 |
| AT1G67840 | 1.94E-05 | 0.005 | 637 | 0.969 |
| AT5G43450 | 1.89E-05 | 0.005 | 718 | 0.633 |
| AT1G22430 | 1.81E-05 | 0.005 | 613 | 0.968 |
| AT1G28220 | 1.80E-05 | 0.005 | 873 | 0.309 |
| AT5G45170 | 1.80E-05 | 0.005 | 743 | 0.975 |
| AT3G23640 | 1.79E-05 | 0.005 | 768 | 0.561 |
| AT1G78060 | 1.75E-05 | 0.005 | 408 | 0.841 |
| AT4G35450 | 1.75E-05 | 0.005 | 981 | 0.704 |
| AT5G12860 | 1.74E-05 | 0.005 | 659 | 0.971 |
| AT4G13670 | 1.64E-05 | 0.005 | 750 | 0.978 |
| AT2G40510 | 1.59E-05 | 0.005 | 947 | 0.000 |
| AT1G58270 | 1.59E-05 | 0.005 | 762 | 0.183 |
| AT3G06100 | 1.50E-05 | 0.005 | 539 | 0.990 |
| AT5G52790 | 1.43E-05 | 0.005 | 466 | 0.885 |
| AT5G50250 | 1.43E-05 | 0.005 | 603 | 0.000 |
| AT5G51890 | 1.39E-05 | 0.005 | 899 | 0.685 |
| AT4G33700 | 1.37E-05 | 0.005 | 353 | 0.877 |
| AT2G03340 | 1.17E-05 | 0.005 | 560 | 0.256 |
| AT1G63260 | 1.16E-05 | 0.005 | 716 | 0.335 |
| AT4G17370 | 1.13E-05 | 0.005 | 822 | 0.598 |
| AT5G25480 | 1.04E-05 | 0.005 | 916 | 0.462 |
| AT3G28490 | 1.02E-05 | 0.005 | 329 | 0.319 |
| AT2G27030 | 4.85E-05 | 0.006 | 901 | 0.875 |
| AT1G53750 | 4.37E-05 | 0.006 | 998 | 0.790 |
| AT5G08650 | 4.34E-05 | 0.006 | 883 | 0.000 |
| AT5G14040 | 3.73E-05 | 0.006 | 829 | 0.945 |
| AT5G55250 | 3.42E-05 | 0.006 | 677 | 0.506 |
| AT4G18370 | 3.35E-05 | 0.006 | 906 | 0.000 |
| AT4G23570 | 3.35E-05 | 0.006 | 974 | 0.590 |
| AT4G39400 | 3.23E-05 | 0.006 | 999 | 0.896 |
| AT3G01910 | 3.23E-05 | 0.006 | 930 | 0.549 |
| AT3G24590 | 3.17E-05 | 0.006 | 837 | 0.000 |
| AT5G65220 | 3.12E-05 | 0.006 | 967 | 0.000 |
| AT1G64820 | 3.10E-05 | 0.006 | 786 | 0.565 |
| AT1G80830 | 2.94E-05 | 0.006 | 534 | 0.569 |
| AT3G03630 | 2.88E-05 | 0.006 | 907 | 0.961 |
| AT2G29290 | 2.88E-05 | 0.006 | 812 | 0.960 |
| AT4G00570 | 2.81E-05 | 0.006 | 972 | 0.857 |
| AT2G02860 | 2.79E-05 | 0.006 | 829 | 0.625 |
| AT4G32840 | 2.76E-05 | 0.006 | 974 | 0.876 |
| AT1G14980 | 2.52E-05 | 0.006 | 971 | 0.990 |
| AT5G56710 | 2.52E-05 | 0.006 | 994 | 0.000 |
| AT5G47435 | 2.28E-05 | 0.006 | 989 | 0.610 |
| AT3G61140 | 2.28E-05 | 0.006 | 830 | 0.624 |
| AT5G17530 | 2.20E-05 | 0.006 | 772 | 0.000 |
| AT4G23100 | 2.17E-05 | 0.006 | 980 | 0.643 |
| AT5G03570 | 2.14E-05 | 0.006 | 371 | 0.745 |
| AT1G04270 | 2.12E-05 | 0.006 | 976 | 0.000 |
| AT1G30510 | 2.03E-05 | 0.006 | 411 | 0.000 |
| AT5G65720 | 1.86E-05 | 0.006 | 745 | 0.642 |
| AT1G11545 | 1.79E-05 | 0.006 | 383 | 0.669 |
| AT1G70580 | 1.76E-05 | 0.006 | 899 | 0.929 |
| AT5G43830 | 1.57E-05 | 0.006 | 593 | 0.382 |
| AT2G47460 | 1.52E-05 | 0.006 | 690 | 0.913 |
| AT5G39660 | 1.47E-05 | 0.006 | 855 | 0.431 |
| AT3G13440 | 1.36E-05 | 0.006 | 969 | 0.000 |
| AT5G20980 | 1.35E-05 | 0.006 | 991 | 0.887 |
| AT4G14240 | 1.28E-05 | 0.006 | 285 | 0.877 |
| AT4G39920 | 1.23E-05 | 0.006 | 424 | 0.248 |
| AT3G49920 | 1.19E-05 | 0.006 | 578 | 0.892 |
| AT4G32208 | 1.07E-05 | 0.006 | 608 | 0.000 |
| AT5G20610 | 1.03E-05 | 0.006 | 595 | 0.326 |
| AT2G43100 | 4.41E-05 | 0.007 | 994 | 0.904 |
| AT1G10970 | 4.21E-05 | 0.007 | 788 | 0.481 |
| AT2G47590 | 4.04E-05 | 0.007 | 848 | 0.000 |
| AT1G64520 | 3.84E-05 | 0.007 | 999 | 0.892 |
| AT1G63710 | 3.78E-05 | 0.007 | 788 | 0.968 |
| AT1G80460 | 3.56E-05 | 0.007 | 899 | 0.846 |
| AT1G01050 | 3.27E-05 | 0.007 | 940 | 0.959 |
| AT3G56310 | 3.14E-05 | 0.007 | 905 | 0.423 |
| AT4G37840 | 3.08E-05 | 0.007 | 966 | 0.997 |
| AT5G52780 | 2.99E-05 | 0.007 | 817 | 0.980 |
| AT2G24370 | 2.93E-05 | 0.007 | 790 | 0.783 |
| AT3G21240 | 2.87E-05 | 0.007 | 969 | 0.000 |
| AT3G24090 | 2.84E-05 | 0.007 | 918 | 0.857 |
| AT5G40760 | 2.68E-05 | 0.007 | 978 | 0.908 |
| AT5G18800 | 2.54E-05 | 0.007 | 930 | 0.968 |
| AT2G29310 | 2.48E-05 | 0.007 | 620 | 0.957 |
| AT1G15950 | 2.21E-05 | 0.007 | 980 | 0.963 |
| AT5G35680 | 2.16E-05 | 0.007 | 725 | 0.000 |
| AT3G57050 | 2.10E-05 | 0.007 | 973 | 0.903 |
| AT4G01370 | 2.04E-05 | 0.007 | 999 | 0.000 |
| AT1G48470 | 1.98E-05 | 0.007 | 959 | 0.897 |
| AT2G25420 | 1.93E-05 | 0.007 | 283 | 0.909 |
| AT2G36460 | 1.92E-05 | 0.007 | 758 | 0.748 |
| AT1G04630 | 1.84E-05 | 0.007 | 924 | 0.752 |
| AT5G13640 | 1.84E-05 | 0.007 | 899 | 0.523 |
| AT1G69870 | 1.73E-05 | 0.007 | 539 | 0.762 |
| AT4G36800 | 1.70E-05 | 0.007 | 855 | 0.381 |
| AT4G40040 | 1.64E-05 | 0.007 | 544 | 0.744 |
| AT5G17520 | 1.64E-05 | 0.007 | 725 | 0.779 |
| AT5G49500 | 1.64E-05 | 0.007 | 971 | 0.981 |
| AT3G15690 | 1.63E-05 | 0.007 | 926 | 0.726 |
| AT1G73660 | 1.62E-05 | 0.007 | 563 | 0.715 |
| AT5G55610 | 1.33E-05 | 0.007 | 716 | 0.719 |
| AT1G47550 | 1.32E-05 | 0.007 | 430 | 0.613 |
| AT1G70770 | 1.20E-05 | 0.007 | 585 | 0.672 |
| AT4G26710 | 1.19E-05 | 0.007 | 899 | 0.704 |
| AT1G64405 | 1.18E-05 | 0.007 | 788 | 0.232 |
| AT1G30560 | 1.16E-05 | 0.007 | 718 | 0.459 |
| AT5G20990 | 5.78E-05 | 0.008 | 977 | 0.703 |
| AT1G62800 | 4.97E-05 | 0.008 | 978 | 0.821 |
| AT2G41680 | 4.94E-05 | 0.008 | 923 | 0.000 |
| AT3G55400 | 4.57E-05 | 0.008 | 904 | 0.972 |
| AT3G63410 | 4.36E-05 | 0.008 | 869 | 0.000 |
| AT3G25690 | 4.12E-05 | 0.008 | 891 | 0.978 |
| AT1G50450 | 4.02E-05 | 0.008 | 734 | 0.974 |
| AT2G23670 | 3.87E-05 | 0.008 | 869 | 0.000 |
| AT1G55260 | 3.53E-05 | 0.008 | 853 | 0.657 |
| AT1G47250 | 3.49E-05 | 0.008 | 998 | 0.867 |
| AT1G19660 | 3.23E-05 | 0.008 | 513 | 0.315 |
| AT1G24510 | 3.07E-05 | 0.008 | 762 | 0.000 |
| AT1G48150 | 3.03E-05 | 0.008 | 774 | 0.963 |
| AT1G69740 | 3.01E-05 | 0.008 | 936 | 0.967 |
| AT1G62380 | 2.92E-05 | 0.008 | 972 | 0.339 |
| AT4G16390 | 2.80E-05 | 0.008 | 774 | 0.961 |
| AT5G04360 | 2.76E-05 | 0.008 | 947 | 0.826 |
| AT5G45950 | 2.62E-05 | 0.008 | 760 | 0.509 |
| AT1G34430 | 2.52E-05 | 0.008 | 994 | 0.972 |
| AT3G14210 | 2.35E-05 | 0.008 | 857 | 0.749 |
| AT5G38980 | 2.33E-05 | 0.008 | 764 | 0.987 |
| AT2G43010 | 2.25E-05 | 0.008 | 905 | 0.749 |
| AT4G00490 | 2.14E-05 | 0.008 | 742 | 0.913 |
| AT4G08390 | 2.10E-05 | 0.008 | 936 | 0.928 |
| AT4G36390 | 1.98E-05 | 0.008 | 663 | 0.981 |
| AT3G53470 | 1.95E-05 | 0.008 | 726 | 0.985 |
| AT2G33830 | 1.92E-05 | 0.008 | 534 | 0.299 |
| AT5G35550 | 1.92E-05 | 0.008 | 966 | 0.934 |
| AT2G30790 | 1.76E-05 | 0.008 | 936 | 0.979 |
| AT4G16250 | 1.74E-05 | 0.008 | 919 | 0.764 |
| AT5G63060 | 1.54E-05 | 0.008 | 603 | 0.951 |
| AT1G68875 | 1.52E-05 | 0.008 | 567 | 0.678 |
| AT5G21100 | 1.49E-05 | 0.008 | 913 | 0.813 |
| AT1G78600 | 1.42E-05 | 0.008 | 406 | 0.614 |
| AT4G23630 | 1.40E-05 | 0.008 | 465 | 0.367 |
| AT2G30980 | 1.38E-05 | 0.008 | 565 | 0.795 |
| AT5G25390 | 1.38E-05 | 0.008 | 802 | 0.781 |
| AT5G51760 | 1.34E-05 | 0.008 | 599 | 0.000 |
| AT1G74910 | 1.07E-05 | 0.008 | 975 | 0.569 |
| AT1G36180 | 4.44E-05 | 0.009 | 998 | 0.000 |
| AT1G44446 | 3.68E-05 | 0.009 | 995 | 0.945 |
| AT5G11110 | 3.29E-05 | 0.009 | 577 | 0.000 |
| AT3G53620 | 3.28E-05 | 0.009 | 940 | 0.942 |
| AT3G51240 | 3.13E-05 | 0.009 | 999 | 0.702 |
| AT3G01790 | 2.91E-05 | 0.009 | 928 | 0.000 |
| AT2G23390 | 2.12E-05 | 0.009 | 726 | 0.974 |
| AT2G19940 | 2.03E-05 | 0.009 | 997 | 0.959 |
| AT1G71220 | 2.01E-05 | 0.009 | 843 | 0.561 |
| AT4G20830 | 1.97E-05 | 0.009 | 560 | 0.711 |
| AT5G60360 | 1.96E-05 | 0.009 | 681 | 0.347 |
| AT2G20360 | 1.94E-05 | 0.009 | 989 | 0.868 |
| AT3G18490 | 1.85E-05 | 0.009 | 681 | 0.781 |
| AT4G00540 | 1.80E-05 | 0.009 | 821 | 0.966 |
| AT4G19810 | 1.63E-05 | 0.009 | 899 | 0.415 |
| AT2G20690 | 1.52E-05 | 0.009 | 899 | 0.941 |
| AT1G06030 | 1.50E-05 | 0.009 | 919 | 0.948 |
| AT5G16980 | 1.04E-05 | 0.009 | 595 | 0.706 |
| AT4G35570 | 4.77E-05 | 0.01 | 657 | 0.918 |
| AT1G25230 | 4.28E-05 | 0.01 | 916 | 0.736 |
| AT1G09570 | 3.93E-05 | 0.01 | 999 | 0.745 |
| AT2G18230 | 3.75E-05 | 0.01 | 940 | 0.947 |
| AT2G17950 | 3.64E-05 | 0.01 | 871 | 0.652 |
| AT2G26910 | 3.57E-05 | 0.01 | 732 | 0.000 |
| AT1G18540 | 3.41E-05 | 0.01 | 997 | 0.000 |
| AT1G20450 | 3.13E-05 | 0.01 | 673 | 0.461 |
| AT3G11270 | 3.02E-05 | 0.01 | 990 | 0.719 |
| AT2G36310 | 2.94E-05 | 0.01 | 677 | 0.352 |
| AT5G41080 | 2.90E-05 | 0.01 | 884 | 0.625 |
| AT5G40950 | 2.80E-05 | 0.01 | 961 | 0.000 |
| AT4G30210 | 2.78E-05 | 0.01 | 612 | 0.877 |
| AT2G18710 | 2.65E-05 | 0.01 | 853 | 0.964 |
| AT3G22630 | 2.63E-05 | 0.01 | 999 | 0.968 |
| AT5G64750 | 2.42E-05 | 0.01 | 659 | 0.519 |
| AT4G25810 | 2.32E-05 | 0.01 | 542 | 0.643 |
| AT5G17490 | 2.28E-05 | 0.01 | 534 | 0.557 |
| AT5G56970 | 2.26E-05 | 0.01 | 572 | 0.482 |
| AT1G27950 | 2.13E-05 | 0.01 | 599 | 0.607 |
| AT3G59060 | 2.12E-05 | 0.01 | 903 | 0.736 |
| AT4G26270 | 2.11E-05 | 0.01 | 974 | 0.867 |
| AT2G38050 | 1.94E-05 | 0.01 | 994 | 0.329 |
| AT3G26744 | 1.80E-05 | 0.01 | 983 | 0.444 |
| AT5G13850 | 1.76E-05 | 0.01 | 948 | 0.000 |
| AT4G30120 | 1.64E-05 | 0.01 | 288 | 0.966 |
| AT4G20760 | 1.48E-05 | 0.01 | 585 | 0.986 |
| AT5G20140 | 1.43E-05 | 0.01 | 776 | 0.974 |
| AT4G17040 | 1.41E-05 | 0.01 | 700 | 0.000 |
| AT4G30310 | 1.35E-05 | 0.01 | 408 | 0.411 |
| AT4G25370 | 1.33E-05 | 0.01 | 605 | 0.000 |
| AT4G25350 | 1.06E-05 | 0.01 | 788 | 0.439 |
| AT1G68590 | 5.22E-05 | 0.011 | 944 | 0.000 |
| AT4G14700 | 4.31E-05 | 0.011 | 998 | 0.780 |
| AT1G26945 | 3.56E-05 | 0.011 | 960 | 0.346 |
| AT5G58290 | 3.53E-05 | 0.011 | 999 | 0.722 |
| AT5G40610 | 3.44E-05 | 0.011 | 899 | 0.927 |
| AT4G24830 | 3.37E-05 | 0.011 | 975 | 0.960 |
| AT5G52440 | 2.84E-05 | 0.011 | 885 | 0.000 |
| AT2G04520 | 2.84E-05 | 0.011 | 769 | 0.000 |
| AT4G36360 | 2.77E-05 | 0.011 | 508 | 0.780 |
| AT4G19650 | 2.72E-05 | 0.011 | 279 | 0.924 |
| AT3G15354 | 2.66E-05 | 0.011 | 906 | 0.873 |
| AT1G04350 | 2.60E-05 | 0.011 | 748 | 0.957 |
| AT1G16540 | 2.49E-05 | 0.011 | 871 | 0.468 |
| AT1G02580 | 2.41E-05 | 0.011 | 946 | 0.830 |
| AT2G18110 | 2.17E-05 | 0.011 | 800 | 0.000 |
| AT4G08770 | 2.11E-05 | 0.011 | 899 | 0.502 |
| AT4G31870 | 1.96E-05 | 0.011 | 916 | 0.766 |
| AT5G15800 | 1.83E-05 | 0.011 | 969 | 0.944 |
| AT2G38960 | 1.78E-05 | 0.011 | 866 | 0.229 |
| AT1G76420 | 1.71E-05 | 0.011 | 408 | 0.495 |
| AT2G14170 | 1.66E-05 | 0.011 | 909 | 0.614 |
| AT4G12310 | 1.66E-05 | 0.011 | 621 | 0.765 |
| AT1G30515 | 1.59E-05 | 0.011 | 411 | 0.000 |
| AT3G27920 | 1.54E-05 | 0.011 | 641 | 0.851 |
| AT1G06110 | 1.38E-05 | 0.011 | 954 | 0.355 |
| AT5G24810 | 1.31E-05 | 0.011 | 314 | 0.000 |
| AT4G09670 | 1.30E-05 | 0.011 | 822 | 0.749 |
| AT3G47860 | 1.27E-05 | 0.011 | 603 | 0.977 |
| AT5G23280 | 1.20E-05 | 0.011 | 407 | 0.419 |
| AT4G29480 | 1.18E-05 | 0.011 | 922 | 0.986 |
| AT5G55290 | 1.17E-05 | 0.011 | 899 | 0.612 |
| AT2G29190 | 1.07E-05 | 0.011 | 542 | 0.943 |
| AT4G39540 | 1.01E-05 | 0.011 | 885 | 0.933 |
| AT3G57800 | 8.39E-05 | 0.012 | 468 | 0.656 |
| AT3G12580 | 4.80E-05 | 0.012 | 935 | 0.000 |
| AT1G68830 | 3.76E-05 | 0.012 | 790 | 0.000 |
| AT4G37200 | 3.16E-05 | 0.012 | 844 | 0.817 |
| AT4G26850 | 3.15E-05 | 0.012 | 659 | 0.718 |
| AT4G17390 | 3.02E-05 | 0.012 | 995 | 0.000 |
| AT3G42850 | 2.84E-05 | 0.012 | 872 | 0.934 |
| AT5G08410 | 2.83E-05 | 0.012 | 896 | 0.985 |
| AT4G12040 | 2.83E-05 | 0.012 | 484 | 0.311 |
| AT4G35230 | 2.76E-05 | 0.012 | 624 | 0.384 |
| AT5G08640 | 2.72E-05 | 0.012 | 999 | 0.712 |
| AT1G50250 | 2.48E-05 | 0.012 | 777 | 0.000 |
| AT4G19170 | 2.45E-05 | 0.012 | 512 | 0.888 |
| AT2G21600 | 2.31E-05 | 0.012 | 813 | 0.389 |
| AT4G21200 | 2.29E-05 | 0.012 | 536 | 0.726 |
| AT1G64440 | 2.09E-05 | 0.012 | 957 | 0.640 |
| AT1G18580 | 1.94E-05 | 0.012 | 899 | 0.428 |
| AT1G29850 | 1.64E-05 | 0.012 | 495 | 0.950 |
| AT4G25900 | 1.62E-05 | 0.012 | 782 | 0.526 |
| AT3G57560 | 1.62E-05 | 0.012 | 937 | 0.972 |
| AT2G24570 | 1.57E-05 | 0.012 | 567 | 0.386 |
| AT3G26520 | 1.54E-05 | 0.012 | 784 | 0.840 |
| AT1G15030 | 1.40E-05 | 0.012 | 368 | 0.250 |
| AT5G18000 | 1.34E-05 | 0.012 | 406 | 0.284 |
| AT3G20210 | 1.33E-05 | 0.012 | 625 | 0.212 |
| AT4G05490 | 1.29E-05 | 0.012 | 788 | 0.597 |
| AT4G02920 | 1.14E-05 | 0.012 | 496 | 0.973 |
| AT3G23700 | 5.37E-05 | 0.013 | 956 | 0.000 |
| AT5G23900 | 5.28E-05 | 0.013 | 997 | 0.000 |
| AT2G44650 | 3.16E-05 | 0.013 | 931 | 0.000 |
| AT1G30620 | 3.12E-05 | 0.013 | 905 | 0.660 |
| AT1G12520 | 3.12E-05 | 0.013 | 815 | 0.456 |
| AT1G23290 | 3.05E-05 | 0.013 | 996 | 0.000 |
| AT5G39210 | 2.85E-05 | 0.013 | 854 | 0.000 |
| AT1G51200 | 2.76E-05 | 0.013 | 457 | 0.405 |
| AT5G65690 | 2.37E-05 | 0.013 | 986 | 0.778 |
| AT4G00180 | 2.35E-05 | 0.013 | 443 | 0.569 |
| AT2G27150 | 2.29E-05 | 0.013 | 970 | 0.826 |
| AT3G16770 | 2.28E-05 | 0.013 | 978 | 0.520 |
| AT3G53420 | 2.16E-05 | 0.013 | 534 | 0.811 |
| AT4G34200 | 2.05E-05 | 0.013 | 517 | 0.901 |
| AT1G63610 | 2.03E-05 | 0.013 | 722 | 0.000 |
| AT5G20960 | 2.00E-05 | 0.013 | 679 | 0.838 |
| ATCG00350 | 1.70E-05 | 0.013 | 938 | 0.944 |
| AT5G17770 | 1.65E-05 | 0.013 | 898 | 0.863 |
| AT4G34215 | 1.61E-05 | 0.013 | 354 | 0.847 |
| AT4G38420 | 1.29E-05 | 0.013 | 679 | 0.952 |
| AT1G09195 | 1.21E-05 | 0.013 | 647 | 1.000 |
| AT1G15750 | 9.99E-05 | 0.014 | 868 | 0.759 |
| AT5G39320 | 4.92E-05 | 0.014 | 971 | 0.660 |
| AT1G01580 | 3.62E-05 | 0.014 | 823 | 0.652 |
| AT3G47470 | 3.56E-05 | 0.014 | 973 | 0.995 |
| AT4G30580 | 3.56E-05 | 0.014 | 936 | 0.931 |
| AT4G09820 | 3.18E-05 | 0.014 | 966 | 0.428 |
| AT1G62640 | 3.09E-05 | 0.014 | 979 | 0.713 |
| AT1G16900 | 3.05E-05 | 0.014 | 790 | 0.520 |
| AT4G16720 | 2.86E-05 | 0.014 | 997 | 0.000 |
| AT4G31500 | 2.63E-05 | 0.014 | 869 | 0.683 |
| AT5G10480 | 2.46E-05 | 0.014 | 565 | 0.475 |
| AT3G01260 | 2.44E-05 | 0.014 | 939 | 0.940 |
| AT4G17520 | 2.35E-05 | 0.014 | 899 | 0.993 |
| AT3G19710 | 2.34E-05 | 0.014 | 534 | 0.846 |
| AT4G21170 | 2.07E-05 | 0.014 | 679 | 0.562 |
| AT5G20240 | 1.99E-05 | 0.014 | 601 | 0.855 |
| AT4G36250 | 1.97E-05 | 0.014 | 810 | 0.871 |
| AT4G39080 | 1.91E-05 | 0.014 | 998 | 0.745 |
| AT4G18350 | 1.85E-05 | 0.014 | 599 | 0.490 |
| AT1G49975 | 1.83E-05 | 0.014 | 688 | 0.984 |
| AT2G22250 | 1.68E-05 | 0.014 | 562 | 0.890 |
| AT1G74810 | 1.66E-05 | 0.014 | 599 | 0.346 |
| AT5G14750 | 1.64E-05 | 0.014 | 677 | 0.894 |
| AT4G09900 | 1.64E-05 | 0.014 | 424 | 0.711 |
| AT5G26800 | 1.56E-05 | 0.014 | 424 | 0.829 |
| AT3G05840 | 1.34E-05 | 0.014 | 567 | 0.776 |
| AT5G51980 | 1.32E-05 | 0.014 | 636 | 0.569 |
| AT1G15290 | 1.16E-05 | 0.014 | 554 | 0.954 |
| AT5G51550 | 1.09E-05 | 0.014 | 679 | 0.328 |
| AT2G47940 | 4.20E-05 | 0.015 | 891 | 0.000 |
| AT5G14660 | 4.18E-05 | 0.015 | 905 | 0.000 |
| AT5G52310 | 4.01E-05 | 0.015 | 958 | 0.524 |
| AT3G07110 | 3.62E-05 | 0.015 | 992 | 0.000 |
| AT5G42650 | 3.24E-05 | 0.015 | 994 | 0.672 |
| AT4G31850 | 2.88E-05 | 0.015 | 712 | 0.968 |
| AT1G60950 | 2.75E-05 | 0.015 | 911 | 0.932 |
| AT1G51760 | 2.73E-05 | 0.015 | 675 | 0.603 |
| AT5G07090 | 2.72E-05 | 0.015 | 990 | 0.000 |
| AT3G01060 | 2.67E-05 | 0.015 | 726 | 0.975 |
| AT4G01800 | 2.66E-05 | 0.015 | 768 | 0.000 |
| AT4G24780 | 2.64E-05 | 0.015 | 899 | 0.493 |
| AT2G38650 | 2.47E-05 | 0.015 | 915 | 0.776 |
| AT1G08540 | 2.44E-05 | 0.015 | 769 | 0.000 |
| AT4G15000 | 2.41E-05 | 0.015 | 995 | 0.000 |
| AT4G24520 | 2.35E-05 | 0.015 | 411 | 0.000 |
| AT2G36160 | 2.15E-05 | 0.015 | 996 | 0.000 |
| AT1G26850 | 2.06E-05 | 0.015 | 501 | 0.802 |
| AT1G08510 | 1.99E-05 | 0.015 | 986 | 0.437 |
| AT1G04810 | 1.79E-05 | 0.015 | 958 | 0.575 |
| AT1G54270 | 1.70E-05 | 0.015 | 771 | 0.986 |
| AT5G53890 | 1.69E-05 | 0.015 | 875 | 0.985 |
| AT1G28300 | 1.64E-05 | 0.015 | 827 | 0.798 |
| AT1G13580 | 1.54E-05 | 0.015 | 575 | 0.382 |
| AT2G02870 | 1.02E-05 | 0.015 | 856 | 0.418 |
| AT5G58140 | 4.02E-05 | 0.016 | 935 | 0.942 |
| AT5G08280 | 3.80E-05 | 0.016 | 988 | 0.982 |
| AT5G15200 | 3.54E-05 | 0.016 | 996 | 0.000 |
| AT2G35500 | 3.17E-05 | 0.016 | 923 | 0.000 |
| AT1G01600 | 3.05E-05 | 0.016 | 765 | 0.977 |
| AT1G73655 | 3.02E-05 | 0.016 | 806 | 0.000 |
| AT3G25805 | 2.89E-05 | 0.016 | 872 | 0.000 |
| AT5G52300 | 2.56E-05 | 0.016 | 767 | 0.488 |
| AT4G01690 | 2.56E-05 | 0.016 | 998 | 0.000 |
| AT3G26220 | 2.54E-05 | 0.016 | 657 | 0.637 |
| AT5G28840 | 2.47E-05 | 0.016 | 951 | 0.960 |
| AT1G51260 | 2.32E-05 | 0.016 | 913 | 0.950 |
| AT5G62140 | 2.16E-05 | 0.016 | 611 | 0.940 |
| AT3G53280 | 2.15E-05 | 0.016 | 655 | 0.946 |
| AT2G25240 | 2.12E-05 | 0.016 | 904 | 0.362 |
| AT5G09600 | 2.08E-05 | 0.016 | 934 | 0.935 |
| AT1G58290 | 2.06E-05 | 0.016 | 816 | 0.963 |
| AT3G13290 | 2.06E-05 | 0.016 | 543 | 0.507 |
| AT4G14440 | 1.74E-05 | 0.016 | 671 | 0.482 |
| AT5G13720 | 1.61E-05 | 0.016 | 633 | 0.968 |
| AT5G61970 | 1.59E-05 | 0.016 | 949 | 0.986 |
| AT1G60980 | 1.59E-05 | 0.016 | 914 | 0.886 |
| AT2G02470 | 1.43E-05 | 0.016 | 424 | 0.357 |
| AT2G17190 | 1.32E-05 | 0.016 | 635 | 0.983 |
| AT3G45310 | 1.24E-05 | 0.016 | 438 | 0.675 |
| AT2G36620 | 3.96E-05 | 0.017 | 985 | 0.000 |
| AT2G21530 | 3.76E-05 | 0.017 | 944 | 0.000 |
| AT5G60600 | 3.67E-05 | 0.017 | 978 | 0.947 |
| AT2G04030 | 3.28E-05 | 0.017 | 917 | 0.950 |
| AT1G80030 | 2.85E-05 | 0.017 | 821 | 0.982 |
| AT2G46340 | 2.85E-05 | 0.017 | 985 | 0.948 |
| AT2G35410 | 2.80E-05 | 0.017 | 802 | 0.982 |
| AT3G61260 | 2.80E-05 | 0.017 | 679 | 0.378 |
| AT1G34130 | 2.50E-05 | 0.017 | 863 | 0.740 |
| AT2G17420 | 2.24E-05 | 0.017 | 720 | 0.935 |
| AT3G47980 | 2.14E-05 | 0.017 | 921 | 0.435 |
| AT5G02560 | 2.12E-05 | 0.017 | 635 | 0.653 |
| AT1G02920 | 2.09E-05 | 0.017 | 939 | 0.982 |
| AT4G26770 | 1.78E-05 | 0.017 | 943 | 0.731 |
| AT3G44010 | 1.73E-05 | 0.017 | 993 | 0.000 |
| AT4G20260 | 1.70E-05 | 0.017 | 510 | 0.418 |
| AT1G20270 | 1.66E-05 | 0.017 | 291 | 0.274 |
| AT1G19140 | 1.63E-05 | 0.017 | 544 | 0.578 |
| AT5G13110 | 1.45E-05 | 0.017 | 967 | 0.898 |
| AT3G15570 | 1.38E-05 | 0.017 | 436 | 0.897 |
| AT4G18990 | 1.32E-05 | 0.017 | 372 | 0.491 |
| AT2G22910 | 1.20E-05 | 0.017 | 939 | 0.896 |
| AT1G16030 | 3.97E-05 | 0.018 | 926 | 0.000 |
| AT3G20770 | 3.42E-05 | 0.018 | 995 | 0.568 |
| AT4G38920 | 3.33E-05 | 0.018 | 980 | 0.986 |
| AT4G34490 | 3.11E-05 | 0.018 | 897 | 0.915 |
| AT4G37040 | 3.09E-05 | 0.018 | 940 | 0.955 |
| AT3G22210 | 3.05E-05 | 0.018 | 881 | 0.987 |
| AT1G56330 | 2.95E-05 | 0.018 | 953 | 0.965 |
| AT1G20440 | 2.70E-05 | 0.018 | 894 | 0.469 |
| AT4G30000 | 2.49E-05 | 0.018 | 360 | 0.849 |
| AT4G39640 | 2.31E-05 | 0.018 | 965 | 0.910 |
| AT5G10470 | 2.27E-05 | 0.018 | 655 | 0.941 |
| AT2G35635 | 2.15E-05 | 0.018 | 534 | 0.999 |
| AT4G32700 | 2.08E-05 | 0.018 | 236 | 0.693 |
| AT4G03150 | 2.04E-05 | 0.018 | 748 | 0.977 |
| AT4G19985 | 1.95E-05 | 0.018 | 710 | 0.000 |
| AT2G18240 | 1.92E-05 | 0.018 | 813 | 0.401 |
| AT5G63180 | 1.74E-05 | 0.018 | 899 | 0.530 |
| AT2G01420 | 1.64E-05 | 0.018 | 544 | 0.404 |
| AT5G42000 | 1.43E-05 | 0.018 | 575 | 0.284 |
| AT2G41310 | 1.32E-05 | 0.018 | 764 | 0.928 |
| AT1G59730 | 1.16E-05 | 0.018 | 629 | 0.000 |
| AT2G27450 | 1.12E-05 | 0.018 | 881 | 0.399 |
| AT2G13360 | 4.83E-05 | 0.019 | 998 | 0.965 |
| AT2G37040 | 4.81E-05 | 0.019 | 998 | 0.439 |
| AT5G01650 | 4.48E-05 | 0.019 | 925 | 0.954 |
| AT2G33450 | 4.13E-05 | 0.019 | 980 | 0.000 |
| AT5G02870 | 3.22E-05 | 0.019 | 992 | 0.000 |
| AT1G42550 | 3.20E-05 | 0.019 | 821 | 0.922 |
| AT4G38160 | 3.17E-05 | 0.019 | 945 | 0.000 |
| AT1G65260 | 2.69E-05 | 0.019 | 833 | 0.000 |
| AT3G12915 | 2.38E-05 | 0.019 | 894 | 0.000 |
| AT4G22570 | 2.30E-05 | 0.019 | 909 | 0.948 |
| AT1G09690 | 2.03E-05 | 0.019 | 992 | 0.000 |
| AT3G60245 | 1.94E-05 | 0.019 | 994 | 0.000 |
| AT3G50520 | 1.91E-05 | 0.019 | 983 | 0.781 |
| AT1G75130 | 1.81E-05 | 0.019 | 407 | 0.982 |
| AT2G26880 | 1.68E-05 | 0.019 | 560 | 0.960 |
| AT1G14030 | 1.57E-05 | 0.019 | 687 | 0.000 |
| AT5G26570 | 1.57E-05 | 0.019 | 755 | 0.818 |
| AT5G37500 | 1.51E-05 | 0.019 | 651 | 0.431 |
| AT2G17470 | 1.44E-05 | 0.019 | 659 | 0.389 |
| AT2G35520 | 1.33E-05 | 0.019 | 784 | 0.805 |
| AT2G33530 | 1.12E-05 | 0.019 | 392 | 0.278 |
| AT3G20580 | 1.11E-05 | 0.019 | 879 | 0.331 |
| AT2G26300 | 4.71E-05 | 0.02 | 944 | 0.910 |
| AT1G72370 | 3.72E-05 | 0.02 | 998 | 0.000 |
| AT4G13700 | 3.25E-05 | 0.02 | 921 | 0.847 |
| AT5G47030 | 3.05E-05 | 0.02 | 999 | 0.989 |
| AT4G03240 | 2.93E-05 | 0.02 | 754 | 0.476 |
| AT5G40770 | 2.61E-05 | 0.02 | 929 | 0.979 |
| AT4G35900 | 2.50E-05 | 0.02 | 534 | 0.519 |
| AT1G72330 | 2.34E-05 | 0.02 | 809 | 0.929 |
| AT2G42130 | 2.08E-05 | 0.02 | 776 | 0.986 |
| AT4G35650 | 2.07E-05 | 0.02 | 978 | 0.915 |
| AT3G51600 | 1.84E-05 | 0.02 | 762 | 0.938 |
| AT5G18930 | 1.79E-05 | 0.02 | 964 | 0.355 |
| AT5G54650 | 1.72E-05 | 0.02 | 655 | 0.468 |
| AT4G32210 | 1.70E-05 | 0.02 | 934 | 0.915 |
| AT1G26550 | 1.40E-05 | 0.02 | 631 | 0.716 |
| AT5G05080 | 1.32E-05 | 0.02 | 308 | 0.557 |
| AT5G61840 | 1.21E-05 | 0.02 | 599 | 0.848 |
| AT3G59380 | 1.19E-05 | 0.02 | 996 | 0.587 |
| AT3G10900 | 1.11E-05 | 0.02 | 925 | 0.380 |
| AT1G78300 | 4.47E-05 | 0.021 | 780 | 0.869 |
| AT2G03750 | 3.75E-05 | 0.021 | 827 | 0.985 |
| AT3G23050 | 3.33E-05 | 0.021 | 908 | 0.635 |
| AT2G31082 | 2.82E-05 | 0.021 | 966 | 0.615 |
| AT2G42010 | 2.09E-05 | 0.021 | 965 | 0.000 |
| AT3G14310 | 2.02E-05 | 0.021 | 812 | 0.503 |
| AT3G58730 | 1.92E-05 | 0.021 | 999 | 0.737 |
| AT3G24503 | 1.87E-05 | 0.021 | 913 | 0.865 |
| AT5G60700 | 1.85E-05 | 0.021 | 915 | 0.477 |
| AT3G29030 | 1.81E-05 | 0.021 | 553 | 0.278 |
| AT1G22410 | 1.77E-05 | 0.021 | 878 | 0.684 |
| AT1G55110 | 1.63E-05 | 0.021 | 788 | 0.998 |
| AT1G69820 | 1.54E-05 | 0.021 | 957 | 0.981 |
| AT2G42590 | 1.46E-05 | 0.021 | 345 | 0.832 |
| AT1G18500 | 1.23E-05 | 0.021 | 913 | 0.920 |
| AT3G06500 | 1.22E-05 | 0.021 | 448 | 0.527 |
| AT1G09540 | 1.17E-05 | 0.021 | 790 | 0.917 |
| AT1G17580 | 1.15E-05 | 0.021 | 756 | 0.877 |
| AT3G60180 | 1.01E-05 | 0.021 | 943 | 0.978 |
| AT3G26570 | 4.45E-05 | 0.022 | 855 | 0.976 |
| AT2G21280 | 4.29E-05 | 0.022 | 903 | 0.000 |
| AT2G31085 | 2.91E-05 | 0.022 | 879 | 0.414 |
| AT5G11060 | 2.56E-05 | 0.022 | 760 | 0.598 |
| AT3G03910 | 2.32E-05 | 0.022 | 965 | 0.972 |
| AT1G52300 | 1.85E-05 | 0.022 | 980 | 0.000 |
| AT2G32230 | 1.83E-05 | 0.022 | 536 | 0.971 |
| AT5G17380 | 1.73E-05 | 0.022 | 889 | 0.907 |
| AT5G59880 | 1.43E-05 | 0.022 | 908 | 0.720 |
| AT1G09790 | 1.15E-05 | 0.022 | 871 | 0.405 |
| AT3G53870 | 3.14E-05 | 0.023 | 984 | 0.000 |
| AT5G41410 | 2.95E-05 | 0.023 | 877 | 0.656 |
| AT3G13450 | 2.68E-05 | 0.023 | 983 | 0.883 |
| AT5G50370 | 2.34E-05 | 0.023 | 940 | 0.982 |
| AT1G17650 | 2.16E-05 | 0.023 | 770 | 0.982 |
| AT5G43930 | 2.03E-05 | 0.023 | 283 | 0.269 |
| AT2G41940 | 1.74E-05 | 0.023 | 475 | 0.576 |
| AT3G44320 | 1.58E-05 | 0.023 | 901 | 0.534 |
| AT5G62110 | 1.44E-05 | 0.023 | 871 | 0.958 |
| AT3G14300 | 1.24E-05 | 0.023 | 534 | 0.752 |
| AT5G53860 | 1.16E-05 | 0.023 | 730 | 0.982 |
| AT4G02700 | 1.15E-05 | 0.023 | 536 | 0.356 |
| AT4G15530 | 4.28E-05 | 0.024 | 978 | 0.784 |
| AT4G24040 | 3.76E-05 | 0.024 | 822 | 0.607 |
| AT3G52770 | 3.42E-05 | 0.024 | 716 | 0.375 |
| AT4G16370 | 3.16E-05 | 0.024 | 669 | 0.621 |
| AT5G17220 | 3.08E-05 | 0.024 | 939 | 0.979 |
| AT5G42070 | 2.99E-05 | 0.024 | 859 | 0.000 |
| AT3G05590 | 2.95E-05 | 0.024 | 994 | 0.000 |
| AT2G17200 | 2.43E-05 | 0.024 | 661 | 0.338 |
| AT5G08200 | 2.27E-05 | 0.024 | 889 | 0.400 |
| AT3G13560 | 2.12E-05 | 0.024 | 608 | 0.585 |
| AT4G17740 | 2.08E-05 | 0.024 | 835 | 0.983 |
| AT3G18040 | 1.86E-05 | 0.024 | 502 | 0.000 |
| AT1G76160 | 1.74E-05 | 0.024 | 635 | 0.867 |
| AT1G80560 | 1.72E-05 | 0.024 | 493 | 0.964 |
| AT1G74550 | 1.57E-05 | 0.024 | 899 | 0.905 |
| AT3G62700 | 1.42E-05 | 0.024 | 560 | 0.000 |
| AT3G18050 | 1.31E-05 | 0.024 | 587 | 0.929 |
| AT1G09240 | 1.21E-05 | 0.024 | 778 | 0.498 |
| AT4G13040 | 1.21E-05 | 0.024 | 407 | 0.492 |
| AT3G62980 | 3.50E-05 | 0.025 | 999 | 0.418 |
| AT2G17720 | 3.49E-05 | 0.025 | 491 | 0.487 |
| AT2G44920 | 3.48E-05 | 0.025 | 816 | 0.983 |
| AT3G19240 | 3.35E-05 | 0.025 | 608 | 0.577 |
| AT5G51010 | 3.02E-05 | 0.025 | 843 | 0.000 |
| AT4G27500 | 2.86E-05 | 0.025 | 871 | 0.202 |
| AT1G50320 | 2.75E-05 | 0.025 | 772 | 0.000 |
| AT5G38510 | 2.10E-05 | 0.025 | 726 | 0.968 |
| AT1G13980 | 2.10E-05 | 0.025 | 649 | 0.500 |
| AT4G38270 | 1.88E-05 | 0.025 | 899 | 0.566 |
| AT3G47500 | 1.83E-05 | 0.025 | 923 | 0.377 |
| AT5G14010 | 1.70E-05 | 0.025 | 534 | 0.542 |
| AT4G12960 | 1.60E-05 | 0.025 | 659 | 0.376 |
| AT4G15700 | 1.56E-05 | 0.025 | 923 | 0.901 |
| AT3G55370 | 1.55E-05 | 0.025 | 659 | 0.696 |
| AT4G21930 | 1.23E-05 | 0.025 | 534 | 0.579 |
| AT4G25280 | 1.01E-05 | 0.025 | 943 | 0.980 |
| AT3G09630 | 3.76E-05 | 0.026 | 993 | 0.000 |
| AT5G51110 | 3.52E-05 | 0.026 | 889 | 0.000 |
| AT1G55020 | 3.52E-05 | 0.026 | 907 | 0.603 |
| AT2G15620 | 2.93E-05 | 0.026 | 989 | 0.732 |
| AT1G62340 | 2.65E-05 | 0.026 | 409 | 0.417 |
| AT5G20230 | 2.55E-05 | 0.026 | 690 | 0.788 |
| AT1G56580 | 2.38E-05 | 0.026 | 548 | 0.723 |
| AT1G09620 | 2.24E-05 | 0.026 | 660 | 0.976 |
| AT3G06483 | 2.20E-05 | 0.026 | 971 | 0.896 |
| AT5G47810 | 2.17E-05 | 0.026 | 974 | 0.886 |
| AT1G75120 | 2.03E-05 | 0.026 | 780 | 0.807 |
| AT1G34030 | 2.00E-05 | 0.026 | 988 | 0.000 |
| AT3G13110 | 1.94E-05 | 0.026 | 998 | 0.869 |
| AT2G31810 | 1.40E-05 | 0.026 | 945 | 0.909 |
| AT4G38580 | 1.38E-05 | 0.026 | 989 | 0.314 |
| AT1G06000 | 1.26E-05 | 0.026 | 730 | 0.792 |
| AT2G42300 | 1.22E-05 | 0.026 | 599 | 0.305 |
| AT4G15260 | 1.21E-05 | 0.026 | 623 | 0.794 |
| AT4G02930 | 4.01E-05 | 0.027 | 855 | 0.000 |
| AT1G60140 | 3.34E-05 | 0.027 | 923 | 0.599 |
| AT5G35360 | 3.23E-05 | 0.027 | 994 | 0.932 |
| AT3G09570 | 3.23E-05 | 0.027 | 679 | 0.299 |
| AT1G22850 | 2.48E-05 | 0.027 | 768 | 0.000 |
| AT3G62870 | 2.43E-05 | 0.027 | 995 | 0.000 |
| AT5G17330 | 2.39E-05 | 0.027 | 946 | 0.000 |
| AT2G47470 | 2.30E-05 | 0.027 | 951 | 0.000 |
| AT4G27230 | 2.03E-05 | 0.027 | 869 | 0.866 |
| AT5G39830 | 1.96E-05 | 0.027 | 759 | 0.000 |
| AT3G09020 | 1.81E-05 | 0.027 | 899 | 0.801 |
| AT1G35340 | 1.78E-05 | 0.027 | 687 | 0.981 |
| AT3G22942 | 1.76E-05 | 0.027 | 657 | 0.544 |
| AT3G63420 | 1.70E-05 | 0.027 | 690 | 0.501 |
| AT3G13550 | 1.68E-05 | 0.027 | 973 | 0.576 |
| AT2G02000 | 1.64E-05 | 0.027 | 909 | 0.000 |
| AT4G26740 | 1.62E-05 | 0.027 | 724 | 0.506 |
| AT1G50730 | 1.49E-05 | 0.027 | 649 | 0.971 |
| AT2G37250 | 1.44E-05 | 0.027 | 911 | 0.981 |
| AT5G63510 | 1.42E-05 | 0.027 | 995 | 0.976 |
| AT3G27020 | 1.34E-05 | 0.027 | 409 | 0.442 |
| AT4G16260 | 1.27E-05 | 0.027 | 621 | 0.446 |
| AT2G29200 | 1.20E-05 | 0.027 | 542 | 0.951 |
| AT3G62660 | 1.14E-05 | 0.027 | 825 | 0.368 |
| AT2G23610 | 1.13E-05 | 0.027 | 563 | 0.605 |
| AT4G33770 | 1.08E-05 | 0.027 | 894 | 0.740 |
| AT5G61410 | 3.92E-05 | 0.028 | 986 | 0.000 |
| AT5G20280 | 3.42E-05 | 0.028 | 930 | 0.000 |
| AT2G04550 | 2.74E-05 | 0.028 | 966 | 0.000 |
| AT3G14240 | 2.63E-05 | 0.028 | 565 | 0.763 |
| AT2G42770 | 2.59E-05 | 0.028 | 659 | 0.000 |
| AT1G28330 | 2.24E-05 | 0.028 | 527 | 0.302 |
| ATCG00020 | 1.92E-05 | 0.028 | 953 | 0.948 |
| AT1G59840 | 1.82E-05 | 0.028 | 812 | 0.000 |
| AT5G07220 | 1.47E-05 | 0.028 | 938 | 0.296 |
| AT2G32920 | 1.41E-05 | 0.028 | 753 | 0.000 |
| AT3G48670 | 1.40E-05 | 0.028 | 679 | 0.399 |
| AT3G12120 | 1.36E-05 | 0.028 | 790 | 0.000 |
| AT3G27890 | 1.22E-05 | 0.028 | 677 | 0.406 |
| AT5G06290 | 5.49E-05 | 0.029 | 949 | 0.000 |
| AT4G33470 | 4.92E-05 | 0.029 | 823 | 0.945 |
| AT3G56940 | 4.83E-05 | 0.029 | 998 | 0.000 |
| AT2G33040 | 4.17E-05 | 0.029 | 998 | 0.986 |
| AT4G17620 | 3.00E-05 | 0.029 | 799 | 0.966 |
| AT4G01610 | 2.93E-05 | 0.029 | 508 | 0.464 |
| AT5G55260 | 2.89E-05 | 0.029 | 606 | 0.000 |
| AT2G42690 | 2.65E-05 | 0.029 | 890 | 0.972 |
| AT1G65960 | 2.52E-05 | 0.029 | 929 | 0.000 |
| AT5G46240 | 2.49E-05 | 0.029 | 869 | 0.336 |
| AT1G25350 | 2.47E-05 | 0.029 | 599 | 0.972 |
| AT1G78050 | 2.12E-05 | 0.029 | 906 | 0.901 |
| AT3G05970 | 2.09E-05 | 0.029 | 817 | 0.644 |
| AT5G04480 | 1.84E-05 | 0.029 | 919 | 0.337 |
| AT1G57770 | 1.75E-05 | 0.029 | 746 | 0.811 |
| AT5G44190 | 1.61E-05 | 0.029 | 619 | 0.904 |
| AT3G43980 | 1.49E-05 | 0.029 | 975 | 0.000 |
| AT5G26160 | 1.26E-05 | 0.029 | 595 | 0.562 |
| AT5G37850 | 1.24E-05 | 0.029 | 983 | 0.428 |
| AT2G35040 | 4.66E-05 | 0.03 | 998 | 0.990 |
| AT3G53260 | 4.51E-05 | 0.03 | 996 | 0.513 |
| AT1G36280 | 3.30E-05 | 0.03 | 998 | 0.761 |
| AT1G15930 | 3.28E-05 | 0.03 | 998 | 0.000 |
| AT1G14810 | 2.88E-05 | 0.03 | 843 | 0.000 |
| AT4G22880 | 2.88E-05 | 0.03 | 991 | 0.578 |
| AT3G29130 | 2.61E-05 | 0.03 | 550 | 0.374 |
| AT2G26340 | 2.48E-05 | 0.03 | 845 | 0.000 |
| AT1G02150 | 2.46E-05 | 0.03 | 781 | 0.000 |
| AT1G19850 | 2.42E-05 | 0.03 | 807 | 0.570 |
| AT5G49480 | 1.99E-05 | 0.03 | 669 | 0.777 |
| AT3G55150 | 1.86E-05 | 0.03 | 539 | 0.823 |
| AT1G71480 | 1.77E-05 | 0.03 | 923 | 0.984 |
| AT5G27670 | 1.74E-05 | 0.03 | 630 | 0.711 |
| AT4G00720 | 1.52E-05 | 0.03 | 659 | 0.785 |
| AT4G11830 | 1.51E-05 | 0.03 | 899 | 0.000 |
| AT1G05140 | 1.49E-05 | 0.03 | 543 | 0.974 |
| AT1G68460 | 1.44E-05 | 0.03 | 682 | 0.958 |
| AT5G49220 | 1.30E-05 | 0.03 | 368 | 0.945 |
| AT1G58430 | 1.14E-05 | 0.03 | 599 | 0.610 |
| AT3G16400 | 1.12E-05 | 0.03 | 623 | 0.414 |
| AT1G07320 | 5.46E-05 | 0.031 | 996 | 0.000 |
| AT3G08580 | 3.69E-05 | 0.031 | 794 | 0.979 |
| AT1G33040 | 3.42E-05 | 0.031 | 924 | 0.000 |
| AT5G24930 | 3.33E-05 | 0.031 | 758 | 0.783 |
| AT1G66530 | 3.21E-05 | 0.031 | 524 | 0.977 |
| AT2G39770 | 3.09E-05 | 0.031 | 991 | 0.595 |
| AT5G54510 | 3.09E-05 | 0.031 | 755 | 0.339 |
| AT1G01430 | 2.48E-05 | 0.031 | 821 | 0.625 |
| AT2G40890 | 2.45E-05 | 0.031 | 978 | 0.876 |
| AT3G53910 | 2.37E-05 | 0.031 | 809 | 0.988 |
| AT5G07990 | 2.35E-05 | 0.031 | 937 | 0.724 |
| AT5G11880 | 2.34E-05 | 0.031 | 681 | 0.914 |
| AT5G52250 | 2.26E-05 | 0.031 | 869 | 0.493 |
| AT2G45740 | 2.20E-05 | 0.031 | 813 | 0.926 |
| AT4G01210 | 1.82E-05 | 0.031 | 919 | 0.369 |
| AT1G53090 | 1.61E-05 | 0.031 | 906 | 0.950 |
| AT5G16910 | 1.60E-05 | 0.031 | 408 | 0.514 |
| AT5G33320 | 1.59E-05 | 0.031 | 533 | 0.699 |
| AT5G26210 | 1.59E-05 | 0.031 | 677 | 0.312 |
| AT2G23600 | 1.52E-05 | 0.031 | 539 | 0.958 |
| AT2G17020 | 1.42E-05 | 0.031 | 875 | 0.534 |
| AT5G42390 | 1.40E-05 | 0.031 | 885 | 0.897 |
| AT3G54810 | 1.33E-05 | 0.031 | 527 | 0.865 |
| AT3G25050 | 1.19E-05 | 0.031 | 288 | 0.703 |
| AT5G54900 | 1.01E-05 | 0.031 | 563 | 0.982 |
| AT3G48730 | 4.17E-05 | 0.032 | 926 | 0.000 |
| AT2G05310 | 3.85E-05 | 0.032 | 911 | 0.000 |
| AT3G04760 | 3.44E-05 | 0.032 | 819 | 0.984 |
| AT3G12390 | 2.94E-05 | 0.032 | 971 | 0.000 |
| AT1G18660 | 2.55E-05 | 0.032 | 624 | 0.652 |
| AT1G22170 | 2.37E-05 | 0.032 | 974 | 0.908 |
| AT3G60370 | 2.29E-05 | 0.032 | 929 | 0.000 |
| AT4G14870 | 2.19E-05 | 0.032 | 837 | 0.000 |
| AT1G31920 | 2.12E-05 | 0.032 | 764 | 0.953 |
| AT5G48670 | 1.89E-05 | 0.032 | 595 | 0.876 |
| AT4G29490 | 1.70E-05 | 0.032 | 821 | 0.000 |
| AT4G20460 | 1.68E-05 | 0.032 | 899 | 0.655 |
| AT4G00120 | 1.55E-05 | 0.032 | 659 | 0.410 |
| AT1G43560 | 5.37E-05 | 0.033 | 860 | 0.833 |
| AT1G14150 | 5.07E-05 | 0.033 | 963 | 0.000 |
| AT1G04190 | 3.73E-05 | 0.033 | 794 | 0.989 |
| AT4G24210 | 3.69E-05 | 0.033 | 996 | 0.583 |
| AT2G05620 | 3.68E-05 | 0.033 | 871 | 0.989 |
| AT5G05170 | 2.86E-05 | 0.033 | 571 | 0.516 |
| AT5G43010 | 2.70E-05 | 0.033 | 998 | 0.836 |
| AT2G19500 | 2.17E-05 | 0.033 | 572 | 0.558 |
| AT2G45180 | 1.83E-05 | 0.033 | 613 | 0.981 |
| AT1G32480 | 1.80E-05 | 0.033 | 978 | 0.923 |
| AT1G70310 | 1.62E-05 | 0.033 | 899 | 0.967 |
| AT2G39270 | 1.57E-05 | 0.033 | 911 | 0.974 |
| AT5G06110 | 1.46E-05 | 0.033 | 510 | 0.971 |
| AT3G46110 | 1.35E-05 | 0.033 | 659 | 0.747 |
| AT2G29060 | 1.14E-05 | 0.033 | 502 | 0.968 |
| AT2G35530 | 1.07E-05 | 0.033 | 980 | 0.577 |
| AT4G10750 | 1.04E-05 | 0.033 | 597 | 0.450 |
| AT5G46110 | 4.61E-05 | 0.034 | 935 | 0.000 |
| AT5G19990 | 4.14E-05 | 0.034 | 999 | 0.497 |
| AT3G18890 | 3.92E-05 | 0.034 | 973 | 0.000 |
| AT3G49470 | 3.71E-05 | 0.034 | 901 | 0.000 |
| AT3G19280 | 3.35E-05 | 0.034 | 885 | 0.492 |
| AT1G15690 | 3.24E-05 | 0.034 | 951 | 0.813 |
| AT3G19290 | 2.47E-05 | 0.034 | 869 | 0.446 |
| AT3G21500 | 2.46E-05 | 0.034 | 991 | 0.777 |
| AT5G01920 | 2.38E-05 | 0.034 | 746 | 0.000 |
| AT1G57720 | 2.35E-05 | 0.034 | 874 | 0.987 |
| AT1G03430 | 2.34E-05 | 0.034 | 964 | 0.716 |
| AT1G67430 | 2.33E-05 | 0.034 | 991 | 0.000 |
| AT1G75080 | 1.82E-05 | 0.034 | 974 | 0.434 |
| AT3G16050 | 1.62E-05 | 0.034 | 988 | 0.698 |
| AT5G17670 | 1.62E-05 | 0.034 | 720 | 0.978 |
| ATCG00280 | 1.37E-05 | 0.034 | 963 | 0.956 |
| AT5G53370 | 1.33E-05 | 0.034 | 871 | 0.728 |
| AT2G39890 | 1.17E-05 | 0.034 | 463 | 0.641 |
| AT4G21640 | 1.03E-05 | 0.034 | 537 | 0.954 |
| AT1G14345 | 5.52E-05 | 0.035 | 955 | 0.000 |
| AT3G01440 | 3.75E-05 | 0.035 | 902 | 0.000 |
| AT5G61170 | 3.66E-05 | 0.035 | 993 | 0.000 |
| AT1G10760 | 2.99E-05 | 0.035 | 907 | 0.664 |
| AT3G28860 | 2.97E-05 | 0.035 | 782 | 0.000 |
| AT4G09020 | 2.83E-05 | 0.035 | 967 | 0.696 |
| AT1G11870 | 2.68E-05 | 0.035 | 643 | 0.959 |
| AT1G63180 | 2.66E-05 | 0.035 | 961 | 0.600 |
| AT2G18290 | 2.45E-05 | 0.035 | 954 | 0.639 |
| AT3G56340 | 2.35E-05 | 0.035 | 991 | 0.000 |
| AT1G21400 | 2.34E-05 | 0.035 | 985 | 0.886 |
| AT4G38900 | 2.03E-05 | 0.035 | 887 | 0.446 |
| AT3G27820 | 1.89E-05 | 0.035 | 929 | 0.980 |
| AT1G79510 | 1.65E-05 | 0.035 | 637 | 0.961 |
| AT5G44100 | 1.44E-05 | 0.035 | 340 | 0.577 |
| AT1G25440 | 1.36E-05 | 0.035 | 634 | 0.898 |
| AT5G57410 | 1.31E-05 | 0.035 | 679 | 0.542 |
| AT5G53390 | 1.27E-05 | 0.035 | 653 | 0.335 |
| AT5G51460 | 1.11E-05 | 0.035 | 296 | 0.628 |
| AT4G08780 | 1.09E-05 | 0.035 | 899 | 0.653 |
| AT3G48000 | 3.40E-05 | 0.036 | 800 | 0.861 |
| AT2G30170 | 2.99E-05 | 0.036 | 825 | 0.000 |
| AT2G31083 | 2.88E-05 | 0.036 | 992 | 0.484 |
| AT1G26230 | 2.84E-05 | 0.036 | 972 | 0.974 |
| AT4G12400 | 2.84E-05 | 0.036 | 666 | 0.499 |
| AT1G09870 | 2.21E-05 | 0.036 | 883 | 0.283 |
| AT1G07920 | 2.17E-05 | 0.036 | 643 | 0.991 |
| AT4G24090 | 2.13E-05 | 0.036 | 722 | 0.000 |
| AT4G37750 | 2.08E-05 | 0.036 | 679 | 0.575 |
| AT1G04550 | 2.05E-05 | 0.036 | 807 | 0.487 |
| AT1G29965 | 2.03E-05 | 0.036 | 962 | 0.000 |
| AT1G74060 | 1.91E-05 | 0.036 | 988 | 0.000 |
| AT5G36120 | 1.75E-05 | 0.036 | 641 | 0.000 |
| AT2G44530 | 1.68E-05 | 0.036 | 922 | 0.951 |
| AT5G28570 | 1.24E-05 | 0.036 | 659 | 0.000 |
| AT5G64400 | 1.19E-05 | 0.036 | 446 | 0.954 |
| AT4G09610 | 1.18E-05 | 0.036 | 538 | 0.370 |
| AT2G28510 | 1.06E-05 | 0.036 | 599 | 0.404 |
| AT1G15350 | 1.00E-05 | 0.036 | 659 | 0.242 |
| AT5G51100 | 6.01E-05 | 0.037 | 974 | 0.973 |
| AT5G56010 | 3.36E-05 | 0.037 | 874 | 0.701 |
| AT4G14330 | 3.31E-05 | 0.037 | 704 | 0.804 |
| AT3G51420 | 3.28E-05 | 0.037 | 877 | 0.984 |
| AT2G24060 | 2.82E-05 | 0.037 | 850 | 0.000 |
| AT5G51750 | 2.80E-05 | 0.037 | 784 | 0.839 |
| AT1G05850 | 2.52E-05 | 0.037 | 565 | 0.699 |
| AT3G55380 | 2.43E-05 | 0.037 | 798 | 0.727 |
| AT1G50840 | 2.41E-05 | 0.037 | 899 | 0.712 |
| AT3G25480 | 1.94E-05 | 0.037 | 824 | 0.000 |
| AT2G41490 | 1.77E-05 | 0.037 | 600 | 0.809 |
| AT2G17972 | 1.70E-05 | 0.037 | 681 | 0.984 |
| AT4G22340 | 1.70E-05 | 0.037 | 974 | 0.718 |
| AT4G33360 | 1.66E-05 | 0.037 | 928 | 0.000 |
| AT4G11100 | 1.58E-05 | 0.037 | 722 | 0.982 |
| AT2G34510 | 1.58E-05 | 0.037 | 548 | 0.930 |
| AT1G79230 | 1.46E-05 | 0.037 | 510 | 0.756 |
| AT4G04890 | 1.24E-05 | 0.037 | 518 | 0.408 |
| AT1G50380 | 1.13E-05 | 0.037 | 544 | 0.500 |
| AT5G14970 | 1.06E-05 | 0.037 | 619 | 0.965 |
| AT2G21190 | 1.00E-05 | 0.037 | 525 | 0.402 |
| AT5G66590 | 9.07E-05 | 0.038 | 278 | 0.263 |
| AT2G27020 | 5.96E-05 | 0.038 | 999 | 0.974 |
| AT1G14920 | 4.02E-05 | 0.038 | 999 | 0.685 |
| AT1G30120 | 3.69E-05 | 0.038 | 998 | 0.951 |
| AT3G54220 | 3.09E-05 | 0.038 | 802 | 0.566 |
| AT1G04250 | 2.78E-05 | 0.038 | 818 | 0.657 |
| AT3G18680 | 2.57E-05 | 0.038 | 992 | 0.000 |
| AT3G48930 | 2.46E-05 | 0.038 | 997 | 0.000 |
| AT3G56240 | 2.40E-05 | 0.038 | 540 | 0.496 |
| AT2G17770 | 2.32E-05 | 0.038 | 575 | 0.535 |
| AT5G05520 | 2.25E-05 | 0.038 | 786 | 0.773 |
| AT3G54090 | 2.17E-05 | 0.038 | 825 | 0.964 |
| AT1G65360 | 1.54E-05 | 0.038 | 920 | 0.908 |
| AT1G23130 | 1.48E-05 | 0.038 | 738 | 0.271 |
| AT1G10470 | 1.34E-05 | 0.038 | 736 | 0.902 |
| AT1G53645 | 1.34E-05 | 0.038 | 621 | 0.948 |
| AT5G66150 | 1.29E-05 | 0.038 | 927 | 0.697 |
| AT4G29840 | 1.23E-05 | 0.038 | 556 | 0.950 |
| AT4G08990 | 1.23E-05 | 0.038 | 899 | 0.511 |
| AT3G59400 | 4.36E-05 | 0.039 | 965 | 0.000 |
| AT1G56500 | 3.90E-05 | 0.039 | 941 | 0.000 |
| AT4G26390 | 2.79E-05 | 0.039 | 968 | 0.926 |
| AT1G63650 | 2.79E-05 | 0.039 | 819 | 0.495 |
| AT3G02580 | 2.68E-05 | 0.039 | 917 | 0.000 |
| AT4G27560 | 2.61E-05 | 0.039 | 488 | 0.467 |
| AT2G43790 | 2.27E-05 | 0.039 | 891 | 0.000 |
| AT4G39220 | 2.20E-05 | 0.039 | 813 | 0.462 |
| AT4G34700 | 2.00E-05 | 0.039 | 976 | 0.968 |
| AT2G18450 | 1.95E-05 | 0.039 | 987 | 0.946 |
| AT1G04120 | 1.85E-05 | 0.039 | 408 | 0.000 |
| AT1G19970 | 1.78E-05 | 0.039 | 548 | 0.547 |
| AT2G48150 | 1.58E-05 | 0.039 | 906 | 0.715 |
| AT5G49810 | 1.58E-05 | 0.039 | 921 | 0.539 |
| AT1G52000 | 1.53E-05 | 0.039 | 726 | 0.408 |
| AT2G21430 | 1.36E-05 | 0.039 | 369 | 0.447 |
| AT4G19530 | 1.25E-05 | 0.039 | 537 | 0.865 |
| AT3G57040 | 1.15E-05 | 0.039 | 567 | 0.914 |
| AT1G02090 | 1.04E-05 | 0.039 | 910 | 0.544 |
| AT2G45470 | 3.33E-05 | 0.04 | 771 | 0.689 |
| AT2G31570 | 2.97E-05 | 0.04 | 944 | 0.821 |
| AT1G49380 | 2.91E-05 | 0.04 | 825 | 0.000 |
| AT2G20370 | 2.87E-05 | 0.04 | 883 | 0.762 |
| AT4G31770 | 2.82E-05 | 0.04 | 285 | 0.299 |
| AT1G62660 | 2.75E-05 | 0.04 | 936 | 0.482 |
| AT2G40190 | 2.74E-05 | 0.04 | 919 | 0.000 |
| AT1G11260 | 2.39E-05 | 0.04 | 715 | 0.670 |
| AT1G52690 | 2.33E-05 | 0.04 | 625 | 0.487 |
| AT5G47780 | 2.21E-05 | 0.04 | 899 | 0.554 |
| AT1G41880 | 2.18E-05 | 0.04 | 992 | 0.000 |
| AT4G34730 | 2.14E-05 | 0.04 | 671 | 0.984 |
| AT2G42530 | 2.03E-05 | 0.04 | 755 | 0.422 |
| AT3G07690 | 1.90E-05 | 0.04 | 536 | 0.961 |
| AT1G06190 | 1.74E-05 | 0.04 | 851 | 0.000 |
| AT5G43940 | 1.72E-05 | 0.04 | 530 | 0.805 |
| AT3G25470 | 1.70E-05 | 0.04 | 532 | 0.971 |
| AT5G13350 | 1.69E-05 | 0.04 | 534 | 0.492 |
| AT2G18600 | 1.50E-05 | 0.04 | 620 | 0.365 |
| AT5G56850 | 1.41E-05 | 0.04 | 645 | 0.972 |
| AT2G32640 | 1.39E-05 | 0.04 | 619 | 0.968 |
| AT4G35760 | 1.37E-05 | 0.04 | 903 | 0.972 |
| AT5G62350 | 1.34E-05 | 0.04 | 427 | 0.245 |
| AT4G10180 | 1.28E-05 | 0.04 | 973 | 0.622 |
| AT3G02290 | 1.24E-05 | 0.04 | 788 | 0.471 |
| AT2G46820 | 3.96E-05 | 0.041 | 945 | 0.000 |
| AT3G48200 | 3.20E-05 | 0.041 | 844 | 0.971 |
| AT1G03310 | 3.14E-05 | 0.041 | 904 | 0.772 |
| AT1G75460 | 3.05E-05 | 0.041 | 821 | 0.984 |
| ATCG00120 | 2.84E-05 | 0.041 | 995 | 0.991 |
| AT3G02530 | 2.65E-05 | 0.041 | 686 | 0.986 |
| AT1G70600 | 2.30E-05 | 0.041 | 966 | 0.000 |
| AT5G09640 | 2.30E-05 | 0.041 | 936 | 0.275 |
| AT5G48170 | 2.22E-05 | 0.041 | 805 | 0.468 |
| ATCG00380 | 2.11E-05 | 0.041 | 976 | 0.000 |
| AT5G08590 | 2.02E-05 | 0.041 | 679 | 0.918 |
| AT4G29700 | 1.99E-05 | 0.041 | 899 | 0.206 |
| AT1G67250 | 1.92E-05 | 0.041 | 975 | 0.984 |
| AT1G15310 | 1.69E-05 | 0.041 | 971 | 0.982 |
| AT5G22510 | 1.59E-05 | 0.041 | 742 | 0.867 |
| AT3G50270 | 1.52E-05 | 0.041 | 621 | 0.981 |
| AT1G80360 | 1.31E-05 | 0.041 | 478 | 0.939 |
| AT5G41000 | 1.18E-05 | 0.041 | 677 | 0.478 |
| AT3G12930 | 3.68E-05 | 0.042 | 885 | 0.000 |
| AT3G14890 | 3.62E-05 | 0.042 | 837 | 0.682 |
| AT1G68480 | 3.13E-05 | 0.042 | 505 | 0.610 |
| AT2G45960 | 3.07E-05 | 0.042 | 562 | 0.951 |
| AT1G12240 | 3.02E-05 | 0.042 | 981 | 0.476 |
| AT3G54900 | 2.84E-05 | 0.042 | 955 | 0.979 |
| AT5G23040 | 2.57E-05 | 0.042 | 525 | 0.950 |
| AT5G15840 | 2.55E-05 | 0.042 | 973 | 0.805 |
| AT2G37260 | 1.94E-05 | 0.042 | 786 | 0.523 |
| AT3G66652 | 1.85E-05 | 0.042 | 540 | 0.439 |
| AT5G54630 | 1.66E-05 | 0.042 | 469 | 0.599 |
| AT2G44870 | 1.46E-05 | 0.042 | 510 | 0.979 |
| AT4G29710 | 1.33E-05 | 0.042 | 899 | 0.202 |
| AT3G60390 | 1.32E-05 | 0.042 | 923 | 0.571 |
| AT3G22830 | 1.26E-05 | 0.042 | 466 | 0.685 |
| AT3G29770 | 1.21E-05 | 0.042 | 502 | 0.814 |
| AT2G20760 | 1.09E-05 | 0.042 | 396 | 0.389 |
| AT1G15980 | 4.77E-05 | 0.043 | 935 | 0.000 |
| AT2G33770 | 2.92E-05 | 0.043 | 554 | 0.460 |
| AT1G22710 | 2.52E-05 | 0.043 | 593 | 0.300 |
| AT4G02195 | 2.52E-05 | 0.043 | 716 | 0.786 |
| AT5G04800 | 2.42E-05 | 0.043 | 995 | 0.000 |
| AT3G04720 | 2.40E-05 | 0.043 | 668 | 0.478 |
| AT1G46408 | 2.39E-05 | 0.043 | 901 | 0.957 |
| AT2G05220 | 2.28E-05 | 0.043 | 975 | 0.000 |
| AT1G70510 | 2.27E-05 | 0.043 | 657 | 0.619 |
| AT4G16830 | 2.27E-05 | 0.043 | 899 | 0.989 |
| AT3G15880 | 2.20E-05 | 0.043 | 440 | 0.877 |
| AT5G46420 | 2.18E-05 | 0.043 | 934 | 0.958 |
| AT2G18800 | 2.18E-05 | 0.043 | 484 | 0.465 |
| AT3G24650 | 2.10E-05 | 0.043 | 960 | 0.736 |
| AT1G65300 | 2.03E-05 | 0.043 | 595 | 0.932 |
| AT4G12830 | 1.99E-05 | 0.043 | 754 | 0.977 |
| AT4G18970 | 1.99E-05 | 0.043 | 679 | 0.544 |
| AT2G18960 | 1.74E-05 | 0.043 | 899 | 0.000 |
| AT3G26740 | 1.61E-05 | 0.043 | 732 | 0.681 |
| AT1G77670 | 1.52E-05 | 0.043 | 478 | 0.899 |
| AT5G52200 | 1.49E-05 | 0.043 | 679 | 0.550 |
| AT4G15330 | 1.45E-05 | 0.043 | 452 | 0.896 |
| AT5G58270 | 1.44E-05 | 0.043 | 534 | 0.530 |
| AT2G15640 | 1.39E-05 | 0.043 | 875 | 0.976 |
| AT1G35580 | 1.30E-05 | 0.043 | 563 | 0.541 |
| AT1G08100 | 1.20E-05 | 0.043 | 819 | 0.000 |
| AT5G10720 | 1.12E-05 | 0.043 | 411 | 0.673 |
| AT5G24410 | 1.09E-05 | 0.043 | 985 | 0.787 |
| AT1G22940 | 3.69E-05 | 0.044 | 999 | 0.862 |
| AT2G43980 | 3.23E-05 | 0.044 | 875 | 0.787 |
| AT5G16760 | 3.05E-05 | 0.044 | 871 | 0.754 |
| AT3G49660 | 3.03E-05 | 0.044 | 302 | 0.886 |
| AT4G36220 | 2.97E-05 | 0.044 | 978 | 0.758 |
| AT3G15352 | 2.66E-05 | 0.044 | 685 | 0.695 |
| AT3G21200 | 2.26E-05 | 0.044 | 815 | 0.000 |
| AT5G63400 | 2.23E-05 | 0.044 | 695 | 0.000 |
| AT4G29220 | 2.06E-05 | 0.044 | 974 | 0.911 |
| AT4G03070 | 1.95E-05 | 0.044 | 500 | 0.659 |
| AT3G27010 | 1.88E-05 | 0.044 | 786 | 0.390 |
| AT1G13700 | 1.85E-05 | 0.044 | 963 | 0.772 |
| AT3G18165 | 1.76E-05 | 0.044 | 938 | 0.816 |
| AT1G13280 | 1.42E-05 | 0.044 | 959 | 0.365 |
| AT2G38150 | 1.41E-05 | 0.044 | 899 | 0.549 |
| AT5G20500 | 1.23E-05 | 0.044 | 802 | 0.942 |
| AT5G60160 | 1.11E-05 | 0.044 | 605 | 0.372 |
| AT2G46910 | 1.08E-05 | 0.044 | 598 | 0.979 |
| AT1G06650 | 1.08E-05 | 0.044 | 305 | 0.735 |
| AT3G26000 | 1.07E-05 | 0.044 | 748 | 0.638 |
| AT1G31540 | 5.43E-05 | 0.045 | 875 | 0.597 |
| AT1G35680 | 5.38E-05 | 0.045 | 990 | 0.000 |
| AT1G31800 | 3.35E-05 | 0.045 | 823 | 0.926 |
| AT1G09750 | 3.32E-05 | 0.045 | 654 | 0.941 |
| AT1G22630 | 3.26E-05 | 0.045 | 839 | 0.000 |
| AT4G08620 | 2.93E-05 | 0.045 | 560 | 0.272 |
| AT3G56910 | 2.93E-05 | 0.045 | 890 | 0.000 |
| AT1G79430 | 2.84E-05 | 0.045 | 923 | 0.385 |
| AT4G04040 | 2.50E-05 | 0.045 | 937 | 0.945 |
| AT3G25760 | 2.48E-05 | 0.045 | 975 | 0.511 |
| AT3G06700 | 2.38E-05 | 0.045 | 995 | 0.000 |
| AT5G13430 | 2.28E-05 | 0.045 | 989 | 0.917 |
| AT1G58360 | 2.20E-05 | 0.045 | 536 | 0.683 |
| AT1G02475 | 2.10E-05 | 0.045 | 764 | 0.969 |
| AT3G62950 | 2.09E-05 | 0.045 | 575 | 0.861 |
| AT2G38120 | 2.08E-05 | 0.045 | 925 | 0.512 |
| AT1G11980 | 1.90E-05 | 0.045 | 701 | 0.984 |
| AT3G03190 | 1.53E-05 | 0.045 | 946 | 0.981 |
| AT3G10410 | 1.51E-05 | 0.045 | 510 | 0.364 |
| AT1G73330 | 1.51E-05 | 0.045 | 597 | 0.226 |
| AT4G11650 | 1.49E-05 | 0.045 | 435 | 0.427 |
| AT1G18210 | 1.35E-05 | 0.045 | 491 | 0.862 |
| AT2G22860 | 1.30E-05 | 0.045 | 873 | 0.589 |
| AT2G45140 | 1.19E-05 | 0.045 | 659 | 0.322 |
| AT1G51100 | 1.04E-05 | 0.045 | 577 | 0.980 |
| AT4G11140 | 1.04E-05 | 0.045 | 681 | 0.507 |
| AT2G26780 | 3.52E-05 | 0.046 | 806 | 0.977 |
| AT5G20290 | 2.89E-05 | 0.046 | 999 | 0.000 |
| AT5G42040 | 2.83E-05 | 0.046 | 997 | 0.625 |
| AT2G37620 | 2.54E-05 | 0.046 | 923 | 0.876 |
| AT5G53300 | 2.34E-05 | 0.046 | 756 | 0.561 |
| AT2G02500 | 2.23E-05 | 0.046 | 999 | 0.967 |
| AT3G52580 | 2.21E-05 | 0.046 | 993 | 0.000 |
| AT1G48270 | 2.20E-05 | 0.046 | 504 | 0.668 |
| AT3G13860 | 2.19E-05 | 0.046 | 912 | 0.993 |
| AT4G14230 | 2.14E-05 | 0.046 | 575 | 0.836 |
| AT3G55560 | 2.13E-05 | 0.046 | 463 | 0.386 |
| AT3G16520 | 1.94E-05 | 0.046 | 746 | 0.960 |
| AT3G02470 | 1.92E-05 | 0.046 | 963 | 0.406 |
| AT1G11970 | 1.57E-05 | 0.046 | 617 | 0.984 |
| AT1G31640 | 1.43E-05 | 0.046 | 786 | 0.941 |
| ATCG00330 | 1.37E-05 | 0.046 | 972 | 0.000 |
| AT5G10300 | 1.27E-05 | 0.046 | 409 | 0.301 |
| AT5G18820 | 1.03E-05 | 0.046 | 919 | 0.984 |
| AT3G01820 | 1.01E-05 | 0.046 | 402 | 0.980 |
| AT4G10960 | 3.29E-05 | 0.047 | 956 | 0.663 |
| AT1G71230 | 3.01E-05 | 0.047 | 900 | 0.684 |
| AT5G36170 | 2.62E-05 | 0.047 | 707 | 0.000 |
| AT3G15220 | 2.51E-05 | 0.047 | 788 | 0.914 |
| AT3G48580 | 2.11E-05 | 0.047 | 348 | 0.487 |
| AT3G52500 | 2.01E-05 | 0.047 | 713 | 0.680 |
| AT4G17090 | 1.93E-05 | 0.047 | 936 | 0.795 |
| AT3G06730 | 1.91E-05 | 0.047 | 665 | 0.786 |
| AT2G38670 | 1.90E-05 | 0.047 | 677 | 0.467 |
| AT5G13390 | 1.51E-05 | 0.047 | 887 | 0.406 |
| AT2G41835 | 1.27E-05 | 0.047 | 645 | 0.732 |
| AT5G53290 | 1.12E-05 | 0.047 | 688 | 0.551 |
| AT2G30570 | 3.32E-05 | 0.048 | 953 | 0.000 |
| AT3G11430 | 3.05E-05 | 0.048 | 936 | 0.564 |
| AT3G61430 | 2.76E-05 | 0.048 | 522 | 0.940 |
| AT3G06300 | 2.08E-05 | 0.048 | 408 | 0.272 |
| AT1G08360 | 1.97E-05 | 0.048 | 987 | 0.000 |
| AT2G34430 | 1.86E-05 | 0.048 | 954 | 0.987 |
| AT3G54420 | 1.79E-05 | 0.048 | 899 | 0.463 |
| AT4G18740 | 1.78E-05 | 0.048 | 681 | 0.984 |
| AT5G57720 | 1.60E-05 | 0.048 | 871 | 0.379 |
| AT2G37020 | 1.51E-05 | 0.048 | 792 | 0.955 |
| AT1G64400 | 1.49E-05 | 0.048 | 502 | 0.508 |
| AT5G26110 | 1.49E-05 | 0.048 | 476 | 0.774 |
| AT5G16540 | 1.46E-05 | 0.048 | 539 | 0.890 |
| AT1G06640 | 1.23E-05 | 0.048 | 408 | 0.781 |
| AT2G43530 | 1.07E-05 | 0.048 | 647 | 0.611 |
| AT1G32340 | 1.04E-05 | 0.048 | 885 | 0.190 |
| AT3G11250 | 3.07E-05 | 0.049 | 988 | 0.000 |
| AT3G08010 | 3.00E-05 | 0.049 | 860 | 0.000 |
| AT2G26930 | 2.92E-05 | 0.049 | 940 | 0.000 |
| AT5G61790 | 2.79E-05 | 0.049 | 993 | 0.723 |
| AT2G39930 | 2.69E-05 | 0.049 | 909 | 0.757 |
| AT5G58420 | 2.55E-05 | 0.049 | 993 | 0.000 |
| AT1G12840 | 2.33E-05 | 0.049 | 998 | 0.676 |
| AT5G09510 | 2.03E-05 | 0.049 | 992 | 0.000 |
| AT3G13340 | 2.02E-05 | 0.049 | 283 | 0.213 |
| AT5G11160 | 1.96E-05 | 0.049 | 944 | 0.937 |
| AT5G38450 | 1.94E-05 | 0.049 | 407 | 0.964 |
| AT5G19530 | 1.86E-05 | 0.049 | 905 | 0.915 |
| AT3G10230 | 1.82E-05 | 0.049 | 757 | 0.934 |
| AT3G58790 | 1.79E-05 | 0.049 | 899 | 0.519 |
| AT2G26320 | 1.66E-05 | 0.049 | 659 | 0.963 |
| AT5G19180 | 1.42E-05 | 0.049 | 880 | 0.792 |
| AT5G14200 | 1.39E-05 | 0.049 | 493 | 0.941 |
| AT2G40670 | 1.37E-05 | 0.049 | 736 | 0.904 |
| AT5G19855 | 1.19E-05 | 0.049 | 887 | 0.925 |
| AT5G28640 | 3.02E-05 | 0.05 | - | - |
| AT5G03290 | 2.89E-05 | 0.05 | - | - |
| AT5G58670 | 2.86E-05 | 0.05 | - | - |
| AT1G43850 | 2.61E-05 | 0.05 | - | - |
| AT1G65290 | 2.52E-05 | 0.05 | - | - |
| AT3G02590 | 2.40E-05 | 0.05 | - | - |
| AT3G15450 | 2.21E-05 | 0.05 | - | - |
| AT3G22370 | 2.20E-05 | 0.05 | - | - |
| AT1G24260 | 2.15E-05 | 0.05 | - | - |
| AT1G62170 | 2.12E-05 | 0.05 | - | - |
| AT4G27140 | 2.07E-05 | 0.05 | - | - |
| AT4G18710 | 1.93E-05 | 0.05 | - | - |
| AT5G35840 | 1.68E-05 | 0.05 | - | - |
| AT5G60950 | 1.54E-05 | 0.05 | - | - |
| AT5G63530 | 1.46E-05 | 0.05 | - | - |
| AT3G49680 | 1.43E-05 | 0.05 | - | - |
| AT1G02300 | 1.15E-05 | 0.05 | - | - |
| AT1G02050 | 1.11E-05 | 0.05 | - | - |
| AT1G27480 | 3.80E-05 | 0.051 | - | - |
| AT3G17930 | 3.26E-05 | 0.051 | - | - |
| AT1G52870 | 2.99E-05 | 0.051 | - | - |
| AT3G51260 | 2.67E-05 | 0.051 | - | - |
| AT1G28230 | 2.62E-05 | 0.051 | - | - |
| AT5G51545 | 2.55E-05 | 0.051 | - | - |
| AT3G04940 | 2.40E-05 | 0.051 | - | - |
| AT1G72280 | 2.22E-05 | 0.051 | - | - |
| AT2G23310 | 2.04E-05 | 0.051 | - | - |
| AT2G16510 | 1.92E-05 | 0.051 | - | - |
| AT3G56090 | 1.61E-05 | 0.051 | - | - |
| AT5G23020 | 1.48E-05 | 0.051 | - | - |
| AT1G80470 | 1.43E-05 | 0.051 | - | - |
| AT2G16900 | 1.23E-05 | 0.051 | - | - |
| AT4G36210 | 1.18E-05 | 0.051 | - | - |
| AT5G66970 | 1.05E-05 | 0.051 | - | - |
| AT4G10480 | 3.74E-05 | 0.052 | - | - |
| AT3G09810 | 3.53E-05 | 0.052 | - | - |
| AT3G49670 | 3.05E-05 | 0.052 | - | - |
| AT5G60670 | 2.94E-05 | 0.052 | - | - |
| AT2G38940 | 2.79E-05 | 0.052 | - | - |
| AT5G20040 | 2.58E-05 | 0.052 | - | - |
| AT3G05020 | 2.48E-05 | 0.052 | - | - |
| AT1G63460 | 2.46E-05 | 0.052 | - | - |
| AT1G61630 | 2.27E-05 | 0.052 | - | - |
| AT5G13520 | 2.17E-05 | 0.052 | - | - |
| AT1G56650 | 2.15E-05 | 0.052 | - | - |
| AT3G53890 | 2.09E-05 | 0.052 | - | - |
| AT3G57230 | 2.07E-05 | 0.052 | - | - |
| AT1G09530 | 2.01E-05 | 0.052 | - | - |
| AT2G14880 | 1.95E-05 | 0.052 | - | - |
| AT5G37600 | 1.86E-05 | 0.052 | - | - |
| AT2G32500 | 1.78E-05 | 0.052 | - | - |
| AT2G36000 | 1.70E-05 | 0.052 | - | - |
| AT2G47190 | 1.68E-05 | 0.052 | - | - |
| AT2G43080 | 1.66E-05 | 0.052 | - | - |
| AT5G64840 | 1.64E-05 | 0.052 | - | - |
| AT3G02110 | 1.55E-05 | 0.052 | - | - |
| AT5G53200 | 1.33E-05 | 0.052 | - | - |
| AT3G63230 | 1.30E-05 | 0.052 | - | - |
| AT3G23920 | 1.28E-05 | 0.052 | - | - |
| AT5G59570 | 1.25E-05 | 0.052 | - | - |
| AT4G01070 | 1.02E-05 | 0.052 | - | - |
| AT1G66670 | 1.01E-05 | 0.052 | - | - |
| AT1G50370 | 3.95E-05 | 0.053 | - | - |
| AT5G03860 | 3.65E-05 | 0.053 | - | - |
| AT4G02500 | 2.84E-05 | 0.053 | - | - |
| AT5G42800 | 2.74E-05 | 0.053 | - | - |
| AT4G31240 | 2.04E-05 | 0.053 | - | - |
| AT2G02180 | 1.78E-05 | 0.053 | - | - |
| AT4G17340 | 1.52E-05 | 0.053 | - | - |
| AT5G60730 | 1.50E-05 | 0.053 | - | - |
| AT2G37130 | 1.47E-05 | 0.053 | - | - |
| AT3G61770 | 1.44E-05 | 0.053 | - | - |
| AT5G62430 | 1.44E-05 | 0.053 | - | - |
| AT2G02230 | 1.34E-05 | 0.053 | - | - |
| AT1G29330 | 1.24E-05 | 0.053 | - | - |
| AT4G21090 | 1.21E-05 | 0.053 | - | - |
| AT1G04860 | 1.21E-05 | 0.053 | - | - |
| AT3G49950 | 1.18E-05 | 0.053 | - | - |
| AT5G03120 | 1.10E-05 | 0.053 | - | - |
| AT1G18270 | 4.26E-05 | 0.054 | - | - |
| AT5G27380 | 2.43E-05 | 0.054 | - | - |
| AT5G09310 | 2.22E-05 | 0.054 | - | - |
| AT1G33850 | 1.53E-05 | 0.054 | - | - |
| AT2G41950 | 1.39E-05 | 0.054 | - | - |
| AT4G32400 | 1.10E-05 | 0.054 | - | - |
| AT1G75900 | 1.09E-05 | 0.054 | - | - |
| AT4G34230 | 4.18E-05 | 0.055 | - | - |
| AT2G18740 | 3.72E-05 | 0.055 | - | - |
| AT4G18440 | 3.69E-05 | 0.055 | - | - |
| AT4G27260 | 3.26E-05 | 0.055 | - | - |
| AT5G26710 | 2.07E-05 | 0.055 | - | - |
| AT2G02930 | 1.91E-05 | 0.055 | - | - |
| AT1G49750 | 1.88E-05 | 0.055 | - | - |
| AT1G49710 | 1.84E-05 | 0.055 | - | - |
| AT1G74020 | 1.79E-05 | 0.055 | - | - |
| AT2G41480 | 1.74E-05 | 0.055 | - | - |
| AT3G59140 | 1.70E-05 | 0.055 | - | - |
| AT5G16510 | 1.47E-05 | 0.055 | - | - |
| AT3G14720 | 1.35E-05 | 0.055 | - | - |
| AT1G01620 | 1.09E-05 | 0.055 | - | - |
| AT5G03555 | 4.90E-05 | 0.056 | - | - |
| AT2G26230 | 4.65E-05 | 0.056 | - | - |
| AT5G51940 | 4.58E-05 | 0.056 | - | - |
| AT1G12780 | 3.64E-05 | 0.056 | - | - |
| AT1G73965 | 3.21E-05 | 0.056 | - | - |
| AT5G60390 | 3.12E-05 | 0.056 | - | - |
| AT3G62410 | 2.83E-05 | 0.056 | - | - |
| AT5G57220 | 2.81E-05 | 0.056 | - | - |
| AT3G27925 | 2.78E-05 | 0.056 | - | - |
| AT1G63800 | 2.39E-05 | 0.056 | - | - |
| AT4G02580 | 2.16E-05 | 0.056 | - | - |
| AT1G19300 | 1.97E-05 | 0.056 | - | - |
| AT5G38480 | 1.88E-05 | 0.056 | - | - |
| AT3G13070 | 1.87E-05 | 0.056 | - | - |
| AT4G27030 | 1.84E-05 | 0.056 | - | - |
| AT5G01930 | 1.73E-05 | 0.056 | - | - |
| AT1G69640 | 1.70E-05 | 0.056 | - | - |
| AT3G54650 | 1.62E-05 | 0.056 | - | - |
| AT1G53790 | 1.50E-05 | 0.056 | - | - |
| AT5G54110 | 1.46E-05 | 0.056 | - | - |
| AT1G20050 | 1.45E-05 | 0.056 | - | - |
| AT5G16715 | 1.45E-05 | 0.056 | - | - |
| AT3G05350 | 1.31E-05 | 0.056 | - | - |
| AT5G02940 | 1.28E-05 | 0.056 | - | - |
| AT1G02065 | 1.23E-05 | 0.056 | - | - |
| AT2G14620 | 1.23E-05 | 0.056 | - | - |
| AT5G59320 | 1.19E-05 | 0.056 | - | - |
| AT1G49005 | 3.16E-05 | 0.057 | - | - |
| AT5G41315 | 2.74E-05 | 0.057 | - | - |
| AT5G12110 | 2.56E-05 | 0.057 | - | - |
| AT3G53020 | 2.53E-05 | 0.057 | - | - |
| AT5G15630 | 2.46E-05 | 0.057 | - | - |
| AT1G56430 | 2.07E-05 | 0.057 | - | - |
| AT1G13270 | 1.87E-05 | 0.057 | - | - |
| AT4G02230 | 1.83E-05 | 0.057 | - | - |
| AT4G35000 | 1.72E-05 | 0.057 | - | - |
| AT4G28880 | 1.52E-05 | 0.057 | - | - |
| AT1G16980 | 1.37E-05 | 0.057 | - | - |
| AT4G38790 | 1.13E-05 | 0.057 | - | - |
| AT3G16560 | 1.06E-05 | 0.057 | - | - |
| AT3G62360 | 3.91E-05 | 0.058 | - | - |
| AT3G06850 | 3.19E-05 | 0.058 | - | - |
| AT2G33210 | 2.74E-05 | 0.058 | - | - |
| AT5G09220 | 2.42E-05 | 0.058 | - | - |
| AT1G08630 | 2.14E-05 | 0.058 | - | - |
| AT3G47430 | 2.12E-05 | 0.058 | - | - |
| AT4G15440 | 1.74E-05 | 0.058 | - | - |
| AT3G25540 | 1.66E-05 | 0.058 | - | - |
| AT4G11840 | 1.52E-05 | 0.058 | - | - |
| AT1G58983 | 1.46E-05 | 0.058 | - | - |
| AT1G78370 | 1.38E-05 | 0.058 | - | - |
| AT5G20480 | 1.21E-05 | 0.058 | - | - |
| AT4G35810 | 1.14E-05 | 0.058 | - | - |
| AT1G32100 | 3.82E-05 | 0.059 | - | - |
| AT4G11175 | 2.64E-05 | 0.059 | - | - |
| AT1G01320 | 2.28E-05 | 0.059 | - | - |
| AT4G36750 | 2.11E-05 | 0.059 | - | - |
| AT3G54960 | 2.06E-05 | 0.059 | - | - |
| AT1G44318 | 1.94E-05 | 0.059 | - | - |
| AT1G29470 | 1.87E-05 | 0.059 | - | - |
| AT3G03530 | 1.86E-05 | 0.059 | - | - |
| AT3G22600 | 1.78E-05 | 0.059 | - | - |
| AT2G34960 | 1.78E-05 | 0.059 | - | - |
| AT2G44210 | 1.70E-05 | 0.059 | - | - |
| ATCG01060 | 1.11E-05 | 0.059 | - | - |
| AT3G17470 | 1.05E-05 | 0.059 | - | - |
| AT5G17690 | 4.17E-05 | 0.06 | - | - |
| AT1G48130 | 3.88E-05 | 0.06 | - | - |
| AT1G15730 | 2.73E-05 | 0.06 | - | - |
| AT4G02890 | 2.66E-05 | 0.06 | - | - |
| AT4G27090 | 2.59E-05 | 0.06 | - | - |
| AT3G28040 | 2.45E-05 | 0.06 | - | - |
| AT4G31550 | 2.08E-05 | 0.06 | - | - |
| AT2G44970 | 1.86E-05 | 0.06 | - | - |
| AT3G25900 | 1.34E-05 | 0.06 | - | - |
| AT5G49650 | 1.13E-05 | 0.06 | - | - |
| AT1G44000 | 3.87E-05 | 0.061 | - | - |
| AT5G52640 | 2.85E-05 | 0.061 | - | - |
| AT1G73260 | 1.80E-05 | 0.061 | - | - |
| AT5G18900 | 1.21E-05 | 0.061 | - | - |
| AT5G15770 | 1.19E-05 | 0.061 | - | - |
| AT5G66400 | 2.49E-05 | 0.062 | - | - |
| AT2G28190 | 2.06E-05 | 0.062 | - | - |
| AT1G32500 | 2.00E-05 | 0.062 | - | - |
| ATCG00480 | 1.85E-05 | 0.062 | - | - |
| AT1G45130 | 1.63E-05 | 0.062 | - | - |
| AT5G47570 | 1.57E-05 | 0.062 | - | - |
| AT1G77990 | 1.17E-05 | 0.062 | - | - |
| AT4G33510 | 1.09E-05 | 0.062 | - | - |
| AT4G23050 | 1.08E-05 | 0.062 | - | - |
| AT3G20740 | 2.64E-05 | 0.063 | - | - |
| AT5G42890 | 2.23E-05 | 0.063 | - | - |
| AT3G23240 | 1.99E-05 | 0.063 | - | - |
| AT5G09900 | 1.93E-05 | 0.063 | - | - |
| AT3G07320 | 1.93E-05 | 0.063 | - | - |
| AT5G02380 | 1.86E-05 | 0.063 | - | - |
| AT1G55920 | 1.70E-05 | 0.063 | - | - |
| AT1G71130 | 1.60E-05 | 0.063 | - | - |
| AT1G71180 | 1.39E-05 | 0.063 | - | - |
| AT3G28930 | 1.29E-05 | 0.063 | - | - |
| AT3G12410 | 1.26E-05 | 0.063 | - | - |
| AT3G56680 | 1.16E-05 | 0.063 | - | - |
| AT3G15250 | 1.09E-05 | 0.063 | - | - |
| AT2G23380 | 3.61E-05 | 0.064 | - | - |
| AT5G49720 | 3.46E-05 | 0.064 | - | - |
| AT1G35420 | 3.07E-05 | 0.064 | - | - |
| AT1G20810 | 2.58E-05 | 0.064 | - | - |
| AT1G04240 | 2.43E-05 | 0.064 | - | - |
| AT2G15050 | 2.29E-05 | 0.064 | - | - |
| AT4G36730 | 1.83E-05 | 0.064 | - | - |
| AT5G10450 | 1.78E-05 | 0.064 | - | - |
| AT4G00850 | 1.73E-05 | 0.064 | - | - |
| AT3G12440 | 1.27E-05 | 0.064 | - | - |
| AT3G02690 | 1.22E-05 | 0.064 | - | - |
| AT1G29950 | 3.58E-05 | 0.065 | - | - |
| AT2G47910 | 3.29E-05 | 0.065 | - | - |
| AT5G44030 | 2.57E-05 | 0.065 | - | - |
| AT5G59300 | 2.46E-05 | 0.065 | - | - |
| AT1G19350 | 2.32E-05 | 0.065 | - | - |
| AT4G11850 | 2.03E-05 | 0.065 | - | - |
| AT4G33090 | 1.89E-05 | 0.065 | - | - |
| AT4G18010 | 1.74E-05 | 0.065 | - | - |
| AT3G44490 | 1.60E-05 | 0.065 | - | - |
| AT1G71170 | 1.39E-05 | 0.065 | - | - |
| AT1G69270 | 1.25E-05 | 0.065 | - | - |
| AT5G14120 | 1.12E-05 | 0.065 | - | - |
| AT4G19760 | 1.01E-05 | 0.065 | - | - |
| AT1G65060 | 2.42E-05 | 0.066 | - | - |
| AT1G16300 | 2.28E-05 | 0.066 | - | - |
| AT1G54820 | 2.26E-05 | 0.066 | - | - |
| AT5G52960 | 2.12E-05 | 0.066 | - | - |
| AT2G04660 | 2.05E-05 | 0.066 | - | - |
| AT5G24490 | 1.85E-05 | 0.066 | - | - |
| AT2G29980 | 1.82E-05 | 0.066 | - | - |
| AT4G30190 | 1.42E-05 | 0.066 | - | - |
| AT4G26860 | 1.41E-05 | 0.066 | - | - |
| AT4G20150 | 1.15E-05 | 0.066 | - | - |
| AT5G55320 | 1.13E-05 | 0.066 | - | - |
| AT5G20935 | 2.68E-05 | 0.067 | - | - |
| AT1G09640 | 2.55E-05 | 0.067 | - | - |
| AT4G29210 | 1.91E-05 | 0.067 | - | - |
| AT4G37790 | 1.84E-05 | 0.067 | - | - |
| AT2G17033 | 1.66E-05 | 0.067 | - | - |
| AT4G35100 | 1.46E-05 | 0.067 | - | - |
| AT5G42990 | 1.29E-05 | 0.067 | - | - |
| AT4G10430 | 1.27E-05 | 0.067 | - | - |
| AT3G62800 | 1.07E-05 | 0.067 | - | - |
| AT1G03090 | 3.03E-05 | 0.068 | - | - |
| AT5G15850 | 2.74E-05 | 0.068 | - | - |
| AT5G44120 | 2.15E-05 | 0.068 | - | - |
| AT2G35120 | 2.05E-05 | 0.068 | - | - |
| AT1G31420 | 1.70E-05 | 0.068 | - | - |
| AT5G04430 | 1.64E-05 | 0.068 | - | - |
| AT2G43420 | 1.45E-05 | 0.068 | - | - |
| AT2G41560 | 1.41E-05 | 0.068 | - | - |
| AT5G62530 | 1.31E-05 | 0.068 | - | - |
| AT1G65090 | 1.07E-05 | 0.068 | - | - |
| AT3G06650 | 3.95E-05 | 0.069 | - | - |
| AT3G47450 | 3.30E-05 | 0.069 | - | - |
| AT3G23940 | 2.57E-05 | 0.069 | - | - |
| AT1G05470 | 2.39E-05 | 0.069 | - | - |
| AT2G29490 | 2.34E-05 | 0.069 | - | - |
| AT2G22330 | 1.65E-05 | 0.069 | - | - |
| AT5G52560 | 1.63E-05 | 0.069 | - | - |
| AT2G17820 | 1.60E-05 | 0.069 | - | - |
| AT1G14330 | 1.59E-05 | 0.069 | - | - |
| AT2G09990 | 1.40E-05 | 0.069 | - | - |
| AT3G54360 | 1.32E-05 | 0.069 | - | - |
| AT3G47560 | 1.17E-05 | 0.069 | - | - |
| AT3G44990 | 1.09E-05 | 0.069 | - | - |
| AT3G44460 | 1.08E-05 | 0.069 | - | - |
| AT1G16820 | 3.27E-05 | 0.07 | - | - |
| AT3G14390 | 3.23E-05 | 0.07 | - | - |
| AT3G56880 | 2.55E-05 | 0.07 | - | - |
| AT3G53920 | 2.29E-05 | 0.07 | - | - |
| AT3G04730 | 2.12E-05 | 0.07 | - | - |
| AT5G04120 | 1.97E-05 | 0.07 | - | - |
| AT4G11120 | 1.93E-05 | 0.07 | - | - |
| AT3G10570 | 1.87E-05 | 0.07 | - | - |
| AT4G19050 | 1.77E-05 | 0.07 | - | - |
| AT4G29080 | 1.64E-05 | 0.07 | - | - |
| AT5G15210 | 1.54E-05 | 0.07 | - | - |
| AT5G24850 | 1.47E-05 | 0.07 | - | - |
| AT1G47210 | 1.08E-05 | 0.07 | - | - |
| AT5G03760 | 3.74E-05 | 0.071 | - | - |
| AT5G27030 | 3.72E-05 | 0.071 | - | - |
| AT3G30775 | 3.01E-05 | 0.071 | - | - |
| AT1G69970 | 2.95E-05 | 0.071 | - | - |
| AT3G23400 | 2.53E-05 | 0.071 | - | - |
| AT5G58220 | 2.01E-05 | 0.071 | - | - |
| AT5G18470 | 1.81E-05 | 0.071 | - | - |
| AT5G05200 | 1.62E-05 | 0.071 | - | - |
| AT1G01220 | 1.55E-05 | 0.071 | - | - |
| AT1G24520 | 1.51E-05 | 0.071 | - | - |
| AT5G64290 | 1.44E-05 | 0.071 | - | - |
| AT4G38360 | 1.34E-05 | 0.071 | - | - |
| AT4G02740 | 1.29E-05 | 0.071 | - | - |
| AT4G36410 | 1.23E-05 | 0.071 | - | - |
| AT3G43540 | 1.19E-05 | 0.071 | - | - |
| AT1G70370 | 2.88E-05 | 0.072 | - | - |
| AT1G10210 | 2.74E-05 | 0.072 | - | - |
| AT5G18070 | 2.62E-05 | 0.072 | - | - |
| AT1G58080 | 2.42E-05 | 0.072 | - | - |
| AT5G06690 | 2.18E-05 | 0.072 | - | - |
| AT2G27730 | 1.71E-05 | 0.072 | - | - |
| AT1G49570 | 1.61E-05 | 0.072 | - | - |
| ATCG00270 | 1.54E-05 | 0.072 | - | - |
| AT1G31070 | 1.11E-05 | 0.072 | - | - |
| AT5G45710 | 1.05E-05 | 0.072 | - | - |
| AT5G11260 | 3.33E-05 | 0.073 | - | - |
| AT5G19510 | 3.17E-05 | 0.073 | - | - |
| AT3G10840 | 2.89E-05 | 0.073 | - | - |
| AT1G09795 | 2.73E-05 | 0.073 | - | - |
| AT3G20540 | 2.30E-05 | 0.073 | - | - |
| AT3G04520 | 2.18E-05 | 0.073 | - | - |
| AT3G05810 | 2.07E-05 | 0.073 | - | - |
| AT2G42910 | 1.99E-05 | 0.073 | - | - |
| ATCG01120 | 1.91E-05 | 0.073 | - | - |
| AT2G04240 | 1.64E-05 | 0.073 | - | - |
| AT1G78690 | 1.54E-05 | 0.073 | - | - |
| AT1G20380 | 1.53E-05 | 0.073 | - | - |
| AT4G05160 | 1.21E-05 | 0.073 | - | - |
| AT5G37510 | 3.60E-05 | 0.074 | - | - |
| AT1G02400 | 2.54E-05 | 0.074 | - | - |
| AT3G23390 | 2.21E-05 | 0.074 | - | - |
| AT4G38840 | 2.06E-05 | 0.074 | - | - |
| AT4G25130 | 1.92E-05 | 0.074 | - | - |
| AT4G34830 | 1.84E-05 | 0.074 | - | - |
| AT3G03100 | 1.57E-05 | 0.074 | - | - |
| AT2G14520 | 1.24E-05 | 0.074 | - | - |
| AT3G44310 | 1.14E-05 | 0.074 | - | - |
| AT1G55210 | 1.13E-05 | 0.074 | - | - |
| AT4G21150 | 3.47E-05 | 0.075 | - | - |
| AT2G04350 | 3.11E-05 | 0.075 | - | - |
| AT1G71860 | 3.08E-05 | 0.075 | - | - |
| AT3G45780 | 2.63E-05 | 0.075 | - | - |
| AT1G06430 | 2.46E-05 | 0.075 | - | - |
| AT2G40690 | 2.37E-05 | 0.075 | - | - |
| AT3G03450 | 2.06E-05 | 0.075 | - | - |
| AT2G35670 | 2.04E-05 | 0.075 | - | - |
| AT5G02830 | 1.92E-05 | 0.075 | - | - |
| AT3G14110 | 1.85E-05 | 0.075 | - | - |
| AT1G77110 | 1.54E-05 | 0.075 | - | - |
| AT1G68020 | 1.53E-05 | 0.075 | - | - |
| AT5G05610 | 1.52E-05 | 0.075 | - | - |
| AT1G12570 | 1.49E-05 | 0.075 | - | - |
| AT3G02170 | 1.47E-05 | 0.075 | - | - |
| AT1G59940 | 1.42E-05 | 0.075 | - | - |
| AT5G01890 | 1.17E-05 | 0.075 | - | - |
| AT1G17120 | 1.12E-05 | 0.075 | - | - |
| AT2G29330 | 1.01E-05 | 0.075 | - | - |
| AT2G25870 | 2.55E-05 | 0.076 | - | - |
| AT3G54340 | 2.44E-05 | 0.076 | - | - |
| AT1G80760 | 2.19E-05 | 0.076 | - | - |
| AT1G14850 | 2.13E-05 | 0.076 | - | - |
| AT5G63570 | 2.08E-05 | 0.076 | - | - |
| AT3G12500 | 2.05E-05 | 0.076 | - | - |
| AT5G21105 | 2.01E-05 | 0.076 | - | - |
| AT4G12290 | 2.00E-05 | 0.076 | - | - |
| AT1G62430 | 1.96E-05 | 0.076 | - | - |
| AT4G02990 | 1.94E-05 | 0.076 | - | - |
| AT4G23750 | 1.57E-05 | 0.076 | - | - |
| AT4G00730 | 1.41E-05 | 0.076 | - | - |
| AT3G46630 | 1.40E-05 | 0.076 | - | - |
| AT4G02380 | 1.35E-05 | 0.076 | - | - |
| AT1G21230 | 1.24E-05 | 0.076 | - | - |
| AT2G21210 | 1.20E-05 | 0.076 | - | - |
| AT1G05900 | 1.14E-05 | 0.076 | - | - |
| AT5G57700 | 1.12E-05 | 0.076 | - | - |
| AT2G27510 | 1.01E-05 | 0.076 | - | - |
| AT5G05490 | 3.84E-05 | 0.077 | - | - |
| AT2G46860 | 3.13E-05 | 0.077 | - | - |
| AT4G12730 | 2.66E-05 | 0.077 | - | - |
| AT5G49630 | 1.95E-05 | 0.077 | - | - |
| AT1G27440 | 1.89E-05 | 0.077 | - | - |
| AT1G22780 | 1.78E-05 | 0.077 | - | - |
| AT1G75040 | 1.71E-05 | 0.077 | - | - |
| AT5G64760 | 1.70E-05 | 0.077 | - | - |
| AT1G23800 | 1.51E-05 | 0.077 | - | - |
| AT2G33070 | 1.30E-05 | 0.077 | - | - |
| AT4G28110 | 1.21E-05 | 0.077 | - | - |
| AT3G13540 | 1.05E-05 | 0.077 | - | - |
| AT2G06960 | 1.03E-05 | 0.077 | - | - |
| AT4G25910 | 3.28E-05 | 0.078 | - | - |
| AT5G64350 | 3.15E-05 | 0.078 | - | - |
| AT3G47370 | 2.95E-05 | 0.078 | - | - |
| AT1G79470 | 2.53E-05 | 0.078 | - | - |
| AT3G04290 | 2.42E-05 | 0.078 | - | - |
| AT1G69620 | 2.39E-05 | 0.078 | - | - |
| AT3G08860 | 2.00E-05 | 0.078 | - | - |
| AT5G53980 | 1.93E-05 | 0.078 | - | - |
| AT5G57040 | 1.86E-05 | 0.078 | - | - |
| AT3G02830 | 1.84E-05 | 0.078 | - | - |
| AT5G14060 | 1.78E-05 | 0.078 | - | - |
| AT5G57960 | 1.77E-05 | 0.078 | - | - |
| AT3G12260 | 1.75E-05 | 0.078 | - | - |
| AT5G28510 | 1.32E-05 | 0.078 | - | - |
| AT5G11560 | 1.14E-05 | 0.078 | - | - |
| AT2G12280 | 1.12E-05 | 0.078 | - | - |
| AT4G19006 | 1.08E-05 | 0.078 | - | - |
| AT3G13870 | 3.50E-05 | 0.079 | - | - |
| AT5G64140 | 2.85E-05 | 0.079 | - | - |
| AT1G33811 | 2.14E-05 | 0.079 | - | - |
| AT5G41340 | 2.03E-05 | 0.079 | - | - |
| AT3G05910 | 1.85E-05 | 0.079 | - | - |
| ATCG00720 | 1.72E-05 | 0.079 | - | - |
| AT1G11790 | 1.55E-05 | 0.079 | - | - |
| AT3G61630 | 1.44E-05 | 0.079 | - | - |
| AT1G14510 | 1.41E-05 | 0.079 | - | - |
| AT3G63110 | 1.39E-05 | 0.079 | - | - |
| AT3G12800 | 1.26E-05 | 0.079 | - | - |
| AT2G44640 | 1.18E-05 | 0.079 | - | - |
| ATCG01300 | 1.12E-05 | 0.079 | - | - |
| AT2G12200 | 1.11E-05 | 0.079 | - | - |
| AT4G07960 | 1.02E-05 | 0.079 | - | - |
| AT5G57560 | 2.52E-05 | 0.08 | - | - |
| AT5G64940 | 1.90E-05 | 0.08 | - | - |
| AT2G43760 | 1.88E-05 | 0.08 | - | - |
| AT5G15860 | 1.83E-05 | 0.08 | - | - |
| AT3G59900 | 1.71E-05 | 0.08 | - | - |
| AT2G38660 | 1.50E-05 | 0.08 | - | - |
| AT1G71040 | 1.14E-05 | 0.08 | - | - |
| AT2G28620 | 3.40E-05 | 0.081 | - | - |
| AT5G13410 | 2.62E-05 | 0.081 | - | - |
| AT1G68660 | 2.47E-05 | 0.081 | - | - |
| AT2G44620 | 2.46E-05 | 0.081 | - | - |
| AT5G50430 | 2.32E-05 | 0.081 | - | - |
| AT1G76810 | 2.28E-05 | 0.081 | - | - |
| AT5G44680 | 2.06E-05 | 0.081 | - | - |
| AT3G20865 | 1.93E-05 | 0.081 | - | - |
| AT4G23900 | 1.64E-05 | 0.081 | - | - |
| AT2G35490 | 1.46E-05 | 0.081 | - | - |
| AT5G40880 | 1.44E-05 | 0.081 | - | - |
| AT5G46430 | 1.43E-05 | 0.081 | - | - |
| AT1G45249 | 1.41E-05 | 0.081 | - | - |
| AT3G46600 | 1.40E-05 | 0.081 | - | - |
| AT3G25230 | 1.24E-05 | 0.081 | - | - |
| AT1G01230 | 1.21E-05 | 0.081 | - | - |
| AT5G53810 | 1.14E-05 | 0.081 | - | - |
| AT3G25570 | 1.10E-05 | 0.081 | - | - |
| AT2G30490 | 2.60E-05 | 0.082 | - | - |
| AT1G69180 | 2.06E-05 | 0.082 | - | - |
| AT4G27160 | 1.77E-05 | 0.082 | - | - |
| AT2G19990 | 1.73E-05 | 0.082 | - | - |
| AT4G30030 | 1.52E-05 | 0.082 | - | - |
| AT2G46410 | 1.42E-05 | 0.082 | - | - |
| ATCG00770 | 1.21E-05 | 0.082 | - | - |
| AT4G29640 | 1.20E-05 | 0.082 | - | - |
| AT3G26590 | 1.09E-05 | 0.082 | - | - |
| AT4G18280 | 1.05E-05 | 0.082 | - | - |
| AT4G28360 | 1.02E-05 | 0.082 | - | - |
| AT1G43170 | 3.09E-05 | 0.083 | - | - |
| AT2G39670 | 2.75E-05 | 0.083 | - | - |
| AT2G30110 | 2.46E-05 | 0.083 | - | - |
| AT5G27770 | 2.38E-05 | 0.083 | - | - |
| AT1G45000 | 1.69E-05 | 0.083 | - | - |
| AT1G08090 | 1.69E-05 | 0.083 | - | - |
| AT5G54290 | 1.45E-05 | 0.083 | - | - |
| AT1G01650 | 1.43E-05 | 0.083 | - | - |
| AT4G19100 | 1.38E-05 | 0.083 | - | - |
| AT5G08170 | 1.22E-05 | 0.083 | - | - |
| AT1G33230 | 1.18E-05 | 0.083 | - | - |
| AT5G54970 | 1.09E-05 | 0.083 | - | - |
| AT5G25370 | 1.07E-05 | 0.083 | - | - |
| AT2G30200 | 3.30E-05 | 0.084 | - | - |
| AT5G08530 | 2.68E-05 | 0.084 | - | - |
| AT3G26280 | 2.53E-05 | 0.084 | - | - |
| AT4G34720 | 2.33E-05 | 0.084 | - | - |
| AT5G55280 | 2.32E-05 | 0.084 | - | - |
| AT5G59090 | 2.26E-05 | 0.084 | - | - |
| AT1G53520 | 2.10E-05 | 0.084 | - | - |
| AT5G16370 | 1.92E-05 | 0.084 | - | - |
| AT2G47400 | 1.84E-05 | 0.084 | - | - |
| AT2G43710 | 1.67E-05 | 0.084 | - | - |
| AT2G34700 | 1.56E-05 | 0.084 | - | - |
| AT1G54520 | 1.09E-05 | 0.084 | - | - |
| AT3G29810 | 1.08E-05 | 0.084 | - | - |
| AT5G56730 | 1.03E-05 | 0.084 | - | - |
| AT1G09130 | 1.02E-05 | 0.084 | - | - |
| AT3G07850 | 3.82E-05 | 0.085 | - | - |
| AT3G27380 | 2.76E-05 | 0.085 | - | - |
| AT5G40650 | 2.72E-05 | 0.085 | - | - |
| AT2G47110 | 2.71E-05 | 0.085 | - | - |
| AT3G42050 | 2.64E-05 | 0.085 | - | - |
| AT2G45190 | 2.37E-05 | 0.085 | - | - |
| AT3G57290 | 2.13E-05 | 0.085 | - | - |
| AT1G27340 | 2.03E-05 | 0.085 | - | - |
| AT1G04710 | 1.95E-05 | 0.085 | - | - |
| AT3G23300 | 1.66E-05 | 0.085 | - | - |
| AT2G21060 | 1.55E-05 | 0.085 | - | - |
| AT5G44130 | 1.46E-05 | 0.085 | - | - |
| AT4G01480 | 4.09E-05 | 0.086 | - | - |
| AT1G75100 | 3.68E-05 | 0.086 | - | - |
| AT5G23050 | 2.54E-05 | 0.086 | - | - |
| AT5G44420 | 2.40E-05 | 0.086 | - | - |
| AT5G43080 | 1.59E-05 | 0.086 | - | - |
| AT2G38905 | 1.57E-05 | 0.086 | - | - |
| AT2G25890 | 1.54E-05 | 0.086 | - | - |
| AT2G38750 | 1.47E-05 | 0.086 | - | - |
| AT1G75440 | 1.14E-05 | 0.086 | - | - |
| AT3G19450 | 2.94E-05 | 0.087 | - | - |
| AT1G52920 | 2.89E-05 | 0.087 | - | - |
| AT1G73500 | 2.17E-05 | 0.087 | - | - |
| AT5G60910 | 2.11E-05 | 0.087 | - | - |
| AT3G09390 | 1.61E-05 | 0.087 | - | - |
| AT4G01120 | 1.51E-05 | 0.087 | - | - |
| AT3G28480 | 1.31E-05 | 0.087 | - | - |
| AT1G54150 | 1.29E-05 | 0.087 | - | - |
| AT1G68520 | 2.74E-05 | 0.088 | - | - |
| AT2G01760 | 2.54E-05 | 0.088 | - | - |
| AT5G52650 | 2.26E-05 | 0.088 | - | - |
| AT3G07430 | 2.10E-05 | 0.088 | - | - |
| AT2G43360 | 2.02E-05 | 0.088 | - | - |
| AT1G55880 | 2.00E-05 | 0.088 | - | - |
| AT4G00560 | 1.75E-05 | 0.088 | - | - |
| AT3G21760 | 1.39E-05 | 0.088 | - | - |
| AT3G50440 | 1.11E-05 | 0.088 | - | - |
| AT1G35190 | 1.03E-05 | 0.088 | - | - |
| AT2G41710 | 1.02E-05 | 0.088 | - | - |
| AT2G43430 | 1.02E-05 | 0.088 | - | - |
| AT4G19750 | 1.01E-05 | 0.088 | - | - |
| AT1G01060 | 2.81E-05 | 0.089 | - | - |
| AT5G60920 | 2.77E-05 | 0.089 | - | - |
| AT4G13170 | 2.45E-05 | 0.089 | - | - |
| AT3G46550 | 2.39E-05 | 0.089 | - | - |
| AT1G51805 | 1.65E-05 | 0.089 | - | - |
| AT1G73530 | 1.50E-05 | 0.089 | - | - |
| ATCG00340 | 1.43E-05 | 0.089 | - | - |
| AT5G28440 | 1.21E-05 | 0.089 | - | - |
| AT1G09420 | 1.19E-05 | 0.089 | - | - |
| AT2G25450 | 1.19E-05 | 0.089 | - | - |
| AT3G12040 | 1.07E-05 | 0.089 | - | - |
| AT1G03860 | 3.27E-05 | 0.09 | - | - |
| AT1G19910 | 2.56E-05 | 0.09 | - | - |
| AT1G73590 | 2.31E-05 | 0.09 | - | - |
| AT1G54580 | 1.96E-05 | 0.09 | - | - |
| AT4G12970 | 1.50E-05 | 0.09 | - | - |
| AT3G01550 | 1.30E-05 | 0.09 | - | - |
| AT2G16005 | 1.27E-05 | 0.09 | - | - |
| AT1G32380 | 1.09E-05 | 0.09 | - | - |
| AT2G46970 | 1.02E-05 | 0.09 | - | - |
| AT1G56600 | 2.23E-05 | 0.091 | - | - |
| AT1G01790 | 2.14E-05 | 0.091 | - | - |
| AT1G02280 | 2.04E-05 | 0.091 | - | - |
| AT2G42840 | 1.70E-05 | 0.091 | - | - |
| AT4G35700 | 1.58E-05 | 0.091 | - | - |
| AT3G05870 | 1.41E-05 | 0.091 | - | - |
| AT5G37780 | 1.36E-05 | 0.091 | - | - |
| AT2G48070 | 2.81E-05 | 0.092 | - | - |
| AT1G76920 | 1.62E-05 | 0.092 | - | - |
| ATMG01080 | 1.57E-05 | 0.092 | - | - |
| AT2G38040 | 1.17E-05 | 0.092 | - | - |
| AT5G44930 | 1.14E-05 | 0.092 | - | - |
| AT4G27110 | 1.09E-05 | 0.092 | - | - |
| AT2G01735 | 1.07E-05 | 0.092 | - | - |
| AT2G33100 | 1.06E-05 | 0.092 | - | - |
| AT3G07670 | 3.09E-05 | 0.093 | - | - |
| AT1G17745 | 2.09E-05 | 0.093 | - | - |
| AT3G58500 | 2.02E-05 | 0.093 | - | - |
| AT5G57590 | 1.67E-05 | 0.093 | - | - |
| AT3G26610 | 1.51E-05 | 0.093 | - | - |
| AT3G62290 | 1.07E-05 | 0.093 | - | - |
| AT5G51020 | 1.05E-05 | 0.093 | - | - |
| AT1G72820 | 1.01E-05 | 0.093 | - | - |
| AT1G74960 | 3.13E-05 | 0.094 | - | - |
| AT3G58140 | 3.04E-05 | 0.094 | - | - |
| AT5G49970 | 2.91E-05 | 0.094 | - | - |
| AT3G09440 | 2.75E-05 | 0.094 | - | - |
| AT2G07698 | 2.49E-05 | 0.094 | - | - |
| AT1G67110 | 2.33E-05 | 0.094 | - | - |
| AT1G51650 | 2.28E-05 | 0.094 | - | - |
| AT1G80680 | 2.26E-05 | 0.094 | - | - |
| AT5G27850 | 2.25E-05 | 0.094 | - | - |
| AT5G18100 | 1.92E-05 | 0.094 | - | - |
| AT3G12977 | 1.82E-05 | 0.094 | - | - |
| AT3G61060 | 1.71E-05 | 0.094 | - | - |
| AT5G35600 | 1.70E-05 | 0.094 | - | - |
| AT5G23010 | 1.65E-05 | 0.094 | - | - |
| AT4G14140 | 1.65E-05 | 0.094 | - | - |
| AT1G30960 | 1.64E-05 | 0.094 | - | - |
| AT1G52150 | 1.44E-05 | 0.094 | - | - |
| AT4G29630 | 1.14E-05 | 0.094 | - | - |
| AT5G09730 | 1.06E-05 | 0.094 | - | - |
| AT1G68720 | 1.01E-05 | 0.094 | - | - |
| AT3G46460 | 2.36E-05 | 0.095 | - | - |
| AT1G02305 | 1.94E-05 | 0.095 | - | - |
| AT1G13400 | 1.93E-05 | 0.095 | - | - |
| AT3G62020 | 1.62E-05 | 0.095 | - | - |
| AT1G69780 | 1.51E-05 | 0.095 | - | - |
| AT5G49450 | 1.43E-05 | 0.095 | - | - |
| AT4G03415 | 1.16E-05 | 0.095 | - | - |
| AT3G14360 | 1.10E-05 | 0.095 | - | - |
| AT2G43330 | 1.06E-05 | 0.095 | - | - |
| AT5G40280 | 2.02E-05 | 0.096 | - | - |
| AT5G13140 | 1.90E-05 | 0.096 | - | - |
| AT2G02010 | 1.60E-05 | 0.096 | - | - |
| AT1G01210 | 1.53E-05 | 0.096 | - | - |
| AT3G23000 | 1.37E-05 | 0.096 | - | - |
| AT5G07210 | 1.30E-05 | 0.096 | - | - |
| AT2G36830 | 1.20E-05 | 0.096 | - | - |
| AT1G53670 | 1.01E-05 | 0.096 | - | - |
| AT2G46560 | 3.72E-05 | 0.097 | - | - |
| AT3G19510 | 3.16E-05 | 0.097 | - | - |
| AT5G24360 | 2.51E-05 | 0.097 | - | - |
| AT1G19670 | 2.48E-05 | 0.097 | - | - |
| AT1G62940 | 2.25E-05 | 0.097 | - | - |
| ATMG00160 | 2.14E-05 | 0.097 | - | - |
| AT1G76180 | 1.91E-05 | 0.097 | - | - |
| AT3G05530 | 1.89E-05 | 0.097 | - | - |
| AT5G40420 | 1.71E-05 | 0.097 | - | - |
| AT1G11910 | 1.66E-05 | 0.097 | - | - |
| AT4G24540 | 1.48E-05 | 0.097 | - | - |
| AT3G12470 | 1.27E-05 | 0.097 | - | - |
| AT1G72030 | 2.86E-05 | 0.098 | - | - |
| AT5G45775 | 2.77E-05 | 0.098 | - | - |
| AT2G34250 | 2.51E-05 | 0.098 | - | - |
| AT5G51900 | 2.00E-05 | 0.098 | - | - |
| AT5G05580 | 1.99E-05 | 0.098 | - | - |
| AT1G64720 | 1.84E-05 | 0.098 | - | - |
| AT2G21580 | 1.73E-05 | 0.098 | - | - |
| AT4G30610 | 1.59E-05 | 0.098 | - | - |
| AT3G16360 | 1.55E-05 | 0.098 | - | - |
| AT4G34350 | 1.51E-05 | 0.098 | - | - |
| AT4G30993 | 1.50E-05 | 0.098 | - | - |
| AT3G21870 | 1.42E-05 | 0.098 | - | - |
| AT3G50210 | 1.30E-05 | 0.098 | - | - |
| AT5G53470 | 1.23E-05 | 0.098 | - | - |
| AT5G55620 | 1.21E-05 | 0.098 | - | - |
| AT1G23205 | 1.10E-05 | 0.098 | - | - |
| AT4G29600 | 1.09E-05 | 0.098 | - | - |
| AT1G20575 | 2.44E-05 | 0.099 | - | - |
| AT3G59980 | 1.90E-05 | 0.099 | - | - |
| AT3G17205 | 1.72E-05 | 0.099 | - | - |
| AT3G23870 | 1.70E-05 | 0.099 | - | - |
| AT5G41210 | 1.68E-05 | 0.099 | - | - |
| AT3G09560 | 1.62E-05 | 0.099 | - | - |
| AT1G04980 | 1.52E-05 | 0.099 | - | - |
| AT1G01170 | 1.48E-05 | 0.099 | - | - |
| AT1G52370 | 1.43E-05 | 0.099 | - | - |
| AT4G11310 | 1.43E-05 | 0.099 | - | - |
| AT5G24900 | 1.34E-05 | 0.099 | - | - |
| AT3G10770 | 1.33E-05 | 0.099 | - | - |
| AT2G25710 | 1.28E-05 | 0.099 | - | - |
| AT2G45030 | 1.20E-05 | 0.099 | - | - |
| AT3G25160 | 1.04E-05 | 0.099 | - | - |
| AT5G48220 | 3.20E-05 | 0.1 | - | - |
| AT5G66680 | 3.17E-05 | 0.1 | - | - |
| AT3G13790 | 3.06E-05 | 0.1 | - | - |
| AT3G52550 | 2.88E-05 | 0.1 | - | - |
| AT3G17040 | 2.59E-05 | 0.1 | - | - |
| AT1G62180 | 2.08E-05 | 0.1 | - | - |
| AT2G36870 | 2.06E-05 | 0.1 | - | - |
| AT3G17760 | 1.72E-05 | 0.1 | - | - |
| AT3G04910 | 1.51E-05 | 0.1 | - | - |
| AT1G07140 | 1.47E-05 | 0.1 | - | - |
| AT4G01950 | 1.41E-05 | 0.1 | - | - |
| AT1G30220 | 1.36E-05 | 0.1 | - | - |
| AT4G17710 | 1.35E-05 | 0.1 | - | - |
| AT3G12460 | 1.23E-05 | 0.1 | - | - |
| AT2G20570 | 1.17E-05 | 0.1 | - | - |
| AT5G65165 | 2.68E-05 | 0.101 | - | - |
| AT5G43810 | 2.26E-05 | 0.101 | - | - |
| AT5G48490 | 2.17E-05 | 0.101 | - | - |
| AT1G52190 | 2.16E-05 | 0.101 | - | - |
| AT1G31230 | 2.14E-05 | 0.101 | - | - |
| AT4G26250 | 2.10E-05 | 0.101 | - | - |
| AT4G26230 | 2.05E-05 | 0.101 | - | - |
| AT5G28350 | 1.56E-05 | 0.101 | - | - |
| AT2G31360 | 1.48E-05 | 0.101 | - | - |
| AT2G35010 | 1.28E-05 | 0.101 | - | - |
| AT2G43400 | 1.25E-05 | 0.101 | - | - |
| AT1G60920 | 1.22E-05 | 0.101 | - | - |
| AT3G62600 | 1.08E-05 | 0.101 | - | - |
| AT5G52520 | 2.73E-05 | 0.102 | - | - |
| AT3G26900 | 2.30E-05 | 0.102 | - | - |
| AT2G41620 | 2.28E-05 | 0.102 | - | - |
| AT5G51290 | 1.88E-05 | 0.102 | - | - |
| AT1G14830 | 1.87E-05 | 0.102 | - | - |
| AT2G33610 | 1.66E-05 | 0.102 | - | - |
| AT1G06080 | 1.46E-05 | 0.102 | - | - |
| AT4G36700 | 1.34E-05 | 0.102 | - | - |
| AT1G79720 | 1.31E-05 | 0.102 | - | - |
| AT5G03545 | 1.05E-05 | 0.102 | - | - |
| AT3G21110 | 3.22E-05 | 0.103 | - | - |
| AT3G53740 | 2.95E-05 | 0.103 | - | - |
| AT2G46270 | 2.84E-05 | 0.103 | - | - |
| AT2G44610 | 2.48E-05 | 0.103 | - | - |
| AT3G04080 | 2.27E-05 | 0.103 | - | - |
| AT2G44040 | 2.17E-05 | 0.103 | - | - |
| AT1G12860 | 2.09E-05 | 0.103 | - | - |
| AT1G60990 | 1.92E-05 | 0.103 | - | - |
| AT5G26742 | 1.62E-05 | 0.103 | - | - |
| AT5G56760 | 1.52E-05 | 0.103 | - | - |
| AT3G12420 | 1.27E-05 | 0.103 | - | - |
| AT4G37470 | 1.14E-05 | 0.103 | - | - |
| AT1G80230 | 1.13E-05 | 0.103 | - | - |
| AT5G65080 | 1.09E-05 | 0.103 | - | - |
| AT3G03640 | 1.06E-05 | 0.103 | - | - |
| AT5G43360 | 1.05E-05 | 0.103 | - | - |
| AT3G18130 | 3.73E-05 | 0.104 | - | - |
| AT1G32900 | 3.26E-05 | 0.104 | - | - |
| AT5G18280 | 2.60E-05 | 0.104 | - | - |
| AT5G01330 | 2.08E-05 | 0.104 | - | - |
| AT2G26800 | 1.98E-05 | 0.104 | - | - |
| AT1G62570 | 1.93E-05 | 0.104 | - | - |
| AT5G59310 | 1.35E-05 | 0.104 | - | - |
| AT3G02140 | 1.33E-05 | 0.104 | - | - |
| AT5G41480 | 1.32E-05 | 0.104 | - | - |
| AT3G21280 | 1.31E-05 | 0.104 | - | - |
| AT2G33740 | 1.25E-05 | 0.104 | - | - |
| AT1G47128 | 1.19E-05 | 0.104 | - | - |
| AT1G61810 | 1.15E-05 | 0.104 | - | - |
| AT2G25840 | 3.08E-05 | 0.105 | - | - |
| AT4G31610 | 2.85E-05 | 0.105 | - | - |
| AT2G20635 | 2.72E-05 | 0.105 | - | - |
| AT5G27200 | 2.08E-05 | 0.105 | - | - |
| AT1G33810 | 1.88E-05 | 0.105 | - | - |
| AT1G21700 | 1.62E-05 | 0.105 | - | - |
| AT1G48020 | 1.54E-05 | 0.105 | - | - |
| AT3G16860 | 1.54E-05 | 0.105 | - | - |
| AT5G09978 | 1.49E-05 | 0.105 | - | - |
| AT5G13780 | 1.37E-05 | 0.105 | - | - |
| AT5G64250 | 1.22E-05 | 0.105 | - | - |
| AT2G30080 | 1.05E-05 | 0.105 | - | - |
| AT2G41530 | 2.17E-05 | 0.106 | - | - |
| AT1G74850 | 1.46E-05 | 0.106 | - | - |
| AT1G14290 | 1.36E-05 | 0.106 | - | - |
| AT5G43320 | 1.35E-05 | 0.106 | - | - |
| AT3G52370 | 1.33E-05 | 0.106 | - | - |
| AT2G40300 | 1.33E-05 | 0.106 | - | - |
| AT3G52290 | 1.24E-05 | 0.106 | - | - |
| AT5G59140 | 2.83E-05 | 0.107 | - | - |
| AT3G13330 | 2.20E-05 | 0.107 | - | - |
| AT5G02450 | 2.10E-05 | 0.107 | - | - |
| AT2G26540 | 1.95E-05 | 0.107 | - | - |
| AT4G38590 | 1.86E-05 | 0.107 | - | - |
| AT4G36870 | 1.61E-05 | 0.107 | - | - |
| AT3G13220 | 1.48E-05 | 0.107 | - | - |
| AT2G47430 | 1.46E-05 | 0.107 | - | - |
| AT3G20050 | 3.86E-05 | 0.108 | - | - |
| AT4G38240 | 3.50E-05 | 0.108 | - | - |
| AT3G12610 | 2.70E-05 | 0.108 | - | - |
| AT5G27620 | 2.62E-05 | 0.108 | - | - |
| AT4G21750 | 2.40E-05 | 0.108 | - | - |
| AT2G23350 | 2.29E-05 | 0.108 | - | - |
| AT5G50460 | 1.71E-05 | 0.108 | - | - |
| AT3G22840 | 1.70E-05 | 0.108 | - | - |
| AT4G15545 | 1.60E-05 | 0.108 | - | - |
| AT3G61030 | 1.52E-05 | 0.108 | - | - |
| AT4G36760 | 1.50E-05 | 0.108 | - | - |
| AT2G23570 | 1.48E-05 | 0.108 | - | - |
| AT4G13420 | 1.22E-05 | 0.108 | - | - |
| AT5G18830 | 1.21E-05 | 0.108 | - | - |
| AT4G00240 | 1.14E-05 | 0.108 | - | - |
| AT2G30590 | 1.06E-05 | 0.108 | - | - |
| AT3G52180 | 2.83E-05 | 0.109 | - | - |
| AT1G29970 | 2.46E-05 | 0.109 | - | - |
| AT1G14420 | 2.44E-05 | 0.109 | - | - |
| AT1G73720 | 2.40E-05 | 0.109 | - | - |
| AT5G02960 | 2.22E-05 | 0.109 | - | - |
| AT4G01320 | 2.21E-05 | 0.109 | - | - |
| AT2G34390 | 2.01E-05 | 0.109 | - | - |
| AT3G60620 | 1.50E-05 | 0.109 | - | - |
| AT2G40960 | 1.34E-05 | 0.109 | - | - |
| AT4G32530 | 1.11E-05 | 0.109 | - | - |
| AT1G62300 | 2.80E-05 | 0.11 | - | - |
| AT5G11380 | 1.99E-05 | 0.11 | - | - |
| AT4G35490 | 1.91E-05 | 0.11 | - | - |
| AT1G15210 | 1.88E-05 | 0.11 | - | - |
| AT1G14280 | 1.49E-05 | 0.11 | - | - |
| AT5G16620 | 1.36E-05 | 0.11 | - | - |
| AT5G24420 | 1.93E-05 | 0.111 | - | - |
| AT2G18030 | 1.48E-05 | 0.111 | - | - |
| AT5G56720 | 5.42E-05 | 0.112 | - | - |
| AT3G55610 | 3.86E-05 | 0.112 | - | - |
| AT1G77120 | 2.65E-05 | 0.112 | - | - |
| AT4G26000 | 2.54E-05 | 0.112 | - | - |
| AT5G55540 | 1.98E-05 | 0.112 | - | - |
| AT5G38530 | 1.75E-05 | 0.112 | - | - |
| AT4G28420 | 1.60E-05 | 0.112 | - | - |
| AT3G21510 | 1.43E-05 | 0.112 | - | - |
| AT1G64290 | 1.38E-05 | 0.112 | - | - |
| AT1G06400 | 1.32E-05 | 0.112 | - | - |
| AT5G66460 | 1.20E-05 | 0.112 | - | - |
| AT1G80100 | 1.14E-05 | 0.112 | - | - |
| AT3G13730 | 1.10E-05 | 0.112 | - | - |
| AT2G16660 | 1.04E-05 | 0.112 | - | - |
| AT3G20440 | 2.17E-05 | 0.113 | - | - |
| AT5G51970 | 2.12E-05 | 0.113 | - | - |
| AT1G51140 | 2.06E-05 | 0.113 | - | - |
| ATCG00470 | 1.66E-05 | 0.113 | - | - |
| AT5G63090 | 1.66E-05 | 0.113 | - | - |
| AT5G47890 | 1.64E-05 | 0.113 | - | - |
| AT4G19420 | 1.46E-05 | 0.113 | - | - |
| AT1G21450 | 1.38E-05 | 0.113 | - | - |
| AT3G19260 | 1.37E-05 | 0.113 | - | - |
| AT5G25270 | 1.36E-05 | 0.113 | - | - |
| AT5G46180 | 2.73E-05 | 0.114 | - | - |
| AT1G64190 | 2.28E-05 | 0.114 | - | - |
| AT1G27450 | 2.16E-05 | 0.114 | - | - |
| AT1G59359 | 1.80E-05 | 0.114 | - | - |
| AT2G29650 | 1.74E-05 | 0.114 | - | - |
| AT3G10600 | 1.38E-05 | 0.114 | - | - |
| AT2G30830 | 1.22E-05 | 0.114 | - | - |
| AT1G05385 | 1.00E-05 | 0.114 | - | - |
| AT1G50430 | 2.48E-05 | 0.115 | - | - |
| AT5G52240 | 2.24E-05 | 0.115 | - | - |
| AT4G36130 | 2.08E-05 | 0.115 | - | - |
| AT1G54630 | 2.01E-05 | 0.115 | - | - |
| AT1G50420 | 1.98E-05 | 0.115 | - | - |
| AT2G02040 | 1.64E-05 | 0.115 | - | - |
| AT5G15580 | 1.55E-05 | 0.115 | - | - |
| AT3G57020 | 1.44E-05 | 0.115 | - | - |
| AT5G49070 | 1.42E-05 | 0.115 | - | - |
| AT1G73960 | 1.32E-05 | 0.115 | - | - |
| AT3G44830 | 1.29E-05 | 0.115 | - | - |
| AT1G74010 | 1.18E-05 | 0.115 | - | - |
| AT3G50070 | 1.14E-05 | 0.115 | - | - |
| AT5G41050 | 1.09E-05 | 0.115 | - | - |
| AT5G16130 | 2.66E-05 | 0.116 | - | - |
| AT1G49650 | 2.45E-05 | 0.116 | - | - |
| AT1G17560 | 2.11E-05 | 0.116 | - | - |
| AT5G19370 | 1.76E-05 | 0.116 | - | - |
| AT4G11820 | 1.73E-05 | 0.116 | - | - |
| ATCG00150 | 1.61E-05 | 0.116 | - | - |
| AT1G05940 | 1.33E-05 | 0.116 | - | - |
| AT3G60900 | 1.14E-05 | 0.116 | - | - |
| AT4G38620 | 1.09E-05 | 0.116 | - | - |
| AT3G46690 | 1.02E-05 | 0.116 | - | - |
| AT4G37260 | 1.02E-05 | 0.116 | - | - |
| AT1G13340 | 3.34E-05 | 0.117 | - | - |
| AT3G53750 | 3.03E-05 | 0.117 | - | - |
| AT2G18400 | 2.63E-05 | 0.117 | - | - |
| AT1G03440 | 2.34E-05 | 0.117 | - | - |
| AT3G57140 | 2.31E-05 | 0.117 | - | - |
| AT1G10370 | 2.22E-05 | 0.117 | - | - |
| AT5G06460 | 2.15E-05 | 0.117 | - | - |
| AT5G15950 | 1.76E-05 | 0.117 | - | - |
| AT5G65280 | 1.23E-05 | 0.117 | - | - |
| AT3G56040 | 1.20E-05 | 0.117 | - | - |
| AT5G52570 | 1.17E-05 | 0.117 | - | - |
| AT4G33910 | 1.14E-05 | 0.117 | - | - |
| AT2G38710 | 1.12E-05 | 0.117 | - | - |
| AT4G19020 | 1.04E-05 | 0.117 | - | - |
| AT5G17170 | 4.32E-05 | 0.118 | - | - |
| AT3G53900 | 2.91E-05 | 0.118 | - | - |
| AT1G69190 | 2.89E-05 | 0.118 | - | - |
| AT3G48560 | 2.63E-05 | 0.118 | - | - |
| AT5G52840 | 2.21E-05 | 0.118 | - | - |
| AT5G17050 | 2.21E-05 | 0.118 | - | - |
| AT1G64650 | 2.10E-05 | 0.118 | - | - |
| AT4G31800 | 1.92E-05 | 0.118 | - | - |
| AT5G20010 | 1.91E-05 | 0.118 | - | - |
| AT1G01160 | 1.79E-05 | 0.118 | - | - |
| AT4G39090 | 1.68E-05 | 0.118 | - | - |
| AT2G30060 | 1.59E-05 | 0.118 | - | - |
| AT1G47610 | 1.50E-05 | 0.118 | - | - |
| AT2G25210 | 1.31E-05 | 0.118 | - | - |
| AT4G27710 | 1.21E-05 | 0.118 | - | - |
| AT2G46480 | 2.78E-05 | 0.119 | - | - |
| AT5G17420 | 2.59E-05 | 0.119 | - | - |
| AT4G37970 | 2.33E-05 | 0.119 | - | - |
| AT1G59970 | 2.09E-05 | 0.119 | - | - |
| AT3G48425 | 1.92E-05 | 0.119 | - | - |
| AT1G01100 | 1.75E-05 | 0.119 | - | - |
| AT1G63120 | 1.30E-05 | 0.119 | - | - |
| AT1G24620 | 1.19E-05 | 0.119 | - | - |
| AT5G65990 | 1.16E-05 | 0.119 | - | - |
| AT1G06550 | 1.06E-05 | 0.119 | - | - |
| AT3G61970 | 1.01E-05 | 0.119 | - | - |
| AT2G20900 | 1.00E-05 | 0.119 | - | - |
| AT3G10340 | 3.09E-05 | 0.12 | - | - |
| AT5G19140 | 2.07E-05 | 0.12 | - | - |
| AT2G45660 | 2.05E-05 | 0.12 | - | - |
| AT4G29680 | 1.94E-05 | 0.12 | - | - |
| AT2G23760 | 1.84E-05 | 0.12 | - | - |
| AT1G64860 | 1.76E-05 | 0.12 | - | - |
| AT2G22980 | 1.70E-05 | 0.12 | - | - |
| AT2G33370 | 1.59E-05 | 0.12 | - | - |
| AT5G06860 | 1.46E-05 | 0.12 | - | - |
| AT4G21590 | 1.23E-05 | 0.12 | - | - |
| AT1G53930 | 1.16E-05 | 0.12 | - | - |
| AT2G31450 | 1.06E-05 | 0.12 | - | - |
| AT1G55740 | 1.05E-05 | 0.12 | - | - |
| AT3G51800 | 3.52E-05 | 0.121 | - | - |
| AT5G58860 | 3.45E-05 | 0.121 | - | - |
| AT5G04560 | 2.37E-05 | 0.121 | - | - |
| AT3G16780 | 2.19E-05 | 0.121 | - | - |
| AT2G26980 | 2.02E-05 | 0.121 | - | - |
| AT5G39340 | 1.95E-05 | 0.121 | - | - |
| AT1G10550 | 1.80E-05 | 0.121 | - | - |
| AT2G20610 | 1.76E-05 | 0.121 | - | - |
| AT4G18360 | 1.68E-05 | 0.121 | - | - |
| AT3G01140 | 1.04E-05 | 0.121 | - | - |
| AT5G54180 | 1.01E-05 | 0.121 | - | - |
| AT5G57930 | 1.01E-05 | 0.121 | - | - |
| AT5G14030 | 3.41E-05 | 0.122 | - | - |
| AT5G04230 | 2.27E-05 | 0.122 | - | - |
| AT5G07340 | 1.96E-05 | 0.122 | - | - |
| AT2G24850 | 1.82E-05 | 0.122 | - | - |
| AT5G57290 | 1.75E-05 | 0.122 | - | - |
| AT3G01120 | 1.72E-05 | 0.122 | - | - |
| AT5G61510 | 1.63E-05 | 0.122 | - | - |
| AT5G25890 | 1.55E-05 | 0.122 | - | - |
| AT5G38620 | 1.40E-05 | 0.122 | - | - |
| AT5G11300 | 1.12E-05 | 0.122 | - | - |
| AT1G77980 | 1.12E-05 | 0.122 | - | - |
| AT4G13250 | 1.05E-05 | 0.122 | - | - |
| AT1G70830 | 1.01E-05 | 0.122 | - | - |
| AT2G27530 | 2.52E-05 | 0.123 | - | - |
| AT3G11830 | 2.36E-05 | 0.123 | - | - |
| AT5G54160 | 1.86E-05 | 0.123 | - | - |
| AT5G02470 | 1.46E-05 | 0.123 | - | - |
| AT5G22300 | 1.39E-05 | 0.123 | - | - |
| AT5G33290 | 1.33E-05 | 0.123 | - | - |
| AT1G53950 | 1.16E-05 | 0.123 | - | - |
| AT5G47670 | 1.13E-05 | 0.123 | - | - |
| ATCG00580 | 1.06E-05 | 0.123 | - | - |
| AT1G09300 | 1.05E-05 | 0.123 | - | - |
| AT5G02100 | 1.05E-05 | 0.123 | - | - |
| AT4G05450 | 1.02E-05 | 0.123 | - | - |
| AT1G17280 | 2.65E-05 | 0.124 | - | - |
| AT2G18020 | 2.64E-05 | 0.124 | - | - |
| AT4G18780 | 2.52E-05 | 0.124 | - | - |
| AT2G21390 | 2.29E-05 | 0.124 | - | - |
| AT1G04480 | 1.56E-05 | 0.124 | - | - |
| AT1G66820 | 1.53E-05 | 0.124 | - | - |
| AT3G20230 | 1.43E-05 | 0.124 | - | - |
| AT1G48860 | 1.29E-05 | 0.124 | - | - |
| AT2G18080 | 1.23E-05 | 0.124 | - | - |
| AT2G28790 | 1.21E-05 | 0.124 | - | - |
| AT5G16570 | 1.20E-05 | 0.124 | - | - |
| AT3G30180 | 1.13E-05 | 0.124 | - | - |
| AT5G03680 | 1.12E-05 | 0.124 | - | - |
| AT2G47890 | 1.03E-05 | 0.124 | - | - |
| AT2G19450 | 3.99E-05 | 0.125 | - | - |
| AT2G28605 | 2.97E-05 | 0.125 | - | - |
| AT5G55730 | 2.96E-05 | 0.125 | - | - |
| AT4G08685 | 2.68E-05 | 0.125 | - | - |
| AT3G01090 | 1.56E-05 | 0.125 | - | - |
| AT1G55370 | 1.48E-05 | 0.125 | - | - |
| AT4G39280 | 1.15E-05 | 0.125 | - | - |
| AT5G66720 | 1.10E-05 | 0.125 | - | - |
| AT5G02030 | 2.51E-05 | 0.126 | - | - |
| AT4G04880 | 2.08E-05 | 0.126 | - | - |
| AT3G04260 | 1.52E-05 | 0.126 | - | - |
| AT5G60640 | 1.49E-05 | 0.126 | - | - |
| AT5G11500 | 1.25E-05 | 0.126 | - | - |
| AT1G21770 | 1.09E-05 | 0.126 | - | - |
| AT2G30440 | 1.01E-05 | 0.126 | - | - |
| AT2G15430 | 3.10E-05 | 0.127 | - | - |
| AT5G22440 | 2.80E-05 | 0.127 | - | - |
| AT2G21890 | 2.19E-05 | 0.127 | - | - |
| AT2G33430 | 2.01E-05 | 0.127 | - | - |
| AT3G45940 | 1.66E-05 | 0.127 | - | - |
| AT5G51990 | 1.53E-05 | 0.127 | - | - |
| AT5G55700 | 1.52E-05 | 0.127 | - | - |
| AT2G24840 | 1.52E-05 | 0.127 | - | - |
| AT5G66280 | 1.42E-05 | 0.127 | - | - |
| AT1G35550 | 1.35E-05 | 0.127 | - | - |
| AT1G48630 | 4.62E-05 | 0.128 | - | - |
| AT4G33150 | 3.70E-05 | 0.128 | - | - |
| AT3G47120 | 2.98E-05 | 0.128 | - | - |
| AT4G19800 | 1.78E-05 | 0.128 | - | - |
| AT5G08000 | 1.70E-05 | 0.128 | - | - |
| AT1G70710 | 1.58E-05 | 0.128 | - | - |
| AT5G61020 | 1.55E-05 | 0.128 | - | - |
| AT5G53550 | 1.49E-05 | 0.128 | - | - |
| AT4G10020 | 1.29E-05 | 0.128 | - | - |
| AT2G21970 | 1.19E-05 | 0.128 | - | - |
| AT2G04390 | 2.35E-05 | 0.129 | - | - |
| AT2G20270 | 2.12E-05 | 0.129 | - | - |
| AT4G16450 | 1.65E-05 | 0.129 | - | - |
| AT2G46950 | 1.62E-05 | 0.129 | - | - |
| AT2G07671 | 1.07E-05 | 0.129 | - | - |
| AT3G18780 | 3.20E-05 | 0.13 | - | - |
| AT5G39850 | 2.64E-05 | 0.13 | - | - |
| AT5G53950 | 1.84E-05 | 0.13 | - | - |
| AT5G43640 | 1.74E-05 | 0.13 | - | - |
| AT1G70200 | 1.41E-05 | 0.13 | - | - |
| AT3G59020 | 1.40E-05 | 0.13 | - | - |
| AT5G47040 | 1.16E-05 | 0.13 | - | - |
| AT1G55090 | 1.16E-05 | 0.13 | - | - |
| AT5G66700 | 1.13E-05 | 0.13 | - | - |
| AT1G24190 | 1.10E-05 | 0.13 | - | - |
| AT4G31920 | 1.08E-05 | 0.13 | - | - |
| AT2G01110 | 2.98E-05 | 0.131 | - | - |
| AT4G16760 | 2.53E-05 | 0.131 | - | - |
| AT3G49010 | 2.29E-05 | 0.131 | - | - |
| AT5G04200 | 2.14E-05 | 0.131 | - | - |
| AT3G06460 | 2.05E-05 | 0.131 | - | - |
| AT2G01450 | 1.82E-05 | 0.131 | - | - |
| AT4G35770 | 1.82E-05 | 0.131 | - | - |
| AT3G50740 | 1.76E-05 | 0.131 | - | - |
| AT2G47180 | 1.61E-05 | 0.131 | - | - |
| AT3G23430 | 1.38E-05 | 0.131 | - | - |
| AT2G39590 | 1.32E-05 | 0.131 | - | - |
| AT1G79620 | 1.23E-05 | 0.131 | - | - |
| AT5G09340 | 1.16E-05 | 0.131 | - | - |
| AT3G10850 | 1.16E-05 | 0.131 | - | - |
| AT1G24120 | 1.10E-05 | 0.131 | - | - |
| AT1G73730 | 1.02E-05 | 0.131 | - | - |
| AT5G01220 | 1.75E-05 | 0.132 | - | - |
| AT5G13790 | 1.74E-05 | 0.132 | - | - |
| AT1G50110 | 1.19E-05 | 0.132 | - | - |
| AT1G69020 | 1.17E-05 | 0.132 | - | - |
| AT4G02950 | 1.05E-05 | 0.132 | - | - |
| AT4G03370 | 1.05E-05 | 0.132 | - | - |
| AT3G16080 | 2.67E-05 | 0.133 | - | - |
| AT1G30630 | 2.01E-05 | 0.133 | - | - |
| AT5G39760 | 1.46E-05 | 0.133 | - | - |
| AT5G63560 | 1.40E-05 | 0.133 | - | - |
| AT1G55325 | 1.38E-05 | 0.133 | - | - |
| AT3G22120 | 1.15E-05 | 0.133 | - | - |
| AT5G04950 | 1.08E-05 | 0.133 | - | - |
| AT3G51870 | 1.01E-05 | 0.133 | - | - |
| AT5G20250 | 2.28E-05 | 0.134 | - | - |
| AT3G02480 | 2.05E-05 | 0.134 | - | - |
| AT4G19710 | 1.96E-05 | 0.134 | - | - |
| AT3G03940 | 1.67E-05 | 0.134 | - | - |
| AT2G38390 | 1.66E-05 | 0.134 | - | - |
| AT4G23940 | 1.60E-05 | 0.134 | - | - |
| AT1G30280 | 1.46E-05 | 0.134 | - | - |
| AT5G19890 | 1.45E-05 | 0.134 | - | - |
| AT2G27050 | 1.31E-05 | 0.134 | - | - |
| AT5G25760 | 1.22E-05 | 0.134 | - | - |
| AT3G21320 | 1.16E-05 | 0.134 | - | - |
| AT3G51895 | 1.07E-05 | 0.134 | - | - |
| AT4G24220 | 1.02E-05 | 0.134 | - | - |
| AT3G61480 | 1.54E-05 | 0.135 | - | - |
| AT3G18030 | 1.37E-05 | 0.135 | - | - |
| AT4G08950 | 1.24E-05 | 0.135 | - | - |
| AT1G71810 | 1.20E-05 | 0.135 | - | - |
| AT1G56050 | 3.81E-05 | 0.136 | - | - |
| AT3G05640 | 2.54E-05 | 0.136 | - | - |
| AT1G71720 | 2.34E-05 | 0.136 | - | - |
| ATCG00140 | 1.95E-05 | 0.136 | - | - |
| AT1G09100 | 1.70E-05 | 0.136 | - | - |
| AT1G37150 | 1.36E-05 | 0.136 | - | - |
| AT3G14650 | 1.12E-05 | 0.136 | - | - |
| AT4G03360 | 1.05E-05 | 0.136 | - | - |
| AT4G16980 | 2.69E-05 | 0.137 | - | - |
| AT5G62700 | 2.36E-05 | 0.137 | - | - |
| AT5G67590 | 2.00E-05 | 0.137 | - | - |
| AT3G23600 | 1.92E-05 | 0.137 | - | - |
| AT4G34860 | 1.72E-05 | 0.137 | - | - |
| AT5G38020 | 1.69E-05 | 0.137 | - | - |
| AT5G16030 | 1.68E-05 | 0.137 | - | - |
| AT4G14340 | 1.62E-05 | 0.137 | - | - |
| AT3G50790 | 1.37E-05 | 0.137 | - | - |
| AT4G00600 | 1.31E-05 | 0.137 | - | - |
| AT3G54110 | 1.04E-05 | 0.137 | - | - |
| AT2G27970 | 3.32E-05 | 0.138 | - | - |
| AT3G16000 | 2.28E-05 | 0.138 | - | - |
| AT5G08160 | 1.78E-05 | 0.138 | - | - |
| AT3G13920 | 1.57E-05 | 0.138 | - | - |
| AT4G34430 | 1.49E-05 | 0.138 | - | - |
| AT4G26120 | 1.25E-05 | 0.138 | - | - |
| AT5G37820 | 1.24E-05 | 0.138 | - | - |
| AT1G26930 | 1.12E-05 | 0.138 | - | - |
| AT4G31700 | 3.42E-05 | 0.139 | - | - |
| AT3G29350 | 2.51E-05 | 0.139 | - | - |
| AT5G58040 | 2.32E-05 | 0.139 | - | - |
| AT3G20780 | 2.24E-05 | 0.139 | - | - |
| AT1G55930 | 1.61E-05 | 0.139 | - | - |
| AT5G64460 | 1.57E-05 | 0.139 | - | - |
| AT5G15230 | 1.56E-05 | 0.139 | - | - |
| AT4G36990 | 1.55E-05 | 0.139 | - | - |
| AT5G14950 | 1.50E-05 | 0.139 | - | - |
| AT1G67680 | 1.39E-05 | 0.139 | - | - |
| AT5G66120 | 1.32E-05 | 0.139 | - | - |
| AT3G51980 | 1.23E-05 | 0.139 | - | - |
| AT2G29380 | 1.21E-05 | 0.139 | - | - |
| AT5G57520 | 1.19E-05 | 0.139 | - | - |
| AT1G64110 | 1.11E-05 | 0.139 | - | - |
| AT2G01460 | 1.02E-05 | 0.139 | - | - |
| AT3G61820 | 2.75E-05 | 0.14 | - | - |
| AT4G14320 | 2.44E-05 | 0.14 | - | - |
| AT2G05140 | 2.18E-05 | 0.14 | - | - |
| AT1G16350 | 2.08E-05 | 0.14 | - | - |
| AT3G49220 | 1.93E-05 | 0.14 | - | - |
| AT3G11710 | 1.73E-05 | 0.14 | - | - |
| AT2G28830 | 1.68E-05 | 0.14 | - | - |
| AT2G27990 | 1.58E-05 | 0.14 | - | - |
| AT2G37450 | 1.23E-05 | 0.14 | - | - |
| AT1G70170 | 1.12E-05 | 0.14 | - | - |
| AT2G32350 | 1.05E-05 | 0.14 | - | - |
| AT2G31610 | 3.09E-05 | 0.141 | - | - |
| AT3G62910 | 2.55E-05 | 0.141 | - | - |
| AT5G24090 | 2.18E-05 | 0.141 | - | - |
| AT2G18370 | 1.85E-05 | 0.141 | - | - |
| AT3G25040 | 1.61E-05 | 0.141 | - | - |
| AT5G39410 | 1.25E-05 | 0.141 | - | - |
| AT4G05270 | 1.05E-05 | 0.141 | - | - |
| AT4G05240 | 1.05E-05 | 0.141 | - | - |
| AT4G05310 | 1.05E-05 | 0.141 | - | - |
| ATMG01190 | 2.72E-05 | 0.142 | - | - |
| AT2G33860 | 2.50E-05 | 0.142 | - | - |
| AT1G48480 | 2.29E-05 | 0.142 | - | - |
| AT4G39120 | 2.23E-05 | 0.142 | - | - |
| AT5G41670 | 2.03E-05 | 0.142 | - | - |
| AT1G65520 | 1.90E-05 | 0.142 | - | - |
| AT3G47390 | 1.80E-05 | 0.142 | - | - |
| AT2G39760 | 1.65E-05 | 0.142 | - | - |
| AT5G61640 | 1.50E-05 | 0.142 | - | - |
| AT3G50880 | 1.25E-05 | 0.142 | - | - |
| AT1G50020 | 1.18E-05 | 0.142 | - | - |
| AT2G17640 | 1.18E-05 | 0.142 | - | - |
| AT1G57860 | 1.51E-05 | 0.143 | - | - |
| AT3G03470 | 1.34E-05 | 0.143 | - | - |
| AT1G53980 | 1.16E-05 | 0.143 | - | - |
| AT3G26710 | 4.01E-05 | 0.144 | - | - |
| AT3G52940 | 3.05E-05 | 0.144 | - | - |
| AT1G53780 | 2.54E-05 | 0.144 | - | - |
| AT2G22450 | 2.33E-05 | 0.144 | - | - |
| AT4G26455 | 2.14E-05 | 0.144 | - | - |
| AT1G31340 | 1.95E-05 | 0.144 | - | - |
| AT4G20960 | 1.88E-05 | 0.144 | - | - |
| AT4G15110 | 1.82E-05 | 0.144 | - | - |
| AT1G06410 | 1.54E-05 | 0.144 | - | - |
| AT2G44520 | 1.51E-05 | 0.144 | - | - |
| AT2G32260 | 1.43E-05 | 0.144 | - | - |
| AT5G03900 | 1.29E-05 | 0.144 | - | - |
| AT3G53580 | 1.17E-05 | 0.144 | - | - |
| AT3G27240 | 2.40E-05 | 0.145 | - | - |
| AT4G02620 | 1.87E-05 | 0.145 | - | - |
| AT3G15640 | 1.70E-05 | 0.145 | - | - |
| AT1G31812 | 1.66E-05 | 0.145 | - | - |
| AT5G25250 | 1.31E-05 | 0.145 | - | - |
| AT5G64300 | 1.29E-05 | 0.145 | - | - |
| AT2G32540 | 1.15E-05 | 0.145 | - | - |
| AT4G12080 | 1.12E-05 | 0.145 | - | - |
| AT4G19120 | 1.10E-05 | 0.145 | - | - |
| AT3G07330 | 2.04E-05 | 0.146 | - | - |
| AT3G10320 | 1.59E-05 | 0.146 | - | - |
| AT5G02490 | 1.58E-05 | 0.146 | - | - |
| AT1G55180 | 1.50E-05 | 0.146 | - | - |
| AT5G59780 | 1.40E-05 | 0.146 | - | - |
| AT1G65330 | 2.10E-05 | 0.147 | - | - |
| AT2G41460 | 1.76E-05 | 0.147 | - | - |
| AT3G10720 | 1.46E-05 | 0.147 | - | - |
| AT1G68670 | 1.45E-05 | 0.147 | - | - |
| AT1G34790 | 1.36E-05 | 0.147 | - | - |
| AT1G23400 | 1.17E-05 | 0.147 | - | - |
| AT2G23240 | 1.05E-05 | 0.147 | - | - |
| AT5G47700 | 1.79E-05 | 0.148 | - | - |
| AT1G80380 | 1.43E-05 | 0.148 | - | - |
| AT5G11420 | 1.29E-05 | 0.148 | - | - |
| AT3G04880 | 1.02E-05 | 0.148 | - | - |
| AT2G27220 | 1.00E-05 | 0.148 | - | - |
| AT5G35530 | 2.91E-05 | 0.149 | - | - |
| AT4G33760 | 2.18E-05 | 0.149 | - | - |
| AT4G30080 | 1.87E-05 | 0.149 | - | - |
| AT3G16640 | 1.77E-05 | 0.149 | - | - |
| AT4G30690 | 1.65E-05 | 0.149 | - | - |
| AT5G09530 | 1.57E-05 | 0.149 | - | - |
| AT1G16180 | 1.03E-05 | 0.149 | - | - |
| AT2G36190 | 2.38E-05 | 0.15 | - | - |
| AT1G60730 | 1.68E-05 | 0.15 | - | - |
| AT2G24940 | 1.55E-05 | 0.15 | - | - |
| AT3G06230 | 1.52E-05 | 0.15 | - | - |
| AT2G28350 | 1.36E-05 | 0.15 | - | - |
| AT2G42210 | 1.21E-05 | 0.15 | - | - |
| AT3G11440 | 1.15E-05 | 0.15 | - | - |
| AT1G75790 | 1.09E-05 | 0.15 | - | - |
| AT5G58250 | 3.84E-05 | 0.151 | - | - |
| AT5G08620 | 2.24E-05 | 0.151 | - | - |
| AT1G69200 | 2.21E-05 | 0.151 | - | - |
| AT2G07695 | 1.95E-05 | 0.151 | - | - |
| AT1G52600 | 1.90E-05 | 0.151 | - | - |
| AT3G54800 | 1.83E-05 | 0.151 | - | - |
| AT5G26667 | 1.49E-05 | 0.151 | - | - |
| AT1G19480 | 1.17E-05 | 0.151 | - | - |
| AT5G57180 | 1.15E-05 | 0.151 | - | - |
| AT2G42260 | 1.13E-05 | 0.151 | - | - |
| AT1G59810 | 1.07E-05 | 0.151 | - | - |
| AT5G38460 | 1.02E-05 | 0.151 | - | - |
| AT3G25520 | 2.98E-05 | 0.152 | - | - |
| AT2G37090 | 2.69E-05 | 0.152 | - | - |
| AT5G40810 | 2.64E-05 | 0.152 | - | - |
| AT1G54280 | 2.15E-05 | 0.152 | - | - |
| AT5G03840 | 2.13E-05 | 0.152 | - | - |
| AT5G10260 | 1.54E-05 | 0.152 | - | - |
| AT5G21040 | 1.53E-05 | 0.152 | - | - |
| AT3G09922 | 1.41E-05 | 0.152 | - | - |
| AT2G39140 | 1.28E-05 | 0.152 | - | - |
| AT1G30825 | 1.22E-05 | 0.152 | - | - |
| AT3G02190 | 1.20E-05 | 0.152 | - | - |
| AT4G32820 | 2.25E-05 | 0.153 | - | - |
| AT5G43710 | 1.37E-05 | 0.153 | - | - |
| AT1G76140 | 1.28E-05 | 0.153 | - | - |
| AT5G17320 | 1.20E-05 | 0.153 | - | - |
| AT1G48170 | 1.15E-05 | 0.153 | - | - |
| AT5G38160 | 1.14E-05 | 0.153 | - | - |
| AT3G10740 | 2.81E-05 | 0.154 | - | - |
| AT1G62390 | 2.60E-05 | 0.154 | - | - |
| AT2G43410 | 1.52E-05 | 0.154 | - | - |
| AT3G23610 | 1.52E-05 | 0.154 | - | - |
| AT5G63160 | 1.44E-05 | 0.154 | - | - |
| AT3G44720 | 1.26E-05 | 0.154 | - | - |
| AT5G59910 | 1.25E-05 | 0.154 | - | - |
| AT5G66960 | 1.15E-05 | 0.154 | - | - |
| AT2G01320 | 1.10E-05 | 0.154 | - | - |
| AT4G23340 | 1.06E-05 | 0.154 | - | - |
| AT3G07390 | 1.03E-05 | 0.154 | - | - |
| AT2G33330 | 1.82E-05 | 0.155 | - | - |
| AT1G80620 | 1.77E-05 | 0.155 | - | - |
| AT5G04810 | 1.66E-05 | 0.155 | - | - |
| AT5G50240 | 1.51E-05 | 0.155 | - | - |
| AT5G53170 | 1.47E-05 | 0.155 | - | - |
| AT2G20860 | 1.42E-05 | 0.155 | - | - |
| AT2G21410 | 1.40E-05 | 0.155 | - | - |
| AT1G21065 | 1.35E-05 | 0.155 | - | - |
| AT5G53360 | 1.27E-05 | 0.155 | - | - |
| AT1G28350 | 1.24E-05 | 0.155 | - | - |
| AT5G53140 | 1.09E-05 | 0.155 | - | - |
| AT2G31060 | 1.01E-05 | 0.155 | - | - |
| AT5G45110 | 2.17E-05 | 0.156 | - | - |
| AT2G28540 | 1.90E-05 | 0.156 | - | - |
| AT3G01390 | 1.77E-05 | 0.156 | - | - |
| AT2G19430 | 1.74E-05 | 0.156 | - | - |
| AT1G04440 | 1.21E-05 | 0.156 | - | - |
| AT5G04250 | 1.06E-05 | 0.156 | - | - |
| AT1G04290 | 1.03E-05 | 0.156 | - | - |
| AT1G10940 | 1.85E-05 | 0.157 | - | - |
| AT5G57170 | 1.76E-05 | 0.157 | - | - |
| AT3G27200 | 1.57E-05 | 0.157 | - | - |
| AT2G39840 | 1.36E-05 | 0.157 | - | - |
| AT5G49980 | 1.33E-05 | 0.157 | - | - |
| AT3G04060 | 1.20E-05 | 0.157 | - | - |
| AT1G79870 | 1.20E-05 | 0.157 | - | - |
| AT2G37690 | 3.11E-05 | 0.158 | - | - |
| AT5G04870 | 2.71E-05 | 0.158 | - | - |
| AT1G13320 | 2.62E-05 | 0.158 | - | - |
| AT5G15530 | 1.81E-05 | 0.158 | - | - |
| AT1G76405 | 1.34E-05 | 0.158 | - | - |
| AT5G16750 | 3.46E-05 | 0.159 | - | - |
| AT2G23810 | 3.24E-05 | 0.159 | - | - |
| AT1G68920 | 2.18E-05 | 0.159 | - | - |
| AT3G61220 | 1.81E-05 | 0.159 | - | - |
| AT3G14040 | 1.43E-05 | 0.159 | - | - |
| AT1G17000 | 1.37E-05 | 0.159 | - | - |
| AT5G04130 | 1.32E-05 | 0.159 | - | - |
| AT2G41790 | 1.24E-05 | 0.159 | - | - |
| AT1G64470 | 1.16E-05 | 0.159 | - | - |
| AT5G58490 | 1.10E-05 | 0.159 | - | - |
| AT4G04910 | 2.98E-05 | 0.16 | - | - |
| AT3G62720 | 2.85E-05 | 0.16 | - | - |
| AT1G30520 | 2.60E-05 | 0.16 | - | - |
| AT1G79920 | 2.41E-05 | 0.16 | - | - |
| AT1G23820 | 2.23E-05 | 0.16 | - | - |
| AT1G32350 | 1.80E-05 | 0.16 | - | - |
| AT3G06110 | 1.73E-05 | 0.16 | - | - |
| AT4G06599 | 1.52E-05 | 0.16 | - | - |
| AT4G33110 | 1.48E-05 | 0.16 | - | - |
| AT1G79560 | 1.43E-05 | 0.16 | - | - |
| AT1G71300 | 1.32E-05 | 0.16 | - | - |
| AT1G26120 | 1.26E-05 | 0.16 | - | - |
| AT5G64900 | 1.16E-05 | 0.16 | - | - |
| AT2G46310 | 1.08E-05 | 0.16 | - | - |
| AT3G53560 | 1.01E-05 | 0.16 | - | - |
| AT3G14610 | 1.01E-05 | 0.16 | - | - |
| AT5G46290 | 2.75E-05 | 0.161 | - | - |
| AT2G24020 | 2.10E-05 | 0.161 | - | - |
| AT2G21730 | 1.98E-05 | 0.161 | - | - |
| AT2G38270 | 1.43E-05 | 0.161 | - | - |
| AT5G54250 | 1.34E-05 | 0.161 | - | - |
| AT1G30860 | 1.12E-05 | 0.161 | - | - |
| AT4G30840 | 1.78E-04 | 0.162 | - | - |
| AT5G03280 | 3.57E-05 | 0.162 | - | - |
| AT5G56670 | 2.82E-05 | 0.162 | - | - |
| AT3G04400 | 2.19E-05 | 0.162 | - | - |
| AT1G31970 | 2.13E-05 | 0.162 | - | - |
| AT2G34810 | 1.75E-05 | 0.162 | - | - |
| AT3G10940 | 1.63E-05 | 0.162 | - | - |
| ATCG00680 | 1.59E-05 | 0.162 | - | - |
| AT3G46640 | 1.44E-05 | 0.162 | - | - |
| AT4G29690 | 1.39E-05 | 0.162 | - | - |
| AT2G27920 | 1.28E-05 | 0.162 | - | - |
| AT1G52820 | 1.19E-05 | 0.162 | - | - |
| AT4G24150 | 1.19E-05 | 0.162 | - | - |
| AT1G70890 | 1.19E-05 | 0.162 | - | - |
| AT5G49530 | 1.01E-05 | 0.162 | - | - |
| AT2G29530 | 2.73E-05 | 0.163 | - | - |
| AT2G36230 | 2.59E-05 | 0.163 | - | - |
| AT5G02710 | 2.14E-05 | 0.163 | - | - |
| AT1G60800 | 1.97E-05 | 0.163 | - | - |
| AT4G33500 | 1.80E-05 | 0.163 | - | - |
| AT3G60950 | 1.71E-05 | 0.163 | - | - |
| AT5G27700 | 1.54E-05 | 0.163 | - | - |
| AT1G17020 | 1.45E-05 | 0.163 | - | - |
| AT5G24120 | 2.28E-05 | 0.164 | - | - |
| AT4G34290 | 2.27E-05 | 0.164 | - | - |
| AT3G60320 | 2.26E-05 | 0.164 | - | - |
| AT5G22060 | 1.80E-05 | 0.164 | - | - |
| AT3G22980 | 1.64E-05 | 0.164 | - | - |
| AT5G61310 | 1.55E-05 | 0.164 | - | - |
| AT1G21480 | 1.28E-05 | 0.164 | - | - |
| AT4G16350 | 1.17E-05 | 0.164 | - | - |
| AT3G19810 | 1.14E-05 | 0.164 | - | - |
| ATCG00810 | 1.02E-05 | 0.164 | - | - |
| AT3G62250 | 3.48E-05 | 0.165 | - | - |
| AT5G23860 | 2.31E-05 | 0.165 | - | - |
| AT1G68800 | 1.68E-05 | 0.165 | - | - |
| AT3G21230 | 1.66E-05 | 0.165 | - | - |
| AT2G46510 | 1.48E-05 | 0.165 | - | - |
| AT4G30660 | 1.36E-05 | 0.165 | - | - |
| AT2G25490 | 1.30E-05 | 0.165 | - | - |
| AT3G18290 | 1.16E-05 | 0.165 | - | - |
| AT4G13360 | 1.14E-05 | 0.165 | - | - |
| AT5G59380 | 1.09E-05 | 0.165 | - | - |
| AT3G21190 | 1.05E-05 | 0.165 | - | - |
| AT4G03960 | 1.05E-05 | 0.165 | - | - |
| AT1G32640 | 2.68E-05 | 0.166 | - | - |
| AT3G57550 | 2.54E-05 | 0.166 | - | - |
| AT4G26450 | 2.24E-05 | 0.166 | - | - |
| AT3G63060 | 2.12E-05 | 0.166 | - | - |
| AT2G35650 | 1.80E-05 | 0.166 | - | - |
| AT3G47640 | 1.25E-05 | 0.166 | - | - |
| AT1G55580 | 1.16E-05 | 0.166 | - | - |
| AT4G32410 | 2.28E-05 | 0.167 | - | - |
| AT5G02050 | 2.17E-05 | 0.167 | - | - |
| AT4G23440 | 2.06E-05 | 0.167 | - | - |
| AT3G46740 | 1.76E-05 | 0.167 | - | - |
| AT5G22630 | 1.72E-05 | 0.167 | - | - |
| AT4G32980 | 1.67E-05 | 0.167 | - | - |
| AT3G04870 | 1.62E-05 | 0.167 | - | - |
| AT3G11910 | 1.37E-05 | 0.167 | - | - |
| AT1G54560 | 1.33E-05 | 0.167 | - | - |
| AT5G56100 | 1.29E-05 | 0.167 | - | - |
| AT4G35640 | 1.28E-05 | 0.167 | - | - |
| AT4G10030 | 1.20E-05 | 0.167 | - | - |
| AT5G37020 | 1.99E-05 | 0.168 | - | - |
| AT2G37870 | 1.86E-05 | 0.168 | - | - |
| AT5G47120 | 1.59E-05 | 0.168 | - | - |
| AT4G27550 | 1.47E-05 | 0.168 | - | - |
| AT1G13560 | 1.39E-05 | 0.168 | - | - |
| AT2G44740 | 1.35E-05 | 0.168 | - | - |
| AT3G43600 | 1.31E-05 | 0.168 | - | - |
| AT2G16790 | 1.30E-05 | 0.168 | - | - |
| AT5G19130 | 1.23E-05 | 0.168 | - | - |
| AT4G36260 | 1.02E-05 | 0.168 | - | - |
| AT1G60600 | 2.94E-05 | 0.169 | - | - |
| AT3G16830 | 2.65E-05 | 0.169 | - | - |
| AT5G25610 | 2.42E-05 | 0.169 | - | - |
| AT5G02290 | 2.41E-05 | 0.169 | - | - |
| AT2G45770 | 2.04E-05 | 0.169 | - | - |
| AT3G48110 | 1.79E-05 | 0.169 | - | - |
| AT3G15670 | 1.75E-05 | 0.169 | - | - |
| AT5G47370 | 1.57E-05 | 0.169 | - | - |
| AT5G24314 | 1.50E-05 | 0.169 | - | - |
| AT2G03200 | 1.36E-05 | 0.169 | - | - |
| AT1G08490 | 1.06E-05 | 0.169 | - | - |
| AT5G52470 | 3.25E-05 | 0.17 | - | - |
| AT5G11480 | 2.91E-05 | 0.17 | - | - |
| AT5G24470 | 2.06E-05 | 0.17 | - | - |
| AT3G45300 | 1.92E-05 | 0.17 | - | - |
| AT4G25440 | 1.53E-05 | 0.17 | - | - |
| AT5G45620 | 1.38E-05 | 0.17 | - | - |
| AT1G76790 | 1.31E-05 | 0.17 | - | - |
| AT1G03890 | 1.25E-05 | 0.17 | - | - |
| AT3G02410 | 1.18E-05 | 0.17 | - | - |
| AT4G09030 | 1.12E-05 | 0.17 | - | - |
| AT3G19160 | 1.02E-05 | 0.17 | - | - |
| AT4G30320 | 2.62E-05 | 0.171 | - | - |
| AT2G44660 | 2.32E-05 | 0.171 | - | - |
| AT1G31817 | 2.01E-05 | 0.171 | - | - |
| AT5G16280 | 1.92E-05 | 0.171 | - | - |
| AT4G33030 | 1.75E-05 | 0.171 | - | - |
| AT3G49700 | 1.21E-05 | 0.171 | - | - |
| AT2G38740 | 1.16E-05 | 0.171 | - | - |
| AT2G29460 | 2.24E-05 | 0.172 | - | - |
| AT5G57530 | 1.80E-05 | 0.172 | - | - |
| AT2G32520 | 1.67E-05 | 0.172 | - | - |
| AT2G43510 | 1.50E-05 | 0.172 | - | - |
| AT4G08160 | 1.40E-05 | 0.172 | - | - |
| AT1G53840 | 1.32E-05 | 0.172 | - | - |
| AT3G21770 | 1.25E-05 | 0.172 | - | - |
| AT5G42620 | 1.05E-05 | 0.172 | - | - |
| AT2G44120 | 2.58E-05 | 0.173 | - | - |
| AT2G26730 | 1.80E-05 | 0.173 | - | - |
| AT5G58590 | 1.58E-05 | 0.173 | - | - |
| AT2G29450 | 1.43E-05 | 0.173 | - | - |
| AT5G18580 | 1.42E-05 | 0.173 | - | - |
| AT4G26080 | 3.38E-05 | 0.174 | - | - |
| AT4G38130 | 2.14E-05 | 0.174 | - | - |
| AT1G72960 | 1.90E-05 | 0.174 | - | - |
| AT5G47930 | 1.75E-05 | 0.174 | - | - |
| AT2G36250 | 1.63E-05 | 0.174 | - | - |
| AT3G54400 | 1.54E-05 | 0.174 | - | - |
| AT4G21810 | 1.42E-05 | 0.174 | - | - |
| AT4G36515 | 1.41E-05 | 0.174 | - | - |
| AT2G07741 | 1.11E-05 | 0.174 | - | - |
| ATCG00065 | 1.02E-05 | 0.174 | - | - |
| AT3G44620 | 1.76E-05 | 0.175 | - | - |
| AT1G21600 | 1.46E-05 | 0.175 | - | - |
| AT5G55740 | 1.24E-05 | 0.175 | - | - |
| AT5G49490 | 1.23E-05 | 0.175 | - | - |
| AT4G00620 | 1.09E-05 | 0.175 | - | - |
| AT3G54940 | 1.83E-05 | 0.176 | - | - |
| AT4G25890 | 1.58E-05 | 0.176 | - | - |
| AT5G22340 | 1.46E-05 | 0.176 | - | - |
| AT1G12244 | 1.00E-05 | 0.176 | - | - |
| AT3G20000 | 2.70E-05 | 0.177 | - | - |
| AT1G02080 | 2.51E-05 | 0.177 | - | - |
| AT5G36160 | 2.33E-05 | 0.177 | - | - |
| AT1G48920 | 1.68E-05 | 0.177 | - | - |
| AT2G46600 | 1.48E-05 | 0.177 | - | - |
| AT5G40720 | 1.48E-05 | 0.177 | - | - |
| AT4G29340 | 1.38E-05 | 0.177 | - | - |
| AT2G16850 | 1.33E-05 | 0.177 | - | - |
| AT5G09500 | 1.25E-05 | 0.177 | - | - |
| AT2G39210 | 1.06E-05 | 0.177 | - | - |
| AT1G12260 | 1.05E-05 | 0.177 | - | - |
| AT1G64390 | 2.43E-05 | 0.178 | - | - |
| AT3G46040 | 2.17E-05 | 0.178 | - | - |
| AT3G44540 | 1.72E-05 | 0.178 | - | - |
| AT3G14990 | 1.62E-05 | 0.178 | - | - |
| AT4G37940 | 1.61E-05 | 0.178 | - | - |
| AT3G09150 | 1.27E-05 | 0.178 | - | - |
| AT1G71695 | 1.13E-05 | 0.178 | - | - |
| AT4G09800 | 2.65E-05 | 0.179 | - | - |
| AT3G48850 | 2.12E-05 | 0.179 | - | - |
| ATCG00710 | 1.65E-05 | 0.179 | - | - |
| AT3G01200 | 1.36E-05 | 0.179 | - | - |
| AT2G24790 | 1.21E-05 | 0.179 | - | - |
| AT1G30360 | 1.13E-05 | 0.179 | - | - |
| AT3G18630 | 1.08E-05 | 0.179 | - | - |
| AT5G63790 | 2.03E-05 | 0.18 | - | - |
| AT3G11200 | 1.75E-05 | 0.18 | - | - |
| AT1G73190 | 1.44E-05 | 0.18 | - | - |
| AT1G48650 | 1.17E-05 | 0.18 | - | - |
| AT3G08770 | 1.09E-05 | 0.18 | - | - |
| AT5G40120 | 1.06E-05 | 0.18 | - | - |
| ATMG01280 | 2.03E-05 | 0.181 | - | - |
| AT2G37600 | 1.81E-05 | 0.181 | - | - |
| AT5G15650 | 1.38E-05 | 0.181 | - | - |
| AT4G37990 | 1.15E-05 | 0.181 | - | - |
| AT1G14720 | 1.14E-05 | 0.181 | - | - |
| AT1G72750 | 1.12E-05 | 0.181 | - | - |
| AT3G48330 | 1.10E-05 | 0.181 | - | - |
| AT4G32590 | 2.09E-05 | 0.182 | - | - |
| AT3G03540 | 1.31E-05 | 0.182 | - | - |
| AT1G12910 | 1.23E-05 | 0.182 | - | - |
| AT3G58510 | 1.18E-05 | 0.182 | - | - |
| AT1G80480 | 1.16E-05 | 0.182 | - | - |
| AT5G06100 | 1.03E-05 | 0.182 | - | - |
| AT5G59870 | 2.47E-05 | 0.183 | - | - |
| AT3G52730 | 2.44E-05 | 0.183 | - | - |
| AT1G27320 | 2.00E-05 | 0.183 | - | - |
| AT3G17170 | 1.95E-05 | 0.183 | - | - |
| AT4G03560 | 1.67E-05 | 0.183 | - | - |
| AT5G10220 | 1.50E-05 | 0.183 | - | - |
| AT1G74540 | 1.49E-05 | 0.183 | - | - |
| AT2G19760 | 1.37E-05 | 0.183 | - | - |
| AT1G68840 | 1.37E-05 | 0.183 | - | - |
| AT1G20850 | 1.26E-05 | 0.183 | - | - |
| AT2G34150 | 1.25E-05 | 0.183 | - | - |
| AT2G46260 | 1.11E-05 | 0.183 | - | - |
| AT1G53030 | 3.14E-05 | 0.184 | - | - |
| AT2G16910 | 2.20E-05 | 0.184 | - | - |
| AT5G49540 | 1.54E-05 | 0.184 | - | - |
| AT1G78860 | 1.15E-05 | 0.184 | - | - |
| AT5G03190 | 1.03E-05 | 0.184 | - | - |
| AT1G74458 | 1.01E-05 | 0.184 | - | - |
| AT1G20010 | 3.24E-05 | 0.185 | - | - |
| AT4G34670 | 1.87E-05 | 0.185 | - | - |
| AT4G07950 | 1.70E-05 | 0.185 | - | - |
| AT3G28180 | 1.60E-05 | 0.185 | - | - |
| AT3G54390 | 1.26E-05 | 0.185 | - | - |
| AT1G01940 | 1.08E-05 | 0.185 | - | - |
| AT5G41685 | 1.04E-05 | 0.185 | - | - |
| AT4G29740 | 1.02E-05 | 0.185 | - | - |
| AT3G11870 | 2.81E-05 | 0.186 | - | - |
| AT1G22770 | 2.17E-05 | 0.186 | - | - |
| AT2G01820 | 1.87E-05 | 0.186 | - | - |
| AT2G39990 | 1.72E-05 | 0.186 | - | - |
| AT5G63580 | 1.63E-05 | 0.186 | - | - |
| AT4G39950 | 1.54E-05 | 0.186 | - | - |
| AT1G11560 | 1.21E-05 | 0.186 | - | - |
| AT2G23220 | 1.04E-05 | 0.186 | - | - |
| AT4G38800 | 1.01E-05 | 0.186 | - | - |
| AT2G38280 | 2.99E-05 | 0.187 | - | - |
| AT5G23190 | 2.74E-05 | 0.187 | - | - |
| AT5G10840 | 2.06E-05 | 0.187 | - | - |
| AT3G54300 | 1.99E-05 | 0.187 | - | - |
| AT1G23380 | 1.98E-05 | 0.187 | - | - |
| AT1G01280 | 1.72E-05 | 0.187 | - | - |
| AT5G45880 | 1.53E-05 | 0.187 | - | - |
| AT2G19170 | 1.37E-05 | 0.187 | - | - |
| AT5G11530 | 1.00E-05 | 0.187 | - | - |
| AT5G20890 | 3.27E-05 | 0.188 | - | - |
| AT2G25625 | 1.95E-05 | 0.188 | - | - |
| AT1G65820 | 1.68E-05 | 0.188 | - | - |
| AT3G48570 | 1.62E-05 | 0.188 | - | - |
| AT4G18100 | 1.36E-05 | 0.188 | - | - |
| AT1G09610 | 1.29E-05 | 0.188 | - | - |
| AT5G48930 | 1.03E-05 | 0.188 | - | - |
| AT1G20160 | 1.01E-05 | 0.188 | - | - |
| AT3G11410 | 4.12E-05 | 0.189 | - | - |
| AT4G10310 | 3.06E-05 | 0.189 | - | - |
| AT3G16920 | 3.02E-05 | 0.189 | - | - |
| AT4G13020 | 2.05E-05 | 0.189 | - | - |
| AT4G26670 | 1.98E-05 | 0.189 | - | - |
| AT1G08450 | 1.56E-05 | 0.189 | - | - |
| AT5G63910 | 1.27E-05 | 0.189 | - | - |
| AT2G22540 | 1.18E-05 | 0.189 | - | - |
| AT3G63210 | 1.16E-05 | 0.189 | - | - |
| AT3G09640 | 1.15E-05 | 0.189 | - | - |
| AT3G27110 | 1.07E-05 | 0.189 | - | - |
| AT4G36050 | 2.31E-05 | 0.19 | - | - |
| AT1G08010 | 1.74E-05 | 0.19 | - | - |
| AT1G55120 | 1.67E-05 | 0.19 | - | - |
| AT3G26810 | 1.49E-05 | 0.19 | - | - |
| AT4G25140 | 1.39E-05 | 0.19 | - | - |
| AT1G27970 | 1.34E-05 | 0.19 | - | - |
| AT3G22740 | 1.17E-05 | 0.19 | - | - |
| AT4G25120 | 4.51E-05 | 0.191 | - | - |
| AT2G16950 | 2.56E-05 | 0.191 | - | - |
| AT5G01360 | 1.94E-05 | 0.191 | - | - |
| AT5G08130 | 1.81E-05 | 0.191 | - | - |
| AT3G44550 | 1.32E-05 | 0.191 | - | - |
| AT4G04870 | 1.17E-05 | 0.191 | - | - |
| AT1G67550 | 3.20E-05 | 0.192 | - | - |
| AT4G36195 | 1.69E-05 | 0.192 | - | - |
| AT4G04720 | 1.47E-05 | 0.192 | - | - |
| AT1G03445 | 1.37E-05 | 0.192 | - | - |
| AT1G68640 | 1.35E-05 | 0.192 | - | - |
| AT1G44100 | 1.10E-05 | 0.192 | - | - |
| AT4G38630 | 3.84E-05 | 0.193 | - | - |
| AT1G16780 | 1.68E-05 | 0.193 | - | - |
| AT5G55760 | 1.51E-05 | 0.193 | - | - |
| AT4G37660 | 1.27E-05 | 0.193 | - | - |
| AT4G13380 | 1.18E-05 | 0.193 | - | - |
| AT5G37530 | 1.09E-05 | 0.193 | - | - |
| AT1G77750 | 2.30E-05 | 0.194 | - | - |
| AT3G15290 | 1.54E-05 | 0.194 | - | - |
| AT4G36520 | 1.47E-05 | 0.194 | - | - |
| AT5G35970 | 1.43E-05 | 0.194 | - | - |
| AT1G31530 | 1.17E-05 | 0.194 | - | - |
| AT2G34470 | 1.10E-05 | 0.194 | - | - |
| AT1G14610 | 1.05E-05 | 0.194 | - | - |
| AT1G18070 | 4.32E-05 | 0.195 | - | - |
| AT1G12800 | 3.50E-05 | 0.195 | - | - |
| AT4G04610 | 2.11E-05 | 0.195 | - | - |
| AT2G44910 | 1.56E-05 | 0.195 | - | - |
| AT5G08740 | 1.10E-05 | 0.195 | - | - |
| AT4G15900 | 2.19E-05 | 0.196 | - | - |
| AT1G04680 | 2.06E-05 | 0.196 | - | - |
| AT5G66690 | 1.75E-05 | 0.196 | - | - |
| AT4G38570 | 1.65E-05 | 0.196 | - | - |
| AT3G23090 | 1.41E-05 | 0.196 | - | - |
| AT5G21170 | 2.92E-05 | 0.197 | - | - |
| AT5G61380 | 2.26E-05 | 0.197 | - | - |
| AT5G60100 | 1.99E-05 | 0.197 | - | - |
| AT2G37630 | 1.54E-05 | 0.197 | - | - |
| AT4G38460 | 1.36E-05 | 0.197 | - | - |
| AT3G01570 | 1.18E-05 | 0.197 | - | - |
| AT3G56840 | 1.17E-05 | 0.197 | - | - |
| AT1G56010 | 2.25E-05 | 0.198 | - | - |
| AT5G40780 | 2.15E-05 | 0.198 | - | - |
| AT1G55000 | 1.82E-05 | 0.198 | - | - |
| AT5G15960 | 1.58E-05 | 0.198 | - | - |
| AT1G69710 | 1.23E-05 | 0.198 | - | - |
| AT1G08960 | 1.16E-05 | 0.198 | - | - |
| AT2G32060 | 2.63E-05 | 0.199 | - | - |
| AT3G52600 | 1.97E-05 | 0.199 | - | - |
| AT5G25460 | 1.95E-05 | 0.199 | - | - |
| AT3G48520 | 1.70E-05 | 0.199 | - | - |
| AT3G44660 | 1.65E-05 | 0.199 | - | - |
| AT5G62220 | 1.45E-05 | 0.199 | - | - |
| AT5G55240 | 1.01E-05 | 0.199 | - | - |
| AT1G51720 | 2.77E-05 | 0.2 | - | - |
| AT3G48610 | 2.38E-05 | 0.2 | - | - |
| AT2G37240 | 1.98E-05 | 0.2 | - | - |
| AT1G16850 | 1.61E-05 | 0.2 | - | - |
| AT5G40020 | 1.36E-05 | 0.2 | - | - |
| AT1G12350 | 1.19E-05 | 0.2 | - | - |
| AT3G05510 | 1.10E-05 | 0.2 | - | - |
| AT3G51840 | 2.90E-05 | 0.201 | - | - |
| AT5G26030 | 1.98E-05 | 0.201 | - | - |
| AT5G66780 | 1.09E-05 | 0.201 | - | - |
| AT3G23490 | 2.05E-05 | 0.202 | - | - |
| AT1G72260 | 1.64E-05 | 0.202 | - | - |
| AT2G16360 | 1.02E-05 | 0.202 | - | - |
| AT4G26130 | 2.86E-05 | 0.203 | - | - |
| AT3G22230 | 2.29E-05 | 0.203 | - | - |
| AT3G11280 | 1.11E-05 | 0.203 | - | - |
| AT1G15340 | 1.10E-05 | 0.203 | - | - |
| AT2G14750 | 1.73E-05 | 0.204 | - | - |
| AT2G34890 | 1.56E-05 | 0.204 | - | - |
| AT4G39620 | 1.51E-05 | 0.204 | - | - |
| AT5G64650 | 1.42E-05 | 0.204 | - | - |
| AT5G04660 | 1.35E-05 | 0.204 | - | - |
| AT5G50680 | 1.31E-05 | 0.204 | - | - |
| AT4G04470 | 1.25E-05 | 0.204 | - | - |
| AT1G66850 | 1.11E-05 | 0.204 | - | - |
| AT1G27370 | 1.09E-05 | 0.204 | - | - |
| AT5G16070 | 2.08E-05 | 0.205 | - | - |
| AT5G07470 | 2.04E-05 | 0.205 | - | - |
| AT4G11640 | 1.66E-05 | 0.205 | - | - |
| AT5G10050 | 1.41E-05 | 0.205 | - | - |
| AT4G28500 | 1.40E-05 | 0.205 | - | - |
| AT5G61420 | 1.28E-05 | 0.205 | - | - |
| AT4G29410 | 2.38E-05 | 0.206 | - | - |
| AT3G11400 | 2.22E-05 | 0.206 | - | - |
| AT5G14730 | 1.61E-05 | 0.206 | - | - |
| AT2G46225 | 1.43E-05 | 0.206 | - | - |
| AT1G77330 | 1.21E-05 | 0.206 | - | - |
| AT4G17750 | 1.02E-05 | 0.206 | - | - |
| AT5G11170 | 2.60E-05 | 0.207 | - | - |
| AT3G29575 | 2.57E-05 | 0.207 | - | - |
| AT1G06950 | 1.55E-05 | 0.207 | - | - |
| AT4G38690 | 1.54E-05 | 0.207 | - | - |
| AT5G13440 | 1.51E-05 | 0.207 | - | - |
| AT2G28100 | 1.25E-05 | 0.207 | - | - |
| AT2G47550 | 1.02E-05 | 0.207 | - | - |
| AT2G27960 | 2.38E-05 | 0.208 | - | - |
| AT1G11190 | 2.24E-05 | 0.208 | - | - |
| AT2G28110 | 1.98E-05 | 0.208 | - | - |
| AT4G30280 | 1.72E-05 | 0.208 | - | - |
| AT4G03190 | 1.36E-05 | 0.208 | - | - |
| AT5G45280 | 1.05E-05 | 0.208 | - | - |
| AT4G08150 | 2.86E-05 | 0.209 | - | - |
| AT4G24570 | 2.20E-05 | 0.209 | - | - |
| AT2G36070 | 1.37E-05 | 0.209 | - | - |
| AT5G02080 | 1.20E-05 | 0.209 | - | - |
| AT4G17650 | 1.10E-05 | 0.209 | - | - |
| AT4G34470 | 1.07E-05 | 0.209 | - | - |
| AT1G74790 | 1.01E-05 | 0.209 | - | - |
| AT1G25410 | 1.01E-05 | 0.209 | - | - |
| AT3G57260 | 2.19E-05 | 0.21 | - | - |
| AT5G23300 | 2.10E-05 | 0.21 | - | - |
| AT3G07270 | 1.62E-05 | 0.21 | - | - |
| AT1G49450 | 1.58E-05 | 0.21 | - | - |
| AT1G06590 | 1.57E-05 | 0.21 | - | - |
| AT1G75230 | 1.38E-05 | 0.21 | - | - |
| AT1G19520 | 1.02E-05 | 0.21 | - | - |
| AT3G54280 | 2.27E-05 | 0.211 | - | - |
| AT3G06980 | 2.09E-05 | 0.211 | - | - |
| AT1G23390 | 1.78E-05 | 0.211 | - | - |
| AT1G32170 | 1.58E-05 | 0.211 | - | - |
| AT3G08610 | 1.34E-05 | 0.211 | - | - |
| AT1G23420 | 1.14E-05 | 0.211 | - | - |
| AT4G33865 | 2.28E-05 | 0.212 | - | - |
| AT1G04200 | 2.25E-05 | 0.212 | - | - |
| AT4G33060 | 2.09E-05 | 0.212 | - | - |
| AT1G12040 | 1.20E-05 | 0.212 | - | - |
| AT5G27130 | 1.15E-05 | 0.212 | - | - |
| AT4G21510 | 1.04E-05 | 0.212 | - | - |
| AT1G69840 | 2.08E-05 | 0.213 | - | - |
| AT5G55400 | 1.73E-05 | 0.213 | - | - |
| AT1G21660 | 1.58E-05 | 0.213 | - | - |
| AT1G31280 | 1.54E-05 | 0.213 | - | - |
| AT4G21470 | 1.33E-05 | 0.213 | - | - |
| AT1G72150 | 1.21E-05 | 0.213 | - | - |
| AT5G59450 | 1.04E-05 | 0.213 | - | - |
| AT2G20580 | 2.30E-05 | 0.214 | - | - |
| AT3G49910 | 2.10E-05 | 0.214 | - | - |
| AT5G07550 | 1.62E-05 | 0.214 | - | - |
| AT2G19400 | 1.31E-05 | 0.214 | - | - |
| AT3G06380 | 1.21E-05 | 0.214 | - | - |
| AT5G60340 | 2.22E-05 | 0.215 | - | - |
| AT4G13610 | 1.43E-05 | 0.215 | - | - |
| AT1G49400 | 1.32E-05 | 0.215 | - | - |
| AT4G04950 | 1.27E-05 | 0.215 | - | - |
| AT3G57490 | 2.92E-05 | 0.216 | - | - |
| AT2G03870 | 2.27E-05 | 0.216 | - | - |
| AT5G03040 | 1.86E-05 | 0.216 | - | - |
| AT5G67370 | 1.76E-05 | 0.216 | - | - |
| AT3G04600 | 1.75E-05 | 0.216 | - | - |
| AT4G02970 | 1.55E-05 | 0.216 | - | - |
| AT3G60140 | 1.37E-05 | 0.216 | - | - |
| AT5G48250 | 1.32E-05 | 0.216 | - | - |
| AT5G50700 | 2.04E-05 | 0.217 | - | - |
| AT4G35080 | 1.60E-05 | 0.217 | - | - |
| AT3G60350 | 1.52E-05 | 0.217 | - | - |
| AT2G45440 | 1.39E-05 | 0.217 | - | - |
| AT4G14690 | 1.12E-05 | 0.217 | - | - |
| AT5G17430 | 1.02E-05 | 0.217 | - | - |
| AT1G18870 | 2.21E-05 | 0.218 | - | - |
| AT3G25220 | 1.71E-05 | 0.218 | - | - |
| AT1G21120 | 1.59E-05 | 0.218 | - | - |
| AT1G72930 | 1.48E-05 | 0.218 | - | - |
| AT2G26070 | 1.37E-05 | 0.218 | - | - |
| AT4G37830 | 1.24E-05 | 0.218 | - | - |
| AT5G61340 | 1.20E-05 | 0.218 | - | - |
| AT2G46790 | 1.08E-05 | 0.218 | - | - |
| AT3G19010 | 1.94E-05 | 0.219 | - | - |
| AT3G48170 | 1.17E-05 | 0.219 | - | - |
| AT3G51040 | 1.17E-05 | 0.219 | - | - |
| AT4G25490 | 1.16E-05 | 0.219 | - | - |
| AT3G10010 | 1.12E-05 | 0.219 | - | - |
| AT1G01530 | 1.03E-05 | 0.219 | - | - |
| AT3G01513 | 1.10E-05 | 0.22 | - | - |
| AT3G51460 | 1.95E-05 | 0.221 | - | - |
| AT5G07460 | 1.58E-05 | 0.221 | - | - |
| AT2G17630 | 1.43E-05 | 0.221 | - | - |
| AT3G17520 | 1.91E-05 | 0.222 | - | - |
| AT3G09680 | 1.54E-05 | 0.222 | - | - |
| AT3G55770 | 1.35E-05 | 0.222 | - | - |
| AT3G50220 | 1.27E-05 | 0.222 | - | - |
| AT2G41260 | 1.12E-05 | 0.222 | - | - |
| AT1G27550 | 1.06E-05 | 0.222 | - | - |
| AT5G57050 | 4.19E-05 | 0.223 | - | - |
| AT1G06570 | 2.38E-05 | 0.223 | - | - |
| AT5G11980 | 1.92E-05 | 0.223 | - | - |
| AT5G56600 | 1.59E-05 | 0.223 | - | - |
| AT1G62290 | 1.38E-05 | 0.223 | - | - |
| AT3G08710 | 1.17E-05 | 0.223 | - | - |
| AT1G06290 | 1.07E-05 | 0.223 | - | - |
| AT3G02450 | 1.05E-05 | 0.223 | - | - |
| AT3G07220 | 3.16E-05 | 0.224 | - | - |
| AT1G54760 | 2.18E-05 | 0.224 | - | - |
| AT1G41830 | 1.93E-05 | 0.224 | - | - |
| AT5G64740 | 1.93E-05 | 0.225 | - | - |
| AT1G07010 | 1.74E-05 | 0.225 | - | - |
| AT3G53130 | 1.72E-05 | 0.225 | - | - |
| AT2G45820 | 1.68E-05 | 0.225 | - | - |
| AT3G53800 | 1.57E-05 | 0.225 | - | - |
| AT3G45030 | 1.51E-05 | 0.225 | - | - |
| AT3G27330 | 1.36E-05 | 0.225 | - | - |
| AT3G06540 | 1.20E-05 | 0.225 | - | - |
| AT2G40080 | 1.07E-05 | 0.225 | - | - |
| AT4G16190 | 2.77E-05 | 0.226 | - | - |
| AT1G15570 | 2.60E-05 | 0.226 | - | - |
| AT5G63420 | 2.33E-05 | 0.226 | - | - |
| AT1G80490 | 2.33E-05 | 0.226 | - | - |
| AT3G18390 | 1.65E-05 | 0.226 | - | - |
| AT2G34650 | 1.37E-05 | 0.226 | - | - |
| AT1G60970 | 1.22E-05 | 0.226 | - | - |
| AT4G36380 | 1.04E-05 | 0.226 | - | - |
| AT3G19980 | 3.78E-05 | 0.227 | - | - |
| AT3G59030 | 2.09E-05 | 0.227 | - | - |
| AT1G61790 | 2.07E-05 | 0.227 | - | - |
| AT2G40590 | 1.85E-05 | 0.227 | - | - |
| AT5G05100 | 1.55E-05 | 0.227 | - | - |
| AT3G57220 | 1.44E-05 | 0.227 | - | - |
| AT4G18730 | 1.41E-05 | 0.227 | - | - |
| AT1G54040 | 1.36E-05 | 0.227 | - | - |
| ATMG00220 | 1.32E-05 | 0.227 | - | - |
| AT1G03880 | 1.23E-05 | 0.227 | - | - |
| AT3G26580 | 1.22E-05 | 0.227 | - | - |
| AT3G60770 | 3.09E-05 | 0.228 | - | - |
| AT1G30580 | 2.71E-05 | 0.228 | - | - |
| AT3G18820 | 2.53E-05 | 0.228 | - | - |
| AT4G15760 | 1.80E-05 | 0.228 | - | - |
| AT3G27660 | 1.64E-05 | 0.228 | - | - |
| AT2G05830 | 1.23E-05 | 0.228 | - | - |
| AT3G29800 | 1.00E-05 | 0.228 | - | - |
| AT3G52140 | 2.08E-05 | 0.229 | - | - |
| AT1G07930 | 1.74E-05 | 0.229 | - | - |
| AT3G61790 | 1.31E-05 | 0.229 | - | - |
| AT1G25380 | 1.23E-05 | 0.229 | - | - |
| AT5G05000 | 1.10E-05 | 0.229 | - | - |
| AT4G29040 | 3.15E-05 | 0.23 | - | - |
| AT5G63890 | 2.78E-05 | 0.23 | - | - |
| AT4G26310 | 1.28E-05 | 0.23 | - | - |
| AT5G21930 | 1.17E-05 | 0.23 | - | - |
| AT5G50210 | 1.10E-05 | 0.23 | - | - |
| AT3G18660 | 2.08E-05 | 0.231 | - | - |
| AT4G30620 | 1.75E-05 | 0.231 | - | - |
| AT1G68620 | 1.32E-05 | 0.231 | - | - |
| AT1G66280 | 1.29E-05 | 0.231 | - | - |
| AT1G22920 | 4.07E-05 | 0.232 | - | - |
| AT2G32220 | 2.10E-05 | 0.232 | - | - |
| AT2G04280 | 1.62E-05 | 0.232 | - | - |
| AT3G03050 | 1.35E-05 | 0.232 | - | - |
| AT5G64050 | 1.23E-05 | 0.232 | - | - |
| AT5G51060 | 1.17E-05 | 0.232 | - | - |
| AT1G75310 | 1.96E-05 | 0.233 | - | - |
| AT1G78490 | 1.71E-05 | 0.233 | - | - |
| AT3G27050 | 1.57E-05 | 0.233 | - | - |
| AT5G07920 | 1.38E-05 | 0.233 | - | - |
| AT5G26120 | 1.28E-05 | 0.233 | - | - |
| AT5G49850 | 1.28E-05 | 0.233 | - | - |
| AT5G02270 | 1.06E-05 | 0.233 | - | - |
| AT3G60600 | 4.19E-05 | 0.234 | - | - |
| AT1G52570 | 2.29E-05 | 0.234 | - | - |
| AT4G37670 | 2.19E-05 | 0.234 | - | - |
| AT5G38710 | 1.79E-05 | 0.234 | - | - |
| AT1G21410 | 1.57E-05 | 0.234 | - | - |
| AT5G49270 | 1.54E-05 | 0.234 | - | - |
| AT1G14230 | 1.50E-05 | 0.234 | - | - |
| AT5G45670 | 1.41E-05 | 0.234 | - | - |
| ATCG00670 | 1.30E-05 | 0.234 | - | - |
| AT1G19050 | 1.23E-05 | 0.234 | - | - |
| AT5G63590 | 1.69E-05 | 0.235 | - | - |
| AT3G62420 | 1.42E-05 | 0.235 | - | - |
| AT4G12650 | 1.39E-05 | 0.235 | - | - |
| AT5G23570 | 1.21E-05 | 0.235 | - | - |
| AT3G18430 | 1.20E-05 | 0.235 | - | - |
| AT3G29200 | 1.02E-05 | 0.235 | - | - |
| AT3G48890 | 1.68E-05 | 0.236 | - | - |
| AT5G28050 | 1.63E-05 | 0.236 | - | - |
| AT1G27520 | 1.28E-05 | 0.236 | - | - |
| AT1G52880 | 1.15E-05 | 0.236 | - | - |
| AT5G45380 | 1.01E-05 | 0.236 | - | - |
| AT2G30810 | 1.68E-05 | 0.237 | - | - |
| AT5G59500 | 1.67E-05 | 0.237 | - | - |
| AT5G07500 | 1.33E-05 | 0.237 | - | - |
| AT1G06090 | 1.15E-05 | 0.237 | - | - |
| AT5G49030 | 2.38E-05 | 0.238 | - | - |
| AT1G04400 | 1.98E-05 | 0.238 | - | - |
| AT3G62120 | 1.86E-05 | 0.238 | - | - |
| AT5G40160 | 1.26E-05 | 0.238 | - | - |
| AT2G17360 | 3.07E-05 | 0.239 | - | - |
| AT1G62360 | 2.11E-05 | 0.239 | - | - |
| AT2G39030 | 1.54E-05 | 0.239 | - | - |
| AT5G15410 | 1.31E-05 | 0.239 | - | - |
| AT1G53800 | 1.15E-05 | 0.239 | - | - |
| AT5G26360 | 2.10E-05 | 0.24 | - | - |
| AT1G71270 | 2.01E-05 | 0.24 | - | - |
| AT4G21990 | 1.48E-05 | 0.24 | - | - |
| AT2G29420 | 1.42E-05 | 0.24 | - | - |
| AT4G27450 | 1.93E-05 | 0.241 | - | - |
| AT1G69490 | 1.82E-05 | 0.241 | - | - |
| AT5G09490 | 1.54E-05 | 0.241 | - | - |
| AT4G13710 | 1.04E-05 | 0.241 | - | - |
| AT2G28040 | 1.03E-05 | 0.241 | - | - |
| AT5G20060 | 1.00E-05 | 0.241 | - | - |
| AT2G23150 | 2.18E-05 | 0.242 | - | - |
| AT3G10590 | 1.48E-05 | 0.242 | - | - |
| AT1G12010 | 1.18E-05 | 0.242 | - | - |
| AT2G32650 | 1.17E-05 | 0.242 | - | - |
| AT4G38470 | 1.01E-05 | 0.242 | - | - |
| AT2G47600 | 1.01E-05 | 0.242 | - | - |
| AT3G49000 | 2.85E-05 | 0.243 | - | - |
| AT4G32040 | 1.64E-05 | 0.243 | - | - |
| AT5G55630 | 1.05E-05 | 0.243 | - | - |
| AT5G53970 | 3.13E-05 | 0.244 | - | - |
| AT4G33670 | 2.16E-05 | 0.244 | - | - |
| AT4G27520 | 1.94E-05 | 0.244 | - | - |
| AT1G23080 | 1.43E-05 | 0.244 | - | - |
| AT2G34690 | 1.41E-05 | 0.244 | - | - |
| AT2G27660 | 1.12E-05 | 0.244 | - | - |
| AT4G18040 | 2.27E-05 | 0.245 | - | - |
| AT3G06200 | 1.92E-05 | 0.245 | - | - |
| AT5G64580 | 1.81E-05 | 0.245 | - | - |
| AT3G11070 | 1.24E-05 | 0.245 | - | - |
| AT2G30520 | 1.17E-05 | 0.245 | - | - |
| AT1G60960 | 1.16E-05 | 0.245 | - | - |
| AT5G14380 | 1.15E-05 | 0.245 | - | - |
| AT5G35220 | 1.08E-05 | 0.245 | - | - |
| AT5G59040 | 1.06E-05 | 0.245 | - | - |
| AT2G39190 | 1.01E-05 | 0.245 | - | - |
| AT3G17609 | 2.70E-05 | 0.246 | - | - |
| AT5G44070 | 2.43E-05 | 0.246 | - | - |
| AT1G26340 | 1.63E-05 | 0.246 | - | - |
| AT4G34160 | 1.62E-05 | 0.246 | - | - |
| AT3G05710 | 1.55E-05 | 0.246 | - | - |
| AT4G39940 | 1.42E-05 | 0.246 | - | - |
| AT2G47320 | 1.03E-05 | 0.246 | - | - |
| AT2G18300 | 1.77E-05 | 0.247 | - | - |
| AT2G02560 | 1.62E-05 | 0.247 | - | - |
| AT5G50360 | 1.30E-05 | 0.247 | - | - |
| AT1G78770 | 2.55E-05 | 0.248 | - | - |
| AT3G21670 | 2.07E-05 | 0.248 | - | - |
| AT1G78720 | 2.02E-05 | 0.248 | - | - |
| AT5G59480 | 1.61E-05 | 0.248 | - | - |
| AT1G47580 | 1.43E-05 | 0.248 | - | - |
| AT1G02800 | 1.33E-05 | 0.248 | - | - |
| AT2G32510 | 1.13E-05 | 0.248 | - | - |
| AT4G30470 | 1.07E-05 | 0.248 | - | - |
| AT1G15960 | 1.04E-05 | 0.248 | - | - |
| AT5G20410 | 1.02E-05 | 0.248 | - | - |
| AT5G23440 | 1.51E-05 | 0.249 | - | - |
| AT3G23410 | 1.19E-05 | 0.249 | - | - |
| AT2G27490 | 1.14E-05 | 0.249 | - | - |
| AT2G01830 | 2.12E-05 | 0.25 | - | - |
| AT1G07430 | 2.00E-05 | 0.25 | - | - |
| AT5G23535 | 1.69E-05 | 0.25 | - | - |
| AT3G08510 | 1.22E-05 | 0.25 | - | - |
| AT3G51880 | 1.07E-05 | 0.25 | - | - |
| AT3G09500 | 2.53E-05 | 0.251 | - | - |
| AT5G61130 | 1.93E-05 | 0.251 | - | - |
| AT3G01420 | 1.92E-05 | 0.251 | - | - |
| AT2G47620 | 1.66E-05 | 0.251 | - | - |
| AT1G21850 | 1.20E-05 | 0.251 | - | - |
| AT1G03850 | 1.10E-05 | 0.251 | - | - |
| AT2G39850 | 1.28E-05 | 0.252 | - | - |
| AT2G45270 | 1.13E-05 | 0.252 | - | - |
| AT3G29330 | 1.05E-05 | 0.252 | - | - |
| AT3G23130 | 1.75E-05 | 0.253 | - | - |
| AT4G12770 | 1.58E-05 | 0.253 | - | - |
| AT1G08980 | 1.53E-05 | 0.253 | - | - |
| AT1G48175 | 1.04E-05 | 0.253 | - | - |
| AT1G69410 | 2.94E-05 | 0.254 | - | - |
| AT5G09870 | 1.81E-05 | 0.254 | - | - |
| AT3G19350 | 1.61E-05 | 0.254 | - | - |
| AT2G28850 | 1.62E-05 | 0.255 | - | - |
| AT4G35630 | 1.25E-05 | 0.255 | - | - |
| AT4G27170 | 1.21E-05 | 0.255 | - | - |
| AT1G07700 | 2.09E-05 | 0.256 | - | - |
| AT2G29350 | 1.71E-05 | 0.256 | - | - |
| AT2G20670 | 1.71E-05 | 0.256 | - | - |
| AT3G01510 | 1.62E-05 | 0.256 | - | - |
| AT5G06060 | 1.45E-05 | 0.256 | - | - |
| AT2G04270 | 1.37E-05 | 0.256 | - | - |
| AT1G32520 | 1.10E-05 | 0.256 | - | - |
| AT1G77640 | 1.07E-05 | 0.256 | - | - |
| AT5G60770 | 1.00E-05 | 0.256 | - | - |
| AT1G20510 | 2.14E-05 | 0.257 | - | - |
| AT2G04400 | 1.84E-05 | 0.257 | - | - |
| AT4G05230 | 1.11E-05 | 0.257 | - | - |
| AT3G10610 | 2.58E-05 | 0.258 | - | - |
| AT1G03870 | 1.93E-05 | 0.258 | - | - |
| AT1G21970 | 1.58E-05 | 0.258 | - | - |
| AT1G01840 | 1.32E-05 | 0.258 | - | - |
| AT4G34000 | 1.20E-05 | 0.258 | - | - |
| AT1G74320 | 1.16E-05 | 0.258 | - | - |
| AT3G18550 | 1.48E-05 | 0.259 | - | - |
| AT5G15350 | 1.41E-05 | 0.259 | - | - |
| AT4G12940 | 1.06E-05 | 0.259 | - | - |
| AT4G14540 | 2.53E-05 | 0.26 | - | - |
| AT1G09830 | 2.23E-05 | 0.26 | - | - |
| AT1G78920 | 1.54E-05 | 0.26 | - | - |
| AT5G13320 | 1.54E-05 | 0.26 | - | - |
| AT1G53165 | 1.25E-05 | 0.26 | - | - |
| AT1G67360 | 1.37E-05 | 0.261 | - | - |
| AT5G04590 | 1.25E-05 | 0.261 | - | - |
| AT4G25820 | 1.77E-05 | 0.262 | - | - |
| AT1G73540 | 1.13E-05 | 0.262 | - | - |
| AT5G19150 | 1.06E-05 | 0.262 | - | - |
| AT5G35620 | 2.49E-05 | 0.263 | - | - |
| AT2G19730 | 2.02E-05 | 0.263 | - | - |
| AT3G10520 | 1.88E-05 | 0.263 | - | - |
| AT4G22200 | 1.79E-05 | 0.263 | - | - |
| AT4G14210 | 1.46E-05 | 0.263 | - | - |
| AT1G18710 | 1.28E-05 | 0.263 | - | - |
| AT4G20850 | 1.78E-05 | 0.264 | - | - |
| AT5G09770 | 1.51E-05 | 0.264 | - | - |
| AT3G22890 | 1.35E-05 | 0.264 | - | - |
| AT1G70940 | 1.30E-05 | 0.264 | - | - |
| AT1G66630 | 1.11E-05 | 0.264 | - | - |
| AT3G11540 | 2.99E-05 | 0.265 | - | - |
| AT1G03950 | 1.79E-05 | 0.265 | - | - |
| AT4G26700 | 1.66E-05 | 0.265 | - | - |
| AT1G17170 | 1.26E-05 | 0.265 | - | - |
| AT4G32770 | 1.22E-05 | 0.265 | - | - |
| AT2G25280 | 1.17E-05 | 0.265 | - | - |
| AT3G57190 | 1.15E-05 | 0.265 | - | - |
| AT1G24530 | 1.08E-05 | 0.265 | - | - |
| AT1G58684 | 2.37E-05 | 0.266 | - | - |
| AT5G01300 | 2.25E-05 | 0.266 | - | - |
| AT1G63290 | 2.18E-05 | 0.266 | - | - |
| AT4G23810 | 2.01E-05 | 0.266 | - | - |
| AT2G30840 | 1.83E-05 | 0.266 | - | - |
| AT2G03760 | 1.57E-05 | 0.266 | - | - |
| AT5G63980 | 1.48E-05 | 0.266 | - | - |
| AT1G32560 | 1.24E-05 | 0.266 | - | - |
| AT4G14680 | 1.23E-05 | 0.266 | - | - |
| AT1G62990 | 2.77E-05 | 0.267 | - | - |
| AT5G59440 | 1.62E-05 | 0.267 | - | - |
| AT4G14960 | 1.56E-05 | 0.267 | - | - |
| AT5G43900 | 1.51E-05 | 0.267 | - | - |
| AT2G04540 | 1.33E-05 | 0.267 | - | - |
| AT3G04070 | 1.27E-05 | 0.267 | - | - |
| AT5G06600 | 1.16E-05 | 0.267 | - | - |
| AT5G45040 | 1.94E-05 | 0.268 | - | - |
| AT4G03205 | 1.37E-05 | 0.268 | - | - |
| AT1G17410 | 1.09E-05 | 0.268 | - | - |
| AT3G27740 | 3.00E-05 | 0.269 | - | - |
| AT4G22920 | 1.11E-05 | 0.269 | - | - |
| AT1G49630 | 1.94E-05 | 0.27 | - | - |
| AT1G32230 | 1.89E-05 | 0.27 | - | - |
| AT5G44790 | 1.64E-05 | 0.27 | - | - |
| AT5G09230 | 1.48E-05 | 0.27 | - | - |
| AT1G17710 | 1.13E-05 | 0.27 | - | - |
| AT2G38860 | 1.06E-05 | 0.27 | - | - |
| AT4G33630 | 2.72E-05 | 0.271 | - | - |
| AT5G57900 | 1.88E-05 | 0.271 | - | - |
| AT3G15660 | 1.74E-05 | 0.271 | - | - |
| AT1G75410 | 1.58E-05 | 0.271 | - | - |
| AT3G23060 | 1.54E-05 | 0.271 | - | - |
| AT5G23790 | 1.37E-05 | 0.271 | - | - |
| AT2G02100 | 1.36E-05 | 0.271 | - | - |
| AT1G32770 | 1.20E-05 | 0.271 | - | - |
| AT1G78680 | 1.12E-05 | 0.271 | - | - |
| AT2G42200 | 1.11E-05 | 0.271 | - | - |
| AT5G51610 | 1.03E-05 | 0.271 | - | - |
| AT3G44750 | 2.47E-05 | 0.272 | - | - |
| AT4G02780 | 2.24E-05 | 0.272 | - | - |
| AT4G27430 | 2.12E-05 | 0.272 | - | - |
| AT5G25220 | 1.97E-05 | 0.272 | - | - |
| AT5G38470 | 1.91E-05 | 0.272 | - | - |
| AT4G16030 | 1.37E-05 | 0.272 | - | - |
| AT3G12490 | 1.31E-05 | 0.272 | - | - |
| AT2G25110 | 1.28E-05 | 0.272 | - | - |
| AT2G07715 | 1.02E-05 | 0.272 | - | - |
| AT3G15170 | 1.18E-05 | 0.273 | - | - |
| AT1G31860 | 3.38E-05 | 0.274 | - | - |
| AT2G20530 | 2.69E-05 | 0.274 | - | - |
| AT1G48830 | 2.50E-05 | 0.274 | - | - |
| AT3G05520 | 2.22E-05 | 0.274 | - | - |
| AT5G66140 | 1.89E-05 | 0.274 | - | - |
| AT4G28470 | 1.47E-05 | 0.274 | - | - |
| AT3G51810 | 1.45E-05 | 0.274 | - | - |
| AT3G28710 | 1.42E-05 | 0.274 | - | - |
| AT3G61110 | 1.77E-05 | 0.275 | - | - |
| AT4G25530 | 1.33E-05 | 0.275 | - | - |
| AT5G50580 | 1.33E-05 | 0.275 | - | - |
| AT4G16700 | 1.52E-05 | 0.276 | - | - |
| AT1G47600 | 1.23E-05 | 0.276 | - | - |
| AT4G01770 | 1.14E-05 | 0.276 | - | - |
| AT3G15940 | 1.11E-05 | 0.276 | - | - |
| AT5G55950 | 1.09E-05 | 0.276 | - | - |
| AT1G20220 | 1.04E-05 | 0.276 | - | - |
| AT4G09040 | 1.87E-05 | 0.277 | - | - |
| AT1G70620 | 1.55E-05 | 0.277 | - | - |
| AT3G09710 | 1.54E-05 | 0.277 | - | - |
| AT5G57090 | 1.46E-05 | 0.277 | - | - |
| AT5G19120 | 1.42E-05 | 0.277 | - | - |
| AT1G80160 | 1.36E-05 | 0.277 | - | - |
| AT1G22490 | 1.10E-05 | 0.277 | - | - |
| AT2G28030 | 1.07E-05 | 0.277 | - | - |
| AT4G02020 | 2.79E-05 | 0.278 | - | - |
| AT2G20490 | 2.43E-05 | 0.278 | - | - |
| AT5G57500 | 1.88E-05 | 0.278 | - | - |
| AT3G16290 | 1.67E-05 | 0.278 | - | - |
| AT2G45210 | 1.11E-05 | 0.278 | - | - |
| AT4G11280 | 2.38E-05 | 0.279 | - | - |
| AT5G06760 | 1.85E-05 | 0.279 | - | - |
| AT5G01310 | 1.73E-05 | 0.279 | - | - |
| AT3G54870 | 1.63E-05 | 0.279 | - | - |
| AT1G45332 | 1.61E-05 | 0.279 | - | - |
| AT5G48010 | 1.47E-05 | 0.279 | - | - |
| AT3G06310 | 1.27E-05 | 0.279 | - | - |
| AT3G21070 | 1.14E-05 | 0.279 | - | - |
| AT5G22940 | 1.12E-05 | 0.279 | - | - |
| AT3G45830 | 1.01E-05 | 0.279 | - | - |
| AT3G56150 | 2.72E-05 | 0.28 | - | - |
| AT1G14240 | 1.61E-05 | 0.28 | - | - |
| AT2G45300 | 1.43E-05 | 0.28 | - | - |
| AT2G21120 | 1.30E-05 | 0.28 | - | - |
| AT5G10870 | 1.09E-05 | 0.28 | - | - |
| AT5G15190 | 1.04E-05 | 0.28 | - | - |
| AT2G45600 | 1.03E-05 | 0.28 | - | - |
| AT1G64660 | 2.16E-05 | 0.281 | - | - |
| AT5G08330 | 1.86E-05 | 0.281 | - | - |
| AT2G02220 | 1.69E-05 | 0.281 | - | - |
| AT3G49620 | 1.09E-05 | 0.281 | - | - |
| AT5G44610 | 1.06E-05 | 0.281 | - | - |
| AT5G04330 | 1.73E-05 | 0.282 | - | - |
| AT3G06350 | 1.63E-05 | 0.282 | - | - |
| AT2G38330 | 1.18E-05 | 0.282 | - | - |
| AT1G62710 | 2.38E-05 | 0.284 | - | - |
| ATMG01170 | 1.45E-05 | 0.284 | - | - |
| AT5G67330 | 1.88E-05 | 0.285 | - | - |
| AT3G08040 | 1.41E-05 | 0.285 | - | - |
| AT5G42870 | 1.35E-05 | 0.285 | - | - |
| AT2G17980 | 1.34E-05 | 0.285 | - | - |
| AT4G14070 | 1.29E-05 | 0.285 | - | - |
| AT5G19750 | 1.25E-05 | 0.285 | - | - |
| AT5G10130 | 1.05E-05 | 0.285 | - | - |
| AT2G02990 | 2.02E-05 | 0.286 | - | - |
| AT5G51570 | 1.98E-05 | 0.286 | - | - |
| AT3G25600 | 1.75E-05 | 0.286 | - | - |
| AT1G48320 | 1.59E-05 | 0.286 | - | - |
| ATCG00800 | 1.31E-05 | 0.286 | - | - |
| AT3G22640 | 1.28E-05 | 0.286 | - | - |
| AT4G01700 | 1.25E-05 | 0.286 | - | - |
| AT4G03010 | 1.08E-05 | 0.286 | - | - |
| AT3G01290 | 2.62E-05 | 0.287 | - | - |
| AT1G63720 | 1.62E-05 | 0.287 | - | - |
| AT4G29430 | 1.44E-05 | 0.287 | - | - |
| AT2G21380 | 1.43E-05 | 0.287 | - | - |
| AT5G20180 | 1.11E-05 | 0.287 | - | - |
| AT1G64780 | 1.05E-05 | 0.287 | - | - |
| AT5G03170 | 2.46E-05 | 0.288 | - | - |
| AT4G09960 | 1.66E-05 | 0.288 | - | - |
| AT1G70700 | 1.16E-05 | 0.288 | - | - |
| AT3G55590 | 1.15E-05 | 0.288 | - | - |
| AT4G25090 | 1.07E-05 | 0.288 | - | - |
| AT4G16780 | 1.58E-05 | 0.289 | - | - |
| AT2G34680 | 1.45E-05 | 0.289 | - | - |
| AT5G12010 | 1.44E-05 | 0.289 | - | - |
| AT3G55605 | 1.43E-05 | 0.289 | - | - |
| AT5G54380 | 1.16E-05 | 0.289 | - | - |
| AT5G59240 | 2.36E-05 | 0.29 | - | - |
| AT4G31480 | 2.14E-05 | 0.29 | - | - |
| AT1G75680 | 2.11E-05 | 0.29 | - | - |
| AT4G02290 | 1.91E-05 | 0.29 | - | - |
| AT1G27510 | 1.76E-05 | 0.29 | - | - |
| AT5G10140 | 1.60E-05 | 0.29 | - | - |
| AT4G36580 | 1.57E-05 | 0.29 | - | - |
| AT2G28380 | 1.24E-05 | 0.29 | - | - |
| AT3G43430 | 1.17E-05 | 0.29 | - | - |
| AT5G58760 | 1.03E-05 | 0.29 | - | - |
| AT5G13210 | 1.01E-05 | 0.29 | - | - |
| AT1G60880 | 1.01E-05 | 0.29 | - | - |
| AT3G02560 | 2.18E-05 | 0.291 | - | - |
| AT1G72520 | 2.00E-05 | 0.291 | - | - |
| AT1G76490 | 1.88E-05 | 0.291 | - | - |
| AT3G11980 | 1.77E-05 | 0.291 | - | - |
| AT3G46100 | 1.60E-05 | 0.291 | - | - |
| AT5G24270 | 1.43E-05 | 0.291 | - | - |
| AT2G25760 | 1.47E-05 | 0.292 | - | - |
| AT4G27370 | 1.20E-05 | 0.292 | - | - |
| AT1G31480 | 1.08E-05 | 0.292 | - | - |
| AT3G06455 | 1.06E-05 | 0.292 | - | - |
| AT1G60780 | 1.04E-05 | 0.292 | - | - |
| AT5G19620 | 1.03E-05 | 0.292 | - | - |
| AT4G33880 | 1.03E-05 | 0.292 | - | - |
| AT5G05960 | 1.00E-05 | 0.292 | - | - |
| AT3G06340 | 1.72E-05 | 0.293 | - | - |
| AT4G29390 | 1.48E-05 | 0.293 | - | - |
| AT5G67070 | 1.39E-05 | 0.293 | - | - |
| AT3G28715 | 1.38E-05 | 0.293 | - | - |
| AT2G21790 | 5.29E-05 | 0.294 | - | - |
| AT2G22290 | 1.98E-05 | 0.294 | - | - |
| AT4G03510 | 1.82E-05 | 0.294 | - | - |
| AT3G51520 | 1.71E-05 | 0.294 | - | - |
| AT2G19750 | 1.31E-05 | 0.294 | - | - |
| AT1G12110 | 1.15E-05 | 0.294 | - | - |
| AT3G10390 | 1.13E-05 | 0.294 | - | - |
| AT4G00480 | 1.08E-05 | 0.294 | - | - |
| AT2G14210 | 1.01E-05 | 0.294 | - | - |
| AT1G49760 | 2.44E-05 | 0.295 | - | - |
| AT1G04170 | 2.09E-05 | 0.295 | - | - |
| AT2G39290 | 1.56E-05 | 0.295 | - | - |
| AT4G23620 | 1.17E-05 | 0.295 | - | - |
| AT1G17190 | 1.10E-05 | 0.295 | - | - |
| AT5G14640 | 1.08E-05 | 0.295 | - | - |
| AT4G08170 | 1.06E-05 | 0.295 | - | - |
| AT5G23740 | 2.75E-05 | 0.296 | - | - |
| AT1G49010 | 2.35E-05 | 0.296 | - | - |
| AT5G45160 | 1.98E-05 | 0.296 | - | - |
| AT4G37520 | 1.45E-05 | 0.296 | - | - |
| AT2G44790 | 1.34E-05 | 0.296 | - | - |
| AT5G15180 | 1.05E-05 | 0.296 | - | - |
| AT1G19180 | 2.35E-05 | 0.297 | - | - |
| AT2G22230 | 1.84E-05 | 0.297 | - | - |
| AT3G29160 | 1.33E-05 | 0.297 | - | - |
| AT1G53400 | 1.30E-05 | 0.297 | - | - |
| AT5G39580 | 1.23E-05 | 0.297 | - | - |
| AT2G31530 | 1.19E-05 | 0.297 | - | - |
| AT5G43700 | 1.17E-05 | 0.297 | - | - |
| AT1G22160 | 1.00E-05 | 0.297 | - | - |
| AT1G24280 | 2.40E-05 | 0.298 | - | - |
| AT5G13280 | 1.87E-05 | 0.298 | - | - |
| AT1G14250 | 1.14E-05 | 0.298 | - | - |
| AT3G23750 | 1.13E-05 | 0.298 | - | - |
| AT4G36420 | 1.08E-05 | 0.298 | - | - |
| AT3G23580 | 2.70E-05 | 0.299 | - | - |
| AT4G26970 | 2.49E-05 | 0.299 | - | - |
| AT2G05790 | 2.13E-05 | 0.299 | - | - |
| AT4G00810 | 1.76E-05 | 0.299 | - | - |
| AT1G09020 | 1.17E-05 | 0.299 | - | - |
| AT4G32880 | 1.88E-05 | 0.3 | - | - |
| AT2G36720 | 1.65E-05 | 0.3 | - | - |
| AT3G07040 | 1.28E-05 | 0.3 | - | - |
| AT3G62830 | 1.27E-05 | 0.3 | - | - |
| AT2G23130 | 1.26E-05 | 0.3 | - | - |
| AT1G48610 | 1.05E-05 | 0.3 | - | - |
| AT2G22670 | 1.41E-05 | 0.301 | - | - |
| AT2G46170 | 1.18E-05 | 0.301 | - | - |
| AT4G11570 | 1.16E-05 | 0.301 | - | - |
| AT1G27730 | 2.60E-05 | 0.302 | - | - |
| AT5G42480 | 1.93E-05 | 0.302 | - | - |
| AT5G20510 | 1.93E-05 | 0.302 | - | - |
| AT1G31220 | 1.51E-05 | 0.303 | - | - |
| AT4G09760 | 1.37E-05 | 0.303 | - | - |
| AT2G25610 | 1.36E-05 | 0.303 | - | - |
| AT2G35070 | 1.16E-05 | 0.303 | - | - |
| AT1G73010 | 1.14E-05 | 0.303 | - | - |
| AT2G01530 | 1.06E-05 | 0.303 | - | - |
| AT1G74510 | 1.83E-05 | 0.304 | - | - |
| AT3G28460 | 1.60E-05 | 0.304 | - | - |
| AT2G02050 | 1.88E-05 | 0.305 | - | - |
| AT1G71030 | 1.28E-05 | 0.305 | - | - |
| AT1G75630 | 2.59E-05 | 0.306 | - | - |
| AT4G29810 | 1.47E-05 | 0.306 | - | - |
| AT3G48460 | 1.37E-05 | 0.306 | - | - |
| AT1G67120 | 1.30E-05 | 0.306 | - | - |
| AT1G64090 | 1.24E-05 | 0.306 | - | - |
| AT2G45750 | 1.09E-05 | 0.306 | - | - |
| AT4G04330 | 1.03E-05 | 0.306 | - | - |
| AT1G14410 | 1.30E-05 | 0.307 | - | - |
| AT3G28345 | 1.08E-05 | 0.307 | - | - |
| AT3G02520 | 1.06E-05 | 0.307 | - | - |
| AT5G47090 | 1.04E-05 | 0.307 | - | - |
| AT1G16410 | 1.36E-05 | 0.308 | - | - |
| AT1G73350 | 1.19E-05 | 0.308 | - | - |
| AT5G13170 | 1.15E-05 | 0.308 | - | - |
| AT2G35300 | 1.04E-05 | 0.308 | - | - |
| AT5G35735 | 2.01E-05 | 0.309 | - | - |
| AT1G10030 | 1.89E-05 | 0.309 | - | - |
| AT1G13950 | 1.58E-05 | 0.309 | - | - |
| AT2G37770 | 1.41E-05 | 0.309 | - | - |
| AT4G30960 | 1.21E-05 | 0.309 | - | - |
| AT5G34930 | 1.17E-05 | 0.309 | - | - |
| AT2G39220 | 1.09E-05 | 0.309 | - | - |
| AT5G03020 | 1.04E-05 | 0.309 | - | - |
| AT3G50260 | 2.49E-05 | 0.31 | - | - |
| AT1G51510 | 2.28E-05 | 0.31 | - | - |
| AT4G03350 | 1.13E-05 | 0.31 | - | - |
| AT2G42580 | 2.33E-05 | 0.311 | - | - |
| AT1G63100 | 1.54E-05 | 0.311 | - | - |
| AT4G37760 | 1.51E-05 | 0.311 | - | - |
| AT1G02850 | 1.48E-05 | 0.311 | - | - |
| AT1G45230 | 1.39E-05 | 0.311 | - | - |
| AT3G13235 | 1.34E-05 | 0.311 | - | - |
| AT1G33800 | 1.34E-05 | 0.311 | - | - |
| AT3G20680 | 1.09E-05 | 0.311 | - | - |
| AT3G25500 | 1.01E-05 | 0.311 | - | - |
| AT2G40170 | 1.93E-05 | 0.312 | - | - |
| AT1G58030 | 1.75E-05 | 0.312 | - | - |
| AT2G34770 | 1.53E-05 | 0.312 | - | - |
| AT1G28490 | 1.53E-05 | 0.312 | - | - |
| AT1G79010 | 1.40E-05 | 0.312 | - | - |
| AT5G04760 | 1.20E-05 | 0.312 | - | - |
| AT2G04750 | 1.18E-05 | 0.312 | - | - |
| AT3G19960 | 1.18E-05 | 0.312 | - | - |
| AT4G30570 | 1.07E-05 | 0.312 | - | - |
| AT4G32940 | 2.35E-05 | 0.313 | - | - |
| AT4G36020 | 1.49E-05 | 0.313 | - | - |
| AT2G38810 | 2.66E-05 | 0.314 | - | - |
| AT1G01910 | 2.17E-05 | 0.314 | - | - |
| AT4G14710 | 1.49E-05 | 0.314 | - | - |
| AT2G17310 | 1.28E-05 | 0.314 | - | - |
| AT5G64030 | 1.11E-05 | 0.314 | - | - |
| AT3G02210 | 1.05E-05 | 0.314 | - | - |
| AT1G74260 | 1.96E-05 | 0.315 | - | - |
| AT5G04180 | 1.38E-05 | 0.315 | - | - |
| ATCG00780 | 1.19E-05 | 0.315 | - | - |
| AT3G28470 | 1.14E-05 | 0.315 | - | - |
| AT4G20890 | 2.59E-05 | 0.316 | - | - |
| ATCG00130 | 1.37E-05 | 0.316 | - | - |
| AT5G55810 | 1.34E-05 | 0.316 | - | - |
| AT2G23190 | 1.22E-05 | 0.316 | - | - |
| AT1G02980 | 1.21E-05 | 0.316 | - | - |
| AT1G16340 | 1.11E-05 | 0.316 | - | - |
| AT1G54100 | 4.14E-05 | 0.317 | - | - |
| AT3G03960 | 2.11E-05 | 0.317 | - | - |
| AT5G04920 | 2.02E-05 | 0.317 | - | - |
| AT1G58380 | 1.59E-05 | 0.317 | - | - |
| AT4G33220 | 1.53E-05 | 0.317 | - | - |
| AT3G06510 | 1.38E-05 | 0.317 | - | - |
| AT5G61820 | 1.26E-05 | 0.317 | - | - |
| AT1G08260 | 4.01E-05 | 0.318 | - | - |
| AT2G13650 | 1.20E-05 | 0.318 | - | - |
| AT3G28900 | 1.93E-05 | 0.319 | - | - |
| AT1G01960 | 1.57E-05 | 0.319 | - | - |
| AT1G71750 | 1.33E-05 | 0.319 | - | - |
| AT5G25630 | 1.32E-05 | 0.319 | - | - |
| AT5G43470 | 1.23E-05 | 0.319 | - | - |
| AT2G04850 | 1.05E-05 | 0.319 | - | - |
| AT4G26900 | 2.56E-05 | 0.32 | - | - |
| AT1G15520 | 1.67E-05 | 0.32 | - | - |
| AT3G28500 | 1.46E-05 | 0.32 | - | - |
| AT1G80820 | 1.40E-05 | 0.321 | - | - |
| AT2G32710 | 1.15E-05 | 0.321 | - | - |
| AT5G01990 | 1.06E-05 | 0.321 | - | - |
| AT2G39390 | 3.03E-05 | 0.322 | - | - |
| AT4G16143 | 1.81E-05 | 0.322 | - | - |
| AT5G44560 | 1.12E-05 | 0.322 | - | - |
| AT5G09970 | 1.03E-05 | 0.322 | - | - |
| AT1G52460 | 1.00E-05 | 0.322 | - | - |
| AT4G08040 | 1.00E-05 | 0.323 | - | - |
| AT2G34380 | 1.80E-05 | 0.324 | - | - |
| AT4G34850 | 1.72E-05 | 0.324 | - | - |
| AT4G31720 | 1.71E-05 | 0.324 | - | - |
| AT4G33930 | 1.58E-05 | 0.324 | - | - |
| AT5G47320 | 1.36E-05 | 0.324 | - | - |
| AT2G39660 | 1.20E-05 | 0.324 | - | - |
| AT1G71780 | 1.06E-05 | 0.324 | - | - |
| AT2G26560 | 2.49E-05 | 0.325 | - | - |
| AT4G15130 | 1.54E-05 | 0.325 | - | - |
| AT4G17770 | 1.46E-05 | 0.325 | - | - |
| AT3G57540 | 1.07E-05 | 0.325 | - | - |
| AT2G28890 | 1.48E-05 | 0.326 | - | - |
| AT4G28570 | 1.34E-05 | 0.326 | - | - |
| AT5G52400 | 1.15E-05 | 0.326 | - | - |
| AT4G04860 | 1.01E-05 | 0.326 | - | - |
| AT3G46090 | 2.12E-05 | 0.327 | - | - |
| AT3G52155 | 1.48E-05 | 0.327 | - | - |
| AT2G19680 | 1.26E-05 | 0.327 | - | - |
| AT1G22070 | 1.26E-05 | 0.328 | - | - |
| AT2G14580 | 1.20E-05 | 0.328 | - | - |
| AT5G53400 | 1.18E-05 | 0.328 | - | - |
| AT5G02810 | 1.04E-05 | 0.328 | - | - |
| AT5G35750 | 2.02E-05 | 0.329 | - | - |
| AT1G30490 | 1.50E-05 | 0.329 | - | - |
| AT5G51070 | 1.54E-05 | 0.33 | - | - |
| AT5G03660 | 1.54E-05 | 0.331 | - | - |
| AT4G01750 | 1.26E-05 | 0.331 | - | - |
| AT1G48600 | 1.07E-05 | 0.331 | - | - |
| AT5G44140 | 1.65E-05 | 0.332 | - | - |
| AT5G07420 | 1.47E-05 | 0.332 | - | - |
| AT3G24140 | 1.26E-05 | 0.332 | - | - |
| AT5G57860 | 1.22E-05 | 0.332 | - | - |
| AT5G01970 | 1.13E-05 | 0.332 | - | - |
| AT2G45150 | 1.10E-05 | 0.332 | - | - |
| AT2G15400 | 2.56E-05 | 0.333 | - | - |
| AT4G22910 | 1.45E-05 | 0.333 | - | - |
| AT2G21045 | 1.09E-05 | 0.333 | - | - |
| ATCG00180 | 3.80E-05 | 0.334 | - | - |
| AT1G02970 | 2.84E-05 | 0.334 | - | - |
| AT5G35930 | 2.79E-05 | 0.334 | - | - |
| AT2G38620 | 2.62E-05 | 0.334 | - | - |
| AT5G58890 | 1.54E-05 | 0.334 | - | - |
| AT3G28230 | 1.52E-05 | 0.334 | - | - |
| AT5G60980 | 1.23E-05 | 0.334 | - | - |
| AT1G69690 | 1.16E-05 | 0.334 | - | - |
| AT5G13490 | 1.10E-05 | 0.334 | - | - |
| AT5G50960 | 1.03E-05 | 0.334 | - | - |
| AT4G34620 | 1.94E-05 | 0.335 | - | - |
| AT3G14640 | 1.85E-05 | 0.335 | - | - |
| AT5G53450 | 1.55E-05 | 0.335 | - | - |
| AT1G12140 | 1.22E-05 | 0.335 | - | - |
| AT1G10510 | 1.60E-05 | 0.336 | - | - |
| AT5G58430 | 1.49E-05 | 0.336 | - | - |
| AT5G13300 | 1.21E-05 | 0.336 | - | - |
| AT1G65440 | 1.21E-05 | 0.336 | - | - |
| AT4G26555 | 1.19E-05 | 0.336 | - | - |
| AT1G48000 | 2.15E-05 | 0.337 | - | - |
| AT1G01470 | 1.85E-05 | 0.337 | - | - |
| AT3G44300 | 1.37E-05 | 0.337 | - | - |
| AT5G67520 | 1.37E-05 | 0.337 | - | - |
| AT1G49880 | 1.11E-05 | 0.337 | - | - |
| AT5G55130 | 1.05E-05 | 0.337 | - | - |
| AT3G16857 | 1.02E-05 | 0.337 | - | - |
| AT3G09910 | 1.01E-05 | 0.337 | - | - |
| AT1G31880 | 1.39E-05 | 0.338 | - | - |
| AT1G50200 | 1.20E-05 | 0.338 | - | - |
| AT1G01970 | 1.13E-05 | 0.338 | - | - |
| AT4G08140 | 1.11E-05 | 0.338 | - | - |
| AT2G19470 | 1.06E-05 | 0.338 | - | - |
| AT3G60510 | 1.01E-05 | 0.338 | - | - |
| AT5G02610 | 2.64E-05 | 0.339 | - | - |
| AT1G53580 | 2.28E-05 | 0.339 | - | - |
| AT2G24260 | 1.36E-05 | 0.339 | - | - |
| AT4G31730 | 1.17E-05 | 0.339 | - | - |
| AT1G74890 | 1.03E-05 | 0.339 | - | - |
| AT1G62020 | 1.98E-05 | 0.34 | - | - |
| AT4G34740 | 1.78E-05 | 0.34 | - | - |
| AT2G25930 | 1.72E-05 | 0.34 | - | - |
| AT1G49770 | 1.55E-05 | 0.34 | - | - |
| AT5G42050 | 1.89E-05 | 0.341 | - | - |
| AT3G15210 | 1.83E-05 | 0.341 | - | - |
| AT5G48460 | 1.25E-05 | 0.341 | - | - |
| AT2G26400 | 1.42E-05 | 0.342 | - | - |
| AT3G18410 | 1.10E-05 | 0.342 | - | - |
| AT3G59040 | 1.05E-05 | 0.342 | - | - |
| AT1G71820 | 2.11E-05 | 0.343 | - | - |
| AT4G35050 | 1.63E-05 | 0.343 | - | - |
| AT2G40780 | 1.28E-05 | 0.343 | - | - |
| AT5G38550 | 1.25E-05 | 0.343 | - | - |
| AT3G01310 | 1.25E-05 | 0.343 | - | - |
| AT2G38400 | 2.04E-05 | 0.344 | - | - |
| AT4G21060 | 1.27E-05 | 0.344 | - | - |
| AT3G59220 | 1.22E-05 | 0.344 | - | - |
| AT1G19700 | 1.10E-05 | 0.344 | - | - |
| AT1G63700 | 1.09E-05 | 0.344 | - | - |
| AT4G23690 | 1.01E-05 | 0.344 | - | - |
| AT5G44840 | 1.39E-05 | 0.345 | - | - |
| AT4G38190 | 1.24E-05 | 0.345 | - | - |
| AT1G59870 | 1.40E-05 | 0.346 | - | - |
| AT3G50970 | 1.39E-05 | 0.346 | - | - |
| AT4G14910 | 1.26E-05 | 0.346 | - | - |
| AT3G03120 | 1.05E-05 | 0.346 | - | - |
| AT3G12110 | 2.00E-05 | 0.347 | - | - |
| AT3G56190 | 1.21E-05 | 0.347 | - | - |
| AT1G52560 | 1.14E-05 | 0.347 | - | - |
| AT1G54570 | 1.04E-05 | 0.347 | - | - |
| AT1G35490 | 2.19E-05 | 0.348 | - | - |
| AT3G27280 | 1.94E-05 | 0.348 | - | - |
| AT5G13190 | 1.81E-05 | 0.348 | - | - |
| AT5G60690 | 1.80E-05 | 0.348 | - | - |
| AT5G59170 | 1.53E-05 | 0.348 | - | - |
| AT4G38225 | 1.47E-05 | 0.348 | - | - |
| AT1G09970 | 1.38E-05 | 0.348 | - | - |
| AT1G07670 | 1.37E-05 | 0.348 | - | - |
| AT5G48900 | 1.04E-05 | 0.348 | - | - |
| AT2G41880 | 1.97E-05 | 0.349 | - | - |
| AT1G47960 | 1.44E-05 | 0.349 | - | - |
| AT1G78240 | 1.23E-05 | 0.349 | - | - |
| AT5G62360 | 1.20E-05 | 0.349 | - | - |
| AT1G01340 | 1.20E-05 | 0.349 | - | - |
| AT2G22360 | 1.01E-05 | 0.349 | - | - |
| AT5G16090 | 1.43E-05 | 0.35 | - | - |
| AT1G09350 | 1.40E-05 | 0.35 | - | - |
| AT3G13784 | 1.37E-05 | 0.35 | - | - |
| AT1G75910 | 1.28E-05 | 0.35 | - | - |
| AT1G19870 | 1.25E-05 | 0.35 | - | - |
| AT5G42150 | 1.09E-05 | 0.35 | - | - |
| AT5G43850 | 2.07E-05 | 0.351 | - | - |
| AT4G13720 | 1.82E-05 | 0.351 | - | - |
| AT1G26130 | 1.47E-05 | 0.351 | - | - |
| AT4G20870 | 1.44E-05 | 0.351 | - | - |
| AT2G18540 | 1.36E-05 | 0.351 | - | - |
| AT1G32450 | 1.03E-05 | 0.351 | - | - |
| AT1G72680 | 1.66E-05 | 0.352 | - | - |
| AT1G31430 | 1.30E-05 | 0.352 | - | - |
| AT3G03900 | 1.19E-05 | 0.352 | - | - |
| AT1G30700 | 1.42E-05 | 0.353 | - | - |
| AT3G55620 | 1.05E-05 | 0.353 | - | - |
| AT4G29190 | 1.52E-05 | 0.354 | - | - |
| AT3G04610 | 1.38E-05 | 0.354 | - | - |
| AT4G14560 | 1.29E-05 | 0.354 | - | - |
| AT4G31900 | 1.25E-05 | 0.354 | - | - |
| AT1G09440 | 1.16E-05 | 0.354 | - | - |
| AT3G07700 | 1.13E-05 | 0.354 | - | - |
| AT4G39510 | 1.07E-05 | 0.354 | - | - |
| AT4G18570 | 1.06E-05 | 0.354 | - | - |
| AT1G36390 | 2.28E-05 | 0.355 | - | - |
| AT5G62690 | 1.54E-05 | 0.355 | - | - |
| AT1G74800 | 1.31E-05 | 0.355 | - | - |
| AT3G22170 | 1.19E-05 | 0.355 | - | - |
| ATCG00840 | 1.14E-05 | 0.355 | - | - |
| AT3G42550 | 1.71E-05 | 0.357 | - | - |
| AT2G11810 | 1.24E-05 | 0.357 | - | - |
| AT5G66110 | 1.17E-05 | 0.357 | - | - |
| AT2G26660 | 1.13E-05 | 0.357 | - | - |
| AT2G34630 | 1.00E-05 | 0.357 | - | - |
| AT1G68000 | 2.15E-05 | 0.358 | - | - |
| AT4G36400 | 1.05E-05 | 0.358 | - | - |
| AT2G25940 | 1.01E-05 | 0.358 | - | - |
| AT3G19580 | 2.23E-05 | 0.359 | - | - |
| AT3G05040 | 2.05E-05 | 0.359 | - | - |
| AT5G55856 | 1.95E-05 | 0.359 | - | - |
| AT5G55230 | 1.69E-05 | 0.359 | - | - |
| AT3G63080 | 1.64E-05 | 0.359 | - | - |
| AT3G54950 | 1.62E-05 | 0.359 | - | - |
| AT4G23850 | 1.29E-05 | 0.359 | - | - |
| AT3G27360 | 1.09E-05 | 0.359 | - | - |
| AT3G04920 | 2.19E-05 | 0.36 | - | - |
| AT3G10300 | 1.22E-05 | 0.36 | - | - |
| AT4G19030 | 4.74E-05 | 0.361 | - | - |
| AT1G29310 | 2.03E-05 | 0.361 | - | - |
| AT5G13710 | 1.38E-05 | 0.361 | - | - |
| AT4G35300 | 1.05E-05 | 0.361 | - | - |
| AT1G10070 | 2.63E-05 | 0.362 | - | - |
| AT3G54640 | 1.90E-05 | 0.362 | - | - |
| AT5G27600 | 1.23E-05 | 0.363 | - | - |
| AT3G27060 | 3.20E-05 | 0.364 | - | - |
| AT2G22500 | 2.52E-05 | 0.364 | - | - |
| AT5G48710 | 1.94E-05 | 0.364 | - | - |
| AT2G07340 | 1.82E-05 | 0.364 | - | - |
| AT3G50060 | 1.63E-05 | 0.364 | - | - |
| AT1G30260 | 1.27E-05 | 0.364 | - | - |
| AT1G29250 | 1.80E-05 | 0.365 | - | - |
| AT1G11755 | 1.36E-05 | 0.365 | - | - |
| AT5G64210 | 1.20E-05 | 0.365 | - | - |
| AT1G75670 | 1.17E-05 | 0.365 | - | - |
| AT2G39900 | 1.07E-05 | 0.365 | - | - |
| AT5G12250 | 1.91E-05 | 0.366 | - | - |
| AT2G25170 | 1.58E-05 | 0.366 | - | - |
| AT3G53490 | 1.11E-05 | 0.366 | - | - |
| AT2G16060 | 1.55E-05 | 0.367 | - | - |
| AT1G70320 | 1.15E-05 | 0.367 | - | - |
| AT2G27120 | 3.99E-05 | 0.368 | - | - |
| AT5G02310 | 2.42E-05 | 0.368 | - | - |
| AT1G16190 | 1.45E-05 | 0.368 | - | - |
| AT4G15610 | 1.31E-05 | 0.368 | - | - |
| AT5G13530 | 1.29E-05 | 0.368 | - | - |
| AT2G26410 | 1.27E-05 | 0.368 | - | - |
| AT2G47570 | 1.18E-05 | 0.368 | - | - |
| AT4G09990 | 1.10E-05 | 0.368 | - | - |
| AT4G37050 | 1.07E-05 | 0.368 | - | - |
| AT2G35690 | 1.41E-05 | 0.369 | - | - |
| AT5G58120 | 1.27E-05 | 0.369 | - | - |
| AT1G63910 | 1.09E-05 | 0.369 | - | - |
| AT5G46860 | 1.95E-05 | 0.37 | - | - |
| AT3G50530 | 1.27E-05 | 0.37 | - | - |
| AT2G32850 | 1.23E-05 | 0.37 | - | - |
| AT3G48030 | 1.09E-05 | 0.37 | - | - |
| AT1G01040 | 2.23E-05 | 0.371 | - | - |
| AT2G47030 | 2.19E-05 | 0.371 | - | - |
| AT1G23870 | 1.96E-05 | 0.371 | - | - |
| AT1G51490 | 1.00E-05 | 0.371 | - | - |
| AT1G63660 | 4.24E-05 | 0.372 | - | - |
| AT4G29780 | 1.77E-05 | 0.372 | - | - |
| AT3G48680 | 1.22E-05 | 0.372 | - | - |
| AT4G27080 | 1.05E-05 | 0.372 | - | - |
| ATCG00170 | 4.08E-05 | 0.373 | - | - |
| AT3G13445 | 2.27E-05 | 0.373 | - | - |
| AT1G20090 | 1.82E-05 | 0.373 | - | - |
| AT5G48140 | 1.49E-05 | 0.374 | - | - |
| AT3G22620 | 1.23E-05 | 0.374 | - | - |
| AT4G02260 | 1.13E-05 | 0.374 | - | - |
| AT5G04040 | 3.51E-05 | 0.375 | - | - |
| AT4G13730 | 1.46E-05 | 0.375 | - | - |
| AT5G02150 | 1.38E-05 | 0.375 | - | - |
| AT1G61580 | 2.62E-05 | 0.376 | - | - |
| AT5G59820 | 2.50E-05 | 0.376 | - | - |
| AT3G01660 | 1.21E-05 | 0.376 | - | - |
| AT3G15840 | 2.61E-05 | 0.377 | - | - |
| AT3G13320 | 2.20E-05 | 0.377 | - | - |
| AT4G09350 | 1.62E-05 | 0.377 | - | - |
| AT1G28320 | 1.43E-05 | 0.377 | - | - |
| AT3G33520 | 1.35E-05 | 0.377 | - | - |
| AT2G35940 | 2.19E-05 | 0.378 | - | - |
| AT1G55320 | 1.57E-05 | 0.378 | - | - |
| AT4G23650 | 1.16E-05 | 0.378 | - | - |
| AT1G72830 | 1.05E-05 | 0.378 | - | - |
| AT1G08610 | 1.63E-05 | 0.379 | - | - |
| AT5G11900 | 1.29E-05 | 0.379 | - | - |
| AT1G18880 | 1.28E-05 | 0.379 | - | - |
| AT4G01010 | 1.13E-05 | 0.379 | - | - |
| AT1G08650 | 1.03E-05 | 0.379 | - | - |
| AT5G50050 | 1.03E-05 | 0.379 | - | - |
| AT2G19740 | 2.11E-05 | 0.38 | - | - |
| AT2G19720 | 1.78E-05 | 0.381 | - | - |
| AT3G26070 | 1.68E-05 | 0.381 | - | - |
| AT1G29900 | 2.36E-05 | 0.382 | - | - |
| AT5G65700 | 1.64E-05 | 0.382 | - | - |
| AT1G18040 | 1.41E-05 | 0.382 | - | - |
| AT5G03415 | 1.38E-05 | 0.382 | - | - |
| AT5G46280 | 3.61E-05 | 0.383 | - | - |
| AT2G41090 | 2.40E-05 | 0.383 | - | - |
| AT4G35350 | 1.56E-05 | 0.383 | - | - |
| AT1G52380 | 1.50E-05 | 0.383 | - | - |
| AT4G02690 | 1.24E-05 | 0.383 | - | - |
| AT3G60500 | 1.22E-05 | 0.383 | - | - |
| AT2G16400 | 1.17E-05 | 0.383 | - | - |
| AT1G44820 | 1.19E-05 | 0.384 | - | - |
| AT5G44320 | 1.10E-05 | 0.384 | - | - |
| AT2G32120 | 1.69E-05 | 0.385 | - | - |
| AT5G64990 | 1.58E-05 | 0.385 | - | - |
| AT1G26820 | 1.44E-05 | 0.385 | - | - |
| AT2G47770 | 1.11E-05 | 0.385 | - | - |
| AT2G36850 | 1.08E-05 | 0.385 | - | - |
| AT1G09500 | 1.08E-05 | 0.385 | - | - |
| AT5G48760 | 2.34E-05 | 0.386 | - | - |
| AT4G18030 | 1.35E-05 | 0.386 | - | - |
| AT4G33250 | 1.21E-05 | 0.386 | - | - |
| AT5G24630 | 1.35E-05 | 0.387 | - | - |
| AT1G60070 | 1.31E-05 | 0.387 | - | - |
| AT5G42760 | 1.11E-05 | 0.387 | - | - |
| AT5G62620 | 1.48E-05 | 0.388 | - | - |
| AT1G19800 | 1.33E-05 | 0.388 | - | - |
| AT4G34460 | 2.68E-05 | 0.389 | - | - |
| AT4G04955 | 2.45E-05 | 0.389 | - | - |
| AT3G01850 | 1.47E-05 | 0.389 | - | - |
| AT5G46900 | 1.01E-05 | 0.389 | - | - |
| AT4G02610 | 1.83E-05 | 0.39 | - | - |
| AT1G59820 | 1.35E-05 | 0.39 | - | - |
| AT1G30600 | 1.03E-05 | 0.39 | - | - |
| AT3G55010 | 2.89E-05 | 0.391 | - | - |
| AT1G09140 | 1.71E-05 | 0.391 | - | - |
| AT1G10500 | 1.53E-05 | 0.391 | - | - |
| AT4G12880 | 1.36E-05 | 0.391 | - | - |
| AT3G15710 | 1.29E-05 | 0.391 | - | - |
| AT5G56180 | 1.28E-05 | 0.391 | - | - |
| AT5G43780 | 1.08E-05 | 0.391 | - | - |
| AT3G48210 | 1.03E-05 | 0.391 | - | - |
| AT4G33200 | 1.01E-05 | 0.391 | - | - |
| AT3G17465 | 2.17E-05 | 0.392 | - | - |
| AT2G30410 | 1.67E-05 | 0.392 | - | - |
| AT2G13810 | 1.42E-05 | 0.392 | - | - |
| AT5G16930 | 1.42E-05 | 0.392 | - | - |
| AT4G34555 | 1.20E-05 | 0.392 | - | - |
| AT5G52020 | 1.15E-05 | 0.392 | - | - |
| AT2G37680 | 1.07E-05 | 0.392 | - | - |
| AT2G37170 | 1.06E-05 | 0.392 | - | - |
| AT1G65480 | 2.04E-05 | 0.393 | - | - |
| AT3G06820 | 1.08E-05 | 0.393 | - | - |
| AT5G01670 | 1.27E-05 | 0.394 | - | - |
| AT4G05070 | 1.26E-05 | 0.394 | - | - |
| AT1G60830 | 1.10E-05 | 0.395 | - | - |
| AT5G38720 | 1.84E-05 | 0.396 | - | - |
| AT3G11940 | 1.78E-05 | 0.396 | - | - |
| AT1G56300 | 1.54E-05 | 0.396 | - | - |
| AT5G17710 | 2.49E-05 | 0.397 | - | - |
| AT3G48090 | 1.56E-05 | 0.397 | - | - |
| AT2G23420 | 1.21E-05 | 0.397 | - | - |
| AT2G37470 | 1.11E-05 | 0.397 | - | - |
| AT5G13680 | 2.52E-05 | 0.398 | - | - |
| AT3G03310 | 1.91E-05 | 0.398 | - | - |
| AT2G04780 | 1.76E-05 | 0.398 | - | - |
| AT4G29570 | 1.13E-05 | 0.398 | - | - |
| AT1G35140 | 1.64E-05 | 0.399 | - | - |
| AT4G39960 | 1.31E-05 | 0.399 | - | - |
| AT5G46160 | 1.30E-05 | 0.399 | - | - |
| AT1G55310 | 1.12E-05 | 0.399 | - | - |
| AT3G46140 | 1.09E-05 | 0.399 | - | - |
| AT4G25740 | 2.41E-05 | 0.4 | - | - |
| AT1G52980 | 1.95E-05 | 0.4 | - | - |
| AT1G64200 | 1.19E-05 | 0.4 | - | - |
| AT3G60830 | 1.09E-05 | 0.4 | - | - |
| AT1G03280 | 1.07E-05 | 0.4 | - | - |
| AT4G04920 | 1.66E-05 | 0.401 | - | - |
| AT2G39400 | 1.46E-05 | 0.401 | - | - |
| AT5G47200 | 1.37E-05 | 0.401 | - | - |
| AT4G14147 | 2.23E-05 | 0.402 | - | - |
| ATCG00740 | 1.76E-05 | 0.402 | - | - |
| AT3G20260 | 1.51E-05 | 0.402 | - | - |
| AT2G29960 | 1.10E-05 | 0.402 | - | - |
| AT2G37678 | 1.06E-05 | 0.402 | - | - |
| AT1G06220 | 3.31E-05 | 0.403 | - | - |
| AT2G35795 | 1.21E-05 | 0.403 | - | - |
| AT3G57270 | 1.02E-05 | 0.403 | - | - |
| AT5G56150 | 2.06E-05 | 0.404 | - | - |
| AT3G49500 | 1.79E-05 | 0.404 | - | - |
| AT1G74950 | 1.33E-05 | 0.404 | - | - |
| AT5G42600 | 1.09E-05 | 0.404 | - | - |
| AT1G17420 | 1.81E-05 | 0.405 | - | - |
| AT5G40040 | 1.34E-05 | 0.405 | - | - |
| AT4G26570 | 1.31E-05 | 0.405 | - | - |
| AT4G04180 | 1.22E-05 | 0.405 | - | - |
| AT3G08550 | 1.47E-05 | 0.406 | - | - |
| AT1G29960 | 1.02E-05 | 0.406 | - | - |
| AT5G64070 | 2.27E-05 | 0.407 | - | - |
| AT4G19110 | 2.00E-05 | 0.407 | - | - |
| AT2G31880 | 1.99E-05 | 0.407 | - | - |
| AT2G40765 | 1.57E-05 | 0.407 | - | - |
| AT3G06440 | 1.46E-05 | 0.407 | - | - |
| AT4G29330 | 1.27E-05 | 0.407 | - | - |
| AT5G65360 | 3.10E-05 | 0.408 | - | - |
| AT1G51710 | 2.36E-05 | 0.408 | - | - |
| AT5G45010 | 1.84E-05 | 0.408 | - | - |
| AT1G64280 | 1.46E-05 | 0.408 | - | - |
| AT3G24495 | 1.78E-05 | 0.409 | - | - |
| AT3G09350 | 1.57E-05 | 0.409 | - | - |
| AT4G31985 | 2.18E-05 | 0.41 | - | - |
| AT3G23250 | 2.03E-05 | 0.41 | - | - |
| AT5G03850 | 1.74E-05 | 0.41 | - | - |
| AT1G11210 | 1.53E-05 | 0.41 | - | - |
| AT2G01470 | 1.30E-05 | 0.41 | - | - |
| AT3G16310 | 1.25E-05 | 0.41 | - | - |
| AT3G23830 | 1.12E-05 | 0.41 | - | - |
| AT3G25780 | 2.10E-05 | 0.411 | - | - |
| AT3G52170 | 1.51E-05 | 0.411 | - | - |
| AT2G17520 | 3.04E-05 | 0.412 | - | - |
| AT5G62300 | 2.37E-05 | 0.412 | - | - |
| AT1G27980 | 1.47E-05 | 0.412 | - | - |
| AT3G44680 | 1.41E-05 | 0.412 | - | - |
| AT1G27380 | 1.03E-05 | 0.412 | - | - |
| AT4G14420 | 1.76E-05 | 0.413 | - | - |
| AT1G64750 | 1.55E-05 | 0.413 | - | - |
| AT1G67980 | 1.41E-05 | 0.413 | - | - |
| AT5G09790 | 1.34E-05 | 0.413 | - | - |
| AT2G47440 | 1.28E-05 | 0.413 | - | - |
| AT1G19890 | 1.27E-05 | 0.413 | - | - |
| AT1G07400 | 1.16E-05 | 0.413 | - | - |
| AT3G48360 | 1.97E-05 | 0.415 | - | - |
| AT3G26618 | 1.55E-05 | 0.415 | - | - |
| AT5G17860 | 1.24E-05 | 0.415 | - | - |
| AT1G75010 | 1.17E-05 | 0.415 | - | - |
| AT4G23820 | 2.39E-05 | 0.416 | - | - |
| AT2G01950 | 1.43E-05 | 0.416 | - | - |
| AT1G72710 | 1.28E-05 | 0.416 | - | - |
| AT1G74460 | 1.22E-05 | 0.416 | - | - |
| AT1G53830 | 1.17E-05 | 0.416 | - | - |
| AT5G12870 | 1.32E-05 | 0.418 | - | - |
| AT1G64500 | 1.19E-05 | 0.418 | - | - |
| AT5G06500 | 1.08E-05 | 0.418 | - | - |
| AT4G38600 | 3.18E-05 | 0.419 | - | - |
| AT3G12050 | 1.14E-05 | 0.419 | - | - |
| AT1G34220 | 1.08E-05 | 0.419 | - | - |
| AT4G18593 | 1.35E-05 | 0.42 | - | - |
| AT3G57510 | 1.21E-05 | 0.42 | - | - |
| AT2G25970 | 1.12E-05 | 0.42 | - | - |
| AT3G66656 | 1.05E-05 | 0.42 | - | - |
| AT1G51060 | 1.69E-05 | 0.421 | - | - |
| AT1G60230 | 1.12E-05 | 0.421 | - | - |
| AT1G07210 | 1.07E-05 | 0.421 | - | - |
| AT1G74050 | 2.12E-05 | 0.422 | - | - |
| AT4G28740 | 1.68E-05 | 0.422 | - | - |
| AT1G18570 | 1.61E-05 | 0.422 | - | - |
| AT5G02230 | 1.08E-05 | 0.422 | - | - |
| AT2G23800 | 1.04E-05 | 0.422 | - | - |
| AT5G65660 | 1.03E-05 | 0.422 | - | - |
| AT3G58550 | 1.02E-05 | 0.422 | - | - |
| AT2G19070 | 1.02E-05 | 0.422 | - | - |
| AT3G52630 | 1.01E-05 | 0.422 | - | - |
| AT3G55270 | 2.11E-05 | 0.423 | - | - |
| AT5G10360 | 2.16E-05 | 0.425 | - | - |
| AT1G75780 | 1.64E-05 | 0.425 | - | - |
| AT3G22380 | 1.02E-05 | 0.425 | - | - |
| AT4G21710 | 3.18E-05 | 0.426 | - | - |
| AT5G10980 | 2.44E-05 | 0.426 | - | - |
| AT1G26810 | 1.89E-05 | 0.427 | - | - |
| AT3G06950 | 1.16E-05 | 0.427 | - | - |
| AT3G44600 | 1.14E-05 | 0.427 | - | - |
| AT5G01380 | 1.12E-05 | 0.427 | - | - |
| AT3G54560 | 3.14E-05 | 0.428 | - | - |
| AT1G02140 | 1.77E-05 | 0.428 | - | - |
| AT2G05520 | 1.43E-05 | 0.428 | - | - |
| AT2G30000 | 1.24E-05 | 0.428 | - | - |
| AT2G24240 | 1.05E-05 | 0.428 | - | - |
| AT1G72550 | 1.80E-05 | 0.429 | - | - |
| AT5G65330 | 1.29E-05 | 0.429 | - | - |
| AT5G62000 | 1.26E-05 | 0.429 | - | - |
| AT3G14067 | 1.94E-05 | 0.43 | - | - |
| AT5G10160 | 1.11E-05 | 0.43 | - | - |
| AT2G30280 | 1.63E-05 | 0.431 | - | - |
| AT3G03070 | 1.37E-05 | 0.431 | - | - |
| AT4G36540 | 2.43E-05 | 0.432 | - | - |
| AT4G30930 | 1.70E-05 | 0.432 | - | - |
| AT1G17680 | 1.29E-05 | 0.432 | - | - |
| AT3G56290 | 1.10E-05 | 0.432 | - | - |
| AT1G33060 | 1.45E-05 | 0.433 | - | - |
| AT1G55570 | 1.16E-05 | 0.433 | - | - |
| AT2G46040 | 1.73E-05 | 0.434 | - | - |
| AT4G37000 | 1.69E-05 | 0.435 | - | - |
| AT2G18770 | 1.26E-05 | 0.435 | - | - |
| AT5G16050 | 1.08E-05 | 0.435 | - | - |
| AT3G49790 | 1.60E-05 | 0.436 | - | - |
| AT4G24700 | 1.34E-05 | 0.436 | - | - |
| AT5G51190 | 1.07E-05 | 0.436 | - | - |
| AT4G30530 | 1.04E-05 | 0.436 | - | - |
| AT1G07940 | 1.71E-05 | 0.437 | - | - |
| AT4G27330 | 1.68E-05 | 0.437 | - | - |
| AT1G18300 | 1.46E-05 | 0.437 | - | - |
| AT3G08730 | 1.33E-05 | 0.437 | - | - |
| AT3G54620 | 2.77E-05 | 0.438 | - | - |
| AT2G19520 | 2.02E-05 | 0.438 | - | - |
| AT4G12780 | 1.77E-05 | 0.439 | - | - |
| AT3G52850 | 1.39E-05 | 0.439 | - | - |
| AT1G16070 | 1.09E-05 | 0.439 | - | - |
| AT1G29270 | 1.04E-05 | 0.439 | - | - |
| AT3G15460 | 2.59E-05 | 0.44 | - | - |
| ATCG01230 | 1.11E-05 | 0.44 | - | - |
| AT5G37670 | 1.04E-05 | 0.44 | - | - |
| AT3G10160 | 1.29E-05 | 0.441 | - | - |
| AT3G46010 | 1.16E-05 | 0.441 | - | - |
| AT4G31810 | 1.51E-05 | 0.442 | - | - |
| AT3G58560 | 1.17E-05 | 0.442 | - | - |
| AT1G20200 | 2.88E-05 | 0.443 | - | - |
| AT1G04820 | 1.49E-05 | 0.443 | - | - |
| AT1G02870 | 1.34E-05 | 0.443 | - | - |
| AT4G14145 | 1.90E-05 | 0.444 | - | - |
| AT4G14550 | 1.48E-05 | 0.444 | - | - |
| AT1G30400 | 1.06E-05 | 0.444 | - | - |
| AT5G58700 | 1.02E-05 | 0.444 | - | - |
| AT1G13710 | 1.57E-05 | 0.445 | - | - |
| AT1G64600 | 1.48E-05 | 0.445 | - | - |
| AT3G49940 | 1.10E-05 | 0.445 | - | - |
| AT4G37770 | 1.04E-05 | 0.445 | - | - |
| AT5G23070 | 1.02E-05 | 0.445 | - | - |
| AT3G54970 | 1.01E-05 | 0.445 | - | - |
| AT1G66520 | 1.60E-05 | 0.446 | - | - |
| AT3G48780 | 1.25E-05 | 0.446 | - | - |
| AT2G38090 | 1.23E-05 | 0.446 | - | - |
| AT1G59406 | 1.04E-05 | 0.446 | - | - |
| AT3G56070 | 2.29E-05 | 0.447 | - | - |
| AT5G02440 | 1.79E-05 | 0.447 | - | - |
| AT3G56400 | 1.28E-05 | 0.447 | - | - |
| AT5G22880 | 1.24E-05 | 0.447 | - | - |
| AT1G31170 | 1.04E-05 | 0.447 | - | - |
| AT2G19770 | 1.73E-05 | 0.448 | - | - |
| AT4G37640 | 1.54E-05 | 0.448 | - | - |
| AT1G03590 | 1.35E-05 | 0.448 | - | - |
| AT1G71697 | 1.16E-05 | 0.448 | - | - |
| AT4G34840 | 1.41E-05 | 0.449 | - | - |
| AT5G39520 | 1.39E-05 | 0.449 | - | - |
| AT5G40870 | 1.37E-05 | 0.449 | - | - |
| AT1G69460 | 1.06E-05 | 0.449 | - | - |
| AT2G45810 | 1.59E-05 | 0.45 | - | - |
| AT2G25620 | 1.55E-05 | 0.45 | - | - |
| AT5G63080 | 1.43E-05 | 0.45 | - | - |
| AT5G41700 | 1.31E-05 | 0.45 | - | - |
| AT2G31270 | 1.23E-05 | 0.45 | - | - |
| AT5G07630 | 1.56E-05 | 0.451 | - | - |
| AT4G17490 | 1.16E-05 | 0.453 | - | - |
| AT2G20140 | 2.61E-05 | 0.454 | - | - |
| AT4G31580 | 1.32E-05 | 0.454 | - | - |
| AT3G08680 | 1.20E-05 | 0.454 | - | - |
| AT3G43270 | 1.12E-05 | 0.454 | - | - |
| AT5G47630 | 2.68E-05 | 0.455 | - | - |
| AT1G03150 | 1.56E-05 | 0.455 | - | - |
| AT2G27500 | 1.56E-05 | 0.455 | - | - |
| AT1G11660 | 1.41E-05 | 0.455 | - | - |
| AT4G21530 | 1.31E-05 | 0.455 | - | - |
| AT1G18640 | 1.41E-05 | 0.456 | - | - |
| AT1G12500 | 1.87E-05 | 0.457 | - | - |
| AT1G55610 | 1.49E-05 | 0.457 | - | - |
| AT5G35460 | 2.31E-05 | 0.458 | - | - |
| AT4G10320 | 1.96E-05 | 0.458 | - | - |
| AT3G48100 | 1.26E-05 | 0.458 | - | - |
| AT3G27440 | 1.23E-05 | 0.458 | - | - |
| AT5G04340 | 2.32E-05 | 0.459 | - | - |
| AT5G56860 | 1.09E-05 | 0.459 | - | - |
| AT5G66230 | 1.08E-05 | 0.459 | - | - |
| AT4G25630 | 3.89E-05 | 0.46 | - | - |
| AT4G35910 | 1.49E-05 | 0.46 | - | - |
| AT2G24500 | 1.33E-05 | 0.46 | - | - |
| AT1G51660 | 1.26E-05 | 0.46 | - | - |
| AT2G01490 | 1.07E-05 | 0.461 | - | - |
| AT2G17560 | 1.02E-05 | 0.461 | - | - |
| AT5G41200 | 1.49E-05 | 0.462 | - | - |
| AT2G39570 | 1.34E-05 | 0.462 | - | - |
| AT5G57160 | 3.81E-05 | 0.463 | - | - |
| AT4G21270 | 1.62E-05 | 0.463 | - | - |
| AT5G44110 | 1.18E-05 | 0.463 | - | - |
| AT3G45040 | 1.72E-05 | 0.464 | - | - |
| AT2G01720 | 1.64E-05 | 0.464 | - | - |
| AT3G10670 | 1.77E-05 | 0.465 | - | - |
| AT1G80370 | 1.21E-05 | 0.465 | - | - |
| AT2G43460 | 1.21E-05 | 0.465 | - | - |
| AT1G54370 | 1.16E-05 | 0.465 | - | - |
| AT2G26140 | 1.15E-05 | 0.465 | - | - |
| AT4G38880 | 1.13E-05 | 0.465 | - | - |
| AT1G64550 | 1.98E-05 | 0.466 | - | - |
| AT5G22260 | 1.96E-05 | 0.466 | - | - |
| AT5G05560 | 1.90E-05 | 0.466 | - | - |
| AT1G27240 | 1.21E-05 | 0.466 | - | - |
| AT4G22010 | 2.23E-05 | 0.467 | - | - |
| AT2G37760 | 1.43E-05 | 0.467 | - | - |
| AT1G60420 | 1.28E-05 | 0.467 | - | - |
| AT5G04170 | 1.13E-05 | 0.467 | - | - |
| AT1G18890 | 2.04E-05 | 0.468 | - | - |
| AT2G34640 | 1.72E-05 | 0.469 | - | - |
| AT2G46230 | 1.39E-05 | 0.469 | - | - |
| AT4G17840 | 1.10E-05 | 0.469 | - | - |
| AT5G55190 | 1.43E-05 | 0.47 | - | - |
| AT2G23080 | 1.15E-05 | 0.47 | - | - |
| AT1G16920 | 1.14E-05 | 0.47 | - | - |
| AT1G12920 | 2.21E-05 | 0.471 | - | - |
| AT2G45070 | 1.31E-05 | 0.471 | - | - |
| AT1G07790 | 1.15E-05 | 0.471 | - | - |
| AT1G76150 | 1.95E-05 | 0.472 | - | - |
| AT4G05260 | 1.53E-05 | 0.472 | - | - |
| AT4G11110 | 1.47E-05 | 0.472 | - | - |
| AT3G15540 | 1.10E-05 | 0.472 | - | - |
| AT5G45230 | 1.61E-05 | 0.473 | - | - |
| AT5G08290 | 1.44E-05 | 0.473 | - | - |
| AT1G07810 | 1.90E-05 | 0.474 | - | - |
| AT4G37650 | 1.88E-05 | 0.474 | - | - |
| AT3G27190 | 1.24E-05 | 0.474 | - | - |
| AT4G05250 | 1.07E-05 | 0.474 | - | - |
| AT1G63280 | 1.85E-05 | 0.475 | - | - |
| AT5G23240 | 1.82E-05 | 0.475 | - | - |
| AT1G22310 | 1.79E-05 | 0.476 | - | - |
| AT5G61880 | 1.32E-05 | 0.476 | - | - |
| AT4G30520 | 1.13E-05 | 0.476 | - | - |
| AT1G19530 | 1.15E-05 | 0.477 | - | - |
| AT4G32280 | 1.11E-05 | 0.477 | - | - |
| AT4G27130 | 1.05E-05 | 0.477 | - | - |
| AT5G62490 | 1.48E-05 | 0.478 | - | - |
| AT1G34200 | 1.26E-05 | 0.478 | - | - |
| AT2G20060 | 2.71E-05 | 0.479 | - | - |
| AT1G72770 | 2.68E-05 | 0.479 | - | - |
| AT4G26110 | 1.65E-05 | 0.479 | - | - |
| AT4G23700 | 1.33E-05 | 0.479 | - | - |
| AT2G40010 | 2.35E-05 | 0.48 | - | - |
| AT3G03710 | 2.19E-05 | 0.48 | - | - |
| AT2G03110 | 1.40E-05 | 0.48 | - | - |
| AT5G20150 | 1.09E-05 | 0.48 | - | - |
| AT3G48960 | 1.48E-05 | 0.481 | - | - |
| AT3G13640 | 1.46E-05 | 0.481 | - | - |
| AT3G18730 | 1.39E-05 | 0.481 | - | - |
| AT1G47230 | 1.21E-05 | 0.481 | - | - |
| AT1G11680 | 2.16E-05 | 0.482 | - | - |
| AT5G48180 | 1.19E-05 | 0.482 | - | - |
| AT3G62040 | 1.01E-05 | 0.482 | - | - |
| AT4G00100 | 3.02E-05 | 0.483 | - | - |
| AT5G17990 | 2.00E-05 | 0.483 | - | - |
| AT5G11920 | 1.68E-05 | 0.483 | - | - |
| AT3G06710 | 1.11E-05 | 0.483 | - | - |
| AT5G28770 | 1.51E-05 | 0.484 | - | - |
| AT3G26290 | 1.16E-05 | 0.484 | - | - |
| AT4G18630 | 1.05E-05 | 0.484 | - | - |
| AT1G55520 | 2.65E-05 | 0.485 | - | - |
| AT1G55860 | 1.23E-05 | 0.485 | - | - |
| AT3G19720 | 1.19E-05 | 0.485 | - | - |
| AT1G60900 | 1.16E-05 | 0.485 | - | - |
| AT2G15890 | 1.96E-05 | 0.486 | - | - |
| AT2G02390 | 1.56E-05 | 0.486 | - | - |
| AT2G15570 | 1.35E-05 | 0.486 | - | - |
| AT5G35700 | 1.32E-05 | 0.486 | - | - |
| AT1G53300 | 1.20E-05 | 0.486 | - | - |
| AT4G25340 | 1.37E-05 | 0.487 | - | - |
| AT3G03060 | 1.33E-05 | 0.488 | - | - |
| AT3G14950 | 1.11E-05 | 0.488 | - | - |
| AT1G33430 | 1.03E-05 | 0.488 | - | - |
| AT3G02020 | 1.03E-05 | 0.488 | - | - |
| AT1G17380 | 2.13E-05 | 0.489 | - | - |
| AT2G18690 | 1.88E-05 | 0.489 | - | - |
| AT1G49530 | 1.83E-05 | 0.489 | - | - |
| AT4G28250 | 1.10E-05 | 0.49 | - | - |
| AT4G16845 | 1.02E-05 | 0.49 | - | - |
| AT1G68890 | 2.69E-05 | 0.491 | - | - |
| AT1G48410 | 1.55E-05 | 0.491 | - | - |
| AT5G03430 | 1.26E-05 | 0.491 | - | - |
| AT1G55810 | 1.70E-05 | 0.492 | - | - |
| AT3G12250 | 1.28E-05 | 0.492 | - | - |
| AT2G36010 | 1.21E-05 | 0.492 | - | - |
| AT5G66390 | 1.08E-05 | 0.492 | - | - |
| AT2G25060 | 1.76E-05 | 0.493 | - | - |
| AT5G53000 | 1.62E-05 | 0.493 | - | - |
| AT1G10630 | 1.65E-05 | 0.494 | - | - |
| AT2G38290 | 1.27E-05 | 0.494 | - | - |
| AT1G59850 | 1.03E-05 | 0.494 | - | - |
| AT5G17660 | 1.52E-05 | 0.495 | - | - |
| AT2G28520 | 1.22E-05 | 0.495 | - | - |
| AT4G14570 | 1.18E-05 | 0.495 | - | - |
| AT2G01980 | 1.12E-05 | 0.495 | - | - |
| AT3G17590 | 3.49E-05 | 0.496 | - | - |
| AT3G46060 | 2.54E-05 | 0.496 | - | - |
| AT4G16340 | 1.26E-05 | 0.496 | - | - |
| AT4G02120 | 1.30E-05 | 0.497 | - | - |
| AT5G46300 | 1.17E-05 | 0.497 | - | - |
| AT1G80670 | 3.76E-05 | 0.499 | - | - |
| AT2G43210 | 2.06E-05 | 0.499 | - | - |
| AT2G30020 | 1.65E-05 | 0.499 | - | - |
| AT2G22400 | 1.37E-05 | 0.499 | - | - |
| AT4G34410 | 1.37E-05 | 0.499 | - | - |
| AT5G13310 | 1.23E-05 | 0.499 | - | - |
| AT1G65470 | 1.14E-05 | 0.499 | - | - |
| AT5G20900 | 1.07E-05 | 0.499 | - | - |
| AT4G39200 | 1.05E-05 | 0.499 | - | - |
| AT1G74670 | 1.27E-05 | 0.5 | - | - |
| AT5G47080 | 1.64E-05 | 0.501 | - | - |
| AT5G06140 | 1.58E-05 | 0.501 | - | - |
| AT3G59540 | 2.27E-05 | 0.502 | - | - |
| AT1G05260 | 1.66E-05 | 0.502 | - | - |
| AT3G02540 | 1.62E-05 | 0.502 | - | - |
| AT1G43800 | 1.37E-05 | 0.502 | - | - |
| AT5G27220 | 1.33E-05 | 0.502 | - | - |
| AT3G45970 | 1.30E-05 | 0.502 | - | - |
| AT2G18510 | 1.29E-05 | 0.502 | - | - |
| AT1G63970 | 2.90E-05 | 0.503 | - | - |
| AT3G45770 | 2.02E-05 | 0.503 | - | - |
| AT3G05540 | 1.09E-05 | 0.503 | - | - |
| AT3G50750 | 1.07E-05 | 0.503 | - | - |
| AT4G17510 | 1.01E-05 | 0.503 | - | - |
| AT1G75990 | 2.65E-05 | 0.504 | - | - |
| AT3G01270 | 2.57E-05 | 0.504 | - | - |
| AT5G26751 | 2.03E-05 | 0.504 | - | - |
| AT5G56940 | 1.46E-05 | 0.504 | - | - |
| AT1G69850 | 1.22E-05 | 0.504 | - | - |
| AT3G13530 | 1.22E-05 | 0.504 | - | - |
| AT1G52750 | 1.09E-05 | 0.504 | - | - |
| AT1G11600 | 1.27E-05 | 0.505 | - | - |
| AT5G40480 | 1.05E-05 | 0.505 | - | - |
| AT3G25980 | 4.24E-05 | 0.506 | - | - |
| AT5G23630 | 2.61E-05 | 0.506 | - | - |
| AT5G45140 | 2.45E-05 | 0.506 | - | - |
| AT1G29280 | 1.13E-05 | 0.506 | - | - |
| AT1G61700 | 1.71E-05 | 0.508 | - | - |
| AT5G39610 | 1.35E-05 | 0.508 | - | - |
| AT1G19770 | 1.44E-05 | 0.509 | - | - |
| ATCG00160 | 1.25E-05 | 0.509 | - | - |
| AT1G74270 | 1.76E-05 | 0.51 | - | - |
| AT3G13400 | 1.01E-05 | 0.51 | - | - |
| AT4G21670 | 1.74E-05 | 0.511 | - | - |
| AT1G53710 | 1.00E-05 | 0.511 | - | - |
| AT1G78510 | 1.92E-05 | 0.512 | - | - |
| AT5G61070 | 1.42E-05 | 0.512 | - | - |
| AT1G74930 | 1.27E-05 | 0.512 | - | - |
| AT3G56370 | 2.26E-05 | 0.513 | - | - |
| AT5G67510 | 1.89E-05 | 0.513 | - | - |
| AT5G54810 | 1.54E-05 | 0.513 | - | - |
| AT2G16960 | 2.80E-05 | 0.514 | - | - |
| AT4G23800 | 1.71E-05 | 0.514 | - | - |
| AT5G16120 | 1.59E-05 | 0.515 | - | - |
| ATMG00410 | 1.44E-05 | 0.515 | - | - |
| AT5G27520 | 1.36E-05 | 0.515 | - | - |
| AT1G77540 | 1.36E-05 | 0.515 | - | - |
| AT5G57110 | 1.14E-05 | 0.515 | - | - |
| AT1G54390 | 1.35E-05 | 0.516 | - | - |
| AT4G13510 | 1.19E-05 | 0.516 | - | - |
| AT2G16740 | 1.00E-05 | 0.516 | - | - |
| AT1G75820 | 1.43E-05 | 0.517 | - | - |
| AT1G68850 | 1.24E-05 | 0.517 | - | - |
| AT5G57870 | 1.68E-05 | 0.518 | - | - |
| AT5G56620 | 1.23E-05 | 0.518 | - | - |
| AT2G25560 | 1.03E-05 | 0.518 | - | - |
| AT3G02380 | 1.66E-05 | 0.519 | - | - |
| AT5G60490 | 1.59E-05 | 0.519 | - | - |
| AT4G12720 | 2.07E-05 | 0.52 | - | - |
| AT1G09070 | 1.87E-05 | 0.52 | - | - |
| AT1G07660 | 1.25E-05 | 0.52 | - | - |
| AT4G33350 | 1.17E-05 | 0.52 | - | - |
| AT1G22280 | 1.53E-05 | 0.521 | - | - |
| AT4G38660 | 1.46E-05 | 0.521 | - | - |
| AT3G19040 | 1.27E-05 | 0.521 | - | - |
| AT4G23180 | 1.24E-05 | 0.521 | - | - |
| AT3G18000 | 1.23E-05 | 0.521 | - | - |
| AT5G61810 | 1.13E-05 | 0.521 | - | - |
| AT1G70290 | 2.19E-05 | 0.522 | - | - |
| AT1G77720 | 1.22E-05 | 0.522 | - | - |
| AT5G01820 | 1.19E-05 | 0.522 | - | - |
| AT3G58120 | 2.01E-05 | 0.523 | - | - |
| AT2G27720 | 1.90E-05 | 0.523 | - | - |
| AT1G49410 | 1.67E-05 | 0.523 | - | - |
| AT4G38080 | 1.22E-05 | 0.523 | - | - |
| AT2G34710 | 1.93E-05 | 0.524 | - | - |
| AT5G16560 | 1.74E-05 | 0.524 | - | - |
| AT4G24270 | 1.43E-05 | 0.524 | - | - |
| AT4G40030 | 2.27E-05 | 0.525 | - | - |
| AT3G57180 | 1.56E-05 | 0.525 | - | - |
| ATMG01275 | 1.06E-05 | 0.525 | - | - |
| AT1G21060 | 1.04E-05 | 0.525 | - | - |
| AT4G16660 | 3.46E-05 | 0.526 | - | - |
| AT4G18510 | 1.25E-05 | 0.526 | - | - |
| AT4G02390 | 1.00E-05 | 0.526 | - | - |
| AT2G45710 | 1.82E-05 | 0.527 | - | - |
| AT5G45970 | 1.36E-05 | 0.527 | - | - |
| ATMG00090 | 1.31E-05 | 0.527 | - | - |
| AT4G34680 | 1.28E-05 | 0.528 | - | - |
| AT2G36910 | 1.07E-05 | 0.528 | - | - |
| AT3G42790 | 1.01E-05 | 0.528 | - | - |
| AT1G07180 | 1.26E-05 | 0.529 | - | - |
| AT1G29320 | 1.09E-05 | 0.529 | - | - |
| AT5G08660 | 1.02E-05 | 0.529 | - | - |
| AT5G49160 | 2.23E-05 | 0.53 | - | - |
| AT5G01230 | 1.28E-05 | 0.53 | - | - |
| AT5G62310 | 1.20E-05 | 0.531 | - | - |
| AT3G02600 | 1.66E-05 | 0.532 | - | - |
| AT5G53350 | 1.15E-05 | 0.532 | - | - |
| AT5G64390 | 1.00E-05 | 0.532 | - | - |
| AT1G74590 | 2.00E-05 | 0.533 | - | - |
| AT1G16700 | 1.47E-05 | 0.533 | - | - |
| AT3G18690 | 1.17E-05 | 0.533 | - | - |
| AT3G57450 | 1.84E-05 | 0.534 | - | - |
| AT2G40000 | 2.29E-05 | 0.537 | - | - |
| ATCG01310 | 1.26E-05 | 0.537 | - | - |
| AT5G16210 | 1.22E-05 | 0.537 | - | - |
| AT5G42570 | 1.07E-05 | 0.537 | - | - |
| AT1G32210 | 1.02E-05 | 0.538 | - | - |
| AT2G31725 | 1.05E-05 | 0.539 | - | - |
| AT3G61111 | 1.01E-05 | 0.539 | - | - |
| AT4G39030 | 1.38E-05 | 0.54 | - | - |
| AT5G14550 | 1.19E-05 | 0.54 | - | - |
| AT1G80440 | 1.78E-05 | 0.541 | - | - |
| AT4G15880 | 1.35E-05 | 0.541 | - | - |
| AT2G15790 | 1.87E-05 | 0.542 | - | - |
| AT4G11380 | 1.80E-05 | 0.542 | - | - |
| AT1G74430 | 1.15E-05 | 0.542 | - | - |
| AT4G29900 | 1.04E-05 | 0.542 | - | - |
| AT2G21040 | 1.04E-05 | 0.543 | - | - |
| AT3G09010 | 1.07E-05 | 0.544 | - | - |
| AT5G59220 | 2.13E-05 | 0.545 | - | - |
| AT1G32750 | 1.97E-05 | 0.545 | - | - |
| AT5G22920 | 1.77E-05 | 0.545 | - | - |
| AT5G05010 | 1.66E-05 | 0.545 | - | - |
| AT3G27530 | 1.23E-05 | 0.545 | - | - |
| AT3G16890 | 1.19E-05 | 0.545 | - | - |
| AT1G01300 | 1.08E-05 | 0.546 | - | - |
| AT2G40340 | 1.83E-05 | 0.547 | - | - |
| AT4G19680 | 1.16E-05 | 0.547 | - | - |
| AT5G11340 | 1.10E-05 | 0.548 | - | - |
| AT3G46560 | 1.66E-05 | 0.549 | - | - |
| AT4G32720 | 1.48E-05 | 0.549 | - | - |
| AT5G44780 | 1.39E-05 | 0.549 | - | - |
| AT5G18500 | 1.07E-05 | 0.549 | - | - |
| AT3G10690 | 2.24E-05 | 0.55 | - | - |
| AT2G41190 | 1.62E-05 | 0.55 | - | - |
| AT4G14160 | 1.45E-05 | 0.55 | - | - |
| AT1G17500 | 1.31E-05 | 0.55 | - | - |
| AT5G59370 | 1.27E-05 | 0.55 | - | - |
| AT5G22800 | 1.61E-05 | 0.552 | - | - |
| AT4G26510 | 1.37E-05 | 0.552 | - | - |
| AT4G17190 | 1.12E-05 | 0.552 | - | - |
| AT3G06010 | 1.40E-05 | 0.553 | - | - |
| AT5G27150 | 1.61E-05 | 0.554 | - | - |
| AT3G16100 | 1.48E-05 | 0.554 | - | - |
| AT1G26380 | 1.41E-05 | 0.554 | - | - |
| AT3G04830 | 1.28E-05 | 0.554 | - | - |
| AT3G10650 | 1.22E-05 | 0.554 | - | - |
| AT3G51300 | 1.06E-05 | 0.554 | - | - |
| AT2G26830 | 1.05E-05 | 0.554 | - | - |
| AT1G02730 | 2.14E-05 | 0.555 | - | - |
| AT5G59690 | 1.64E-05 | 0.555 | - | - |
| AT5G47560 | 1.60E-05 | 0.555 | - | - |
| AT2G16570 | 1.36E-05 | 0.555 | - | - |
| AT5G56950 | 1.14E-05 | 0.555 | - | - |
| AT2G24170 | 1.12E-05 | 0.555 | - | - |
| AT3G45640 | 2.29E-05 | 0.556 | - | - |
| AT1G06040 | 1.31E-05 | 0.556 | - | - |
| AT1G18650 | 1.75E-05 | 0.557 | - | - |
| AT4G26410 | 1.29E-05 | 0.557 | - | - |
| AT1G50010 | 1.23E-05 | 0.557 | - | - |
| AT1G63680 | 2.07E-05 | 0.558 | - | - |
| AT3G47460 | 1.79E-05 | 0.558 | - | - |
| AT4G05020 | 1.28E-05 | 0.558 | - | - |
| AT5G19310 | 1.28E-05 | 0.558 | - | - |
| AT5G56580 | 1.12E-05 | 0.558 | - | - |
| AT1G13370 | 2.13E-05 | 0.559 | - | - |
| AT1G11475 | 1.65E-05 | 0.559 | - | - |
| AT1G68710 | 1.18E-05 | 0.559 | - | - |
| AT1G80500 | 1.15E-05 | 0.559 | - | - |
| AT4G27720 | 1.02E-05 | 0.559 | - | - |
| AT4G21580 | 1.62E-05 | 0.56 | - | - |
| AT3G07820 | 2.27E-05 | 0.561 | - | - |
| AT3G60240 | 1.62E-05 | 0.561 | - | - |
| AT4G04350 | 2.92E-05 | 0.562 | - | - |
| AT4G29510 | 1.64E-05 | 0.562 | - | - |
| AT5G10860 | 1.15E-05 | 0.562 | - | - |
| AT4G39050 | 1.11E-05 | 0.562 | - | - |
| AT3G13490 | 1.09E-05 | 0.562 | - | - |
| AT2G25580 | 1.31E-05 | 0.563 | - | - |
| AT2G32765 | 2.58E-05 | 0.564 | - | - |
| AT3G06400 | 2.05E-05 | 0.564 | - | - |
| AT5G28060 | 1.96E-05 | 0.564 | - | - |
| AT4G01000 | 1.44E-05 | 0.564 | - | - |
| AT5G47770 | 1.18E-05 | 0.564 | - | - |
| AT5G55170 | 2.77E-05 | 0.566 | - | - |
| AT4G30170 | 1.62E-05 | 0.566 | - | - |
| AT4G25240 | 1.01E-05 | 0.567 | - | - |
| AT4G33950 | 1.50E-05 | 0.568 | - | - |
| AT4G17500 | 1.41E-05 | 0.568 | - | - |
| AT3G27800 | 1.50E-05 | 0.569 | - | - |
| AT5G38650 | 1.46E-05 | 0.569 | - | - |
| AT2G25900 | 1.37E-05 | 0.569 | - | - |
| AT3G05690 | 1.44E-05 | 0.57 | - | - |
| AT1G36240 | 1.34E-05 | 0.57 | - | - |
| AT3G25560 | 1.01E-05 | 0.57 | - | - |
| AT5G42080 | 1.57E-05 | 0.571 | - | - |
| AT5G37770 | 1.25E-05 | 0.571 | - | - |
| AT3G62530 | 1.09E-05 | 0.571 | - | - |
| AT5G19485 | 1.02E-05 | 0.571 | - | - |
| AT5G61060 | 1.41E-05 | 0.572 | - | - |
| AT4G20320 | 1.15E-05 | 0.572 | - | - |
| AT2G32000 | 1.13E-05 | 0.572 | - | - |
| AT1G67350 | 1.02E-05 | 0.572 | - | - |
| AT2G20450 | 3.33E-05 | 0.573 | - | - |
| AT5G19820 | 2.34E-05 | 0.573 | - | - |
| AT2G06530 | 1.10E-05 | 0.573 | - | - |
| AT1G64880 | 2.24E-05 | 0.574 | - | - |
| AT3G04240 | 2.18E-05 | 0.574 | - | - |
| AT1G65790 | 1.40E-05 | 0.574 | - | - |
| AT1G13090 | 1.10E-05 | 0.574 | - | - |
| AT3G48150 | 2.11E-05 | 0.575 | - | - |
| AT5G04720 | 1.74E-05 | 0.575 | - | - |
| AT1G79830 | 1.60E-05 | 0.575 | - | - |
| AT5G47390 | 1.01E-05 | 0.575 | - | - |
| AT1G67100 | 1.00E-05 | 0.575 | - | - |
| AT3G51190 | 1.46E-05 | 0.577 | - | - |
| AT5G49180 | 1.29E-05 | 0.577 | - | - |
| AT2G24540 | 1.17E-05 | 0.577 | - | - |
| AT1G22190 | 1.06E-05 | 0.577 | - | - |
| AT2G33840 | 1.58E-05 | 0.578 | - | - |
| AT5G64370 | 1.38E-05 | 0.578 | - | - |
| AT1G23100 | 1.07E-05 | 0.578 | - | - |
| AT3G10090 | 1.37E-05 | 0.58 | - | - |
| AT2G32950 | 3.69E-05 | 0.581 | - | - |
| AT5G10390 | 2.49E-05 | 0.581 | - | - |
| AT3G06720 | 1.28E-05 | 0.581 | - | - |
| AT5G39890 | 1.18E-05 | 0.581 | - | - |
| AT5G10790 | 1.26E-05 | 0.582 | - | - |
| AT4G37110 | 1.32E-05 | 0.583 | - | - |
| AT3G13300 | 1.10E-05 | 0.583 | - | - |
| AT1G80210 | 1.08E-05 | 0.583 | - | - |
| AT1G27120 | 2.11E-05 | 0.584 | - | - |
| AT5G63620 | 1.79E-05 | 0.584 | - | - |
| AT4G30800 | 2.76E-05 | 0.585 | - | - |
| AT1G80270 | 2.01E-05 | 0.585 | - | - |
| AT1G69930 | 1.53E-05 | 0.585 | - | - |
| AT1G69400 | 1.44E-05 | 0.585 | - | - |
| AT5G67280 | 2.00E-05 | 0.586 | - | - |
| AT4G35750 | 1.88E-05 | 0.586 | - | - |
| AT1G80740 | 1.14E-05 | 0.586 | - | - |
| AT4G27280 | 1.86E-05 | 0.587 | - | - |
| AT1G32330 | 1.29E-05 | 0.587 | - | - |
| AT1G20370 | 1.20E-05 | 0.587 | - | - |
| AT3G54380 | 1.03E-05 | 0.587 | - | - |
| AT2G21550 | 3.67E-05 | 0.588 | - | - |
| AT3G20330 | 2.77E-05 | 0.588 | - | - |
| AT1G12610 | 1.59E-05 | 0.588 | - | - |
| AT4G33270 | 1.35E-05 | 0.588 | - | - |
| AT5G54940 | 1.33E-05 | 0.588 | - | - |
| AT1G54160 | 1.28E-05 | 0.588 | - | - |
| AT4G35550 | 2.57E-05 | 0.589 | - | - |
| AT3G12670 | 1.52E-05 | 0.589 | - | - |
| AT5G05730 | 1.27E-05 | 0.59 | - | - |
| AT3G51590 | 1.04E-05 | 0.59 | - | - |
| AT3G17840 | 1.02E-05 | 0.59 | - | - |
| AT3G12270 | 1.89E-05 | 0.591 | - | - |
| AT3G50500 | 1.79E-05 | 0.591 | - | - |
| AT2G44810 | 1.13E-05 | 0.591 | - | - |
| AT3G60540 | 1.10E-05 | 0.591 | - | - |
| AT1G04210 | 3.36E-05 | 0.592 | - | - |
| AT4G26780 | 1.60E-05 | 0.592 | - | - |
| AT1G17050 | 1.49E-05 | 0.592 | - | - |
| AT2G46290 | 1.07E-05 | 0.592 | - | - |
| AT1G52890 | 1.56E-05 | 0.593 | - | - |
| AT2G37550 | 1.61E-05 | 0.594 | - | - |
| AT2G19790 | 1.42E-05 | 0.594 | - | - |
| AT4G25450 | 1.31E-05 | 0.594 | - | - |
| AT1G24250 | 1.31E-05 | 0.594 | - | - |
| AT3G53190 | 2.35E-05 | 0.595 | - | - |
| AT1G01370 | 1.65E-05 | 0.595 | - | - |
| AT2G41720 | 1.19E-05 | 0.595 | - | - |
| AT5G54950 | 1.10E-05 | 0.595 | - | - |
| AT3G08850 | 2.15E-05 | 0.596 | - | - |
| AT2G27550 | 1.50E-05 | 0.596 | - | - |
| AT4G01330 | 1.09E-05 | 0.596 | - | - |
| AT2G28900 | 2.66E-05 | 0.597 | - | - |
| AT3G02780 | 1.68E-05 | 0.598 | - | - |
| AT4G19210 | 1.57E-05 | 0.599 | - | - |
| AT5G27820 | 1.57E-05 | 0.599 | - | - |
| AT1G70610 | 1.49E-05 | 0.599 | - | - |
| AT3G43230 | 1.32E-05 | 0.599 | - | - |
| AT2G36950 | 1.24E-05 | 0.599 | - | - |
| AT2G26690 | 1.46E-05 | 0.6 | - | - |
| AT4G27070 | 1.34E-05 | 0.6 | - | - |
| AT2G43650 | 1.28E-05 | 0.6 | - | - |
| AT1G15250 | 1.15E-05 | 0.6 | - | - |
| AT4G19640 | 2.34E-05 | 0.601 | - | - |
| AT1G04020 | 1.46E-05 | 0.601 | - | - |
| AT1G01630 | 1.41E-05 | 0.601 | - | - |
| AT5G14620 | 1.11E-05 | 0.601 | - | - |
| AT2G18330 | 2.58E-05 | 0.602 | - | - |
| AT1G60490 | 2.49E-05 | 0.602 | - | - |
| AT1G33760 | 1.32E-05 | 0.602 | - | - |
| AT1G16610 | 1.11E-05 | 0.602 | - | - |
| AT4G15930 | 1.03E-05 | 0.602 | - | - |
| AT2G33435 | 1.03E-05 | 0.602 | - | - |
| AT5G59970 | 1.49E-05 | 0.603 | - | - |
| AT1G30680 | 1.43E-05 | 0.603 | - | - |
| AT1G02410 | 1.75E-05 | 0.604 | - | - |
| AT4G14250 | 1.11E-05 | 0.604 | - | - |
| AT1G64790 | 3.11E-05 | 0.605 | - | - |
| AT3G52090 | 2.50E-05 | 0.605 | - | - |
| AT4G28850 | 1.59E-05 | 0.605 | - | - |
| AT4G37070 | 1.37E-05 | 0.605 | - | - |
| AT5G21274 | 1.35E-05 | 0.605 | - | - |
| AT4G34570 | 3.87E-05 | 0.606 | - | - |
| AT3G47620 | 2.31E-05 | 0.606 | - | - |
| AT5G39740 | 1.99E-05 | 0.606 | - | - |
| AT4G39370 | 1.09E-05 | 0.606 | - | - |
| AT2G16780 | 1.03E-05 | 0.606 | - | - |
| AT5G48700 | 2.07E-05 | 0.607 | - | - |
| AT5G48030 | 1.70E-05 | 0.607 | - | - |
| AT4G35850 | 1.62E-05 | 0.607 | - | - |
| AT3G16190 | 1.52E-05 | 0.607 | - | - |
| AT2G47000 | 1.44E-05 | 0.607 | - | - |
| AT1G27310 | 1.11E-05 | 0.607 | - | - |
| AT2G19080 | 1.05E-05 | 0.607 | - | - |
| AT5G03030 | 1.04E-05 | 0.607 | - | - |
| AT4G35580 | 2.47E-05 | 0.608 | - | - |
| AT5G55200 | 2.41E-05 | 0.608 | - | - |
| AT5G65210 | 2.00E-05 | 0.608 | - | - |
| AT5G47910 | 1.53E-05 | 0.608 | - | - |
| AT1G73480 | 1.31E-05 | 0.608 | - | - |
| AT5G66750 | 2.72E-05 | 0.609 | - | - |
| AT2G07040 | 1.46E-05 | 0.609 | - | - |
| AT5G16470 | 1.24E-05 | 0.609 | - | - |
| AT4G37450 | 1.04E-05 | 0.61 | - | - |
| AT5G23670 | 1.03E-05 | 0.61 | - | - |
| AT2G14610 | 1.57E-05 | 0.611 | - | - |
| AT3G44530 | 1.22E-05 | 0.611 | - | - |
| AT5G61720 | 1.12E-05 | 0.611 | - | - |
| AT2G41380 | 1.06E-05 | 0.611 | - | - |
| AT4G00585 | 1.04E-05 | 0.611 | - | - |
| AT3G24010 | 1.82E-05 | 0.612 | - | - |
| AT1G61250 | 1.05E-05 | 0.612 | - | - |
| AT5G40240 | 1.02E-05 | 0.612 | - | - |
| AT4G23660 | 1.18E-05 | 0.613 | - | - |
| AT1G76400 | 2.43E-05 | 0.614 | - | - |
| AT3G01020 | 1.64E-05 | 0.614 | - | - |
| AT3G03600 | 1.49E-05 | 0.614 | - | - |
| AT5G55590 | 1.35E-05 | 0.614 | - | - |
| AT3G62110 | 1.30E-05 | 0.614 | - | - |
| AT2G38700 | 1.11E-05 | 0.614 | - | - |
| ATCG00190 | 2.37E-05 | 0.615 | - | - |
| AT3G53110 | 1.55E-05 | 0.615 | - | - |
| AT5G60960 | 1.44E-05 | 0.615 | - | - |
| AT5G23660 | 1.39E-05 | 0.615 | - | - |
| AT1G34510 | 1.26E-05 | 0.615 | - | - |
| AT5G12200 | 2.55E-05 | 0.616 | - | - |
| AT2G28290 | 2.24E-05 | 0.616 | - | - |
| AT1G69770 | 1.63E-05 | 0.616 | - | - |
| AT1G03520 | 1.53E-05 | 0.616 | - | - |
| AT1G71800 | 1.45E-05 | 0.616 | - | - |
| ATCG01240 | 1.44E-05 | 0.616 | - | - |
| AT5G19450 | 1.24E-05 | 0.616 | - | - |
| AT4G12550 | 1.16E-05 | 0.616 | - | - |
| AT3G04230 | 1.57E-05 | 0.617 | - | - |
| AT4G29160 | 2.12E-05 | 0.618 | - | - |
| AT1G20710 | 2.24E-05 | 0.619 | - | - |
| AT5G52490 | 1.84E-05 | 0.619 | - | - |
| AT3G18740 | 1.77E-05 | 0.619 | - | - |
| ATCG00900 | 1.44E-05 | 0.619 | - | - |
| AT2G26990 | 1.37E-05 | 0.619 | - | - |
| AT5G16830 | 2.07E-05 | 0.621 | - | - |
| AT3G51430 | 1.82E-05 | 0.621 | - | - |
| AT5G47880 | 1.76E-05 | 0.621 | - | - |
| AT4G17615 | 1.72E-05 | 0.621 | - | - |
| AT4G39740 | 1.53E-05 | 0.621 | - | - |
| AT2G39780 | 1.51E-05 | 0.621 | - | - |
| AT3G59300 | 1.09E-05 | 0.621 | - | - |
| AT1G30820 | 1.07E-05 | 0.621 | - | - |
| AT5G19860 | 1.02E-05 | 0.621 | - | - |
| AT5G54490 | 1.85E-05 | 0.623 | - | - |
| AT1G36310 | 1.30E-05 | 0.623 | - | - |
| AT4G02460 | 1.05E-05 | 0.623 | - | - |
| AT1G54490 | 2.28E-05 | 0.624 | - | - |
| AT1G69420 | 1.39E-05 | 0.624 | - | - |
| AT2G38250 | 1.09E-05 | 0.624 | - | - |
| AT1G68740 | 1.52E-05 | 0.625 | - | - |
| ATCG00830 | 1.27E-05 | 0.625 | - | - |
| AT4G39520 | 1.19E-05 | 0.625 | - | - |
| AT1G36730 | 1.05E-05 | 0.625 | - | - |
| AT3G62550 | 1.48E-05 | 0.626 | - | - |
| AT2G41010 | 1.22E-05 | 0.626 | - | - |
| AT3G06040 | 1.14E-05 | 0.626 | - | - |
| AT3G06680 | 1.73E-05 | 0.627 | - | - |
| AT1G59910 | 1.29E-05 | 0.628 | - | - |
| AT2G33290 | 1.11E-05 | 0.628 | - | - |
| AT2G43570 | 1.72E-05 | 0.629 | - | - |
| AT2G41640 | 1.48E-05 | 0.629 | - | - |
| AT3G46170 | 1.42E-05 | 0.629 | - | - |
| AT2G20000 | 1.42E-05 | 0.629 | - | - |
| AT2G17370 | 1.26E-05 | 0.629 | - | - |
| AT4G20410 | 1.06E-05 | 0.629 | - | - |
| AT1G16870 | 1.47E-05 | 0.63 | - | - |
| AT4G39830 | 1.44E-05 | 0.63 | - | - |
| AT5G65930 | 1.06E-05 | 0.63 | - | - |
| AT3G07910 | 1.04E-05 | 0.63 | - | - |
| AT5G13750 | 1.00E-05 | 0.63 | - | - |
| AT1G20700 | 3.29E-05 | 0.631 | - | - |
| AT5G17630 | 1.12E-05 | 0.631 | - | - |
| AT3G51130 | 1.67E-05 | 0.632 | - | - |
| AT5G45130 | 3.31E-05 | 0.633 | - | - |
| AT3G13310 | 1.71E-05 | 0.633 | - | - |
| AT5G51560 | 1.42E-05 | 0.633 | - | - |
| AT2G17500 | 1.86E-05 | 0.634 | - | - |
| AT4G32915 | 2.16E-05 | 0.635 | - | - |
| AT3G61620 | 1.70E-05 | 0.635 | - | - |
| AT1G13990 | 2.60E-05 | 0.636 | - | - |
| AT1G17760 | 1.61E-05 | 0.636 | - | - |
| AT5G63110 | 1.95E-05 | 0.637 | - | - |
| AT3G05200 | 1.61E-05 | 0.637 | - | - |
| AT5G63130 | 1.22E-05 | 0.637 | - | - |
| AT1G22480 | 1.11E-05 | 0.637 | - | - |
| AT3G20630 | 1.73E-05 | 0.638 | - | - |
| AT5G62520 | 1.14E-05 | 0.638 | - | - |
| AT5G09350 | 1.54E-05 | 0.639 | - | - |
| AT2G42870 | 1.14E-05 | 0.639 | - | - |
| AT4G24160 | 1.52E-05 | 0.64 | - | - |
| AT4G37010 | 1.51E-05 | 0.64 | - | - |
| AT5G60410 | 1.43E-05 | 0.64 | - | - |
| AT1G02160 | 1.13E-05 | 0.64 | - | - |
| AT5G26980 | 1.48E-05 | 0.641 | - | - |
| AT4G11360 | 1.15E-05 | 0.641 | - | - |
| AT3G18190 | 1.89E-05 | 0.643 | - | - |
| AT5G04930 | 1.52E-05 | 0.643 | - | - |
| AT1G54130 | 1.26E-05 | 0.643 | - | - |
| AT3G52400 | 2.44E-05 | 0.645 | - | - |
| AT1G28210 | 1.12E-05 | 0.645 | - | - |
| AT5G47600 | 1.27E-05 | 0.646 | - | - |
| AT1G28480 | 2.17E-05 | 0.647 | - | - |
| AT5G44240 | 1.53E-05 | 0.647 | - | - |
| AT2G27840 | 1.27E-05 | 0.647 | - | - |
| AT1G66270 | 1.06E-05 | 0.647 | - | - |
| AT4G23740 | 1.02E-05 | 0.647 | - | - |
| AT5G13330 | 1.07E-05 | 0.648 | - | - |
| AT5G12410 | 1.02E-05 | 0.649 | - | - |
| AT5G62410 | 2.26E-05 | 0.65 | - | - |
| AT1G08130 | 1.84E-05 | 0.65 | - | - |
| AT3G60840 | 1.30E-05 | 0.65 | - | - |
| AT3G60440 | 1.03E-05 | 0.65 | - | - |
| AT1G12820 | 1.01E-05 | 0.65 | - | - |
| AT2G42740 | 1.77E-05 | 0.651 | - | - |
| AT5G13480 | 3.27E-05 | 0.653 | - | - |
| AT2G03670 | 1.18E-05 | 0.653 | - | - |
| AT2G05060 | 1.15E-05 | 0.653 | - | - |
| AT2G36930 | 1.86E-05 | 0.654 | - | - |
| AT5G13840 | 1.42E-05 | 0.654 | - | - |
| AT3G06770 | 1.09E-05 | 0.654 | - | - |
| AT3G08720 | 3.09E-05 | 0.655 | - | - |
| AT2G17930 | 1.58E-05 | 0.655 | - | - |
| AT2G40700 | 1.48E-05 | 0.655 | - | - |
| AT1G60770 | 1.15E-05 | 0.655 | - | - |
| AT1G73600 | 1.07E-05 | 0.655 | - | - |
| AT1G44110 | 2.01E-05 | 0.656 | - | - |
| AT3G08950 | 1.89E-05 | 0.656 | - | - |
| AT3G54710 | 1.83E-05 | 0.656 | - | - |
| AT2G19830 | 1.46E-05 | 0.656 | - | - |
| AT1G69670 | 1.13E-05 | 0.656 | - | - |
| AT1G07070 | 2.05E-05 | 0.658 | - | - |
| AT2G35630 | 1.69E-05 | 0.658 | - | - |
| AT1G55890 | 1.26E-05 | 0.658 | - | - |
| AT4G17640 | 1.10E-05 | 0.658 | - | - |
| AT1G61520 | 3.50E-05 | 0.659 | - | - |
| AT1G08780 | 2.29E-05 | 0.659 | - | - |
| AT1G05100 | 1.60E-05 | 0.659 | - | - |
| AT4G20980 | 1.21E-05 | 0.659 | - | - |
| AT5G09920 | 2.24E-05 | 0.66 | - | - |
| AT5G38900 | 1.40E-05 | 0.66 | - | - |
| AT5G63600 | 1.36E-05 | 0.66 | - | - |
| AT2G22340 | 1.03E-05 | 0.66 | - | - |
| AT5G10660 | 1.00E-05 | 0.66 | - | - |
| AT3G01340 | 2.45E-05 | 0.661 | - | - |
| AT5G65110 | 1.96E-05 | 0.661 | - | - |
| AT3G03270 | 1.09E-05 | 0.661 | - | - |
| AT2G40270 | 1.04E-05 | 0.661 | - | - |
| AT2G47580 | 1.32E-05 | 0.662 | - | - |
| AT4G01250 | 1.29E-05 | 0.662 | - | - |
| AT1G75510 | 1.24E-05 | 0.662 | - | - |
| AT5G11270 | 1.16E-05 | 0.662 | - | - |
| AT3G11820 | 2.97E-05 | 0.663 | - | - |
| AT2G01810 | 1.69E-05 | 0.663 | - | - |
| AT3G10380 | 1.55E-05 | 0.663 | - | - |
| AT1G80770 | 1.85E-05 | 0.664 | - | - |
| AT5G27280 | 1.36E-05 | 0.664 | - | - |
| AT1G48360 | 1.21E-05 | 0.664 | - | - |
| AT4G36180 | 1.20E-05 | 0.665 | - | - |
| AT2G14890 | 1.18E-05 | 0.665 | - | - |
| AT1G67280 | 1.70E-05 | 0.666 | - | - |
| AT3G58160 | 1.34E-05 | 0.666 | - | - |
| AT2G24550 | 1.03E-05 | 0.666 | - | - |
| AT5G06150 | 1.92E-05 | 0.667 | - | - |
| AT1G77450 | 1.82E-05 | 0.667 | - | - |
| AT2G44065 | 1.79E-05 | 0.667 | - | - |
| AT4G26650 | 1.37E-05 | 0.667 | - | - |
| AT2G27950 | 1.22E-05 | 0.667 | - | - |
| AT1G06270 | 1.12E-05 | 0.667 | - | - |
| AT1G47490 | 1.02E-05 | 0.667 | - | - |
| AT2G37990 | 2.94E-05 | 0.668 | - | - |
| AT4G22220 | 2.62E-05 | 0.668 | - | - |
| AT3G62230 | 1.54E-05 | 0.668 | - | - |
| AT5G23880 | 1.41E-05 | 0.668 | - | - |
| AT1G80350 | 1.22E-05 | 0.668 | - | - |
| AT4G14365 | 2.48E-05 | 0.669 | - | - |
| AT5G11200 | 1.51E-05 | 0.67 | - | - |
| ATMG00560 | 1.26E-05 | 0.67 | - | - |
| AT5G44750 | 1.03E-05 | 0.67 | - | - |
| AT2G19540 | 3.22E-05 | 0.671 | - | - |
| AT1G21900 | 1.28E-05 | 0.671 | - | - |
| ATCG00420 | 1.45E-05 | 0.673 | - | - |
| ATMG01270 | 1.25E-05 | 0.673 | - | - |
| AT4G27410 | 1.56E-05 | 0.674 | - | - |
| AT5G47230 | 1.21E-05 | 0.674 | - | - |
| AT1G74560 | 1.80E-05 | 0.675 | - | - |
| AT1G56280 | 1.56E-05 | 0.675 | - | - |
| AT3G25150 | 1.31E-05 | 0.675 | - | - |
| AT2G15580 | 1.20E-05 | 0.675 | - | - |
| AT1G02890 | 1.11E-05 | 0.675 | - | - |
| AT5G26340 | 1.45E-05 | 0.676 | - | - |
| AT1G05720 | 1.26E-05 | 0.676 | - | - |
| AT2G41160 | 1.10E-05 | 0.676 | - | - |
| AT4G30890 | 1.81E-05 | 0.677 | - | - |
| AT1G49910 | 1.43E-05 | 0.677 | - | - |
| AT1G25260 | 2.99E-05 | 0.678 | - | - |
| AT1G28960 | 1.04E-05 | 0.678 | - | - |
| AT4G32150 | 1.49E-05 | 0.679 | - | - |
| AT5G17020 | 2.00E-05 | 0.68 | - | - |
| AT3G10140 | 1.85E-05 | 0.68 | - | - |
| AT1G01140 | 1.12E-05 | 0.68 | - | - |
| AT3G20670 | 1.03E-05 | 0.68 | - | - |
| AT2G05920 | 2.43E-05 | 0.681 | - | - |
| AT3G26560 | 2.21E-05 | 0.681 | - | - |
| AT5G11770 | 2.11E-05 | 0.681 | - | - |
| AT1G68820 | 1.54E-05 | 0.681 | - | - |
| AT3G01190 | 1.78E-05 | 0.682 | - | - |
| AT1G61010 | 1.22E-05 | 0.682 | - | - |
| AT5G07410 | 1.15E-05 | 0.682 | - | - |
| AT2G23680 | 1.04E-05 | 0.682 | - | - |
| AT3G62970 | 1.08E-05 | 0.683 | - | - |
| AT5G23290 | 2.60E-05 | 0.684 | - | - |
| AT4G04080 | 1.65E-05 | 0.684 | - | - |
| AT1G80630 | 1.42E-05 | 0.684 | - | - |
| AT4G33920 | 1.37E-05 | 0.684 | - | - |
| AT2G29540 | 1.83E-05 | 0.685 | - | - |
| AT2G16370 | 4.47E-05 | 0.686 | - | - |
| AT3G19760 | 1.78E-05 | 0.686 | - | - |
| AT3G23530 | 1.20E-05 | 0.686 | - | - |
| AT1G27630 | 1.10E-05 | 0.688 | - | - |
| AT1G54140 | 1.03E-05 | 0.688 | - | - |
| AT3G26830 | 1.82E-05 | 0.689 | - | - |
| AT4G34640 | 1.27E-05 | 0.689 | - | - |
| AT3G54750 | 1.64E-05 | 0.69 | - | - |
| AT4G25500 | 1.37E-05 | 0.69 | - | - |
| AT5G63650 | 1.21E-05 | 0.69 | - | - |
| AT5G22100 | 1.52E-05 | 0.691 | - | - |
| AT5G49060 | 1.42E-05 | 0.691 | - | - |
| AT3G13170 | 1.24E-05 | 0.691 | - | - |
| AT4G24690 | 1.88E-05 | 0.692 | - | - |
| AT5G10350 | 1.19E-05 | 0.692 | - | - |
| AT2G43490 | 1.08E-05 | 0.693 | - | - |
| AT1G54610 | 1.05E-05 | 0.693 | - | - |
| AT3G54180 | 2.23E-05 | 0.694 | - | - |
| AT3G22910 | 1.48E-05 | 0.694 | - | - |
| AT2G40610 | 1.22E-05 | 0.694 | - | - |
| AT3G48190 | 1.46E-05 | 0.695 | - | - |
| AT4G24970 | 1.75E-05 | 0.697 | - | - |
| AT1G65630 | 1.31E-05 | 0.697 | - | - |
| AT3G46620 | 1.23E-05 | 0.697 | - | - |
| AT3G49080 | 1.86E-05 | 0.698 | - | - |
| AT5G12090 | 1.13E-05 | 0.698 | - | - |
| AT4G38540 | 1.74E-05 | 0.7 | - | - |
| AT4G37370 | 1.45E-05 | 0.7 | - | - |
| AT4G18640 | 1.38E-05 | 0.7 | - | - |
| AT3G03300 | 1.32E-05 | 0.7 | - | - |
| AT2G19480 | 1.24E-05 | 0.7 | - | - |
| AT3G22880 | 1.23E-05 | 0.7 | - | - |
| AT1G30480 | 1.03E-05 | 0.7 | - | - |
| AT2G30710 | 1.34E-05 | 0.701 | - | - |
| AT5G50870 | 1.23E-05 | 0.701 | - | - |
| AT5G18270 | 1.03E-05 | 0.701 | - | - |
| AT2G44840 | 1.78E-05 | 0.703 | - | - |
| AT1G31150 | 1.17E-05 | 0.703 | - | - |
| AT2G18050 | 1.09E-05 | 0.703 | - | - |
| AT5G06160 | 3.16E-05 | 0.704 | - | - |
| AT1G05340 | 1.42E-05 | 0.704 | - | - |
| AT4G02450 | 1.40E-05 | 0.704 | - | - |
| AT5G06550 | 1.13E-05 | 0.704 | - | - |
| AT1G14540 | 1.61E-05 | 0.705 | - | - |
| AT1G11960 | 1.05E-05 | 0.705 | - | - |
| AT5G09420 | 1.02E-05 | 0.705 | - | - |
| AT3G27080 | 1.54E-05 | 0.706 | - | - |
| AT3G55840 | 1.51E-05 | 0.706 | - | - |
| AT5G46250 | 1.20E-05 | 0.706 | - | - |
| AT2G32150 | 1.02E-05 | 0.706 | - | - |
| AT4G12570 | 2.77E-05 | 0.707 | - | - |
| AT5G20350 | 1.09E-05 | 0.707 | - | - |
| AT4G25550 | 1.07E-05 | 0.707 | - | - |
| AT2G36130 | 1.05E-05 | 0.707 | - | - |
| AT4G39890 | 2.40E-05 | 0.709 | - | - |
| AT4G34110 | 1.05E-05 | 0.709 | - | - |
| AT3G55980 | 1.32E-05 | 0.71 | - | - |
| AT5G05990 | 1.09E-05 | 0.71 | - | - |
| AT2G04630 | 1.55E-05 | 0.711 | - | - |
| AT5G14800 | 1.51E-05 | 0.711 | - | - |
| AT2G33440 | 1.13E-05 | 0.711 | - | - |
| AT4G12600 | 2.56E-05 | 0.712 | - | - |
| AT1G71790 | 2.02E-05 | 0.712 | - | - |
| AT3G22310 | 1.40E-05 | 0.712 | - | - |
| AT3G12370 | 1.39E-05 | 0.712 | - | - |
| AT5G28220 | 1.38E-05 | 0.713 | - | - |
| AT4G16630 | 1.20E-05 | 0.713 | - | - |
| AT1G08920 | 1.10E-05 | 0.713 | - | - |
| AT3G18520 | 1.10E-05 | 0.713 | - | - |
| AT1G80410 | 1.08E-05 | 0.713 | - | - |
| AT3G44590 | 2.25E-05 | 0.714 | - | - |
| AT3G13580 | 1.80E-05 | 0.714 | - | - |
| AT5G58030 | 1.31E-05 | 0.714 | - | - |
| AT2G07696 | 1.26E-05 | 0.714 | - | - |
| AT3G03340 | 1.24E-05 | 0.714 | - | - |
| AT3G09700 | 1.23E-05 | 0.714 | - | - |
| AT4G36810 | 1.74E-05 | 0.715 | - | - |
| AT3G15500 | 1.09E-05 | 0.716 | - | - |
| AT1G78530 | 1.30E-05 | 0.717 | - | - |
| AT1G79050 | 1.25E-05 | 0.717 | - | - |
| AT2G19330 | 1.06E-05 | 0.717 | - | - |
| AT1G55900 | 1.65E-05 | 0.718 | - | - |
| AT3G25070 | 1.32E-05 | 0.718 | - | - |
| AT1G79730 | 1.19E-05 | 0.718 | - | - |
| AT1G03000 | 1.17E-05 | 0.718 | - | - |
| AT2G43770 | 1.16E-05 | 0.718 | - | - |
| AT3G13970 | 1.09E-05 | 0.718 | - | - |
| AT1G10140 | 1.73E-05 | 0.719 | - | - |
| AT5G52040 | 1.58E-05 | 0.719 | - | - |
| AT5G45900 | 1.29E-05 | 0.719 | - | - |
| AT5G48870 | 1.60E-05 | 0.72 | - | - |
| AT1G76460 | 1.32E-05 | 0.72 | - | - |
| AT2G23260 | 1.19E-05 | 0.72 | - | - |
| AT1G44350 | 1.14E-05 | 0.72 | - | - |
| AT5G08220 | 1.08E-05 | 0.72 | - | - |
| AT5G64870 | 1.16E-05 | 0.721 | - | - |
| AT4G02790 | 2.26E-05 | 0.722 | - | - |
| AT2G24000 | 1.80E-05 | 0.723 | - | - |
| AT5G58350 | 1.67E-05 | 0.723 | - | - |
| AT3G53640 | 1.24E-05 | 0.723 | - | - |
| AT1G26420 | 1.58E-05 | 0.724 | - | - |
| AT1G66410 | 1.28E-05 | 0.724 | - | - |
| AT5G18620 | 1.26E-05 | 0.724 | - | - |
| AT1G79650 | 1.13E-05 | 0.724 | - | - |
| AT2G45170 | 2.17E-05 | 0.725 | - | - |
| AT1G28370 | 1.94E-05 | 0.725 | - | - |
| AT4G35310 | 1.29E-05 | 0.725 | - | - |
| AT3G55170 | 1.58E-05 | 0.726 | - | - |
| AT3G16850 | 1.03E-05 | 0.726 | - | - |
| AT1G29940 | 3.23E-05 | 0.727 | - | - |
| AT5G05410 | 1.37E-05 | 0.727 | - | - |
| AT1G55450 | 1.09E-05 | 0.727 | - | - |
| AT1G72440 | 3.92E-05 | 0.728 | - | - |
| AT1G25490 | 2.03E-05 | 0.728 | - | - |
| AT2G27600 | 1.93E-05 | 0.729 | - | - |
| AT5G59180 | 1.71E-05 | 0.729 | - | - |
| AT1G16740 | 1.11E-05 | 0.729 | - | - |
| AT2G30750 | 1.39E-05 | 0.73 | - | - |
| AT1G34370 | 1.45E-05 | 0.731 | - | - |
| AT4G17690 | 1.03E-05 | 0.731 | - | - |
| AT2G39740 | 1.58E-05 | 0.732 | - | - |
| AT3G61860 | 1.43E-05 | 0.732 | - | - |
| AT3G13180 | 2.18E-05 | 0.733 | - | - |
| AT4G30220 | 2.04E-05 | 0.733 | - | - |
| AT3G10640 | 1.28E-05 | 0.733 | - | - |
| AT5G19780 | 1.08E-05 | 0.733 | - | - |
| AT4G27680 | 1.00E-05 | 0.733 | - | - |
| AT3G11130 | 2.87E-05 | 0.734 | - | - |
| AT1G61570 | 2.13E-05 | 0.734 | - | - |
| AT2G18700 | 1.49E-05 | 0.734 | - | - |
| AT5G38830 | 1.02E-05 | 0.734 | - | - |
| AT5G22330 | 2.30E-05 | 0.735 | - | - |
| AT1G09080 | 2.25E-05 | 0.735 | - | - |
| AT1G22840 | 4.07E-05 | 0.736 | - | - |
| AT5G07960 | 1.54E-05 | 0.736 | - | - |
| AT5G24150 | 1.34E-05 | 0.736 | - | - |
| AT5G22080 | 1.34E-05 | 0.736 | - | - |
| AT4G14110 | 1.16E-05 | 0.736 | - | - |
| AT2G41430 | 1.17E-05 | 0.737 | - | - |
| AT3G16760 | 1.14E-05 | 0.737 | - | - |
| AT4G31360 | 1.00E-05 | 0.737 | - | - |
| AT1G77710 | 1.29E-05 | 0.738 | - | - |
| AT2G15960 | 1.09E-05 | 0.738 | - | - |
| AT4G11420 | 3.06E-05 | 0.739 | - | - |
| AT5G66210 | 2.51E-05 | 0.739 | - | - |
| AT4G29730 | 1.07E-05 | 0.741 | - | - |
| AT1G73690 | 1.14E-05 | 0.742 | - | - |
| AT5G66170 | 1.08E-05 | 0.742 | - | - |
| AT1G63750 | 1.07E-05 | 0.742 | - | - |
| AT5G60460 | 2.08E-05 | 0.744 | - | - |
| AT1G74710 | 1.85E-05 | 0.744 | - | - |
| AT3G02760 | 1.26E-05 | 0.744 | - | - |
| AT1G20960 | 1.98E-05 | 0.745 | - | - |
| AT2G31350 | 1.90E-05 | 0.745 | - | - |
| AT2G02710 | 1.81E-05 | 0.745 | - | - |
| AT5G48380 | 1.80E-05 | 0.745 | - | - |
| AT2G02570 | 1.48E-05 | 0.745 | - | - |
| AT1G80840 | 2.44E-05 | 0.746 | - | - |
| AT3G15630 | 1.35E-05 | 0.747 | - | - |
| AT1G73980 | 1.14E-05 | 0.747 | - | - |
| AT5G63190 | 1.71E-05 | 0.748 | - | - |
| AT4G35140 | 1.31E-05 | 0.748 | - | - |
| AT5G56280 | 1.28E-05 | 0.748 | - | - |
| AT5G35080 | 1.02E-05 | 0.748 | - | - |
| AT2G40770 | 1.41E-05 | 0.749 | - | - |
| AT1G18250 | 1.82E-05 | 0.75 | - | - |
| AT5G48970 | 1.44E-05 | 0.75 | - | - |
| AT3G07010 | 1.31E-05 | 0.75 | - | - |
| AT5G60590 | 1.22E-05 | 0.75 | - | - |
| AT5G64200 | 1.22E-05 | 0.75 | - | - |
| AT1G54690 | 1.19E-05 | 0.75 | - | - |
| AT1G77140 | 1.08E-05 | 0.75 | - | - |
| AT3G01920 | 1.21E-05 | 0.751 | - | - |
| AT2G06210 | 1.16E-05 | 0.751 | - | - |
| AT1G08050 | 1.59E-05 | 0.752 | - | - |
| AT1G68410 | 1.35E-05 | 0.752 | - | - |
| AT4G02350 | 1.14E-05 | 0.752 | - | - |
| AT4G33905 | 1.37E-05 | 0.753 | - | - |
| AT5G01770 | 1.18E-05 | 0.753 | - | - |
| AT2G17290 | 1.57E-05 | 0.754 | - | - |
| AT2G05950 | 1.14E-05 | 0.754 | - | - |
| AT4G10500 | 1.08E-05 | 0.754 | - | - |
| AT3G13390 | 1.37E-05 | 0.755 | - | - |
| AT5G59550 | 1.30E-05 | 0.755 | - | - |
| AT1G18850 | 1.17E-05 | 0.755 | - | - |
| AT2G47040 | 1.01E-05 | 0.755 | - | - |
| AT1G16240 | 2.10E-05 | 0.757 | - | - |
| AT4G18880 | 1.86E-05 | 0.757 | - | - |
| AT5G43960 | 1.08E-05 | 0.758 | - | - |
| AT4G05010 | 1.05E-05 | 0.758 | - | - |
| AT1G01720 | 1.21E-05 | 0.759 | - | - |
| AT3G44340 | 1.12E-05 | 0.759 | - | - |
| AT5G14530 | 1.04E-05 | 0.76 | - | - |
| AT3G02550 | 1.01E-05 | 0.76 | - | - |
| AT3G50360 | 1.78E-05 | 0.761 | - | - |
| AT5G58970 | 1.58E-05 | 0.761 | - | - |
| AT5G25440 | 1.57E-05 | 0.761 | - | - |
| AT1G49810 | 1.28E-05 | 0.761 | - | - |
| AT2G37230 | 1.06E-05 | 0.761 | - | - |
| AT1G56220 | 1.55E-05 | 0.762 | - | - |
| AT1G01820 | 1.06E-05 | 0.762 | - | - |
| AT5G54840 | 1.71E-05 | 0.763 | - | - |
| AT5G02530 | 2.18E-05 | 0.764 | - | - |
| AT5G15520 | 1.75E-05 | 0.764 | - | - |
| AT1G12960 | 1.75E-05 | 0.764 | - | - |
| AT1G11880 | 1.49E-05 | 0.764 | - | - |
| AT1G77830 | 1.14E-05 | 0.764 | - | - |
| AT3G16320 | 1.17E-05 | 0.765 | - | - |
| AT1G48230 | 1.09E-05 | 0.765 | - | - |
| AT4G36480 | 1.23E-05 | 0.766 | - | - |
| AT3G49960 | 1.04E-05 | 0.766 | - | - |
| AT5G18630 | 1.17E-05 | 0.767 | - | - |
| AT4G09140 | 1.15E-05 | 0.767 | - | - |
| AT5G08610 | 1.15E-05 | 0.767 | - | - |
| AT5G09250 | 1.74E-05 | 0.768 | - | - |
| AT5G63640 | 1.30E-05 | 0.768 | - | - |
| AT5G27640 | 1.68E-05 | 0.769 | - | - |
| AT3G46080 | 1.27E-05 | 0.769 | - | - |
| AT3G29680 | 1.11E-05 | 0.769 | - | - |
| AT3G26320 | 1.43E-05 | 0.77 | - | - |
| AT2G36100 | 1.17E-05 | 0.77 | - | - |
| AT5G15510 | 1.13E-05 | 0.771 | - | - |
| AT5G49510 | 2.06E-05 | 0.772 | - | - |
| AT2G47970 | 1.12E-05 | 0.772 | - | - |
| AT5G05230 | 1.05E-05 | 0.772 | - | - |
| AT3G32920 | 1.02E-05 | 0.772 | - | - |
| AT1G70590 | 1.56E-05 | 0.773 | - | - |
| AT1G24150 | 1.06E-05 | 0.773 | - | - |
| AT1G67480 | 1.04E-05 | 0.773 | - | - |
| AT3G16980 | 1.20E-05 | 0.774 | - | - |
| AT3G62680 | 1.02E-05 | 0.774 | - | - |
| AT2G33730 | 1.21E-05 | 0.775 | - | - |
| AT5G44730 | 1.15E-05 | 0.775 | - | - |
| AT4G32930 | 1.09E-05 | 0.775 | - | - |
| AT4G33260 | 1.26E-05 | 0.776 | - | - |
| AT3G18760 | 1.07E-05 | 0.776 | - | - |
| AT5G45800 | 1.15E-05 | 0.777 | - | - |
| AT1G60850 | 2.82E-05 | 0.778 | - | - |
| AT5G41010 | 2.19E-05 | 0.778 | - | - |
| AT5G39670 | 1.76E-05 | 0.778 | - | - |
| AT1G10430 | 1.64E-05 | 0.778 | - | - |
| AT5G54640 | 1.42E-05 | 0.778 | - | - |
| AT1G32920 | 1.20E-05 | 0.778 | - | - |
| AT1G09760 | 1.96E-05 | 0.779 | - | - |
| AT5G61600 | 1.16E-05 | 0.779 | - | - |
| AT2G44850 | 1.15E-05 | 0.779 | - | - |
| AT2G25160 | 1.45E-05 | 0.78 | - | - |
| AT4G17230 | 1.32E-05 | 0.78 | - | - |
| AT1G44170 | 1.27E-05 | 0.78 | - | - |
| AT2G21240 | 1.14E-05 | 0.78 | - | - |
| AT1G76070 | 1.11E-05 | 0.78 | - | - |
| AT1G66880 | 1.38E-05 | 0.781 | - | - |
| AT1G17720 | 2.05E-05 | 0.782 | - | - |
| AT1G68780 | 1.58E-05 | 0.782 | - | - |
| AT3G44260 | 1.36E-05 | 0.782 | - | - |
| AT4G12610 | 1.35E-05 | 0.782 | - | - |
| AT5G63070 | 1.31E-05 | 0.782 | - | - |
| AT3G06420 | 1.15E-05 | 0.782 | - | - |
| AT5G06410 | 1.07E-05 | 0.783 | - | - |
| AT2G02760 | 2.03E-05 | 0.784 | - | - |
| AT2G30600 | 1.18E-05 | 0.784 | - | - |
| AT5G67300 | 1.16E-05 | 0.784 | - | - |
| AT1G22790 | 1.04E-05 | 0.784 | - | - |
| AT3G25800 | 1.17E-05 | 0.785 | - | - |
| AT2G13540 | 3.24E-05 | 0.786 | - | - |
| AT1G66680 | 1.30E-05 | 0.786 | - | - |
| AT1G75200 | 1.25E-05 | 0.786 | - | - |
| AT4G27250 | 1.33E-05 | 0.787 | - | - |
| AT5G59120 | 1.23E-05 | 0.787 | - | - |
| AT3G03090 | 1.56E-05 | 0.788 | - | - |
| AT5G03210 | 1.30E-05 | 0.788 | - | - |
| AT2G36740 | 1.23E-05 | 0.788 | - | - |
| AT5G20160 | 2.27E-05 | 0.789 | - | - |
| AT4G37360 | 1.66E-05 | 0.789 | - | - |
| AT4G30600 | 1.38E-05 | 0.789 | - | - |
| AT3G43520 | 1.32E-05 | 0.789 | - | - |
| AT1G55510 | 3.44E-05 | 0.79 | - | - |
| AT5G58060 | 1.44E-05 | 0.79 | - | - |
| AT4G36690 | 1.66E-05 | 0.791 | - | - |
| AT3G11490 | 1.17E-05 | 0.791 | - | - |
| AT5G11650 | 1.10E-05 | 0.791 | - | - |
| AT2G40640 | 1.05E-05 | 0.791 | - | - |
| AT1G48570 | 1.37E-05 | 0.792 | - | - |
| AT5G23450 | 1.27E-05 | 0.792 | - | - |
| AT3G51830 | 1.07E-05 | 0.792 | - | - |
| AT4G35800 | 3.23E-05 | 0.793 | - | - |
| AT2G27110 | 1.40E-05 | 0.793 | - | - |
| AT3G15980 | 1.15E-05 | 0.793 | - | - |
| AT5G59840 | 1.04E-05 | 0.793 | - | - |
| AT5G15750 | 2.58E-05 | 0.794 | - | - |
| AT3G18090 | 1.77E-05 | 0.794 | - | - |
| AT1G22800 | 1.38E-05 | 0.794 | - | - |
| AT2G30050 | 1.31E-05 | 0.794 | - | - |
| AT1G25400 | 1.29E-05 | 0.794 | - | - |
| AT1G76970 | 1.22E-05 | 0.794 | - | - |
| AT5G05470 | 1.42E-05 | 0.795 | - | - |
| AT5G22290 | 4.03E-05 | 0.796 | - | - |
| AT1G76650 | 2.02E-05 | 0.796 | - | - |
| AT2G03150 | 1.58E-05 | 0.796 | - | - |
| AT4G20380 | 1.19E-05 | 0.796 | - | - |
| AT2G30360 | 1.05E-05 | 0.796 | - | - |
| AT1G27770 | 1.83E-05 | 0.797 | - | - |
| AT2G31170 | 1.75E-05 | 0.797 | - | - |
| AT1G27650 | 1.06E-05 | 0.797 | - | - |
| AT2G27250 | 1.47E-05 | 0.798 | - | - |
| AT1G80940 | 1.07E-05 | 0.799 | - | - |
| AT2G40140 | 1.55E-05 | 0.801 | - | - |
| AT2G46610 | 1.37E-05 | 0.801 | - | - |
| AT4G36040 | 1.48E-05 | 0.802 | - | - |
| AT4G20440 | 1.20E-05 | 0.802 | - | - |
| AT3G04770 | 2.88E-05 | 0.803 | - | - |
| AT5G04240 | 1.87E-05 | 0.803 | - | - |
| AT4G18840 | 1.03E-05 | 0.803 | - | - |
| AT3G23780 | 1.82E-05 | 0.804 | - | - |
| AT3G14050 | 1.67E-05 | 0.804 | - | - |
| AT5G64670 | 1.44E-05 | 0.804 | - | - |
| AT3G53230 | 1.02E-05 | 0.804 | - | - |
| AT2G18040 | 2.19E-05 | 0.805 | - | - |
| AT1G13140 | 1.04E-05 | 0.805 | - | - |
| AT1G08560 | 2.41E-05 | 0.807 | - | - |
| AT1G13210 | 1.50E-05 | 0.807 | - | - |
| AT5G52270 | 1.21E-05 | 0.807 | - | - |
| AT5G44740 | 1.18E-05 | 0.808 | - | - |
| AT1G11920 | 1.17E-05 | 0.808 | - | - |
| AT3G58180 | 1.02E-05 | 0.808 | - | - |
| AT3G08530 | 2.95E-05 | 0.809 | - | - |
| AT3G61190 | 1.58E-05 | 0.809 | - | - |
| AT3G47690 | 1.50E-05 | 0.809 | - | - |
| AT1G79590 | 1.48E-05 | 0.809 | - | - |
| AT5G03520 | 1.24E-05 | 0.809 | - | - |
| AT3G21630 | 1.02E-05 | 0.809 | - | - |
| AT3G51000 | 2.00E-05 | 0.81 | - | - |
| AT1G10840 | 1.96E-05 | 0.81 | - | - |
| AT2G19490 | 1.18E-05 | 0.81 | - | - |
| AT1G04780 | 1.17E-05 | 0.81 | - | - |
| AT5G51120 | 1.04E-05 | 0.81 | - | - |
| AT1G17530 | 1.39E-05 | 0.811 | - | - |
| AT5G60900 | 1.07E-05 | 0.811 | - | - |
| AT2G42550 | 1.06E-05 | 0.811 | - | - |
| AT2G26530 | 1.03E-05 | 0.811 | - | - |
| AT5G37370 | 1.13E-05 | 0.812 | - | - |
| AT1G69070 | 2.23E-05 | 0.813 | - | - |
| AT5G63030 | 1.12E-05 | 0.813 | - | - |
| AT3G20830 | 1.09E-05 | 0.813 | - | - |
| AT3G10500 | 1.50E-05 | 0.814 | - | - |
| AT3G63000 | 1.05E-05 | 0.814 | - | - |
| AT3G48750 | 2.58E-05 | 0.815 | - | - |
| AT4G33170 | 1.37E-05 | 0.815 | - | - |
| AT4G26760 | 1.06E-05 | 0.815 | - | - |
| AT4G34150 | 1.59E-05 | 0.816 | - | - |
| AT5G19320 | 1.36E-05 | 0.816 | - | - |
| AT2G32790 | 1.28E-05 | 0.816 | - | - |
| AT2G42450 | 1.16E-05 | 0.816 | - | - |
| AT5G60070 | 1.09E-05 | 0.816 | - | - |
| AT2G34800 | 1.04E-05 | 0.816 | - | - |
| AT1G16430 | 1.04E-05 | 0.816 | - | - |
| AT5G38290 | 1.41E-05 | 0.817 | - | - |
| AT5G18790 | 1.28E-05 | 0.817 | - | - |
| AT1G17980 | 2.24E-05 | 0.818 | - | - |
| AT1G59990 | 1.63E-05 | 0.818 | - | - |
| AT5G10630 | 1.40E-05 | 0.818 | - | - |
| AT5G09550 | 1.05E-05 | 0.818 | - | - |
| AT5G44340 | 1.78E-05 | 0.819 | - | - |
| AT2G37340 | 1.76E-05 | 0.819 | - | - |
| AT3G06290 | 1.57E-05 | 0.819 | - | - |
| AT4G24380 | 1.30E-05 | 0.819 | - | - |
| AT3G43920 | 1.29E-05 | 0.819 | - | - |
| AT3G12160 | 1.27E-05 | 0.819 | - | - |
| AT4G16420 | 1.24E-05 | 0.819 | - | - |
| AT1G68420 | 1.08E-05 | 0.819 | - | - |
| AT3G03430 | 1.06E-05 | 0.819 | - | - |
| AT2G42750 | 1.18E-05 | 0.82 | - | - |
| AT5G61030 | 1.07E-05 | 0.82 | - | - |
| AT4G19000 | 1.03E-05 | 0.82 | - | - |
| AT1G04870 | 2.22E-05 | 0.821 | - | - |
| AT5G44635 | 1.39E-05 | 0.821 | - | - |
| AT5G17250 | 1.13E-05 | 0.821 | - | - |
| AT3G54840 | 1.98E-05 | 0.822 | - | - |
| AT3G12990 | 1.64E-05 | 0.822 | - | - |
| AT4G16970 | 1.24E-05 | 0.822 | - | - |
| AT2G13370 | 1.20E-05 | 0.822 | - | - |
| AT5G03480 | 1.12E-05 | 0.822 | - | - |
| AT2G41500 | 1.90E-05 | 0.823 | - | - |
| AT5G47010 | 1.26E-05 | 0.823 | - | - |
| AT1G56660 | 1.18E-05 | 0.823 | - | - |
| AT5G16140 | 1.18E-05 | 0.823 | - | - |
| AT1G05220 | 1.10E-05 | 0.823 | - | - |
| AT5G10695 | 1.57E-05 | 0.824 | - | - |
| AT2G27080 | 1.30E-05 | 0.824 | - | - |
| AT2G40420 | 1.50E-05 | 0.825 | - | - |
| AT5G63750 | 1.08E-05 | 0.825 | - | - |
| AT4G02150 | 1.04E-05 | 0.825 | - | - |
| AT3G05790 | 1.65E-05 | 0.826 | - | - |
| AT4G23190 | 1.12E-05 | 0.826 | - | - |
| AT3G12590 | 1.54E-05 | 0.827 | - | - |
| AT5G62500 | 1.53E-05 | 0.827 | - | - |
| AT4G30430 | 1.09E-05 | 0.827 | - | - |
| AT5G40690 | 1.35E-05 | 0.828 | - | - |
| AT5G02400 | 1.27E-05 | 0.828 | - | - |
| AT1G57820 | 1.23E-05 | 0.828 | - | - |
| AT2G17510 | 1.14E-05 | 0.828 | - | - |
| AT3G18790 | 1.29E-05 | 0.829 | - | - |
| AT2G19670 | 1.86E-05 | 0.83 | - | - |
| AT3G58660 | 2.05E-05 | 0.831 | - | - |
| AT1G30870 | 1.55E-05 | 0.831 | - | - |
| ATCG01110 | 1.02E-05 | 0.831 | - | - |
| AT5G62190 | 1.02E-05 | 0.831 | - | - |
| AT1G20580 | 1.70E-05 | 0.832 | - | - |
| AT5G22410 | 1.18E-05 | 0.832 | - | - |
| AT5G24160 | 1.18E-05 | 0.832 | - | - |
| AT5G03870 | 1.01E-05 | 0.832 | - | - |
| AT4G13780 | 3.32E-05 | 0.833 | - | - |
| AT3G25610 | 2.45E-05 | 0.833 | - | - |
| AT2G01180 | 1.86E-05 | 0.833 | - | - |
| AT5G67080 | 1.13E-05 | 0.833 | - | - |
| AT5G49930 | 1.12E-05 | 0.833 | - | - |
| AT3G12280 | 2.73E-05 | 0.834 | - | - |
| AT3G04740 | 1.53E-05 | 0.834 | - | - |
| AT3G12640 | 4.27E-05 | 0.835 | - | - |
| AT1G72070 | 1.55E-05 | 0.835 | - | - |
| AT5G35520 | 1.30E-05 | 0.835 | - | - |
| AT1G49050 | 1.15E-05 | 0.835 | - | - |
| AT3G58030 | 1.09E-05 | 0.835 | - | - |
| AT1G66160 | 2.02E-05 | 0.836 | - | - |
| AT1G63440 | 1.73E-05 | 0.836 | - | - |
| AT4G05420 | 1.20E-05 | 0.836 | - | - |
| AT5G20020 | 1.62E-05 | 0.837 | - | - |
| AT1G77370 | 1.48E-05 | 0.837 | - | - |
| ATMG00510 | 1.15E-05 | 0.837 | - | - |
| AT3G48530 | 2.10E-05 | 0.838 | - | - |
| AT2G40660 | 1.66E-05 | 0.838 | - | - |
| AT1G77180 | 2.98E-05 | 0.839 | - | - |
| AT3G52430 | 1.70E-05 | 0.839 | - | - |
| AT5G61530 | 1.53E-05 | 0.839 | - | - |
| AT4G13830 | 1.31E-05 | 0.839 | - | - |
| AT1G50490 | 1.02E-05 | 0.839 | - | - |
| AT3G61240 | 1.12E-05 | 0.841 | - | - |
| AT3G50950 | 1.02E-05 | 0.841 | - | - |
| AT2G42710 | 1.39E-05 | 0.842 | - | - |
| AT3G46930 | 1.24E-05 | 0.842 | - | - |
| AT3G26020 | 1.19E-05 | 0.842 | - | - |
| AT2G35890 | 1.11E-05 | 0.842 | - | - |
| AT5G52320 | 1.18E-05 | 0.843 | - | - |
| AT2G36170 | 2.40E-05 | 0.844 | - | - |
| AT5G25230 | 1.60E-05 | 0.844 | - | - |
| AT5G16040 | 1.43E-05 | 0.844 | - | - |
| AT5G17850 | 1.09E-05 | 0.844 | - | - |
| AT3G28210 | 1.08E-05 | 0.844 | - | - |
| AT1G63490 | 1.02E-05 | 0.844 | - | - |
| AT3G07260 | 4.03E-05 | 0.845 | - | - |
| AT3G06530 | 2.28E-05 | 0.845 | - | - |
| AT5G49580 | 1.20E-05 | 0.845 | - | - |
| AT3G51450 | 1.04E-05 | 0.846 | - | - |
| AT2G46500 | 1.88E-05 | 0.847 | - | - |
| AT3G50110 | 1.74E-05 | 0.847 | - | - |
| AT2G42160 | 1.18E-05 | 0.847 | - | - |
| AT5G63670 | 2.07E-05 | 0.848 | - | - |
| AT1G61340 | 2.02E-05 | 0.848 | - | - |
| AT4G34030 | 1.93E-05 | 0.848 | - | - |
| AT3G03110 | 1.56E-05 | 0.848 | - | - |
| AT5G60040 | 1.23E-05 | 0.848 | - | - |
| AT3G22845 | 1.17E-05 | 0.848 | - | - |
| AT1G76600 | 1.30E-05 | 0.849 | - | - |
| AT1G73820 | 1.13E-05 | 0.849 | - | - |
| AT2G29640 | 1.08E-05 | 0.849 | - | - |
| AT4G32760 | 1.06E-05 | 0.849 | - | - |
| AT4G11220 | 1.04E-05 | 0.849 | - | - |
| AT1G03360 | 1.77E-05 | 0.85 | - | - |
| AT4G22380 | 1.75E-05 | 0.85 | - | - |
| AT4G21070 | 1.17E-05 | 0.85 | - | - |
| AT1G31370 | 1.15E-05 | 0.85 | - | - |
| AT3G07680 | 1.11E-05 | 0.85 | - | - |
| AT1G14650 | 1.09E-05 | 0.85 | - | - |
| AT5G64270 | 3.05E-05 | 0.851 | - | - |
| AT5G14460 | 2.41E-05 | 0.851 | - | - |
| AT5G37720 | 1.62E-05 | 0.851 | - | - |
| AT1G08460 | 1.20E-05 | 0.851 | - | - |
| AT3G53710 | 1.19E-05 | 0.851 | - | - |
| AT3G23890 | 2.70E-05 | 0.852 | - | - |
| AT5G15810 | 1.58E-05 | 0.852 | - | - |
| AT1G19020 | 2.00E-05 | 0.854 | - | - |
| AT5G65260 | 1.61E-05 | 0.854 | - | - |
| AT1G57600 | 1.39E-05 | 0.854 | - | - |
| AT1G78980 | 1.04E-05 | 0.854 | - | - |
| AT2G18900 | 1.03E-05 | 0.854 | - | - |
| AT2G18380 | 1.40E-05 | 0.855 | - | - |
| AT2G03780 | 1.10E-05 | 0.855 | - | - |
| AT5G65250 | 1.03E-05 | 0.856 | - | - |
| AT1G33980 | 1.71E-05 | 0.857 | - | - |
| AT1G28060 | 1.46E-05 | 0.857 | - | - |
| AT2G34260 | 1.24E-05 | 0.857 | - | - |
| AT3G05380 | 1.12E-05 | 0.857 | - | - |
| AT3G07740 | 1.17E-05 | 0.858 | - | - |
| AT5G43500 | 1.09E-05 | 0.858 | - | - |
| AT5G61590 | 1.08E-05 | 0.858 | - | - |
| AT1G80920 | 1.77E-05 | 0.859 | - | - |
| AT5G66240 | 1.54E-05 | 0.859 | - | - |
| AT1G53690 | 1.20E-05 | 0.859 | - | - |
| AT4G16265 | 1.19E-05 | 0.859 | - | - |
| AT3G08620 | 1.13E-05 | 0.859 | - | - |
| AT3G09830 | 1.04E-05 | 0.859 | - | - |
| AT5G15400 | 1.40E-05 | 0.86 | - | - |
| AT4G12820 | 1.12E-05 | 0.86 | - | - |
| AT4G26440 | 1.05E-05 | 0.86 | - | - |
| AT3G18160 | 1.02E-05 | 0.86 | - | - |
| AT5G03920 | 1.24E-05 | 0.861 | - | - |
| AT2G24765 | 1.17E-05 | 0.861 | - | - |
| AT3G22320 | 2.11E-05 | 0.862 | - | - |
| AT1G11890 | 1.60E-05 | 0.862 | - | - |
| AT5G41970 | 1.37E-05 | 0.862 | - | - |
| AT2G25570 | 1.36E-05 | 0.862 | - | - |
| AT5G42380 | 1.31E-05 | 0.862 | - | - |
| AT2G01440 | 1.03E-05 | 0.862 | - | - |
| AT1G80750 | 1.70E-05 | 0.863 | - | - |
| AT1G19380 | 1.46E-05 | 0.863 | - | - |
| AT1G27300 | 1.86E-05 | 0.864 | - | - |
| AT1G57990 | 1.48E-05 | 0.864 | - | - |
| AT2G41630 | 1.96E-05 | 0.865 | - | - |
| AT1G09320 | 1.67E-05 | 0.865 | - | - |
| AT1G77290 | 1.32E-05 | 0.865 | - | - |
| AT4G34450 | 1.56E-05 | 0.866 | - | - |
| ATCG00790 | 1.29E-05 | 0.866 | - | - |
| AT1G80070 | 2.77E-05 | 0.867 | - | - |
| AT1G20930 | 1.36E-05 | 0.867 | - | - |
| AT3G59500 | 1.16E-05 | 0.867 | - | - |
| AT1G51380 | 1.12E-05 | 0.867 | - | - |
| AT2G16930 | 1.10E-05 | 0.867 | - | - |
| AT5G06360 | 2.45E-05 | 0.868 | - | - |
| AT5G51660 | 2.18E-05 | 0.869 | - | - |
| AT5G13080 | 1.70E-05 | 0.869 | - | - |
| AT1G14300 | 1.27E-05 | 0.87 | - | - |
| AT3G50000 | 1.20E-05 | 0.87 | - | - |
| AT1G63210 | 1.01E-05 | 0.87 | - | - |
| AT4G28450 | 1.89E-05 | 0.871 | - | - |
| AT3G04640 | 1.58E-05 | 0.871 | - | - |
| AT5G43970 | 1.55E-05 | 0.871 | - | - |
| AT4G21820 | 1.38E-05 | 0.871 | - | - |
| AT5G17560 | 1.14E-05 | 0.871 | - | - |
| AT4G25200 | 1.06E-05 | 0.871 | - | - |
| AT1G60620 | 1.76E-05 | 0.872 | - | - |
| AT1G07135 | 1.13E-05 | 0.872 | - | - |
| AT1G28380 | 1.06E-05 | 0.872 | - | - |
| AT3G07750 | 1.27E-05 | 0.873 | - | - |
| AT3G20090 | 1.12E-05 | 0.873 | - | - |
| AT3G23860 | 1.04E-05 | 0.873 | - | - |
| AT1G59830 | 1.12E-05 | 0.874 | - | - |
| AT2G16710 | 1.02E-05 | 0.874 | - | - |
| AT5G45430 | 1.88E-05 | 0.875 | - | - |
| AT3G46940 | 1.72E-05 | 0.875 | - | - |
| AT2G37410 | 1.57E-05 | 0.875 | - | - |
| AT2G42180 | 1.20E-05 | 0.875 | - | - |
| AT4G39850 | 2.50E-05 | 0.876 | - | - |
| AT3G49530 | 1.68E-05 | 0.876 | - | - |
| AT3G49430 | 1.27E-05 | 0.876 | - | - |
| AT2G02790 | 1.06E-05 | 0.876 | - | - |
| AT1G66070 | 1.71E-05 | 0.877 | - | - |
| AT4G29380 | 1.32E-05 | 0.877 | - | - |
| AT1G72040 | 1.21E-05 | 0.877 | - | - |
| AT5G67240 | 1.11E-05 | 0.877 | - | - |
| AT2G05720 | 1.08E-05 | 0.877 | - | - |
| AT4G24440 | 1.86E-05 | 0.878 | - | - |
| AT3G10730 | 1.70E-05 | 0.878 | - | - |
| AT5G66070 | 1.31E-05 | 0.878 | - | - |
| AT2G43900 | 1.20E-05 | 0.878 | - | - |
| AT5G45560 | 1.19E-05 | 0.878 | - | - |
| AT4G37490 | 1.33E-05 | 0.879 | - | - |
| AT3G54150 | 1.94E-05 | 0.88 | - | - |
| AT1G76300 | 1.69E-05 | 0.88 | - | - |
| AT5G14580 | 1.37E-05 | 0.88 | - | - |
| AT2G40290 | 1.28E-05 | 0.88 | - | - |
| AT5G20920 | 1.23E-05 | 0.88 | - | - |
| AT4G17730 | 1.07E-05 | 0.88 | - | - |
| AT4G26010 | 1.06E-05 | 0.88 | - | - |
| AT1G30890 | 1.51E-05 | 0.881 | - | - |
| AT2G22880 | 1.45E-05 | 0.881 | - | - |
| AT2G44860 | 2.72E-05 | 0.882 | - | - |
| AT5G42200 | 1.18E-05 | 0.882 | - | - |
| AT1G44050 | 1.04E-05 | 0.883 | - | - |
| AT5G13240 | 1.03E-05 | 0.883 | - | - |
| AT1G60660 | 1.03E-05 | 0.884 | - | - |
| AT2G42270 | 1.74E-05 | 0.885 | - | - |
| AT2G31340 | 1.56E-05 | 0.885 | - | - |
| AT5G26680 | 1.24E-05 | 0.885 | - | - |
| AT3G22590 | 2.41E-05 | 0.886 | - | - |
| AT3G57530 | 1.78E-05 | 0.886 | - | - |
| AT5G35690 | 1.09E-05 | 0.886 | - | - |
| AT1G67320 | 1.05E-05 | 0.886 | - | - |
| AT3G47480 | 1.22E-05 | 0.887 | - | - |
| AT2G17840 | 1.19E-05 | 0.887 | - | - |
| AT2G38910 | 1.02E-05 | 0.887 | - | - |
| AT1G48870 | 1.46E-05 | 0.888 | - | - |
| AT2G33580 | 1.35E-05 | 0.889 | - | - |
| AT5G13220 | 1.02E-05 | 0.889 | - | - |
| AT3G59080 | 1.40E-05 | 0.89 | - | - |
| AT4G32910 | 1.18E-05 | 0.89 | - | - |
| AT1G08930 | 1.67E-05 | 0.891 | - | - |
| AT5G22250 | 1.46E-05 | 0.891 | - | - |
| AT1G21370 | 1.00E-05 | 0.891 | - | - |
| AT5G63610 | 2.42E-05 | 0.892 | - | - |
| AT1G02840 | 1.51E-05 | 0.892 | - | - |
| AT5G52750 | 1.04E-05 | 0.892 | - | - |
| AT1G07040 | 1.48E-05 | 0.893 | - | - |
| AT2G29550 | 1.37E-05 | 0.893 | - | - |
| AT1G47900 | 1.18E-05 | 0.893 | - | - |
| AT1G68990 | 1.04E-05 | 0.893 | - | - |
| AT4G12620 | 2.22E-05 | 0.894 | - | - |
| AT1G19170 | 1.81E-05 | 0.894 | - | - |
| AT5G61900 | 1.71E-05 | 0.894 | - | - |
| AT3G25940 | 1.56E-05 | 0.894 | - | - |
| AT1G26665 | 1.11E-05 | 0.894 | - | - |
| AT4G33050 | 1.92E-05 | 0.895 | - | - |
| ATMG00080 | 1.30E-05 | 0.895 | - | - |
| AT1G42440 | 2.26E-05 | 0.896 | - | - |
| AT5G03460 | 1.95E-05 | 0.896 | - | - |
| AT4G38890 | 1.41E-05 | 0.896 | - | - |
| AT3G50670 | 1.22E-05 | 0.896 | - | - |
| AT1G12360 | 1.77E-05 | 0.897 | - | - |
| AT4G28025 | 2.47E-05 | 0.898 | - | - |
| AT3G54670 | 1.48E-05 | 0.898 | - | - |
| AT3G01830 | 1.48E-05 | 0.898 | - | - |
| AT2G35930 | 1.06E-05 | 0.898 | - | - |
| ATMG00070 | 1.50E-05 | 0.899 | - | - |
| AT2G39260 | 1.49E-05 | 0.899 | - | - |
| AT2G47640 | 1.03E-05 | 0.899 | - | - |
| AT3G11520 | 1.61E-05 | 0.9 | - | - |
| AT5G04750 | 1.58E-05 | 0.9 | - | - |
| AT5G24840 | 1.26E-05 | 0.9 | - | - |
| AT5G45600 | 1.20E-05 | 0.9 | - | - |
| AT2G23250 | 1.02E-05 | 0.9 | - | - |
| AT4G30820 | 2.90E-05 | 0.901 | - | - |
| AT5G61150 | 2.19E-05 | 0.901 | - | - |
| AT1G79940 | 1.76E-05 | 0.901 | - | - |
| AT3G22480 | 1.44E-05 | 0.901 | - | - |
| AT5G38890 | 1.18E-05 | 0.901 | - | - |
| AT5G67270 | 1.79E-05 | 0.902 | - | - |
| AT5G45550 | 1.37E-05 | 0.902 | - | - |
| AT3G02840 | 1.47E-05 | 0.903 | - | - |
| AT5G57280 | 1.35E-05 | 0.903 | - | - |
| AT1G68730 | 1.17E-05 | 0.903 | - | - |
| AT5G66360 | 1.04E-05 | 0.903 | - | - |
| AT1G08620 | 1.02E-05 | 0.903 | - | - |
| AT2G45640 | 1.25E-05 | 0.904 | - | - |
| AT2G18000 | 1.10E-05 | 0.904 | - | - |
| AT1G05250 | 1.07E-05 | 0.905 | - | - |
| AT2G33340 | 1.84E-05 | 0.906 | - | - |
| AT1G08880 | 1.43E-05 | 0.906 | - | - |
| AT5G63120 | 1.13E-05 | 0.906 | - | - |
| AT5G50810 | 1.47E-05 | 0.907 | - | - |
| AT5G24440 | 1.17E-05 | 0.907 | - | - |
| AT1G74250 | 1.14E-05 | 0.907 | - | - |
| AT3G18580 | 1.10E-05 | 0.907 | - | - |
| AT2G23930 | 1.83E-05 | 0.908 | - | - |
| AT2G40540 | 1.31E-05 | 0.908 | - | - |
| AT4G23510 | 1.15E-05 | 0.908 | - | - |
| AT5G54080 | 3.21E-05 | 0.909 | - | - |
| AT3G11964 | 2.53E-05 | 0.909 | - | - |
| AT1G15440 | 2.28E-05 | 0.909 | - | - |
| AT3G15580 | 1.77E-05 | 0.909 | - | - |
| AT1G44900 | 1.52E-05 | 0.909 | - | - |
| AT5G43110 | 1.13E-05 | 0.909 | - | - |
| AT3G63130 | 2.53E-05 | 0.91 | - | - |
| AT1G56110 | 2.40E-05 | 0.91 | - | - |
| AT3G50930 | 1.78E-05 | 0.91 | - | - |
| AT1G10570 | 1.40E-05 | 0.91 | - | - |
| AT1G11400 | 1.26E-05 | 0.91 | - | - |
| AT1G22810 | 1.00E-05 | 0.91 | - | - |
| AT3G57940 | 1.91E-05 | 0.911 | - | - |
| AT4G00752 | 1.59E-05 | 0.911 | - | - |
| AT4G32850 | 1.31E-05 | 0.911 | - | - |
| AT5G42540 | 1.07E-05 | 0.911 | - | - |
| AT1G22740 | 1.04E-05 | 0.911 | - | - |
| AT2G17265 | 2.04E-05 | 0.912 | - | - |
| AT1G51690 | 1.66E-05 | 0.912 | - | - |
| AT2G18220 | 1.49E-05 | 0.912 | - | - |
| AT1G14400 | 1.34E-05 | 0.912 | - | - |
| AT3G60250 | 1.22E-05 | 0.912 | - | - |
| AT5G18130 | 1.15E-05 | 0.912 | - | - |
| AT4G33100 | 1.06E-05 | 0.912 | - | - |
| AT1G75850 | 1.46E-05 | 0.913 | - | - |
| AT1G78070 | 1.38E-05 | 0.913 | - | - |
| AT2G20142 | 1.30E-05 | 0.913 | - | - |
| AT1G03110 | 1.48E-05 | 0.914 | - | - |
| AT4G08360 | 1.16E-05 | 0.914 | - | - |
| AT5G09390 | 1.04E-05 | 0.914 | - | - |
| AT3G57660 | 2.38E-05 | 0.915 | - | - |
| AT1G14370 | 1.57E-05 | 0.915 | - | - |
| AT5G64660 | 1.56E-05 | 0.915 | - | - |
| AT5G57460 | 1.29E-05 | 0.915 | - | - |
| AT1G66740 | 2.24E-05 | 0.916 | - | - |
| AT5G12430 | 1.75E-05 | 0.916 | - | - |
| AT5G28740 | 1.70E-05 | 0.916 | - | - |
| AT3G20250 | 1.65E-05 | 0.916 | - | - |
| AT5G25930 | 1.42E-05 | 0.916 | - | - |
| AT3G11840 | 1.23E-05 | 0.916 | - | - |
| AT4G27640 | 1.13E-05 | 0.916 | - | - |
| AT3G54490 | 1.01E-05 | 0.916 | - | - |
| AT5G63960 | 4.22E-05 | 0.917 | - | - |
| AT1G54250 | 1.17E-05 | 0.917 | - | - |
| AT2G30760 | 1.14E-05 | 0.917 | - | - |
| AT3G62840 | 1.11E-05 | 0.917 | - | - |
| AT1G50260 | 1.09E-05 | 0.917 | - | - |
| AT1G71260 | 1.08E-05 | 0.917 | - | - |
| AT3G58250 | 1.08E-05 | 0.917 | - | - |
| AT3G23620 | 3.02E-05 | 0.918 | - | - |
| AT1G14620 | 1.75E-05 | 0.918 | - | - |
| AT5G52350 | 1.29E-05 | 0.918 | - | - |
| AT4G22720 | 1.23E-05 | 0.918 | - | - |
| AT3G20550 | 1.18E-05 | 0.918 | - | - |
| AT4G04770 | 1.09E-05 | 0.918 | - | - |
| AT3G54850 | 1.51E-05 | 0.919 | - | - |
| AT4G16520 | 1.40E-05 | 0.919 | - | - |
| AT3G21300 | 1.36E-05 | 0.919 | - | - |
| AT4G22140 | 1.51E-05 | 0.92 | - | - |
| AT2G24600 | 1.27E-05 | 0.92 | - | - |
| AT1G14680 | 1.11E-05 | 0.921 | - | - |
| AT5G35400 | 1.07E-05 | 0.921 | - | - |
| AT5G67630 | 1.37E-05 | 0.922 | - | - |
| AT5G42300 | 1.21E-05 | 0.922 | - | - |
| AT1G23710 | 1.21E-05 | 0.922 | - | - |
| AT3G61650 | 1.14E-05 | 0.922 | - | - |
| AT3G52250 | 1.09E-05 | 0.922 | - | - |
| AT4G01560 | 2.33E-05 | 0.923 | - | - |
| AT4G22970 | 1.45E-05 | 0.923 | - | - |
| AT1G77300 | 1.33E-05 | 0.923 | - | - |
| AT5G35910 | 1.26E-05 | 0.923 | - | - |
| AT1G75660 | 1.19E-05 | 0.923 | - | - |
| AT3G01520 | 1.13E-05 | 0.923 | - | - |
| AT2G03820 | 1.89E-05 | 0.924 | - | - |
| AT3G18860 | 1.26E-05 | 0.924 | - | - |
| AT2G23320 | 1.16E-05 | 0.924 | - | - |
| AT4G21660 | 1.12E-05 | 0.924 | - | - |
| AT5G53940 | 1.12E-05 | 0.924 | - | - |
| AT1G13290 | 1.08E-05 | 0.924 | - | - |
| AT5G57980 | 1.04E-05 | 0.924 | - | - |
| AT1G07000 | 2.03E-05 | 0.925 | - | - |
| AT2G47760 | 1.83E-05 | 0.925 | - | - |
| AT5G20320 | 1.54E-05 | 0.925 | - | - |
| AT3G02060 | 1.51E-05 | 0.925 | - | - |
| AT3G59700 | 1.39E-05 | 0.925 | - | - |
| AT1G03330 | 1.55E-05 | 0.926 | - | - |
| AT2G28450 | 1.07E-05 | 0.926 | - | - |
| AT3G12810 | 1.06E-05 | 0.926 | - | - |
| AT4G39670 | 1.20E-05 | 0.927 | - | - |
| AT3G25250 | 1.19E-05 | 0.927 | - | - |
| AT1G45976 | 1.34E-05 | 0.928 | - | - |
| AT2G03630 | 1.08E-05 | 0.928 | - | - |
| AT5G41190 | 1.52E-05 | 0.929 | - | - |
| AT3G56990 | 1.38E-05 | 0.929 | - | - |
| AT1G18800 | 1.32E-05 | 0.929 | - | - |
| AT5G02250 | 1.23E-05 | 0.929 | - | - |
| AT4G13570 | 1.03E-05 | 0.929 | - | - |
| AT5G14520 | 2.76E-05 | 0.93 | - | - |
| AT3G51730 | 1.69E-05 | 0.93 | - | - |
| ATCG00500 | 1.17E-05 | 0.93 | - | - |
| AT1G29690 | 1.01E-05 | 0.93 | - | - |
| AT1G61040 | 1.01E-05 | 0.93 | - | - |
| AT1G50030 | 2.76E-05 | 0.931 | - | - |
| AT3G20920 | 1.28E-05 | 0.931 | - | - |
| AT3G48900 | 1.01E-05 | 0.931 | - | - |
| AT1G31660 | 2.42E-05 | 0.932 | - | - |
| AT2G40360 | 2.27E-05 | 0.932 | - | - |
| AT3G49180 | 1.34E-05 | 0.932 | - | - |
| AT1G21690 | 1.30E-05 | 0.932 | - | - |
| AT2G27200 | 1.13E-05 | 0.932 | - | - |
| AT5G13270 | 1.09E-05 | 0.932 | - | - |
| AT2G28400 | 1.05E-05 | 0.932 | - | - |
| AT1G62980 | 1.01E-05 | 0.932 | - | - |
| AT3G10530 | 1.80E-05 | 0.933 | - | - |
| AT2G34570 | 1.49E-05 | 0.933 | - | - |
| AT3G57080 | 1.36E-05 | 0.933 | - | - |
| AT4G10670 | 1.20E-05 | 0.933 | - | - |
| AT5G67340 | 1.18E-05 | 0.933 | - | - |
| AT3G58330 | 1.05E-05 | 0.933 | - | - |
| AT3G02820 | 1.28E-05 | 0.934 | - | - |
| AT3G04820 | 1.16E-05 | 0.934 | - | - |
| AT1G01860 | 1.06E-05 | 0.934 | - | - |
| AT1G18390 | 1.32E-05 | 0.935 | - | - |
| AT1G65780 | 1.25E-05 | 0.935 | - | - |
| AT1G35510 | 1.07E-05 | 0.935 | - | - |
| AT3G01320 | 1.97E-05 | 0.936 | - | - |
| AT3G17410 | 1.61E-05 | 0.936 | - | - |
| AT1G52630 | 1.26E-05 | 0.936 | - | - |
| AT1G24140 | 1.19E-05 | 0.936 | - | - |
| AT3G02800 | 1.08E-05 | 0.936 | - | - |
| AT1G18370 | 1.05E-05 | 0.936 | - | - |
| AT1G76540 | 1.01E-05 | 0.936 | - | - |
| AT5G55300 | 1.27E-05 | 0.937 | - | - |
| AT1G10580 | 1.11E-05 | 0.937 | - | - |
| AT1G01880 | 1.08E-05 | 0.937 | - | - |
| AT5G44200 | 2.02E-05 | 0.938 | - | - |
| AT4G21980 | 1.43E-05 | 0.938 | - | - |
| AT2G41340 | 1.02E-05 | 0.938 | - | - |
| AT1G10490 | 1.31E-05 | 0.939 | - | - |
| AT2G32030 | 1.14E-05 | 0.939 | - | - |
| AT4G21110 | 1.12E-05 | 0.939 | - | - |
| AT2G40430 | 1.11E-05 | 0.939 | - | - |
| AT2G32600 | 2.44E-05 | 0.94 | - | - |
| AT2G34357 | 2.01E-05 | 0.94 | - | - |
| AT4G05190 | 1.21E-05 | 0.94 | - | - |
| AT1G11520 | 1.04E-05 | 0.94 | - | - |
| AT3G10050 | 3.49E-05 | 0.941 | - | - |
| AT1G50920 | 2.72E-05 | 0.941 | - | - |
| AT5G04990 | 1.93E-05 | 0.941 | - | - |
| AT2G25100 | 1.67E-05 | 0.941 | - | - |
| AT4G12120 | 1.35E-05 | 0.941 | - | - |
| AT2G36900 | 1.19E-05 | 0.941 | - | - |
| AT1G65030 | 1.07E-05 | 0.941 | - | - |
| AT5G64610 | 1.57E-05 | 0.942 | - | - |
| AT2G37430 | 1.16E-05 | 0.942 | - | - |
| AT1G12060 | 1.08E-05 | 0.942 | - | - |
| AT4G15770 | 2.36E-05 | 0.943 | - | - |
| AT5G61210 | 2.19E-05 | 0.943 | - | - |
| AT5G27120 | 1.78E-05 | 0.943 | - | - |
| AT2G17250 | 1.73E-05 | 0.943 | - | - |
| AT5G27420 | 1.46E-05 | 0.943 | - | - |
| AT2G42890 | 1.31E-05 | 0.943 | - | - |
| AT1G29990 | 1.07E-05 | 0.943 | - | - |
| AT1G78410 | 1.06E-05 | 0.943 | - | - |
| AT3G51280 | 1.19E-05 | 0.944 | - | - |
| AT2G39830 | 1.65E-05 | 0.945 | - | - |
| AT5G26920 | 1.46E-05 | 0.945 | - | - |
| AT3G62260 | 1.23E-05 | 0.945 | - | - |
| AT2G27690 | 1.19E-05 | 0.945 | - | - |
| AT2G23450 | 1.05E-05 | 0.945 | - | - |
| AT2G29570 | 4.21E-05 | 0.946 | - | - |
| AT3G14120 | 1.76E-05 | 0.946 | - | - |
| AT5G20600 | 1.43E-05 | 0.946 | - | - |
| AT2G21440 | 1.40E-05 | 0.946 | - | - |
| AT2G46400 | 1.17E-05 | 0.946 | - | - |
| AT5G67320 | 1.14E-05 | 0.946 | - | - |
| AT2G44510 | 1.12E-05 | 0.946 | - | - |
| AT5G13200 | 1.09E-05 | 0.946 | - | - |
| AT1G62040 | 1.05E-05 | 0.946 | - | - |
| AT2G29680 | 2.01E-05 | 0.947 | - | - |
| AT1G52740 | 1.80E-05 | 0.947 | - | - |
| AT2G31500 | 1.59E-05 | 0.947 | - | - |
| AT2G06990 | 1.38E-05 | 0.947 | - | - |
| AT5G62540 | 1.06E-05 | 0.947 | - | - |
| AT3G57150 | 2.69E-05 | 0.948 | - | - |
| AT5G03455 | 2.09E-05 | 0.948 | - | - |
| AT5G59950 | 1.84E-05 | 0.948 | - | - |
| AT3G28730 | 1.81E-05 | 0.948 | - | - |
| AT1G74360 | 1.69E-05 | 0.948 | - | - |
| AT3G04460 | 1.67E-05 | 0.948 | - | - |
| AT3G07050 | 1.55E-05 | 0.948 | - | - |
| AT1G48760 | 1.18E-05 | 0.948 | - | - |
| AT1G15420 | 1.13E-05 | 0.948 | - | - |
| AT2G15750 | 1.04E-05 | 0.948 | - | - |
| AT2G25830 | 7.04E-05 | 0.949 | - | - |
| AT1G79150 | 1.62E-05 | 0.949 | - | - |
| AT5G24590 | 1.35E-05 | 0.949 | - | - |
| AT3G28030 | 1.20E-05 | 0.949 | - | - |
| AT5G05110 | 1.02E-05 | 0.949 | - | - |
| AT1G01560 | 1.84E-05 | 0.95 | - | - |
| AT1G06720 | 1.33E-05 | 0.95 | - | - |
| AT5G63920 | 1.19E-05 | 0.95 | - | - |
| AT2G31970 | 2.04E-05 | 0.951 | - | - |
| AT2G32140 | 1.25E-05 | 0.951 | - | - |
| AT1G23860 | 1.25E-05 | 0.951 | - | - |
| AT5G20850 | 1.25E-05 | 0.951 | - | - |
| AT1G08840 | 1.10E-05 | 0.951 | - | - |
| AT1G43860 | 1.06E-05 | 0.951 | - | - |
| AT3G09720 | 1.05E-05 | 0.951 | - | - |
| AT3G13940 | 1.97E-05 | 0.952 | - | - |
| AT5G66540 | 1.38E-05 | 0.952 | - | - |
| AT1G13160 | 1.32E-05 | 0.952 | - | - |
| AT5G17820 | 1.21E-05 | 0.952 | - | - |
| AT3G42660 | 1.21E-05 | 0.952 | - | - |
| AT5G16780 | 1.20E-05 | 0.952 | - | - |
| AT1G15740 | 1.06E-05 | 0.952 | - | - |
| AT3G11730 | 1.02E-05 | 0.952 | - | - |
| AT5G50320 | 2.15E-05 | 0.953 | - | - |
| AT3G05060 | 2.01E-05 | 0.953 | - | - |
| AT2G07690 | 1.79E-05 | 0.953 | - | - |
| AT4G10710 | 1.39E-05 | 0.953 | - | - |
| AT2G46380 | 1.19E-05 | 0.953 | - | - |
| AT5G52760 | 1.13E-05 | 0.953 | - | - |
| AT1G22930 | 1.08E-05 | 0.953 | - | - |
| AT3G01160 | 1.04E-05 | 0.953 | - | - |
| AT1G48510 | 3.95E-05 | 0.954 | - | - |
| AT1G67500 | 3.92E-05 | 0.954 | - | - |
| AT1G49180 | 3.14E-05 | 0.954 | - | - |
| AT2G37560 | 2.82E-05 | 0.954 | - | - |
| AT3G13230 | 2.19E-05 | 0.954 | - | - |
| AT3G57000 | 1.81E-05 | 0.954 | - | - |
| AT5G02880 | 1.49E-05 | 0.954 | - | - |
| AT1G66260 | 1.25E-05 | 0.954 | - | - |
| AT5G47540 | 1.06E-05 | 0.954 | - | - |
| AT5G67100 | 4.50E-05 | 0.955 | - | - |
| AT5G04600 | 2.96E-05 | 0.955 | - | - |
| AT2G26350 | 1.76E-05 | 0.955 | - | - |
| AT1G18340 | 1.50E-05 | 0.955 | - | - |
| AT4G38780 | 1.37E-05 | 0.955 | - | - |
| AT1G08370 | 1.17E-05 | 0.955 | - | - |
| AT5G08180 | 2.88E-05 | 0.956 | - | - |
| AT4G28200 | 2.02E-05 | 0.956 | - | - |
| AT3G18600 | 1.46E-05 | 0.956 | - | - |
| AT3G12860 | 1.09E-05 | 0.956 | - | - |
| AT1G63020 | 2.18E-05 | 0.957 | - | - |
| AT5G40820 | 1.92E-05 | 0.957 | - | - |
| AT1G04510 | 1.77E-05 | 0.957 | - | - |
| AT5G15550 | 1.58E-05 | 0.957 | - | - |
| AT5G54260 | 1.54E-05 | 0.957 | - | - |
| AT5G15680 | 1.47E-05 | 0.957 | - | - |
| AT1G07270 | 1.39E-05 | 0.957 | - | - |
| AT1G26670 | 1.37E-05 | 0.957 | - | - |
| AT1G63810 | 1.20E-05 | 0.957 | - | - |
| AT5G13010 | 1.12E-05 | 0.957 | - | - |
| AT1G77470 | 1.03E-05 | 0.957 | - | - |
| AT1G59760 | 1.00E-05 | 0.957 | - | - |
| AT2G40030 | 2.54E-05 | 0.958 | - | - |
| AT3G16810 | 2.42E-05 | 0.958 | - | - |
| AT4G30825 | 1.94E-05 | 0.958 | - | - |
| AT3G60360 | 1.72E-05 | 0.958 | - | - |
| AT4G17760 | 1.47E-05 | 0.958 | - | - |
| AT3G21540 | 1.39E-05 | 0.958 | - | - |
| AT4G21180 | 1.38E-05 | 0.958 | - | - |
| AT5G16850 | 1.31E-05 | 0.958 | - | - |
| AT5G61330 | 1.24E-05 | 0.958 | - | - |
| AT2G26430 | 1.23E-05 | 0.958 | - | - |
| AT5G12190 | 1.05E-05 | 0.958 | - | - |
| AT5G40830 | 1.00E-05 | 0.958 | - | - |
| AT2G46080 | 1.00E-05 | 0.958 | - | - |
| AT1G23280 | 1.77E-05 | 0.959 | - | - |
| AT5G14050 | 1.52E-05 | 0.959 | - | - |
| AT1G18090 | 1.28E-05 | 0.959 | - | - |
| AT5G06370 | 1.15E-05 | 0.959 | - | - |
| AT5G41150 | 1.15E-05 | 0.959 | - | - |
| AT1G09770 | 1.11E-05 | 0.959 | - | - |
| AT3G17910 | 4.24E-05 | 0.96 | - | - |
| AT1G52930 | 2.68E-05 | 0.96 | - | - |
| AT4G02060 | 2.50E-05 | 0.96 | - | - |
| AT1G63780 | 1.99E-05 | 0.96 | - | - |
| AT4G25730 | 1.86E-05 | 0.96 | - | - |
| AT4G17270 | 1.60E-05 | 0.96 | - | - |
| AT5G54910 | 1.45E-05 | 0.96 | - | - |
| AT5G48600 | 1.11E-05 | 0.96 | - | - |
| AT4G37120 | 1.01E-05 | 0.96 | - | - |
| AT2G20050 | 6.32E-05 | 0.961 | - | - |
| AT3G19590 | 1.70E-05 | 0.961 | - | - |
| AT4G13530 | 1.39E-05 | 0.961 | - | - |
| AT1G60170 | 1.30E-05 | 0.961 | - | - |
| AT5G37055 | 1.27E-05 | 0.961 | - | - |
| AT1G10930 | 1.06E-05 | 0.961 | - | - |
| AT1G66500 | 1.05E-05 | 0.961 | - | - |
| AT2G16440 | 2.85E-05 | 0.962 | - | - |
| AT4G01360 | 1.24E-05 | 0.962 | - | - |
| AT1G65700 | 1.18E-05 | 0.962 | - | - |
| AT5G51880 | 1.05E-05 | 0.962 | - | - |
| AT4G04940 | 1.93E-05 | 0.963 | - | - |
| AT3G18524 | 1.83E-05 | 0.963 | - | - |
| AT4G05410 | 1.60E-05 | 0.963 | - | - |
| AT5G55920 | 1.29E-05 | 0.963 | - | - |
| AT5G27140 | 1.10E-05 | 0.963 | - | - |
| AT5G60990 | 1.06E-05 | 0.963 | - | - |
| AT1G73805 | 1.05E-05 | 0.963 | - | - |
| AT3G27230 | 1.43E-05 | 0.964 | - | - |
| AT3G62310 | 1.03E-05 | 0.964 | - | - |
| AT5G64420 | 1.85E-05 | 0.965 | - | - |
| AT1G16970 | 1.58E-05 | 0.965 | - | - |
| AT1G03190 | 1.53E-05 | 0.965 | - | - |
| AT5G08420 | 1.25E-05 | 0.965 | - | - |
| AT4G31210 | 1.23E-05 | 0.965 | - | - |
| AT2G25880 | 1.07E-05 | 0.965 | - | - |
| AT1G07370 | 4.23E-05 | 0.966 | - | - |
| AT2G24490 | 2.14E-05 | 0.966 | - | - |
| AT4G26600 | 1.26E-05 | 0.966 | - | - |
| AT2G19385 | 1.23E-05 | 0.966 | - | - |
| AT2G47420 | 1.07E-05 | 0.966 | - | - |
| AT3G17000 | 2.67E-05 | 0.967 | - | - |
| AT5G61770 | 1.59E-05 | 0.967 | - | - |
| AT4G19860 | 1.47E-05 | 0.967 | - | - |
| AT5G16630 | 1.75E-05 | 0.968 | - | - |
| AT5G57120 | 1.10E-05 | 0.969 | - | - |
| AT5G27740 | 1.05E-05 | 0.969 | - | - |
| AT5G09740 | 1.20E-05 | 0.97 | - | - |
| AT3G03920 | 2.24E-05 | 0.972 | - | - |
| AT3G51270 | 1.02E-05 | 0.974 | - | - |
| AT3G22660 | 1.56E-05 | 0.978 | - | - |
