# Supplementary material for: Identifying novel fruit-related genes in Arabidopsis thaliana based on the random walk with restart algorithm
Source: PLoS One. 2017 May 4;12(5):e0177017. doi: 10.1371/journal.pone.0177017 (PMC5417634; doi:10.1371/journal.pone.0177017)
Supplement: S3 Table — (DOCX) [file pone.0177017.s003.docx]

**S3 Table.** The 255 putative fruit-related genes.

| **ID** | **Probability** | **P-value** | **MIS** | **MFS** | **Tag ^a^** |
| --- | --- | --- | --- | --- | --- |
| AT5G37640 | 7.47E-05 | <0.001 | 946 | 0.999 | - |
| AT3G09790 | 7.49E-05 | <0.001 | 946 | 0.999 | - |
| AT4G05050 | 8.00E-05 | <0.001 | 946 | 0.999 | - |
| AT4G05320 | 8.37E-05 | <0.001 | 940 | 0.999 | - |
| AT1G36370 | 2.82E-05 | 0.001 | 994 | 0.997 | - |
| AT4G13890 | 3.11E-05 | 0.001 | 995 | 0.997 | - |
| AT1G22020 | 3.10E-05 | <0.001 | 994 | 0.997 | - |
| AT4G37840 | 3.08E-05 | 0.007 | 966 | 0.997 | - |
| AT3G60100 | 2.94E-05 | <0.001 | 980 | 0.997 | - |
| AT1G79530 | 2.98E-05 | 0.001 | 998 | 0.997 | - |
| AT5G42270 | 2.14E-05 | <0.001 | 956 | 0.996 | - |
| AT1G30380 | 3.05E-05 | <0.001 | 988 | 0.996 | - |
| AT3G61470 | 3.48E-05 | <0.001 | 962 | 0.996 | - |
| AT1G15820 | 2.90E-05 | <0.001 | 923 | 0.995 | + |
| AT3G47470 | 3.56E-05 | 0.014 | 973 | 0.995 | - |
| AT5G26780 | 3.60E-05 | <0.001 | 995 | 0.995 | - |
| AT3G21055 | 2.22E-05 | <0.001 | 942 | 0.995 | - |
| AT5G54270 | 3.00E-05 | <0.001 | 950 | 0.995 | - |
| AT5G08690 | 2.99E-05 | <0.001 | 998 | 0.994 | + |
| AT3G13860 | 2.19E-05 | 0.046 | 912 | 0.993 | - |
| AT5G02160 | 3.59E-05 | 0.001 | 933 | 0.992 | - |
| AT5G03240 | 7.56E-05 | <0.001 | 946 | 0.992 | - |
| AT1G79040 | 2.10E-05 | 0.004 | 977 | 0.991 | + |
| ATCG00120 | 2.84E-05 | 0.041 | 995 | 0.991 | - |
| AT2G34420 | 2.05E-05 | <0.001 | 957 | 0.99 | + |
| AT2G35040 | 4.66E-05 | 0.03 | 998 | 0.99 | - |
| AT1G14980 | 2.52E-05 | 0.006 | 971 | 0.99 | - |
| AT1G72610 | 5.54E-05 | <0.001 | 909 | 0.989 | - |
| AT1G72730 | 2.90E-05 | <0.001 | 925 | 0.989 | - |
| AT5G47030 | 3.05E-05 | 0.02 | 999 | 0.989 | + |
| AT4G39970 | 3.09E-05 | <0.001 | 904 | 0.988 | - |
| AT4G09010 | 4.40E-05 | <0.001 | 968 | 0.988 | - |
| AT1G15700 | 2.32E-05 | 0.001 | 991 | 0.988 | - |
| AT4G26530 | 7.17E-05 | <0.001 | 984 | 0.988 | - |
| AT2G05070 | 1.78E-05 | <0.001 | 920 | 0.987 | - |
| AT2G34430 | 1.86E-05 | 0.048 | 954 | 0.987 | - |
| AT5G23310 | 6.39E-05 | <0.001 | 973 | 0.986 | - |
| AT2G33040 | 4.17E-05 | 0.029 | 998 | 0.986 | + |
| AT5G61970 | 1.59E-05 | 0.016 | 949 | 0.986 | - |
| AT4G29480 | 1.18E-05 | 0.011 | 922 | 0.986 | - |
| AT4G38920 | 3.33E-05 | 0.018 | 980 | 0.986 | - |
| AT1G56070 | 4.71E-05 | <0.001 | 963 | 0.985 | - |
| AT2G22780 | 5.98E-05 | <0.001 | 982 | 0.985 | - |
| AT1G74470 | 3.80E-05 | <0.001 | 968 | 0.985 | - |
| AT1G67250 | 1.92E-05 | 0.041 | 975 | 0.984 | - |
| AT5G56500 | 1.72E-05 | <0.001 | 946 | 0.984 | - |
| AT5G18820 | 1.03E-05 | 0.046 | 919 | 0.984 | - |
| AT4G16155 | 3.47E-05 | <0.001 | 978 | 0.984 | - |
| AT4G26520 | 7.06E-05 | <0.001 | 984 | 0.984 | - |
| AT2G44350 | 4.79E-05 | <0.001 | 988 | 0.984 | - |
| AT1G71480 | 1.77E-05 | 0.03 | 923 | 0.984 | - |
| AT1G02920 | 2.09E-05 | 0.017 | 939 | 0.982 | + |
| AT1G15310 | 1.69E-05 | 0.041 | 971 | 0.982 | - |
| AT1G12250 | 3.36E-05 | <0.001 | 913 | 0.982 | - |
| AT5G08280 | 3.80E-05 | 0.016 | 988 | 0.982 | + |
| AT3G10970 | 2.75E-05 | 0.002 | 970 | 0.982 | - |
| AT5G50370 | 2.34E-05 | 0.023 | 940 | 0.982 | - |
| AT2G37250 | 1.44E-05 | 0.027 | 911 | 0.981 | - |
| AT3G03190 | 1.53E-05 | 0.045 | 946 | 0.981 | - |
| AT1G69820 | 1.54E-05 | 0.021 | 957 | 0.981 | + |
| AT5G49500 | 1.64E-05 | 0.007 | 971 | 0.981 | - |
| AT3G55040 | 2.69E-05 | <0.001 | 916 | 0.981 | + |
| AT2G25080 | 3.28E-05 | 0.003 | 906 | 0.98 | - |
| AT3G27820 | 1.89E-05 | 0.035 | 929 | 0.98 | - |
| AT4G25280 | 1.01E-05 | 0.025 | 943 | 0.98 | - |
| AT3G54900 | 2.84E-05 | 0.042 | 955 | 0.979 | - |
| AT3G58750 | 3.64E-05 | <0.001 | 980 | 0.979 | - |
| AT1G74030 | 3.74E-05 | <0.001 | 994 | 0.979 | + |
| AT5G28030 | 1.75E-05 | <0.001 | 907 | 0.979 | - |
| AT2G06520 | 2.87E-05 | <0.001 | 946 | 0.979 | - |
| AT2G29560 | 3.67E-05 | <0.001 | 987 | 0.979 | - |
| AT3G14290 | 3.65E-05 | 0.005 | 998 | 0.979 | - |
| AT5G40770 | 2.61E-05 | 0.02 | 929 | 0.979 | - |
| AT5G28020 | 2.09E-05 | 0.001 | 907 | 0.979 | - |
| AT2G30790 | 1.76E-05 | 0.008 | 936 | 0.979 | - |
| AT5G17220 | 3.08E-05 | 0.024 | 939 | 0.979 | + |
| AT1G48900 | 2.09E-05 | 0.003 | 971 | 0.979 | + |
| AT2G21160 | 2.56E-05 | 0.001 | 964 | 0.978 | - |
| AT3G60180 | 1.01E-05 | 0.021 | 943 | 0.978 | - |
| AT5G54770 | 3.17E-05 | <0.001 | 955 | 0.977 | - |
| AT3G59760 | 2.50E-05 | <0.001 | 906 | 0.977 | - |
| AT4G36530 | 1.67E-05 | 0.001 | 933 | 0.977 | - |
| AT5G23250 | 2.93E-05 | <0.001 | 997 | 0.977 | - |
| AT5G63510 | 1.42E-05 | 0.027 | 995 | 0.976 | - |
| AT4G02520 | 2.04E-05 | 0.001 | 939 | 0.976 | - |
| AT5G51820 | 6.48E-05 | <0.001 | 998 | 0.976 | + |
| AT1G29910 | 2.45E-05 | 0.001 | 945 | 0.976 | - |
| AT1G08380 | 3.07E-05 | 0.004 | 916 | 0.975 | - |
| AT2G39270 | 1.57E-05 | 0.033 | 911 | 0.974 | - |
| AT1G26230 | 2.84E-05 | 0.036 | 972 | 0.974 | - |
| AT4G27440 | 3.08E-05 | <0.001 | 955 | 0.974 | + |
| AT3G22110 | 5.48E-05 | <0.001 | 999 | 0.974 | - |
| AT5G43330 | 5.57E-05 | <0.001 | 972 | 0.974 | - |
| AT2G27020 | 5.96E-05 | 0.038 | 999 | 0.974 | - |
| AT3G25860 | 3.93E-05 | 0.001 | 975 | 0.974 | - |
| AT5G51100 | 6.01E-05 | 0.037 | 974 | 0.973 | - |
| AT3G06860 | 2.90E-05 | 0.005 | 995 | 0.972 | - |
| AT3G57560 | 1.62E-05 | 0.012 | 937 | 0.972 | - |
| AT3G03910 | 2.32E-05 | 0.022 | 965 | 0.972 | - |
| AT3G55400 | 4.57E-05 | 0.008 | 904 | 0.972 | - |
| AT1G31190 | 5.17E-05 | <0.001 | 963 | 0.972 | + |
| AT4G35760 | 1.37E-05 | 0.04 | 903 | 0.972 | - |
| AT1G34430 | 2.52E-05 | 0.008 | 994 | 0.972 | - |
| AT5G64380 | 4.90E-05 | <0.001 | 969 | 0.972 | - |
| AT1G73110 | 3.10E-05 | <0.001 | 971 | 0.971 | - |
| AT5G10920 | 2.55E-05 | <0.001 | 962 | 0.971 | - |
| AT1G48520 | 4.08E-05 | <0.001 | 903 | 0.97 | - |
| AT5G42790 | 3.92E-05 | 0.005 | 999 | 0.97 | - |
| AT1G75330 | 2.62E-05 | 0.001 | 958 | 0.97 | + |
| AT5G16390 | 2.83E-05 | 0.001 | 999 | 0.97 | - |
| AT2G42790 | 5.42E-05 | 0.002 | 997 | 0.97 | + |
| AT1G10670 | 3.04E-05 | <0.001 | 955 | 0.97 | - |
| AT5G08300 | 3.96E-05 | <0.001 | 999 | 0.969 | - |
| AT5G14590 | 2.32E-05 | <0.001 | 969 | 0.969 | - |
| AT3G13930 | 3.45E-05 | <0.001 | 993 | 0.969 | - |
| AT4G34700 | 2.00E-05 | 0.039 | 976 | 0.968 | - |
| AT5G44520 | 2.17E-05 | 0.002 | 971 | 0.968 | - |
| AT3G22630 | 2.63E-05 | 0.01 | 999 | 0.968 | - |
| AT1G77440 | 3.27E-05 | <0.001 | 999 | 0.968 | - |
| AT5G18800 | 2.54E-05 | 0.007 | 930 | 0.968 | - |
| AT5G55070 | 2.60E-05 | <0.001 | 998 | 0.967 | - |
| AT1G76730 | 3.76E-05 | <0.001 | 905 | 0.967 | - |
| AT2G02500 | 2.23E-05 | 0.046 | 999 | 0.967 | + |
| AT1G69740 | 3.01E-05 | 0.008 | 936 | 0.967 | + |
| ATCG00490 | 4.72E-05 | <0.001 | 993 | 0.965 | + |
| AT2G13360 | 4.83E-05 | 0.019 | 998 | 0.965 | - |
| AT1G56330 | 2.95E-05 | 0.018 | 953 | 0.965 | - |
| AT1G74040 | 1.91E-05 | <0.001 | 913 | 0.964 | - |
| AT3G09940 | 3.36E-05 | <0.001 | 991 | 0.963 | - |
| AT1G15950 | 2.21E-05 | 0.007 | 980 | 0.963 | - |
| AT2G43090 | 5.07E-05 | 0 | 992 | 0.962 | - |
| AT1G24180 | 2.20E-05 | 0.005 | 980 | 0.962 | - |
| AT5G49460 | 3.53E-05 | <0.001 | 969 | 0.962 | - |
| AT5G19760 | 2.64E-05 | <0.001 | 935 | 0.961 | - |
| AT3G03630 | 2.88E-05 | 0.006 | 907 | 0.961 | - |
| AT4G24830 | 3.37E-05 | 0.011 | 975 | 0.96 | + |
| AT1G07110 | 2.64E-05 | 0.001 | 962 | 0.96 | - |
| AT5G28840 | 2.47E-05 | 0.016 | 951 | 0.96 | - |
| AT3G22460 | 1.75E-05 | <0.001 | 906 | 0.96 | - |
| AT5G05690 | 3.20E-05 | <0.001 | 911 | 0.959 | + |
| AT1G01050 | 3.27E-05 | 0.007 | 940 | 0.959 | - |
| AT3G52200 | 2.57E-05 | <0.001 | 994 | 0.959 | - |
| AT2G19940 | 2.03E-05 | 0.009 | 997 | 0.959 | + |
| AT5G46420 | 2.18E-05 | 0.043 | 934 | 0.958 | - |
| AT2G43750 | 2.71E-05 | 0 | 907 | 0.958 | - |
| AT5G24240 | 7.72E-05 | <0.001 | 946 | 0.957 | - |
| AT1G46408 | 2.39E-05 | 0.043 | 901 | 0.957 | - |
| AT1G54220 | 2.02E-05 | <0.001 | 992 | 0.957 | - |
| ATCG00280 | 1.37E-05 | 0.034 | 963 | 0.956 | - |
| AT4G37040 | 3.09E-05 | 0.018 | 940 | 0.955 | + |
| AT1G12000 | 4.10E-05 | <0.001 | 962 | 0.955 | - |
| AT5G01650 | 4.48E-05 | 0.019 | 925 | 0.954 | + |
| AT4G26910 | 3.15E-05 | <0.001 | 998 | 0.954 | + |
| AT1G60810 | 1.63E-05 | <0.001 | 948 | 0.953 | - |
| AT1G20950 | 3.52E-05 | <0.001 | 947 | 0.953 | - |
| AT3G02090 | 4.99E-05 | 0.001 | 989 | 0.953 | - |
| AT5G65010 | 4.33E-05 | <0.001 | 992 | 0.952 | - |
| AT5G66760 | 3.18E-05 | 0.002 | 989 | 0.951 | - |
| AT2G44530 | 1.68E-05 | 0.036 | 922 | 0.951 | - |
| AT1G30120 | 3.69E-05 | 0.038 | 998 | 0.951 | - |
| AT2G21370 | 1.30E-05 | <0.001 | 914 | 0.95 | - |
| AT3G59480 | 1.37E-05 | 0.001 | 919 | 0.95 | - |
| AT2G04030 | 3.28E-05 | 0.017 | 917 | 0.95 | - |
| AT1G53090 | 1.61E-05 | 0.031 | 906 | 0.95 | - |
| AT1G51260 | 2.32E-05 | 0.016 | 913 | 0.95 | - |
| ATCG00020 | 1.92E-05 | 0.028 | 953 | 0.948 | - |
| AT1G06030 | 1.50E-05 | 0.009 | 919 | 0.948 | - |
| AT2G46340 | 2.85E-05 | 0.017 | 985 | 0.948 | - |
| AT4G22570 | 2.30E-05 | 0.019 | 909 | 0.948 | - |
| AT5G03650 | 4.37E-05 | <0.001 | 983 | 0.947 | - |
| AT1G66430 | 3.54E-05 | 0.001 | 919 | 0.947 | - |
| AT5G60600 | 3.67E-05 | 0.017 | 978 | 0.947 | - |
| AT5G50850 | 3.54E-05 | <0.001 | 999 | 0.947 | - |
| AT2G18230 | 3.75E-05 | 0.01 | 940 | 0.947 | - |
| AT3G10700 | 3.08E-05 | 0.003 | 912 | 0.946 | - |
| AT2G18450 | 1.95E-05 | 0.039 | 987 | 0.946 | - |
| AT1G09430 | 3.09E-05 | 0.001 | 951 | 0.946 | - |
| AT4G04040 | 2.50E-05 | 0.045 | 937 | 0.945 | - |
| AT4G09000 | 3.31E-05 | 0.001 | 940 | 0.945 | + |
| AT1G44446 | 3.68E-05 | 0.009 | 995 | 0.945 | - |
| AT5G09300 | 1.17E-05 | <0.001 | 971 | 0.944 | - |
| AT5G15800 | 1.83E-05 | 0.011 | 969 | 0.944 | - |
| ATCG00350 | 1.70E-05 | 0.013 | 938 | 0.944 | - |
| AT3G53620 | 3.28E-05 | 0.009 | 940 | 0.942 | - |
| AT5G58140 | 4.02E-05 | 0.016 | 935 | 0.942 | - |
| AT2G34590 | 2.35E-05 | <0.001 | 994 | 0.942 | + |
| AT3G56350 | 5.05E-05 | <0.001 | 973 | 0.941 | - |
| AT3G22960 | 4.73E-05 | 0.002 | 987 | 0.941 | + |
| AT5G08570 | 4.88E-05 | 0.001 | 987 | 0.941 | - |
| AT1G21720 | 3.29E-05 | <0.001 | 999 | 0.94 | - |
| AT3G01260 | 2.44E-05 | 0.014 | 939 | 0.94 | - |
| AT5G52920 | 4.67E-05 | <0.001 | 988 | 0.94 | + |
| AT1G06020 | 1.67E-05 | <0.001 | 931 | 0.939 | - |
| AT1G54340 | 3.86E-05 | <0.001 | 976 | 0.938 | - |
| AT4G25100 | 5.17E-05 | <0.001 | 982 | 0.937 | + |
| AT1G32440 | 4.53E-05 | <0.001 | 986 | 0.937 | - |
| AT4G17260 | 4.36E-05 | 0.001 | 918 | 0.937 | - |
| AT5G11160 | 1.96E-05 | 0.049 | 944 | 0.937 | - |
| AT5G25900 | 6.13E-05 | <0.001 | 992 | 0.936 | + |
| AT4G10260 | 1.47E-05 | <0.001 | 919 | 0.936 | + |
| AT3G55410 | 3.83E-05 | <0.001 | 998 | 0.935 | - |
| AT5G09600 | 2.08E-05 | 0.016 | 934 | 0.935 | - |
| AT3G03780 | 3.18E-05 | <0.001 | 998 | 0.934 | + |
| AT5G35550 | 1.92E-05 | 0.008 | 966 | 0.934 | - |
| AT5G05780 | 3.58E-05 | <0.001 | 999 | 0.934 | - |
| AT3G47800 | 3.13E-05 | <0.001 | 939 | 0.932 | - |
| AT5G35360 | 3.23E-05 | 0.027 | 994 | 0.932 | - |
| AT5G56350 | 6.56E-05 | <0.001 | 995 | 0.932 | - |
| AT1G60950 | 2.75E-05 | 0.015 | 911 | 0.932 | - |
| AT4G30580 | 3.56E-05 | 0.014 | 936 | 0.931 | - |
| AT2G36580 | 5.20E-05 | <0.001 | 990 | 0.929 | + |
| AT4G02570 | 5.42E-05 | 0.004 | 999 | 0.929 | + |
| AT3G55650 | 4.48E-05 | <0.001 | 987 | 0.928 | - |
| AT4G08390 | 2.10E-05 | 0.008 | 936 | 0.928 | + |
| AT3G04050 | 4.43E-05 | <0.001 | 986 | 0.928 | - |
| AT3G55810 | 4.43E-05 | <0.001 | 986 | 0.927 | - |
| AT3G25960 | 4.44E-05 | <0.001 | 986 | 0.927 | - |
| AT4G26390 | 2.79E-05 | 0.039 | 968 | 0.926 | - |
| AT1G32480 | 1.80E-05 | 0.033 | 978 | 0.923 | - |
| AT4G17830 | 1.54E-05 | <0.001 | 944 | 0.923 | - |
| AT5G63680 | 5.61E-05 | <0.001 | 988 | 0.922 | - |
| AT1G13060 | 5.28E-05 | 0.003 | 999 | 0.921 | - |
| AT1G18500 | 1.23E-05 | 0.021 | 913 | 0.92 | - |
| AT4G24620 | 6.39E-05 | 0 | 998 | 0.917 | + |
| AT5G13430 | 2.28E-05 | 0.045 | 989 | 0.917 | - |
| AT3G49160 | 3.05E-05 | <0.001 | 968 | 0.916 | - |
| AT1G12230 | 3.53E-05 | <0.001 | 996 | 0.916 | - |
| AT4G32210 | 1.70E-05 | 0.02 | 934 | 0.915 | - |
| AT4G35650 | 2.07E-05 | 0.02 | 978 | 0.915 | - |
| AT5G19530 | 1.86E-05 | 0.049 | 905 | 0.915 | - |
| AT3G17820 | 3.45E-05 | 0.002 | 985 | 0.913 | - |
| AT4G13430 | 4.73E-05 | 0.002 | 994 | 0.911 | - |
| AT4G29220 | 2.06E-05 | 0.044 | 974 | 0.911 | - |
| AT4G35260 | 3.57E-05 | <0.001 | 985 | 0.911 | - |
| AT4G39640 | 2.31E-05 | 0.018 | 965 | 0.91 | - |
| AT2G26300 | 4.71E-05 | 0.02 | 944 | 0.91 | - |
| AT4G35090 | 6.38E-05 | <0.001 | 993 | 0.909 | + |
| AT2G22480 | 2.74E-05 | <0.001 | 974 | 0.909 | - |
| AT2G31810 | 1.40E-05 | 0.026 | 945 | 0.909 | - |
| AT5G40760 | 2.68E-05 | 0.007 | 978 | 0.908 | - |
| AT2G17130 | 2.69E-05 | <0.001 | 979 | 0.908 | - |
| AT1G22170 | 2.37E-05 | 0.032 | 974 | 0.908 | - |
| AT1G65360 | 1.54E-05 | 0.038 | 920 | 0.908 | + |
| AT1G76030 | 2.86E-05 | <0.001 | 991 | 0.908 | + |
| AT5G56630 | 3.31E-05 | <0.001 | 974 | 0.907 | - |
| AT2G47450 | 3.61E-05 | 0.001 | 947 | 0.906 | - |
| AT2G43100 | 4.41E-05 | 0.007 | 994 | 0.904 | - |
| AT1G17290 | 3.03E-05 | <0.001 | 929 | 0.903 | - |
| AT5G13420 | 4.50E-05 | <0.001 | 996 | 0.903 | - |
| AT3G57050 | 2.10E-05 | 0.007 | 973 | 0.903 | - |
| AT3G58990 | 4.16E-05 | 0.003 | 992 | 0.902 | - |
| AT5G65750 | 3.36E-05 | <0.001 | 998 | 0.902 | - |
| AT1G78050 | 2.12E-05 | 0.029 | 906 | 0.901 | + |
| AT5G19550 | 5.70E-05 | 0 | 989 | 0.901 | + |
| AT4G15700 | 1.56E-05 | 0.025 | 923 | 0.901 | - |

a: This column indicates whether the gene was predicted in Zhu *et al.*’s study that used shortest path algorithm to search possible genes. “+” indicates that the gene was predicted in Zhu *et al.*’s study, while “-” indicates that the gene was not predicted in their study.
